# 5-Amino- and 6-amino-uracils in the synthesis of various heterocycles as therapeutic agents: a review

**DOI:** 10.1039/d5ra05123a

**Published:** 2025-10-20

**Authors:** Ashraf A. Aly, Esraa M. Osman, Sara M. Mostafa, Tarek M. Bedair, Mohamed Abd-Elmonem, Kamal Usef Sadek, Asmaa H. Mohamed

**Affiliations:** a Chemistry Department, Faculty of Science, Minia University 61519-El-Minia Egypt ashrafaly63@yahoo.com ashraf.shehata@mu.edu.eg esraamah33@gmail.com sara.ahmed@mu.edu.eg dr.tarek.bedair.2@mu.edu.eg m_chemistry4you@yahoo.com kusadek@yahoo.com asmaa.hamouda@mu.edu.eg

## Abstract

5-Amino- and 6-amino-uracil derivatives are used as precursors in the synthesis of various heterocyclic compounds, which have attracted great interest because of their potent biological activities and therapeutic uses. Multicomponent reactions (MCRs) are a quite significant green technique as they directly correlate with fewer byproducts as well as lower time and energy consumption. These benefits of MCRs expand their potential in the preparation of a variety of new catalytic systems for the synthesis of essential organic compounds in environmentally friendly reaction circumstances. Herein, we focus on some MCR sequences that evolved during the last decade, especially from 2014 to 2024, for the synthesis of target heterocycles starting from either 5-amino- or 6-amino-uracil derivatives. In addition, we discuss the mechanism by which the selected catalyst helps in the selectivity of the target molecules. Furthermore, the biological activity of the synthesized materials as therapeutic agents was reviewed.

## Abstract 2

5-Amino- and 6-amino-uracil derivatives are used as precursors in the synthesis of various heterocyclic compounds, which have attracted great interest because of their potent biological activities and therapeutic uses.

## Graphical abstract

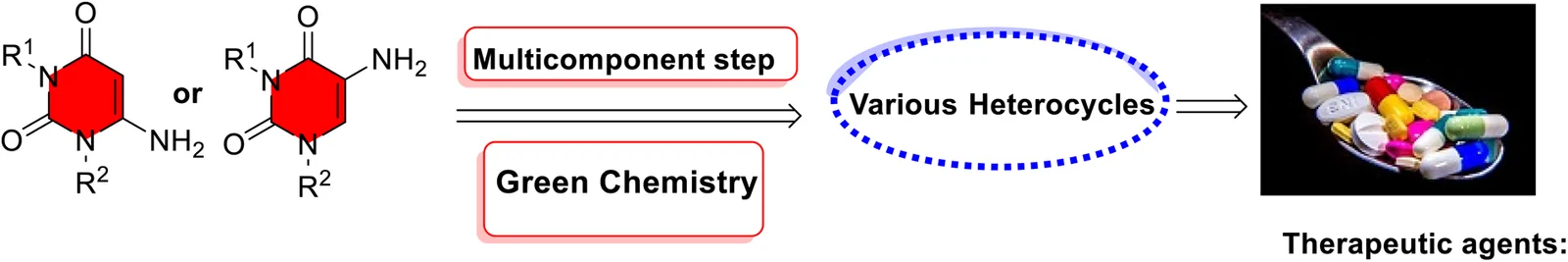

## 1 Introduction

**Fig. 1:**
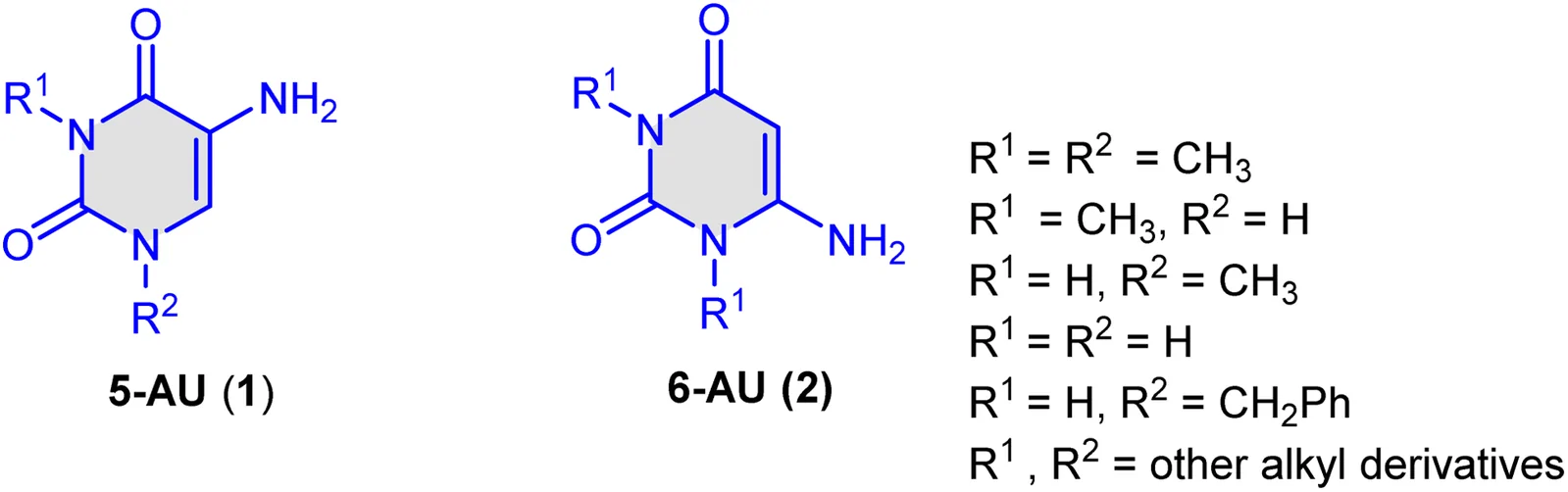
Structures of 5-amino- and 6-amino-uracil derivatives 1 and 2.

Uracil is a naturally occurring pyrimidine-based compound, which is considered one among the four nucleobases in RNA.^1–3^ The chemical characteristics of uracil and its derivatives, such as 5-aminouracils and 6-aminouracils (Fig. 1), vary because these molecules have the ability to function as both electrophiles and nucleophiles.^4^ Numerous bioactive chemical compounds can be synthesized using 5-aminouracils and/or 6-aminouracils as precursors.^5–8^ Because of their synthetic accessibility and a variety of biological actions, uracil derivatives are regarded as preferred structures in drug discovery.^9–14^ Particularly, 5- and 6-aminouracils and their derivatives have revealed various biological properties such as antioxidant,^15^ antimicrobial,^16,17^ anticancer,^18–20^ anti-Alzheimer^21^ and antiviral activity.^9,22^ Pyrido-, pyrrolo- and pyrimido-pyrimidines, phenazine, chromenes and xanthines of 5- and 6-aminouracils and their derivatives have been reported to be biologically active compounds.^23–38^

### 1.1. Synthesis of 5-amino- and 6-amino-uracil derivatives

Aminouracil-based compounds are effective ligands in chemical reactions and readily form metal complexes.^39–41^ In the presence of Cu(ii) and molecular oxygen, 5-aminouracil (1) underwent oxidation to form 5,5,6-trihydroxy-pyrimidine-2,4(1*H*,3*H*)-dione.^42^ During the wet chemical production of silver nanoparticles (AgNPs), 5-aminouracils were employed as a capping and reducing agent.^43,44^ Furthermore, 6-aminouracil (2) can be functionalized as it was sulfenylated through visible-light or electrochemical reactions^45^ in addition to thioarylation,^46^ thiocyanation,^47^ nitrosation^48^ and formylation.^49,50^ Despite the wide range of reactions involving amino uracil derivatives, chemists have shown great interest in green synthesis techniques,^51,52^ metal nanocatalysts,^53,54^ and catalyst free methods.^55,56^

Several approaches explaining the synthetic routes of 5-aminouracil (1) have been previously reported in the literature. The first methods dealt with the amination process of 5-halouracils using aqueous ammonia treatment, whereas the second method includes the reduction process of 5-nitrouracil^57–59^ with various reducing agents such as Sn/HCl,^24,60^ Zn/HCl,^23^ Zn ammonia solution, Fe_2_SO_4_, sodium thiolate (NaSH), or Al amalgam.^23,61,62^ Additionally, a sealed tube was used to heat the appropriate 5-bromo/nitro-methyl(aryl)-uracil with excess NH_3_ to obtain 5-amino-1-methyluracil and 5-amino-1-phenyluracil.^24^ The preparation of 5-aminouridines has also been demonstrated to be used in the biosynthesis of nucleic acids, which was accomplished by reacting either 5-bromouridine with ammonia^23,60–62^ or by reducing 5-nitrouridine.^63^

### 1.2. Some biological aspects of 5-amino- and 6-amino-uracil derivatives

**Fig. 2:**
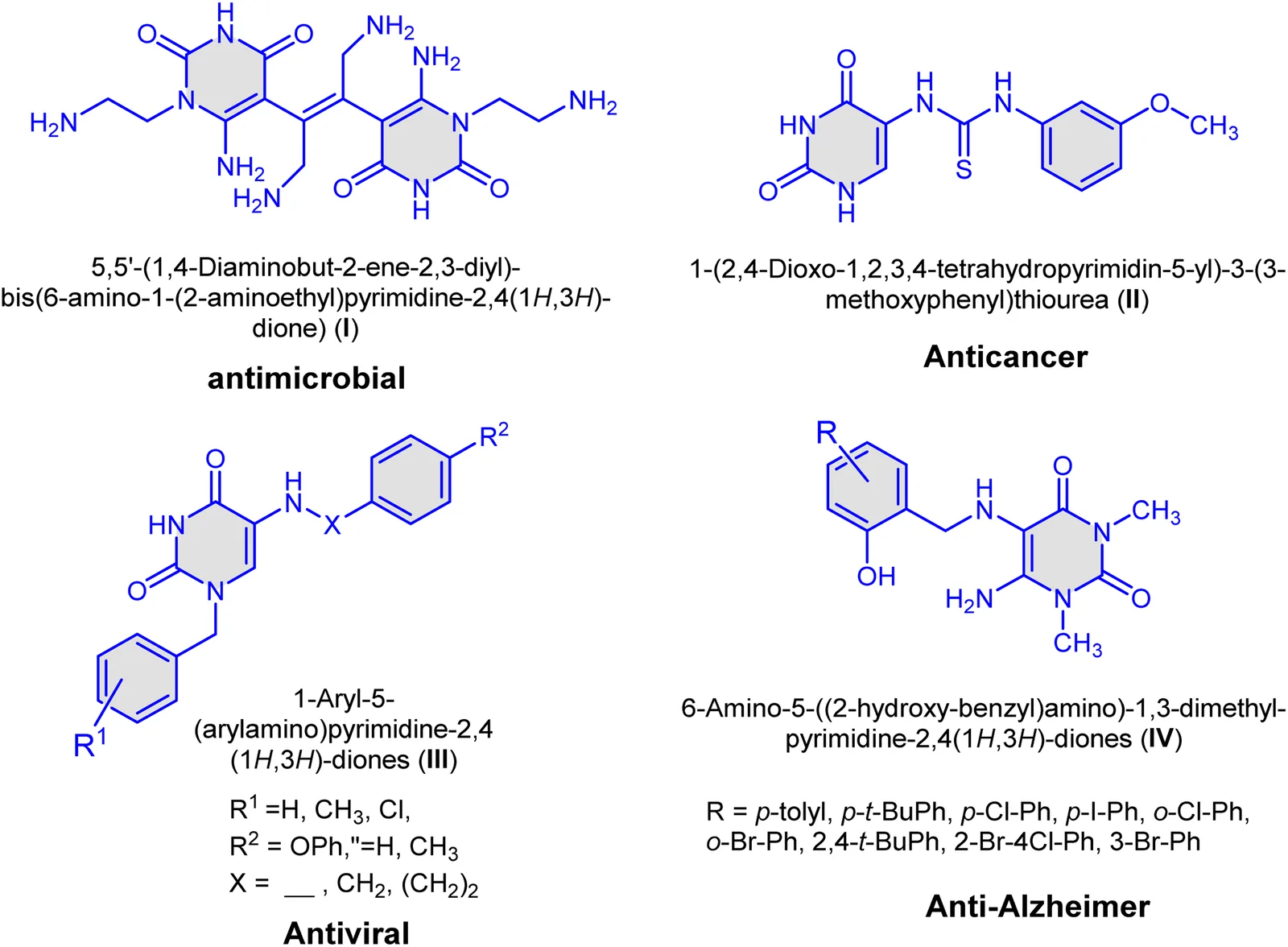
Examples of some biologically active aminouracil-based compounds I–IV.

Fig. 2 illustrates the most biologically active aminouracil compounds such as 5,5′-(1,4-diamino-but-2-ene-2,3-diyl)-bis(6-amino-1-(2-aminoethyl)pyrimidine-2,4-dione) (I, Fig. 2), which is known as an antimicrobial agent.^16^ The synthesized compound I revealed an inhibitory effect towards various bacterial strains. The inhibitory effect of the synthesized acyclic nucleoside I was more pronounced against Gram-positive bacteria (*B. cereus*, *B. subtilis*, and *S. aureus*) than against those of Gram-negative bacteria in comparison to the reference drug. The most susceptible bacterial species to the synthesized compound I was *B. cereus*, where the recorded MIC and MLC values were 32 and 64 μg mL^−1^, respectively, compared with 64 and 128 μg mL^−1^ for gentamicin, respectively.^16^

Moreover, 1-(2,4-dioxo-1,2,3,4-tetrahydropyrimidin-5-yl)-3-(3-methoxyphenyl)thiourea (II, Fig. 2) is known as an anticancer agent.^64^ Synthesis of compound II was achieved *via* the reaction of 5-aminouracil (1) with 3-methoxyphenyl isothiocyanate in refluxing methanol. Using the MTT assay and doxorubicin as the reference drug, compound II was tested for its antiproliferative activity against four human cancer cell lines: Panc-1 (pancreatic cancer cell line), MCF-7 (breast cancer cell line), HT-29 (colon cancer cell line), and A-549 (epithelial cancer cell line). The synthesized compound II had the main backbone assigned as the thio of uracil, which was tested with an IC_50_ value of 125 ± 11 nM.^64^

Both 1-aryl-5-(arylamino)pyrimidine-2,4(1*H*,3*H*)-diones (III, Fig. 2)^21^ and 6-amino-5-((2-hydroxy-benzyl)amino)-1,3-dimethyl-pyrimidine-2,4(1*H*,3*H*)-diones (IV, Fig. 2) were reported as antiviral and anti-Alzheimer agents, respectively.^22^

The synthesis of series III was achieved by the reaction of 2,4-bis(trimethylsilyloxy)-5-phenylaminouracil with benzyl bromides in refluxing 1,2-dichloroethane.^21^ The compounds were then subjected to screening across a broad range of viruses in order to evaluate their biological potential. Two of the compounds R^1^ = 3,5-Me, R^2^ = H, X = – and 3,5-Me, R^2^ = H, X = CH_2_ revealed promising inhibitory activity against HIV. A 50% protective effect was observed at concentrations of 11.9 and 9.5 μM, respectively, in the CEM-SS cell culture. It is noteworthy that both former compounds possess the same benzyl fragment, that is, a 3,5-dimethylphenylmethyl residue at the *N*−1 position of the uracil ring. It was found that the presence of the methyl substituents in the *m*-position of the benzyl fragment favorably affects the inhibitory properties of these compounds.^21^ In addition to the anti-HIV activity noted, several of the compounds also exhibited activity against the Epstein–Barr virus in the AKATA cell culture. The most active compound was 1-(3-phenoxybenzyl)-5-(phenylamino)uracil, with an IC_50_ value of 2.3 μM, and no toxicity was observed at a concentration of 100 μM. The second active compound was 1-(2-methylbenzyl)-5-(phenylamino)uracil, with an IC_50_ value of 12 μM.^21^

For series IV (Fig. 2), the synthesis was established by reductive amination, with moderate to good yields (30–84% yields). 5-(Arylidene)-6-aminouracils were *in situ* prepared *via* a condensation reaction between 5,6-diamino-1,3-dimethyluracil and substituted salicylaldehydes using an excess of sodium borohydride. The inhibitory abilities of uracil attached to benzylic amines were examined against acetylcholinesterase (AChE) and human carbonic anhydrase I and II (hCA I and II) isoenzymes, which were linked to some global disorders, such as Alzheimer's disease (AD), epilepsy, diabetes and glaucoma. The compounds displayed inhibition profiles with Ki values ranging from 2.28 ± 0.41 nM to 5.25 ± 0.75 nM for AChE, 36.10 ± 5.22–110.31 ± 54.81 nM for hCA I and 16.33 ± 4.91–72.03 ± 28.86 for hCA II.^22^

## 2 Discussion

In this review, we discuss the synthesis of heterocycles using 5-amino- and 6-amino-uracils over the past 10 years, especially from 2014 to 2024. We also provide more insight into the multi-component reaction in one-pot process pertaining to the synthesis of target compounds. Some reaction mechanisms are also covered. We also address the biological significance of target heterocycles as therapeutic agents.

### 2.1. Heterocycles from 5-aminouracil

5-Aminouracil and its building blocks behave as anticancer, antibacterial, and antiviral drugs.^19,20,65–68^ In 2016,^68^ a review article dealt with the synthesis and reactions of 5-aminouracil (1) and its derivatives. The review also dealt with a brief survey of biological activities, such as the chemotherapeutic and pharmacological activities of the announced uracil 1.

#### 2.1.1. Synthesis of pyrazolo-pyrimidine derivatives

**Scheme 1:**
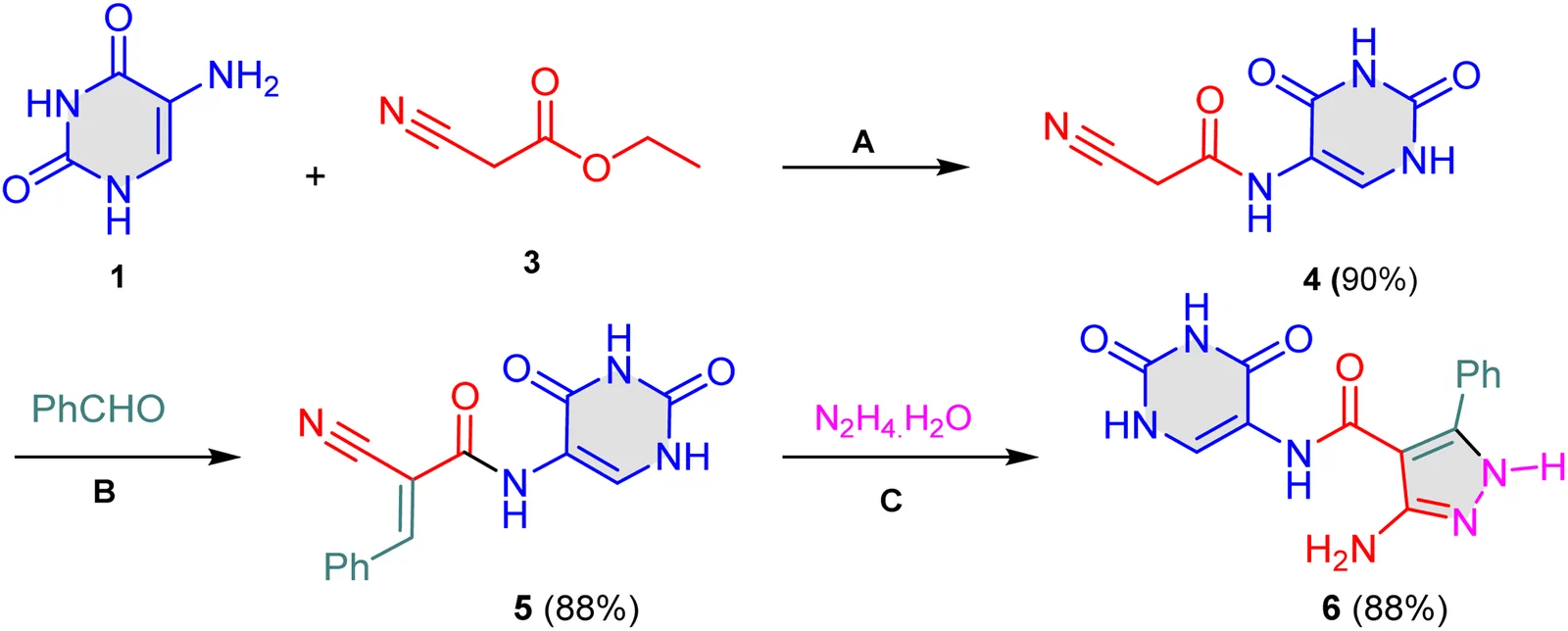
Synthesis of aminopyrazole 6. Reagents and conditions: A = neat, MW, 180 °C, 5 min. B = PhCHO, Pip, EtOH, MW. C = EtOH, MW, 130 °C, 10 min.

The reaction of 5-aminouracil (1) with ethyl cyanoacetate (3) under neat conditions under microwave (MW) irradiation for 5 min afforded 2-cyano-*N*-(2,4-dioxo-1,2,3,4-tetrahydropyrimidin-5-yl)acetamide (4) in 90% yield. Subsequently, the addition of benzaldehyde to ethanol using piperidine as the catalyst afforded the corresponding arylidine 5 in 88% yield (Scheme 1). Finally, the arylidine was led to react with hydrazine hydrate in ethanol (EtOH) under MW at 130 °C to produce 3-amino-*N*-(2,4-dioxo-1,2,3,4-tetrahydropyrimidin-5-yl)-5-phenyl-1*H*-pyrazole-4-carboxamide (6) in 88% yield (Scheme 1).^69^

**Scheme 2:**
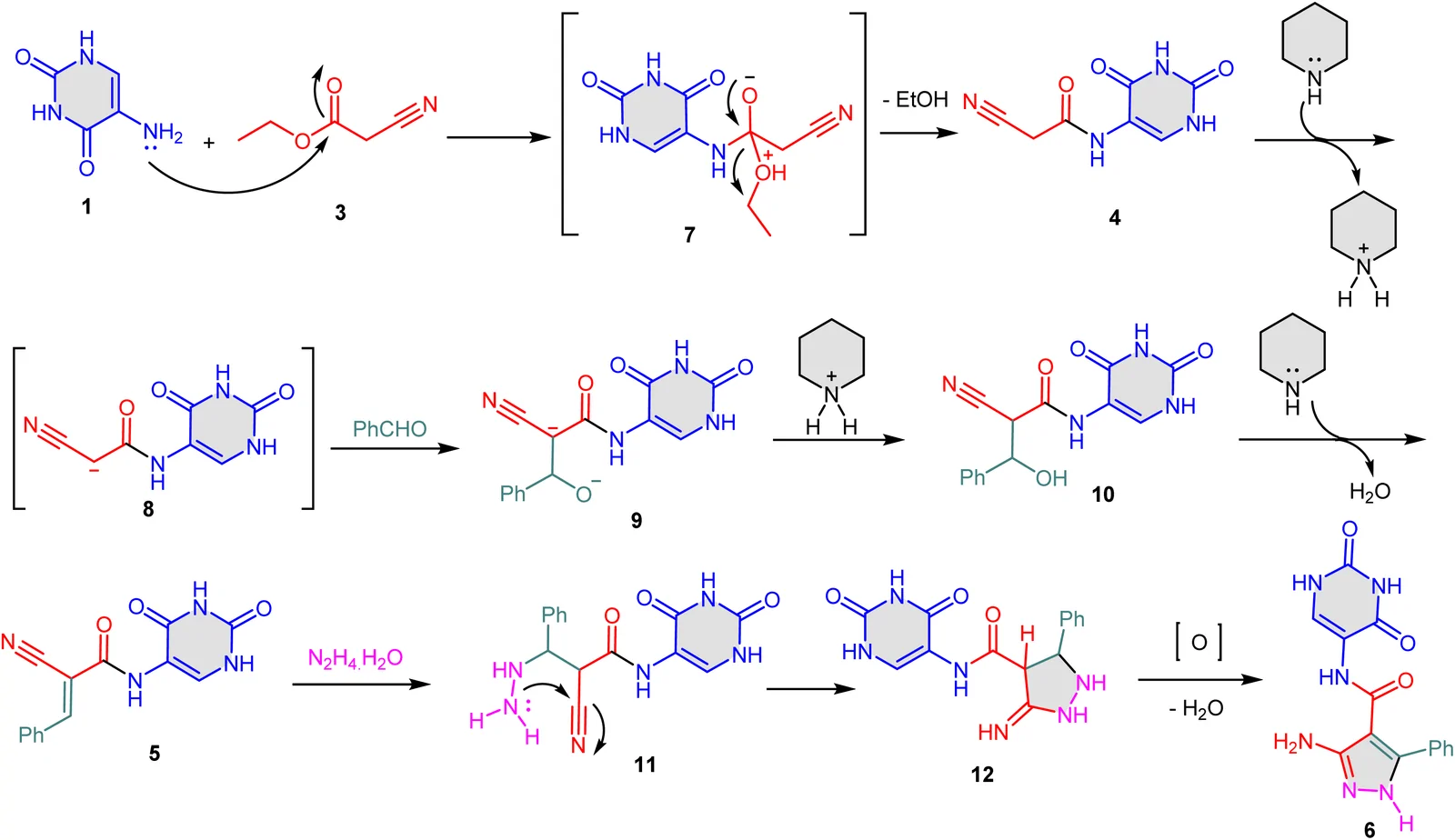
Proposed mechanism for the formation of aminopyrazole 6.

The mechanism described for the formation of 6 is shown in Scheme 2. First, the lone pair of nitrogen of 5-aminouracil (1) attacked the carbonyl group of ethyl cyanoacetate (3) to give intermediate 7 (Scheme 2). Subsequently, cyanoacetamide 4 was produced when intermediate 7 lost an ethanol molecule. Piperidine then abstracted a hydrogen proton from 7 to form the enol form 8. Next, the active methylene of intermediate 8 was added to the carbonyl of benzaldehyde to produce intermediate 9. The *E* isomer of compound 5 was then obtained when a water molecule was eliminated from adduct 10 and a piperidine molecule was recycled (Scheme 2). Finally, Michael's addition of hydrazine hydrate to the β-carbon of compound 5 afforded intermediate 11. Finally, an intramolecular cyclization of intermediate 11 would give intermediate 12, which underwent an autoxidation to give (pyrimidin-5-yl)-5-phenyl-1*H*-pyrazole-4-carboxamide 6 (Scheme 2).^69^

**Scheme 3:**
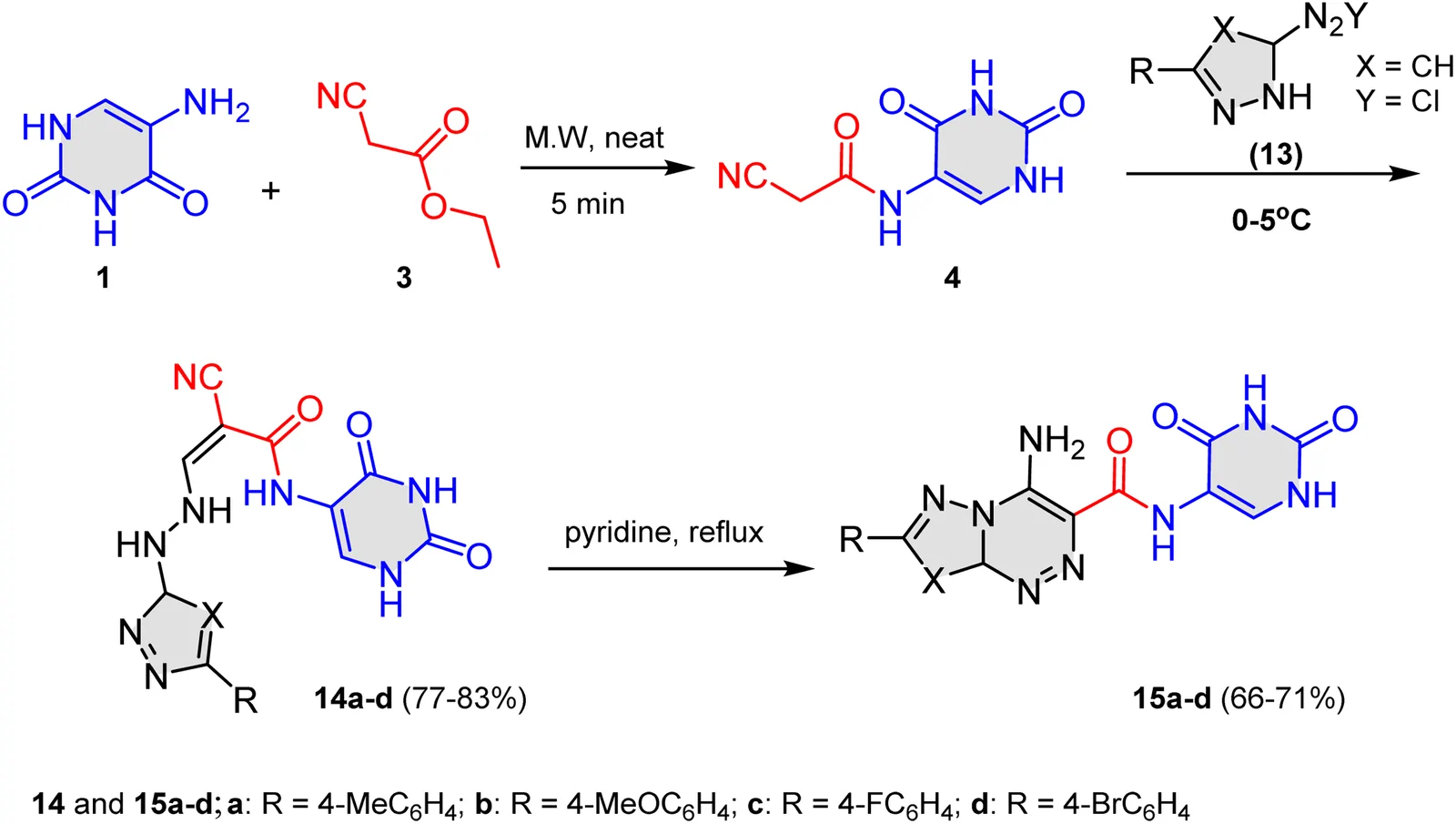
Synthesis of pyrazolo[5,1-*c*][1,2,4]triazine-3-carboxamides 15a–d.

Another work reported the synthesis of compounds 15a–d*via* the reaction of compound 4 with diazonium salt derivatives 13 to give the corresponding hydrazones 14a–d. Heating the hydrazones 14a–d in pyridine gave the final of pyrazolo[5,1-*c*][1,2,4]triazine-3-carboxamides 15a–d (Scheme 3).^66^ Compounds 15a–d were screened against Gram-positive and Gram-negative bacteria as well as four spore-forming fungal strains (Table 1). Compound 15c showed high activity against all strains because it exhibited activity against *Bacillus subtilis* (G+)Bs (IC_50_ = 23.2 ± 0.23 mm) compared with Ampicillin (IC_50_ = 32.4 ± 0.3 mm). Moreover, compound 15c revealed IC_50_ = 16.3 ± 0.15 against Ampicillin (IC_50_ = 23.8 ± 0.2) for *Streptococcus pneumoniae* (G+)Sp. However, compound 15a showed high activity against *Geotrichum candidum* (Gc) with IC_50_ = 19.9 ± 0.3 compared with Amphotericin B (IC_50_ = 25.4 ± 0.1). Table 1 illustrates the activities of compounds 15a–d against antibacterial Gram (+ve), Gram (−ve) and fungal microorganisms. Methoxy substituent 15c exhibited the most active compound as a promising antimicrobial and anti-fungal agent.

**Table 1:** Antimicrobial and antifungal screening of compounds 15a–d^a^

Compd	*Bacillus subtilis*	*Streptococcus pneumoniae*	*Escherichia coli*	*Pseudomonas aeruginosa*	*Aspergillus flavus* (fungus) Af	*Syncephalastrum racemosum* (Sr)	*Geotrichum candidum* (Gc)
	(G+) Bs	(G+) Sp	(G−) Ec	(G−) Pa			
15a	21.3 ± 0.12	15.2 ± 0.23	11.3 ± 0.12	10.6 ± 0.09	13.5 ± 0.13	15.5 ± 0.11	19.8 ± 0.19
15b	17.2 ± 0.26	10.2 ± 0.29	10.02 ± 0.11	8.9 ± 0.12	11.4 ± 0.14	13.6 ± 0.13	17.6 ± 0.21
15c	23.2 ± 0.23	16.3 ± 0.15	11.6 ± 0.09	10.9 ± 0.15	14.2 ± 0.09	18.5 ± 0.06	15.7 ± 0.25
15d	15.2 ± 0.33	12.3 ± 0.12	9.8 ± 0.08	9.8 ± 0.17	11.3 ± 0.08	11.2 ± 0.08	12.4 ± 0.19
Amphotericin B	—	—			23.7 ± 0.1	28.7 ± 0.2	25.4 ± 0.1
Ampicillin	32.4 ± 0.3	23.8 ± 0.2	—	—	—	—	—
Gentamicin	—	—	19.9 ± 0.3	17.3 ± 0.1	—	—	—

a Screening organisms, Mold: *A. flavus* (RCMB 02568, Af); *Syncephalastrum racemosum* (RCMB, 016 001, Sr) and *Geotrichum candidum* (RCMB, 052 006 Gc); two Gram-positive bacteria: *S. pneumoniae* (RCMB 010010, Sp) and *B. subtilis* (RCMB 010069, Bs); two Gram-negative bacteria: *E. coli* (RCMB 010052, Ec) and *P. aeruginosa* (RCMB 004, Pa); inhibition zone (IZ): high activity >15 (mm); moderate activity 11–14 (mm); slight activity 8–10 (mm) and non-sensitive 0–7 (mm).

#### 2.1.2. Synthesis of thiazolo-pyrimidines

**Scheme 4:**
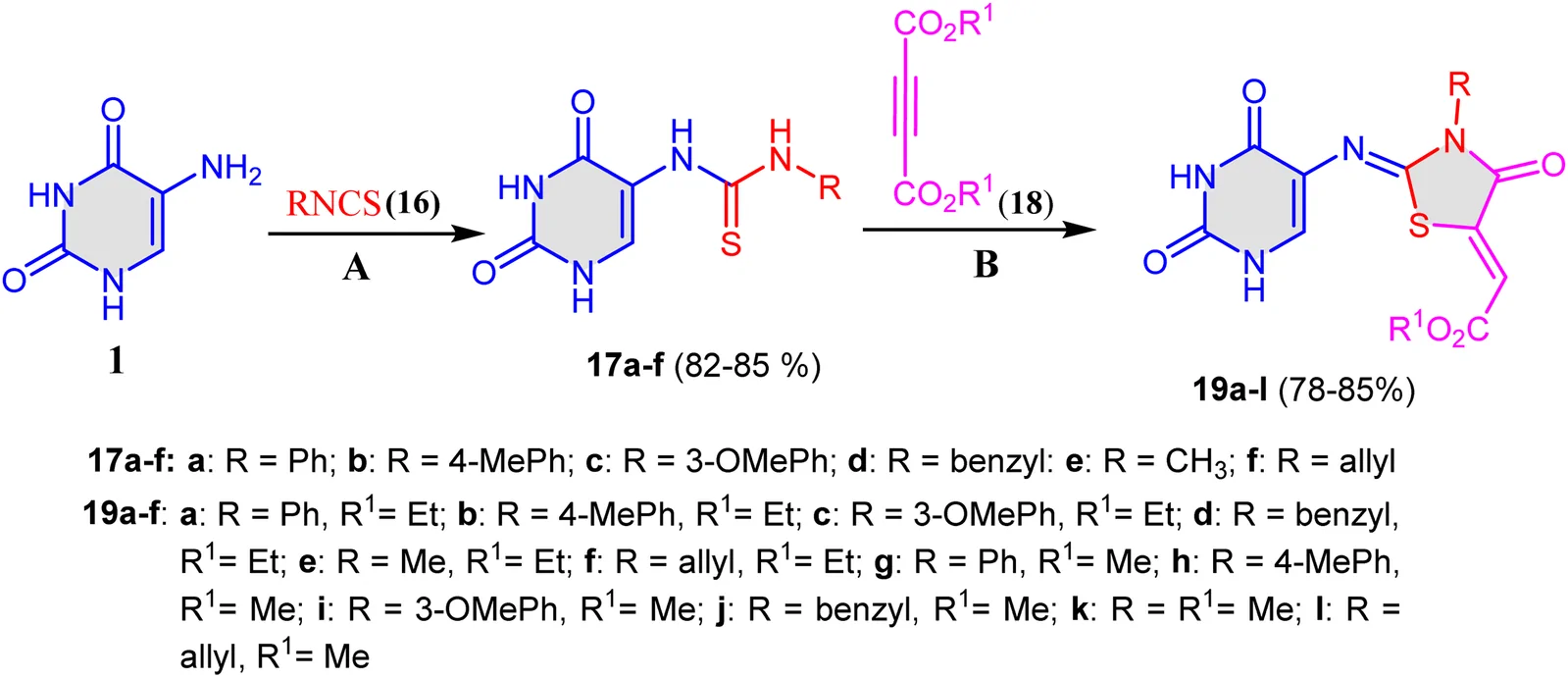
Synthesis of uracil–thiazoles hybrid molecules 19a–l. Reagents and conditions: A = MeOH, reflux, Et_3_N, 10–12 h. B; EtOH, reflux, 6–12 h.

Aly *et al.*^64^ reported the synthetic route of uracil-thios 17a–f by refluxing 5-aminouracil (1) with different isothiocyanates (16) in methanol (MeOH) and triethylamine (Et_3_N) (Scheme 4). Thereafter, thio derivatives 17a–f were reacted with acetylene dicarboxylate derivatives 18a, b in EtOH to produce thiazolidin-4-ones 19a–l in good yields (78–85%). The IC_50_ values for the synthesized compounds ranged from 1.1 μM to 10 μM, indicating an antiproliferative action (Table 2). Compounds 17c, 17b, 19c, 19h, 19i, and 19j were the most effective derivatives, with IC_50_ values ranging from 1.1 μM to 1.8 μM. Compound 19j displayed the most potent activity, with an IC_50_ value of 1.1 μM, in comparison to the reference doxorubicin (IC_50_ = 1.1 μM) and was even more potent than doxorubicin against A-549 and Panc-1 cancer cell lines. The six most potent antiproliferative derivatives (17c, 19b, 19c, 19h, 19i, and 19j) were further investigated against EGFR as potential targets for their antiproliferative activity (Table 3). Compound 19b displayed potent inhibitory activity against EGFR and BRAFV^600E^ with IC_50_ values of 91 ± 0.7 and 93 ± 0.8 μM, respectively, indicating that this compound could act as a dual inhibitor of EGFR and BRAFV^600E^ with significant antiproliferative activities.^64^

**Table 2:** Antiproliferative properties of compounds 17a–f and 19a–l

Compd		Cell viability%	Antiproliferative activity IC_50_ ± SEM (nM)				
			A-549	MCF-7	Panc-1	HT-29	Average
17a	R = Ph	86	8.90 ± 0.80	8.50 ± 0.80	8.80 ± 0.80	9.10 ± 0.80	8.80
17b	R = 4-MePh	87	3.70 ± 0.30	3.60 ± 0.30	4.10 ± 0.30	3.90 ± 0.30	3.80
17c	R = 3-OMePh	89	1.80 ± 0.20	1.40 ± 0.10	2.10 ± 0.20	2.10 ± 0.20	1.85
17d	R = benzyl	91	4.10 ± 0.40	3.90 ± 0.40	4.30 ± 0.40	4.30 ± 0.40	4.15
17e	R = Me	89	9.70 ± 0.80	9.60 ± 0.80	9.80 ± 0.80	10.80 ± 0.90	10.0
17f	R = allyl	89	3.80 ± 0.30	3.70 ± 0.30	3.90 ± 0.30	4.10 ± 0.30	3.90
19a	R = Ph, R^1^ = Et	91	3.50 ± 0.30	3.10 ± 0.30	3.30 ± 0.30	3.90 ± 0.30	3.45
19b	R = 4-MePh, R^1^ = Et	92	1.20 ± 0.10	1.10 ± 0.10	1.40 ± 0.10	1.40 ± 0.10	1.30
19c	R = 3-MeOPh, R^1^ = Et	96	1.50 ± 0.10	1.60 ± 0.10	1.90 ± 0.20	1.80 ± 0.10	1.70
19d	R = benzyl, R^1^ = Et	86	4.90 ± 0.50	4.70 ± 0.40	5.50 ± 0.50	5.50 ± 0.50	5.15
19e	R = Me, R^1^ = Et	86	7.20 ± 0.60	6.70 ± 0.70	7.30 ± 0.70	7.20 ± 0.70	7.10
19f	R = allyl, R^1^ = Et	89	8.20 ± 0.70	7.90 ± 0.70	8.80 ± 0.70	8.90 ± 0.70	8.50
19g	R = Ph, R^1^ = Me	87	2.70 ± 0.20	2.20 ± 0.20	2.90 ± 0.20	2.20 ± 0.20	2.50
19h	R = 4-MePh, R^1^ = Me	92	1.40 ± 0.10	1.70 ± 0.10	1.80 ± 0.10	1.70 ± 0.10	1.65
19i	R = 3-MeOPh, R^1^ = Me	89	1.30 ± 0.10	1.00 ± 0.08	1.50 ± 0.10	1.60 ± 0.10	1.35
19j	R = benzyl, R^1^ = Me	89	1.10 ± 0.10	0.90 ± 0.10	1.20 ± 0.10	1.20 ± 0.10	1.10
19k	R = R^1^ = Me	89	5.70 ± 0.60	5.10 ± 0.50	5.90 ± 0.50	6.20 ± 0.60	5.70
19l	R = allyl, R^1^ = Me	86	6.00 ± 0.60	6.50 ± 0.60	6.40 ± 0.60	6.60 ± 0.60	6.40
**Doxorubicin**		**—**	**1.20** ± **0.10**	**0.90** ± **0.10**	**1.40** ± **0.10**	**1.00** ± **0.10**	**1.10**

**Table 3:** IC_50_ of compounds 17c, 19b, 19c, 19h, 19i, and 19j against EGFR and BRAFV^600E^

Compd	EGFR inhibition	BRAF^V600E^ inhibition
	IC_50_ ± SEM (nM)	IC_50_ ± SEM (nM)
17c	125 ± 11	148 ± 12
19b	91 ± 0.7	93 ± 0.8
19c	115 ± 10	107 ± 10
19h	112 ± 10	137 ± 12
19i	96 ± 0.7	122 ± 12
19j	87 ± 0.5	115 ± 12
**Erlotinib**	**80** ±**0.5**	**60** ±**0.5**

##### 2.1.2.1 Structure activity relationship

**Fig. 3:**
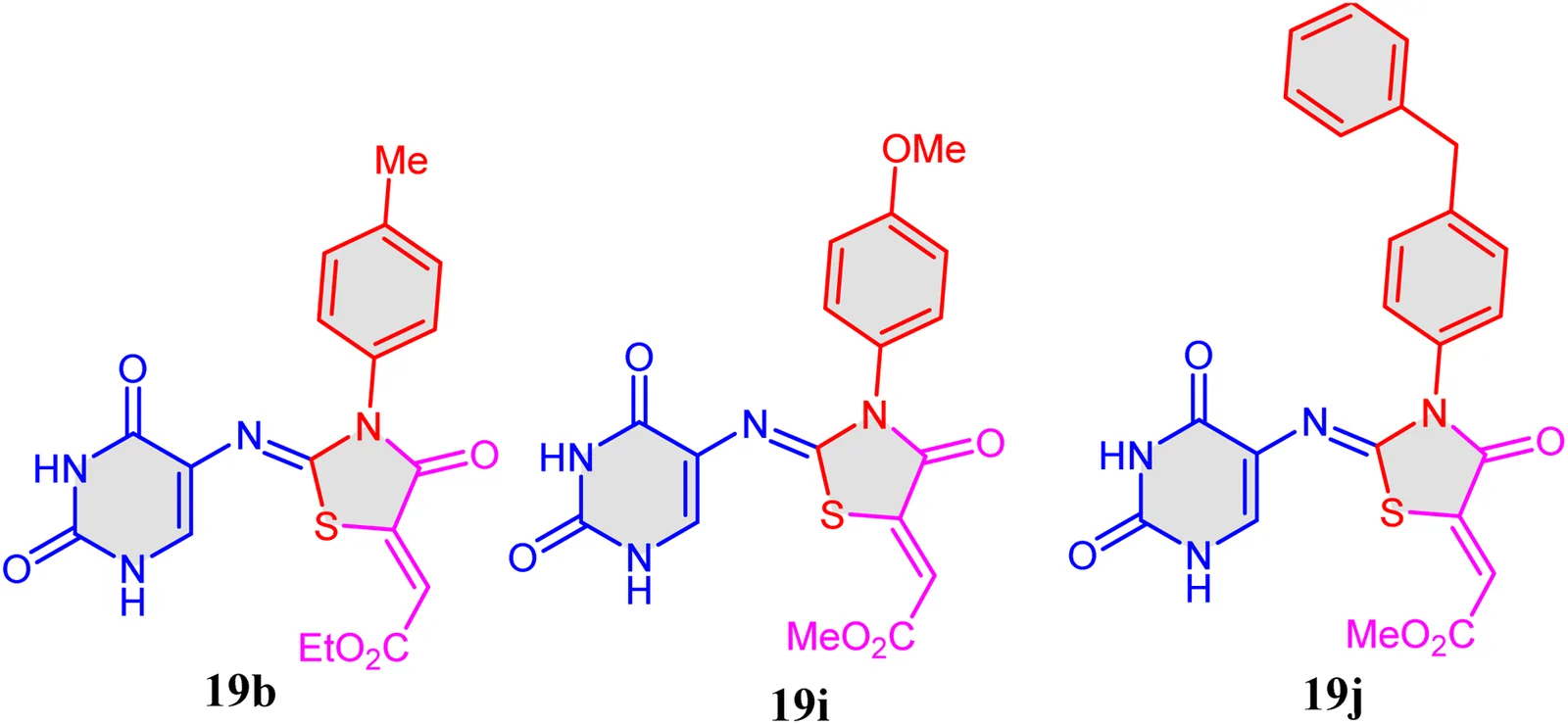
Structure of antiproliferative uracil–thiazolidin-4-one derivatives 19b, 19i and 19i.

The data in Tables 2 and 3 show the findings of the antiproliferative assay, in which compound 19j (R = benzyl, R^1^ = Me; thiazolidin-4-one) was found to be the most potent antiproliferative and demonstrated the highest inhibitory activity against EGFR with an IC_50_ value of 87 ± 05 nM, which is very close to that of the reference erlotinib (IC_50_ = 80 ± 05 nM). Compounds 19b (R = 4-CH_3_-Ph, R^1^ = Et; thiazolidin-4-one) and 19i (R = 3-OCH_3_-Ph, R^1^ = Me; thiazolidin-4-one) rank second and third in activity, with IC_50_ values of 91 ± 07 nM and 97 ± 07 nM, respectively. Based on the results of this assay, compounds 19b, 19i, and 19j (Fig. 3) showed promising antiproliferative activities and have the potential to act as EGFR inhibitors.

Compound 19b (R = 4-CH_3_-Ph, R^1^ = Et; thiazolidin-4-one) was the most potent derivative as BRAF^V600E^ inhibitor with an IC_50_ value of 93 ± 08 nM, indicating that this compound could behave as a dual inhibitor of EGFR and BRAFV^600E^ with promising antiproliferative properties. It can be concluded that the presence of ethyl ester together with an *N*-aromatic system attached to an electron donating methyl group, as in 19b, would increase the antiproliferative activity in the series of 19a–l.

**Scheme 5:**
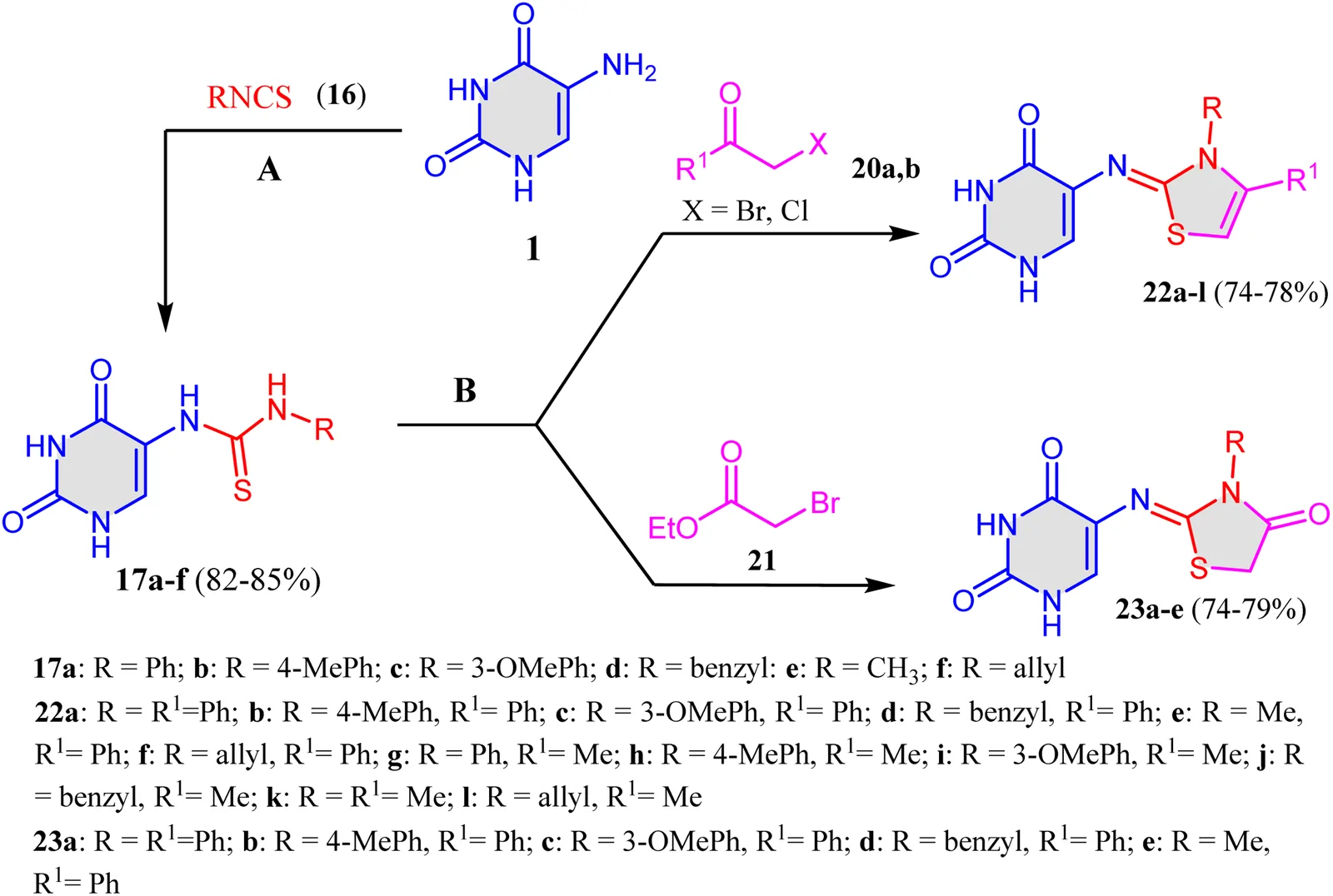
Synthesis of uracil–thiazole 22a–l and 23a–e building blocks. Reagents and conditions: A = MeOH, reflux, 10–12 h. B; EtOH, Et_3_N, reflux.

Correspondingly, Aly *et al.*^70^ reacted thioureas derived from 5-aminouracil 17a–f with α-halo-acetophenones 20a,b, and ethyl bromoacetate (21) in EtOH using Et_3_N as a base catalyst to obtain uracil–thiazolidene derivatives 22a–l and uracil–thiazoliden-4-one hybrids 23a–e, respectively (Scheme 5).^70^ The cell viability of compounds was evaluated using the normal human mammary gland epithelial (MCF-10A) cell line. None of the compounds under investigation showed cytotoxic effects, with cell viability higher than 87% when investigated at 50 μM (Table 4). When tested against four human cancer cell lines (A-549, MCF-7, Panc-1, and HT-29), the antiproliferative properties of 22a–l and 23a–e demonstrated significant effects when compared to doxorubicin (IC_50_ = 1.1 μM). It was found that the most effective derivatives were compounds 22a, 22c, 22f, 22i, and 23b, with IC_50_ values ranging from 0.9 mM to 1.7 mM against the four cancer cell lines.

**Table 4:** IC_50_ values of compounds 22a–l and 23a–e against four cancer cell lines

Compd	Cell viability%	Antiproliferative activity IC_50_ ± SEM (mM)				
		A-549	MCF-7	Panc-1	HT-29	Average (GI_50_)
22a	90	1.20 ± 0.20	1.10 ± 0.10	1.40 ± 0.20	1.30 ± 0.20	1.25
22b	89	2.70 ± 0.30	2.60 ± 0.30	3.10 ± 0.30	2.90 ± 0.30	2.80
22c	91	0.80 ± 0.10	0.90 ± 0.10	1.00 ± 0.10	0.90 ± 0.10	0.90
22d	91	2.00 ± 0.20	1.90 ± 0.20	2.30 ± 0.20	2.20 ± 0.20	2.10
22e	87	2.90 ± 0.30	2.80 ± 0.30	3.30 ± 0.30	3.20 ± 0.30	3.05
22f	89	1.80 ± 0.20	1.60 ± 0.10	2.10 ± 0.20	2.10 ± 0.20	1.90
22g	91	2.30 ± 0.20	2.10 ± 0.20	2.50 ± 0.20	2.40 ± 0.20	2.30
22h	92	3.30 ± 0.30	3.10 ± 0.30	3.50 ± 0.30	3.60 ± 0.30	3.40
22i	89	1.10 ± 0.10	1.00 ± 0.10	1.30 ± 0.10	1.20 ± 0.10	1.15
22j	87	6.20 ± 0.60	6.00 ± 0.60	6.50 ± 0.60	6.40 ± 0.60	6.30
22k	90	4.80 ± 0.50	4.70 ± 0.40	4.90 ± 0.50	4.90 ± 0.50	4.80
22l	87	5.50 ± 0.60	5.30 ± 0.50	5.80 ± 0.60	5.70 ± 0.60	5.60
23a	92	2.40 ± 0.20	2.30 ± 0.20	2.80 ± 0.20	2.70 ± 0.20	2.55
23b	90	1.50 ± 0.10	1.60 ± 0.10	1.80 ± 0.20	1.80 ± 0.20	1.70
23c	91	1.40 ± 0.10	1.30 ± 0.10	1.60 ± 0.10	1.50 ± 0.10	1.45
23d	87	7.70 ± 0.70	7.50 ± 0.70	7.80 ± 0.80	7.90 ± 0.80	7.70
23e	89	7.00 ± 0.60	6.80 ± 0.70	7.30 ± 0.70	7.20 ± 0.70	7.10
Doxorubicin	—	1.20 ± 0.10	0.90 ± 0.10	1.40 ± 0.10	1.00 ± 0.10	1.10

##### 2.1.2.2 Structure activity relationship

**Fig. 4:**
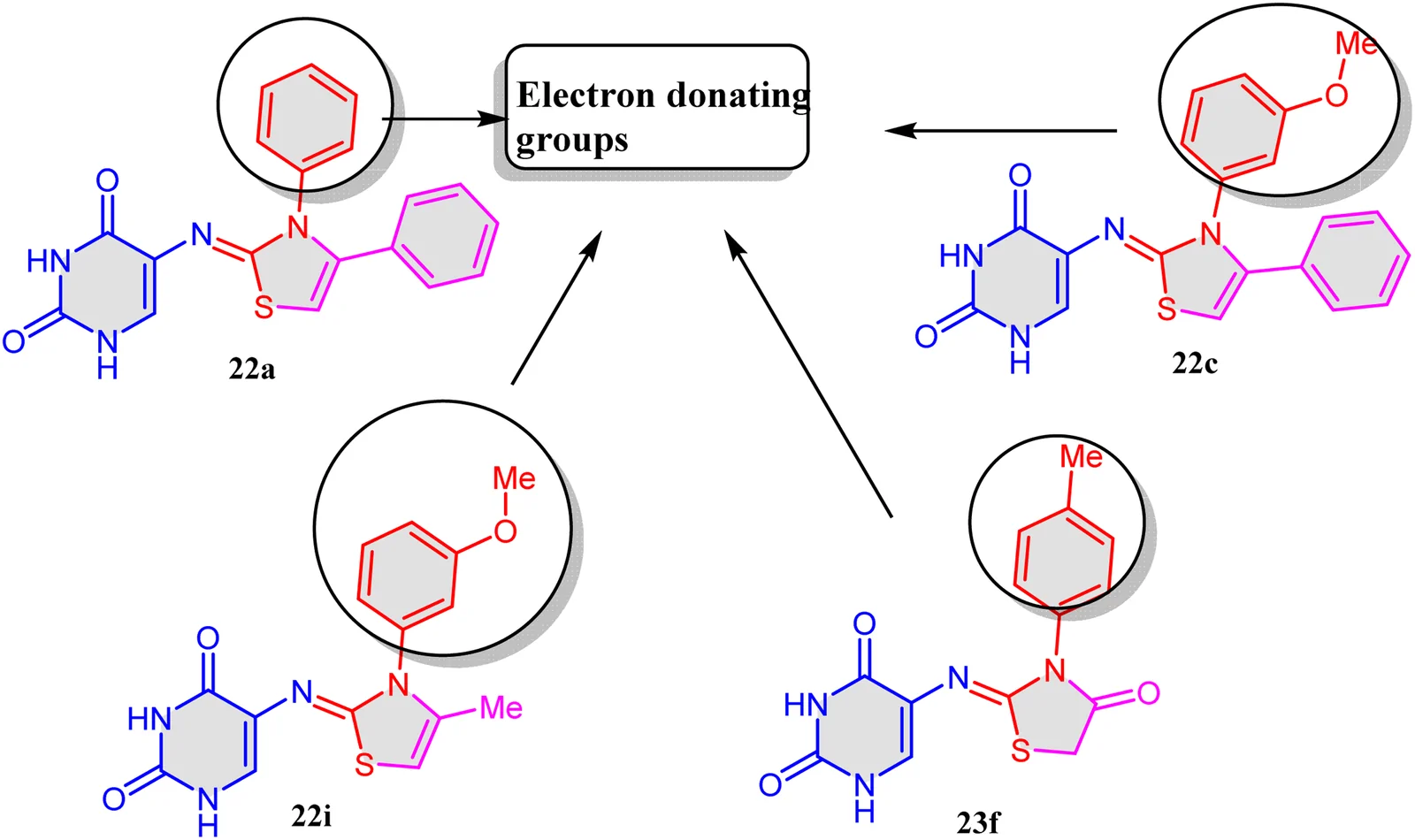
Structure of antiproliferative uracil–thiazole derivatives.

From the data provided in (Table 4), it was illustrated that the most potent derivatives were compounds 22a, 22c, 22f, 22i, and 23b (Fig. 4). Compound 22c (R = 3-MeO-Ph, R^1^ = Ph) was the most effective molecule among the tested products compared to doxorubicin (GI_50_ = 1.10 mM). Compound 22i (R = 3-MeO-Ph, R^1^ = CH_3_) has a GI_50_ value of 1.15 mM, which is equivalent to the potency of doxorubicin. With the exception of the methyl group at position four of the thiazolidine ring, compound 22i resembles the same structural backbone as compound 22c. This suggests that, like compound 22c, the phenyl group has a better effect on antiproliferative action than the methyl group. Compounds 22a (R = R^1^ = Ph) and 22b (R = 4-Me-Ph, R^1^ = Me) demonstrated significant antiproliferative activity, with GI_50_ values of 1.25 mM and 2.80 mM, respectively, being 1.4-fold and 3-fold less potent than 22c, respectively. These results demonstrated that the phenyl moiety's substitution pattern in the thiazolidine ring's third position is necessary for its antiproliferative properties. Compounds 22e (R = Me, R^1^ = Ph) and 22f (R = allyl, R^1^ = Ph) were less effective than 22a (R = R^1^ = Ph), with GI_50_ values of 3.05 mM and 1.90 mM, respectively. Compounds 22c (R = 3-MeO-Ph) and 22d (R = CH_2_-Ph) demonstrated great activity, with GI_50_ values of 1.70 mM and 1.45 mM, respectively. However, compounds 22e (R = CH_3_) and 22f (R = allyl) were the least potent derivatives, with GI_50_ values of 7.70 mM and 7.10 mM, respectively.

#### 2.1.3. Synthesis of diverse hybrid-pyrimidine compounds

**Scheme 6:**
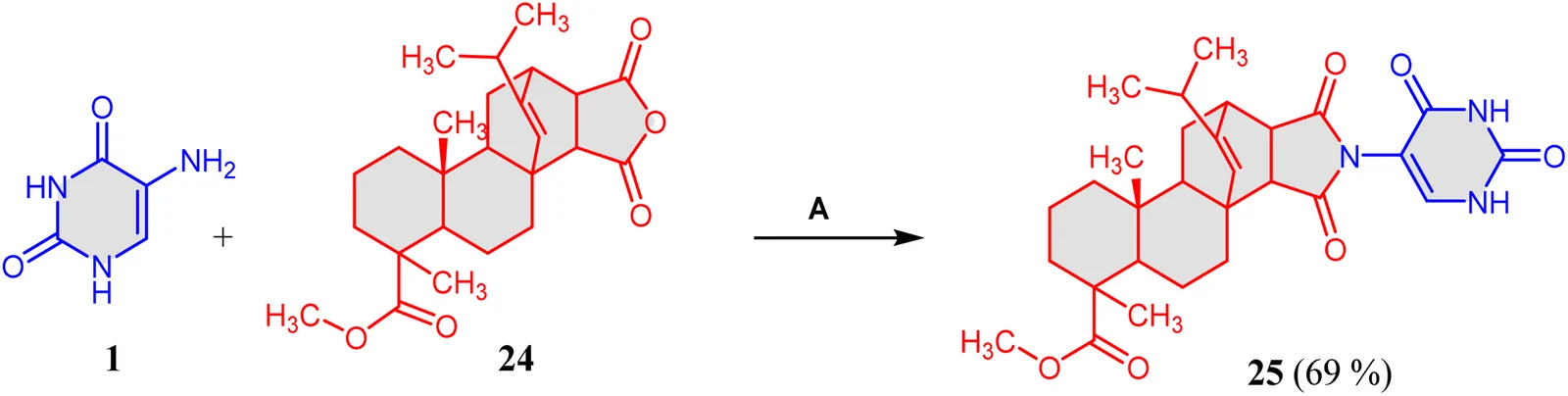
Ultrasound irradiation synthesis of compound 25. Reagents and conditions: A = DMSO, ultrasound, 120 °C, 30 min.

It has reported on the synthesis of maleopimarimide 25 in 69% yield *via* the condensation reaction between 5-aminouracil (1) and compound 24 in DMSO under ultrasonic (US) irradiation at 120 °C for 30 min (Scheme 6).^71^

**Scheme 7:**
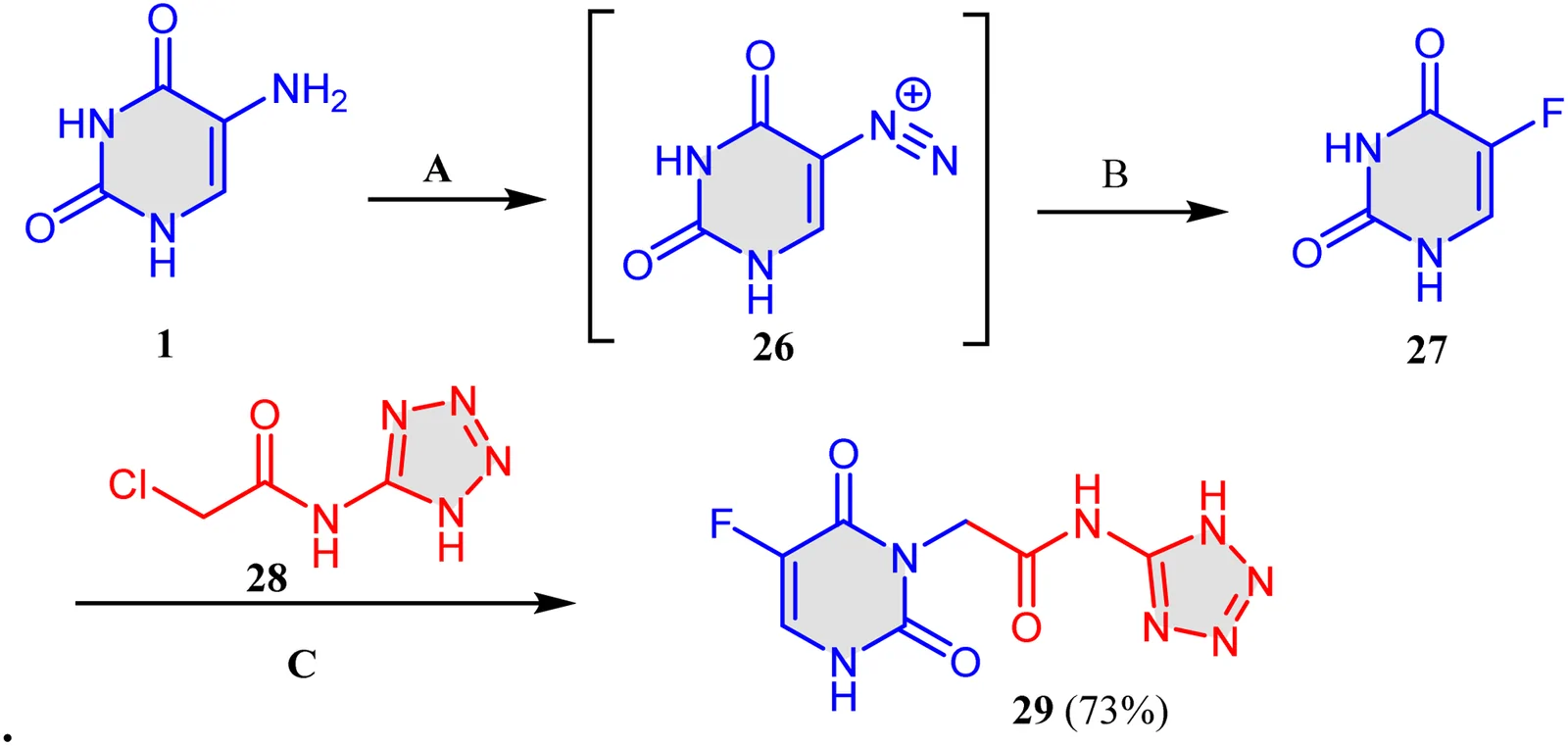
Synthesis of tetrazole-uracil hybrid 29. Reagents and conditions: A = NaNO_2_, AcOH. B; HF, pyridine. C = solvent free, 90 °C, 1 h.

Nowdehi *et al.*^71^ developed an efficient synthetic route for tetrazine-uracil hybrid 29 (Scheme 7). First, 5-aminouracil (1) was treated with sodium nitrite in acetic acid (AcOH) to produce diazonium salt 26, which reacted with hydrogen fluoride (HF) in pyridine to produce 5-flourouracil (27). Eventually, 2-(5-fluoro-2,6-dioxo-3,6-dihydropyrimidin-1(2*H*)-yl)-*N*-(1*H*-tetrazol-5-yl)acetamide (29) was obtained from the reaction of 27 with 2-chloro-*N*-(1*H*-tetrazol-5-yl)acetamide (28) (Scheme 7). Compound 29 displayed enhanced inhibition of AGS cancer cell (a human gastric adenocarcinoma cell line derived from stomach tissues) proliferation compared to 5-fluorouracil, with an IC_50_ value of 15.67 μg mL^−1^*vs.* 36.42 μg mL^−1^ and 45.90 μg mL^−1^ for synthesized and reported 5-FU values, respectively.

### 2.2. Heterocycles from 6-aminouracil

6-Aminouracil is a privileged scaffold and one of the main starting materials for synthesizing complex compounds because it plays dual roles as an electrophile and a nucleophile.^5^ The most notable nucleophilic activity is found at position-3, and it is easy to prepare polycyclic compounds with biological targets like pyrido-, pyrrolo-, and pyrimido-pyrimidines as well as fused annulated compounds with other materials.^72,73^ A wide range of pharmacological and biological effects have been demonstrated by these compounds, such as antimicrobial^74–76^ and anticancer,^77–79^ and they are employed as anticoagulant,^80,81^ antifungal,^82,83^ antiviral,^84,85^ antitumor,^86,87^ antioxidant,^72,88^ and anti-inflammatory agents,^89^ as well as HIV protease inhibitors^90–93^ and tyrosine kinase inhibitors.^94^

**Fig. 5:**
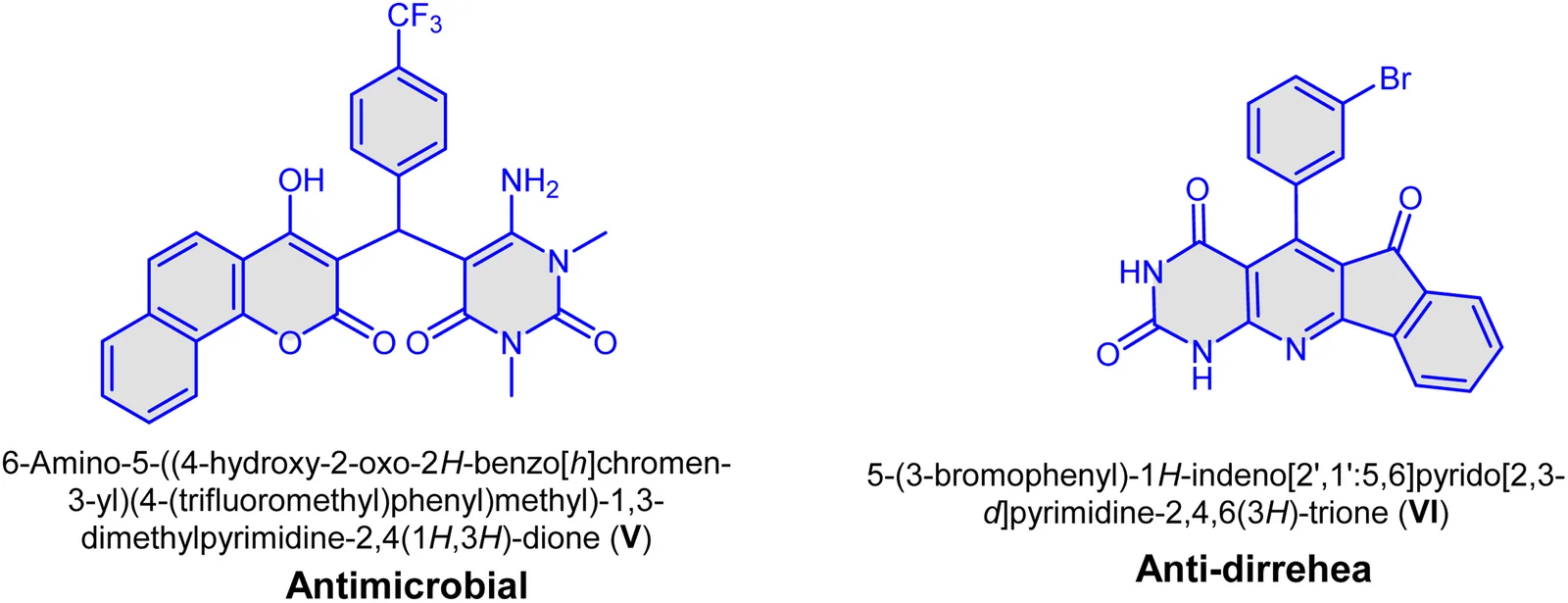
Structures of some biologically active and fused 6-aminouracil compounds V–VI.

6-Aminouracil (2) is an essential component of numerous synthetic^5,95–98^ and natural compounds^94,95^ with medicinal properties. Also, 6-aminouracil(s) has/have broad biological activities such as antiviral,^99,100^ antidiarrheal,^101^ anti-microbial,^102^ antiallergic,^103^ anticancer,^78^ adenosine receptor antagonist antifungal,^104^ insecticidal,^105^ and acaricidal activities.^106^ They are found in pyrido, pyrrolo, pyrimido, fused spiro oxindole, and arylmethane structures.^107^ For example, 6-amino-5-((4-hydroxy-2-oxo-2*H*-benzo[*h*]chromen-3-yl)(4-(trifluoromethyl)phenyl)methyl)-1,3-dimethylpyrimidine-2,4-dione (V, Fig. 5) and 5-(3-bromophenyl)-1*H*-indeno[2′,1′:5,6]pyrido[2,3-*d*]pyrimidine-2,4,6-trione (VI, Fig. 5) were known as antimicrobial and antidiarrhea, respectively.^101,102^ Moreover, Fuentes-Rios *et al.*^108^ utilized 1,3-dimethyl-4,5-diaminouracil to form an imine group, which effectively protected the carbonyl group in sugars. It is interesting to mention that compound V could be generally prepared *via* the one-pot reaction of 4-hydroxy-2*H*-benzo[*h*]chromen-2-one with 4-trifloromethyl benzaldehyde and 6-amino-1,3-dimethylpyrimidine-2,4(1*H*,3H)-dione in refluxing AcOH.^102^ Compound V exhibited significant activity against *Staphylococcus aureus* MTCC 96 with IC_50_ = 9.37 μg mL^−1^, and very good activity against *Staphylococcus aureus* MLS 16 MTCC 2940 with IC_50_ = 2.34 μg mL^−1^, comparable to the reference Ciprofloxacin (IC_50_ = 0.58 μg mL^−1^).^102^

Similarly, compound VI can be generally prepared by the one-pot condensation of 3-bromobenzaldehyde, 1,3-indandione and 6-aminouracil (2) in water in the presence of graphene oxide.^109^ It was reported that compound VI suppresses cyclic nucleotide synthesis in the presence of STa (with an IC_50_ value of 3.4 ± 1.6 μM at 100 nM STa) and is active *in vivo* in an intestinal loop animal model of acute diarrhea.^101^

#### 2.2.1. Synthesis of bis-(6-aminouracils)

**Scheme 8:**
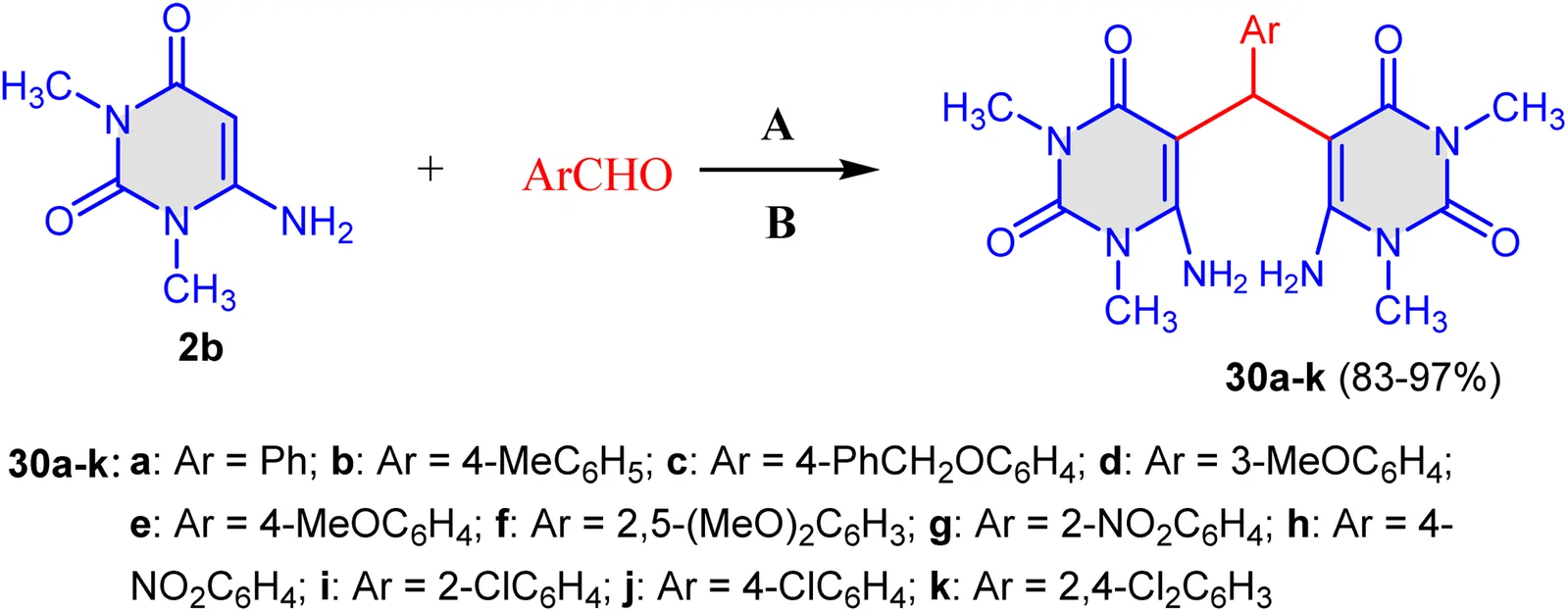
Formation of bis uracils 30a–k. Reagents and conditions: A; Nano-[FSRN][H_2_PO_4_]. B; solvent-free, 120 °C.

Using the green approach, Lotfifar *et al.*^53^ were able to successfully synthesize a series of bis(6-amino-1,3-dimethyluracil-5-yl)methane derivatives 30a–k. The catalyst nano-[Fe_3_O_4_@SiO_2_-R-NHMe_2_][H_2_PO_4_] is magnetically recyclable. It was used during the reaction of 6-amino-1,3-dimethyluracil (2b) with different aldehydes. The formed products were obtained in excellent yield of 80–97%. Meanwhile, another nano catalyst was announced as *N*,*N*,*N*,*N′*-tetramethyl-ethylenediaminium bisulfate ([TMEDA][HSO_4_]_2_), which was utilized to produce the final products 30a–k (Scheme 8).^110^

**Scheme 9:**
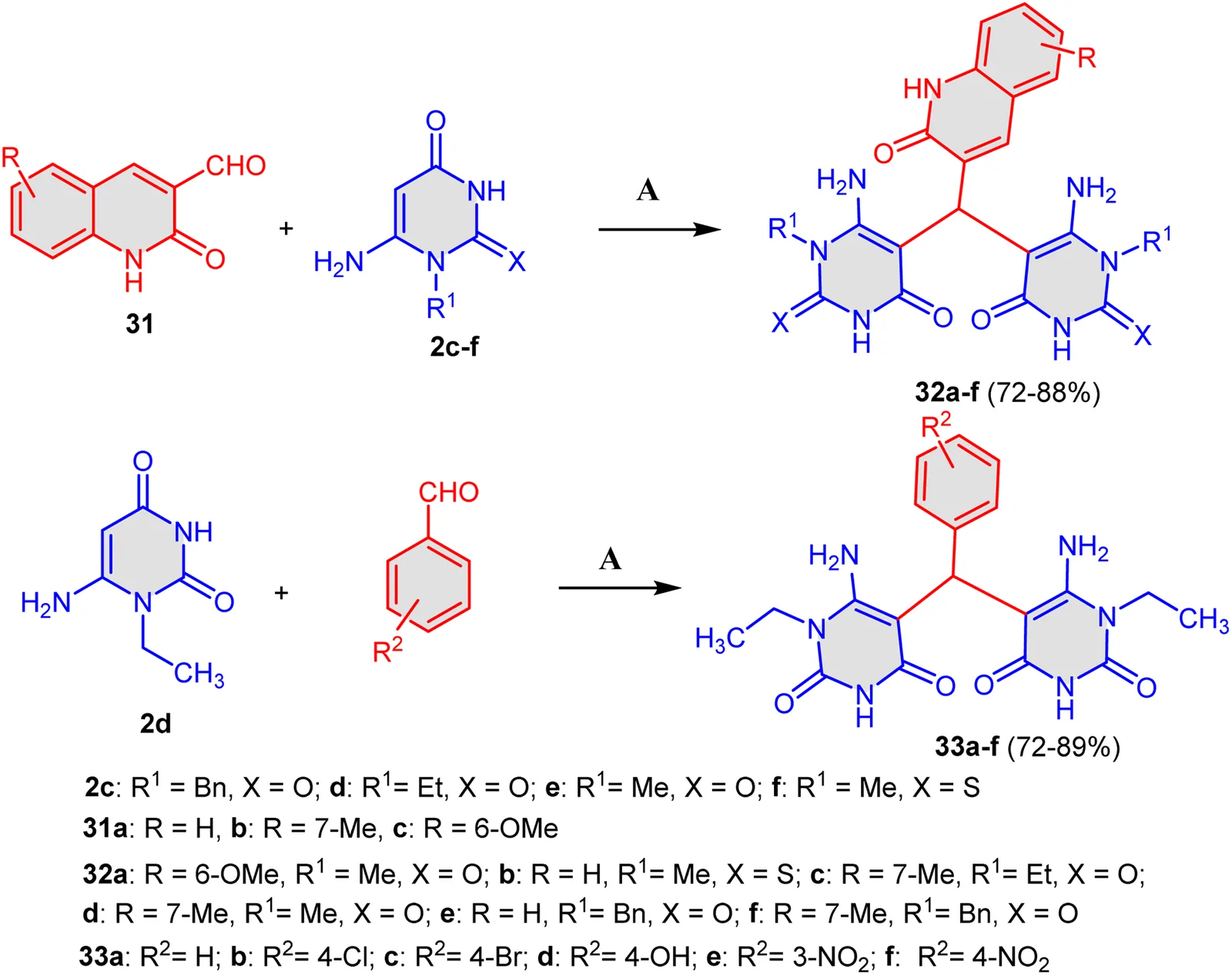
Synthesis of quinoline-bis uracil compounds 32a–f and 33a–f. Reagents and conditions: A; AcOH, reflux, 3–4 h.

In 2023, El-Kalyoubi *et al.*^111^ reacted quinolone-3-carbaldehydes 31 with 6-aminouracil derivatives 2c–f in refluxing acetic acid (AcOH) to obtain bis-uracils 32a–f. Under the same conditions, 6-amino-1-ethyl-uracil (2d) reacted with aromatic aldehydes to produce bis-aminouracils 33a–f (Scheme 9). The anti-proliferative activities of these compounds were screened against three distinct cancer cell lines (HepG-2 hepatic carcinoma, MCF-7 breast adenocarcinoma, and A549 lung cancer) and compared with the standard reference doxorubicin (Tables 5 and 6).^111^

**Table 5:** Anti-proliferative activity of bis-uracil/quinoline derivatives 32a–f and 33a–f against human cancer cell lines (IC_50_ [μM])

Compd	R	R^1^	R^2^	X	IC_50_ values		
					A549	MCF7	HepG-2
32a	6-OMe	Me	—	O	63.01 ± 0.9	129.8 ± 1.02	58.31 ± 1.26
32b	H	Me	—	S	49.13 ± 0.16	60.31 ± 0.62	59.55 ± 1.01
32c	7-Me	Et	—	O	54 ± 0.41	64.19 ± 0.34	92.68 ± 0.44
32d	7-Me	Me	—	O	66.84 ± 0.66	107.39 ± 2.17	185.99 ± 0.58
32e	H	Bz	—	O	52.92 ± 0.25	103.27 ± 1.73	97.08 ± 0.85
32f	7-Me	Bz	—	O	2.49 ± 0.15	5.00 ± 0.16	6.24 ± 0.04
33a	—	—	H	—	108.52 ± 1.77	77.97 ± 1.52	253.77 ± 4.37
33b	—	—	4-Cl	—	102.1 ± 0.23	199.28 ± 1.97	243.79 ± 1.04
33c	—	—	4-Br	—	53.52 ± 0.7	177.56 ± 1.26	107.66 ± 0.31
33d	—	—	4-OH	—	66.2 ± 1.55	230.17 ± 3.75	202.56 ± 7.34
33e	—	—	3-NO_2_	—	127.41 ± 3.27	230.25 ± 1.83	203.7 ± 0.67
33f	—	—	4-NO_2_	—	56.41 ± 0.87	219.33 ± 4.77	185.6 ± 8.04
Doxorubicin	—	—	—	—	43.11 ± 1.22	9.93 ± 0.57	35.5 ± 0.46

**Table 6:** Cytotoxic effects of the hybrids 32b, 32c and 32f on both A549 and normal cell lines

Compd	Cytotoxicity IC_50_ μM		
	A549	Vero	SI
32b	49.13 ± 0.16	204.15 ± 0.49	4.15
32c	54 ± 0.41	251.79 ± 4.41	4.66
32f	2.49 ± 0.15	28.49 ± 3.12	11.44
Doxorubicin	43.11 ± 1.22	80.4 ± 0.73	1.86

##### 2.2.1.1 Structure activity relationship

**Fig. 6:**
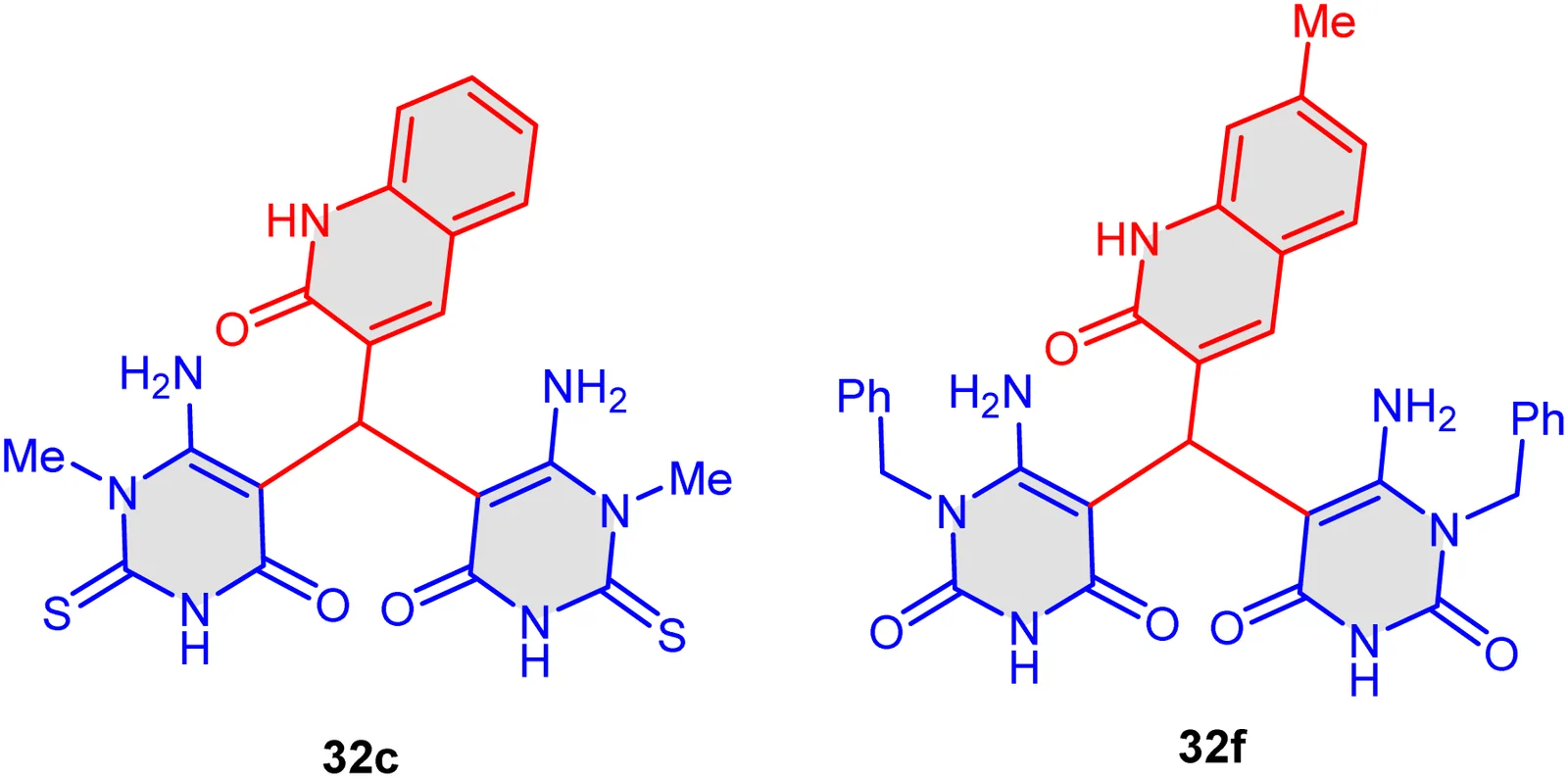
Structures of antiproliferative compounds 32c and 32f.

Based on the results presented in Tables 5–7, compounds 32a (R = 6-Me, R^1^ = Me) and 32d (R = 6-Me, R^1^ = Me) revealed a moderate activity against the standard drug (doxorubicin). However, compound 32e, which contains non-substituted quinoline and R^1^ = benzyl, demonstrated non-selective efficacy against A549, MCF-7, and HepG-2 cell lines in the micromolar range with IC_50_ values of 52.92, 103.27 and 97.08 μM, which compared to 43.11, 9.93 and 35.50 μM for doxorubicin, respectively. The activity increased when additional electron-donating groups, where (R = Et) 32c (Fig. 6), were substituted for the methyl group on the bisuracil unit (IC_50_ = 54.00, 64.19, and 92.68, respectively). It was also shown that biological effectiveness was significantly influenced by the type of heteroatom present on the uracil ring. When the oxygen atom was replaced with a sulfur atom, the antiproliferative activity was extremely improved, as observed in 32b (IC_50_ values of 49.13, 60.31, and 59.55 μM in A549, MCF-7, and HepG-2 cell lines, respectively). The activity increased as a result of substituents at the uracil ring (R^1^ = benzyl) and the electron-donating substituent's impact on the quinoline moiety (R = Me), as shown in compound 32f (Fig. 6). An acceptable activity that appeared unlikely to be better for the activity was obtained by substituting the quinoline moiety into a substituted monoaryl system. Summarily, adding a rigid bulky group to the uracil moiety and/or a lipophilic electron donating group to the quinoline ring resulted in a significant improvement in anti-proliferative inhibitory activity.

**Table 7:** Inhibitory efficacy of compounds (32b, 32c and 32f) against topoisomerases I and II

Compd	Topoisomerase I (IC_50_) μM	Topoisomerase II (IC_50_) μM
32b	12.17 ± 0.58	20.28 ± 0.94
32c	43.57 ± 2.09	32.42 ± 1.50
32f	2.83 ± 0.14	7.34 ± 0.34
Camptothecin	1.07 ± 0.05	—
Doxorubicin	—	4.22 ± 0.19

#### 2.2.2. Synthesis of pyrrolo-pyrimidine derivatives

**Scheme 10:**
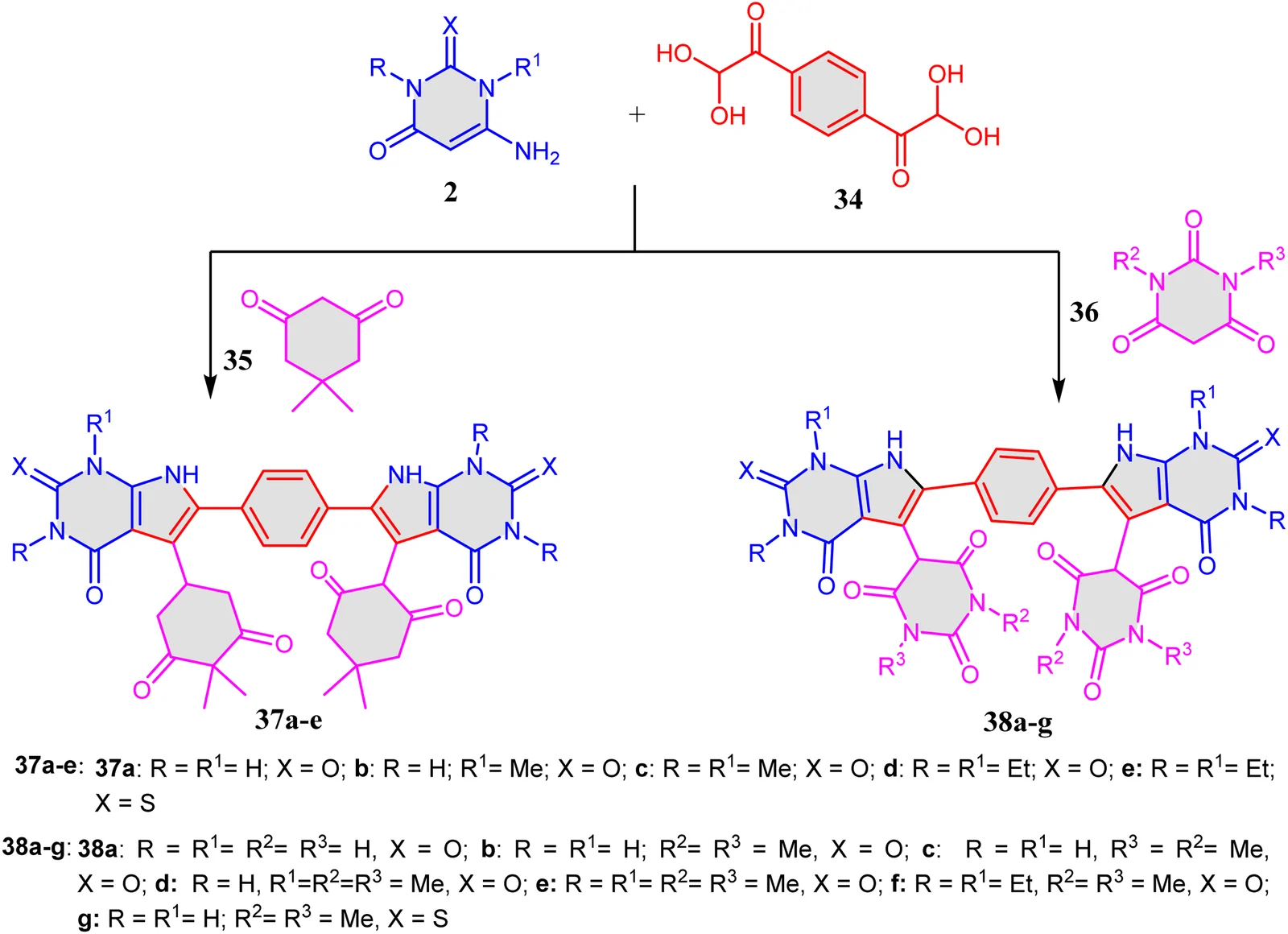
Synthesis of bispyrrolo[2,3-*d*]pyrimidines 37a–e and 38a–g. Reagents and conditions: A; TRAB, EtOH, reflux.

Bis-pyrrolo[2,3-*d*]pyrimidine derivatives 37a–e or 38a–g were obtained in 90–96% yields *via* a one-pot multicomponent reaction between 6-aminouracils (2), 1,4-phenylene-bis-glyoxal (34), and dimedone (35) or derivatives of barbituric acid (36), and the reaction was performed in 5% tetrapropylammonium bromide (TRAB) and EtOH at reflux temperature (Scheme 10).^112^ In a trial of study, the reaction of 1,4-phenylene-bis-glyoxal (34), 6-aminouracil (2), and dimedone (35) was chosen as a model reaction to form products 38a–e. However, the reaction of 34, 2, and barbituric acid (36) was chosen as a model reaction for products 38a–g (Table 8). First, these model reactions were carried out in the absence of a catalyst, but no products were observed even after 4 h of stirring at 50 °C (Table 8, entry 1). It was found that the yields improved when the temperature was increased to reflux. The best result was obtained in terms of yield (95% and 92%) and reaction time (1.5 h) when the reaction was performed using 5 mol% of TPAB (Table 8, entry 6). The use of EtOH proved to be the best in terms of yield and reaction time (Table 8, entry 6).

**Table 8:** Effects of several solvents, temperatures, and mol% of TPAB on the synthesis of 37a–e and 38a–e^a^

Entry	Catalyst	Temperature (°C)	Solvent	Time (h)	37a yield (%)	38a yield (%)
1	No catalyst	50	EtOH	4	No reaction	No reaction
2	No catalyst	Reflux	EtOH	2	48	41
3	TPAB (5 mol%)	RT	EtOH	1.5	51	47
4	TPAB (5 mol%)	50	EtOH	1.5	48	47
5	TPAB (5 mol%)	60	EtOH	1.5	55	50
6	TPAB (5 mol%)	Reflux	EtOH	1.5	95	92
7	TPAB (10 mol%)	Reflux	EtOH	1.5	94	90
8	TPAB (5 mol%)	Reflux	THF	1.5	40	40
9	TPAB (5 mol%)	Reflux	MeOH	1.5	55	50
10	TPAB (5 mol%)	Reflux	CH_2_Cl_2_	1.5	20	Trace
11	TPAB (5 mol%)	Reflux	CH_3_CN	1.5	28	Trace
12	TPAB (5 mol%)	Reflux	DMF	1.5	45	43
13	TPAB (5 mol%)	Reflux	H_2_O	1.5	61	58

a 1,4-Phenylene-bis-glyoxal (1 mmol), 6-aminouracil (2 mmol), and barbituric acid (2 mmol)/EtOH (5 mL). 1,4-phenylene-bis-glyoxal (1 mmol), 6-aminouracil (2 mmol), and dimedone (2 mmol)/EtOH (5 mL). RT, room temperature; TPAB, tetrapropylammonium bromide.

**Scheme 11:**
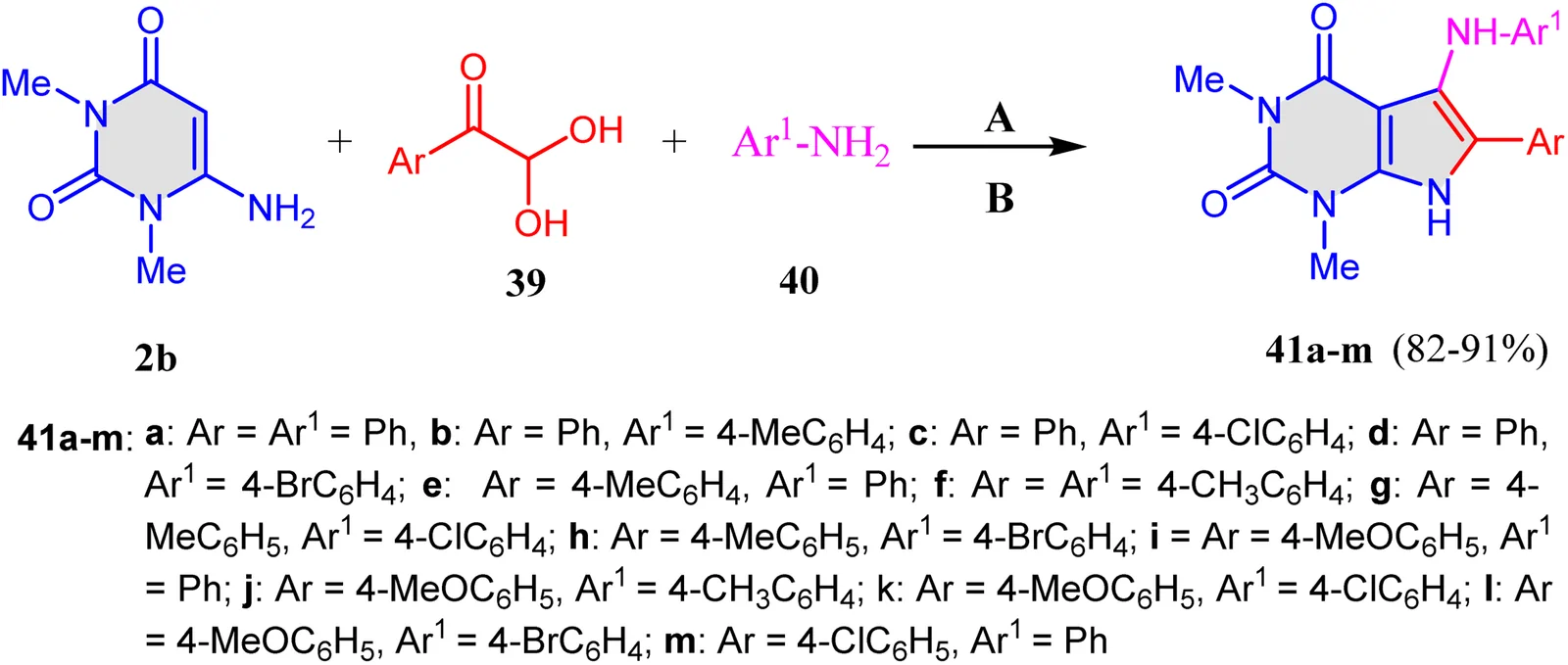
Three-component reaction for the formation of pyrrolo[2,3-*d*]pyrimidines 41a–m. Reagents and conditions: A; AcOH, MW 110 °C, 5 min.

In the same previous manner, a microwave-assisted one-pot reaction involving *N*,*N*-dimethyl-6-aminouracil (2b), aryl glyoxal monohydrates 39, and aryl amines 40 was used to synthesize 5-arylamino-pyrrolo[2,3-*d*]pyrimidine derivatives 41a–m (Scheme 11).^113^ It is worth mentioning that the reaction proceeded efficiently in AcOH at 110 °C to give the corresponding product 41a in 84% yield (Table 9, entry 8).

**Table 9:** Optimized conditions for a model example of 2b, 39, and aniline (40a) under microwave irradiation

Entry	Solvent	Catalyst	Temp (°C)	Time (min)	Yield (%)
1	EtOH	—	75	5	25
2	EtOH	PTSA	75	5	40
3	EtOH	InCl_3_	75	5	35
4	EtOH	Sc(OTf)_3_	75	5	30
5	CH_3_CN	PTSA	80	5	Trace
6	Toluene	PTSA	100	5	Trace
7	DMF	PTSA	100	5	Trace
8	AcOH	—	100	5	84

**Scheme 12:**
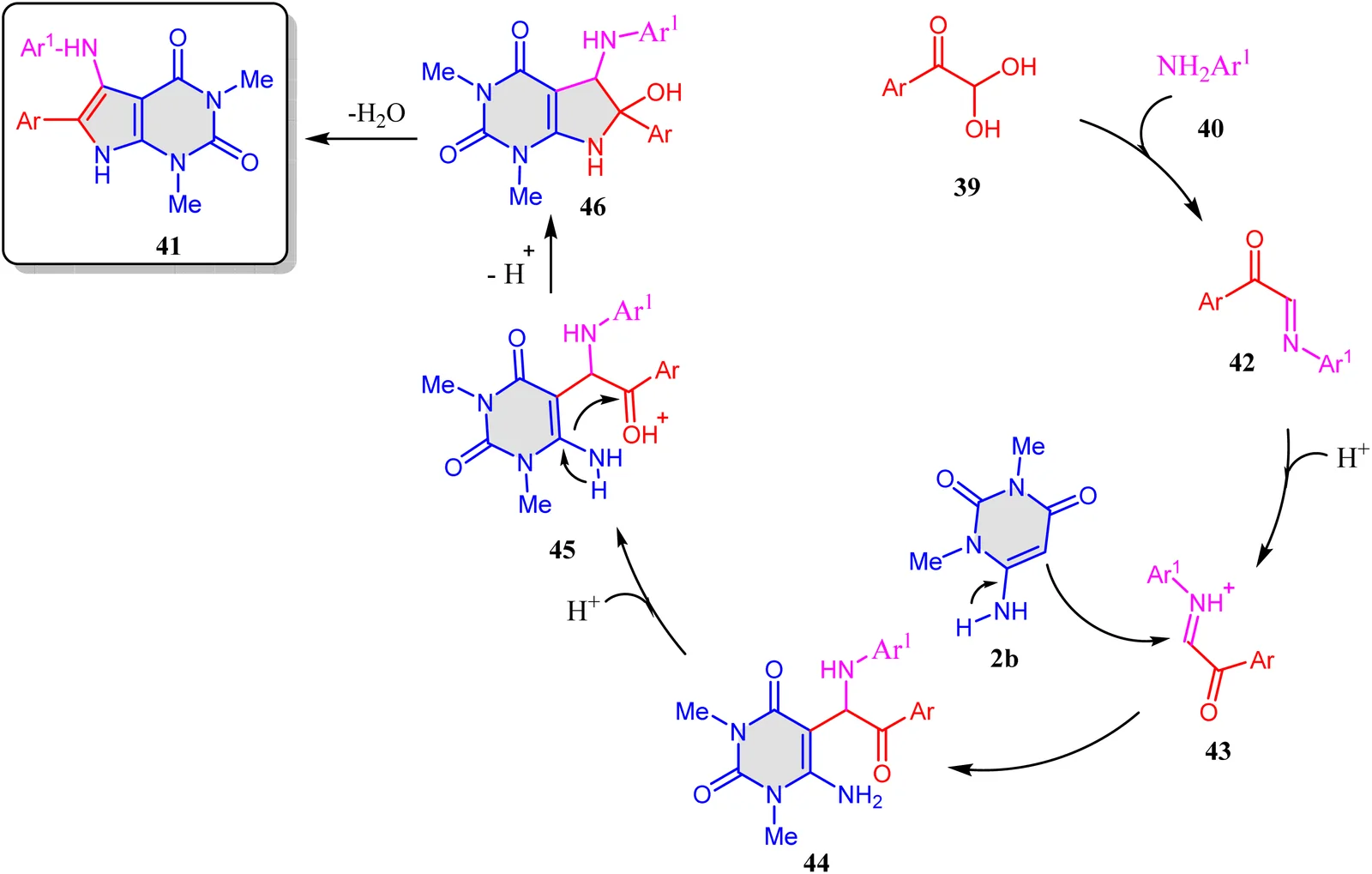
Proposed mechanism for the formation of 41a–m.

Scheme 12 illustrates the suggested mechanism in which AcOH would act as a solvent and promoter of Brønsted acid during the reaction as well. Initially, the condensation reaction occurs between aryl glyoxal (39) and aryl amines 40 to give compound 42. The latter in the presence of acidic media was then converted into intermediate 43, which would be nucleophilically attacked by C-5 of 2b to give intermediate 44. Then, the transformation of 44 into intermediate 45 was occurred, which was subsequently followed by an intermolecular cyclization to form intermediate 46. Finally, the loss of a hydrogen proton from 46, followed by dehydration, produces the targeted products 41a–m.^113^

#### 2.2.3. Synthesis of thiazolo-pyrimidine derivatives

**Scheme 13:**
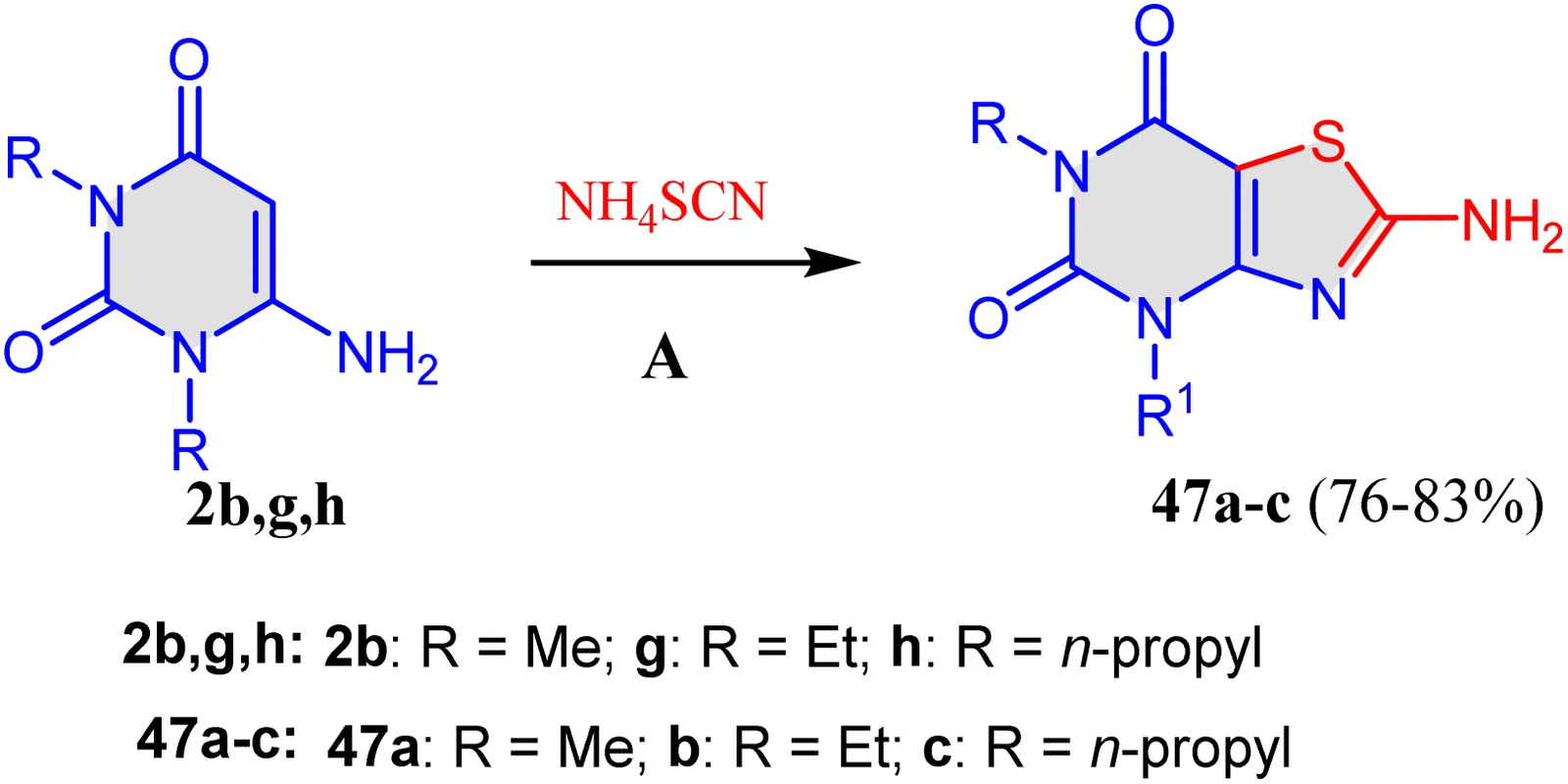
Synthesis of uracil-fused 2-aminobenzothiazoles 47a–c. Reagents and conditions: A; (i) H_2_O_2_, rt; (ii) NaOH, rt, 1 h.

Interestingly, Ali *et al.*^47^ successfully synthesized amino-thiazolo[4,5-*d*]pyrimidine-5,7-(4*H*,6*H*)-dione derivatives 47a–c in good yields (76–83%) by treating 6-aminouracils 2b, g, and h with ammonium thiocyanate (NH_4_SCN) and catalyzed by H_2_O_2_ at room temperature (Scheme 13).

#### 2.2.4. Synthesis of imidazo-pyrimidinedione (xanthine) derivatives

**Scheme 14:**
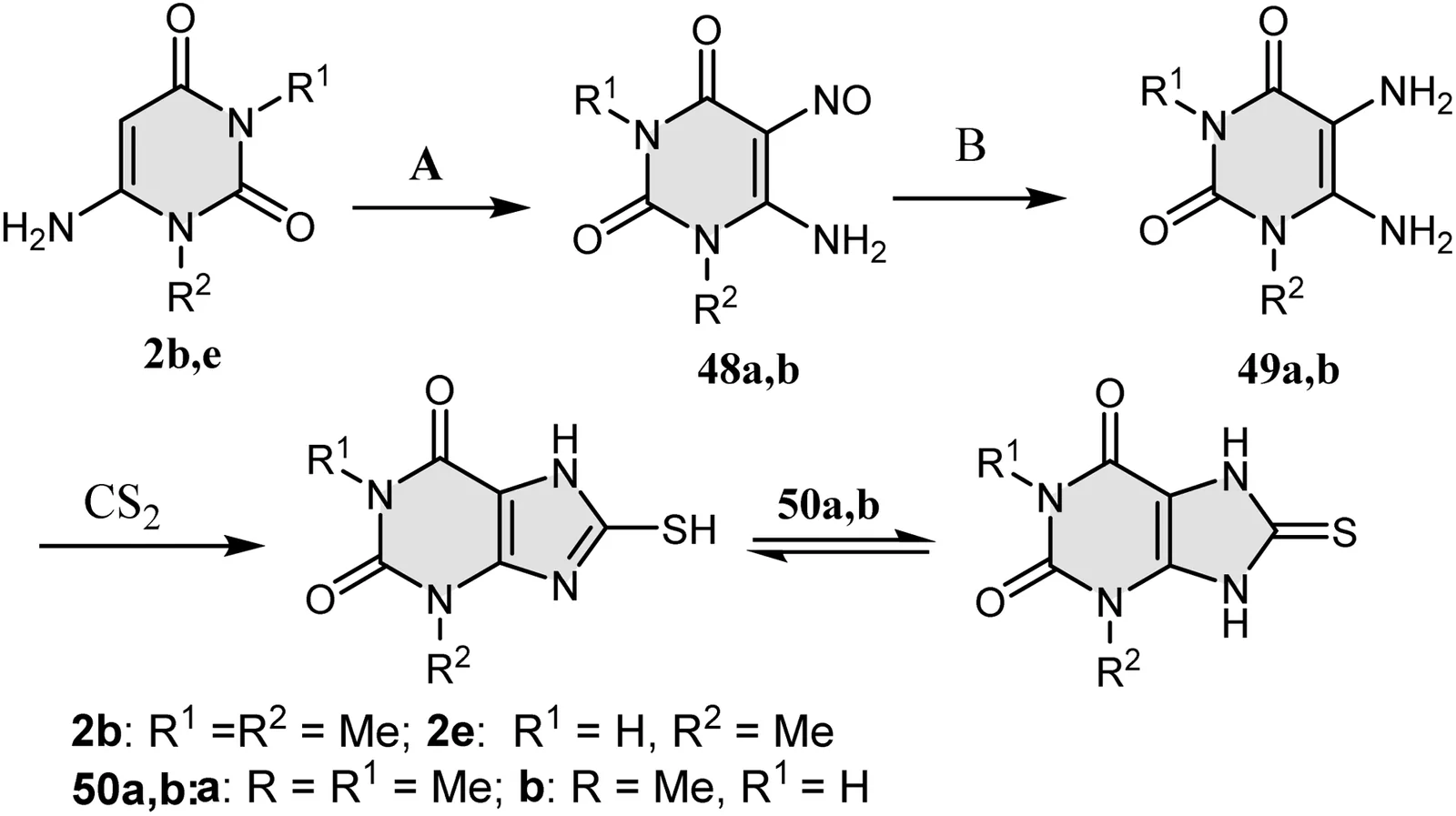
Synthesis of xanthine derivatives 50a,b. Reagents and conditions: A; NaNO_2_/AcOH. B; NH_3_/Na_2_S_2_O_4_. C; CS_2_/(DMF/EtOH), reflux.

The nitrosation of 6-aminouracils 2b,e directly produced 5-nitroso-6-aminouracils 48a and b by employing sodium nitrite in 50% aqueous AcOH. In an aqueous ammonia solution and sodium dithionite (Na_2_S_2_O_4_), reduction of 48a,b produced 5,6-diamino-1,3-dimethyluracils 49a,b. As shown in Scheme 14,^31^ xanthine derivatives 50a,b were produced by directly cyclizing 5,6-diamino-uracils 49a,b using carbon disulfide (CS_2_) in DMF under reflux for 5 h.^31^

**Scheme 15:**
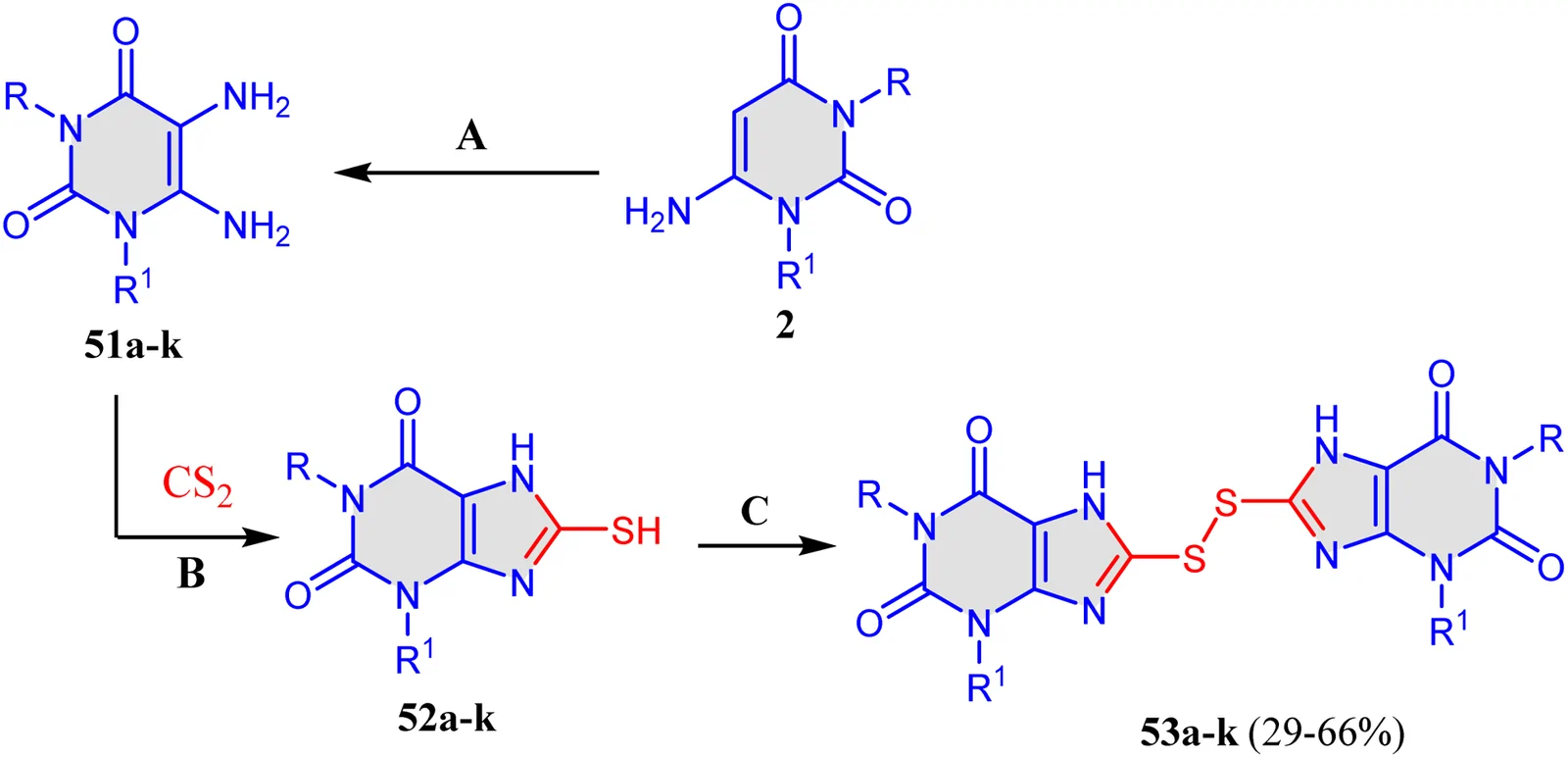
Synthesis of 8,8′-disulfanediylbis(dihydro-1*H*-purine-2,6-diones) 53a–k. Reagents and conditions: A; NaNO_2_/AcOH, 70 °C, Na_2_S_2_O_4_, NH_4_OH, 70 °C. B; NaHCO_3_, EtOH:H_2_O, 65 °C, C; (i) I_2_, KOH, EtOH, rt. (ii) reflux, overnight.

In 2020, Han *et al.*^114^ developed a synthesis of a series of 8,8′-disulfanediyl-bis(3-ethyl-1-substituted-3,7-dihydro-1*H*-purine-2,6-diones) 53a–k (Scheme 15). Moreover, 5,6-diamino-uracils 51a–k were prepared through the reaction of 6-aminouracils 2 with sodium nitrite (NaNO_2_) in the presence of AcOH. Following that, 5,6-diaminouracils 51a–k were treated with CS_2_ in EtOH/H_2_O in the presence of sodium bicarbonate (NaHCO_3_) at 65 °C to give 8-mercapto-3,7-dihydro-1*H*-purine-2,6-diones 52a–k. The resulting mercapto-purines were then refluxed in EtOH/KOH, and the reaction proceeded to give the final products 53a–k (Scheme 15). The potential uses of sirtuin inhibitors include the treatment of several types of cancer and neurological diseases. The tested compounds 53a–k showed that they are potent SIRT1/2/3/5 pan-inhibitors. Besides, compound 53f (R = phenylethyl) exhibited the highest activity among all the examined derivatives. The results showed that 53f was nontoxic at concentrations higher than 20 μM and stable in the assays, as shown in Table 10.^114^

**Table 10:** Inhibitory activities of compound 53a–k and their derivatives for SIRT1/2/3/5/6

Compd	R	R^1^	IC_50_ (μM)				
			SIRT3	SIRT1	SIRT2	SIRT5	SIRT6
53a	–CH_2_CH_3_	–CH_2_CH_3_	0.79 ± 0.06	0.10 ± 0.01	1.17 ± 0.04	0.42 ± 0.01	116.0 ± 6.2
53b	–CH_2_CH_2_CH_3_	–CH_2_CH_2_CH_3_	0.54 ± 0.05	0.12 ± 0.01	1.19 ± 0.06	0.39 ± 0.03	128.7 ± 16.1
53c	–CH_2_CH(CH_3_)_2_	–CH_2_CH_3_	1.77 ± 0.05	0.43 ± 0.03	4.86 ± 0.08	1.51 ± 0.10	296.6 ± 11.3
53d	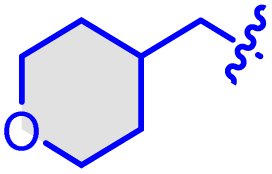	–CH_2_CH_3_	0.72 ± 0.06	0.17 ± 0.01	2.25 ± 0.12	0.50 ± 0.01	155.8 ± 6.5
53e	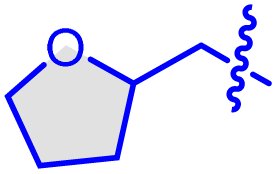	–CH_2_CH_3_	0.69 ± 0.12	0.15 ± 0.02	1.62 ± 0.05	0.54 ± 0.02	126.5 ± 10.2
53f	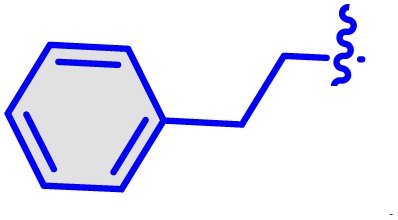	–CH_2_CH_3_	0.37 ± 0.05	0.17 ± 0.01	1.35 ± 0.05	0.45 ± 0.01	103.3 ± 6.3
53g	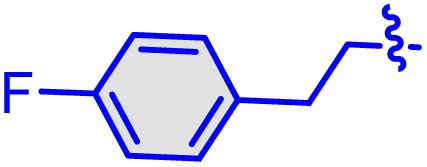	–CH_2_CH_3_	0.46 ± 0.04	0.19 ± 0.01	2.03 ± 0.04	0.84 ± 0.08	110.9 ± 2.6
53h	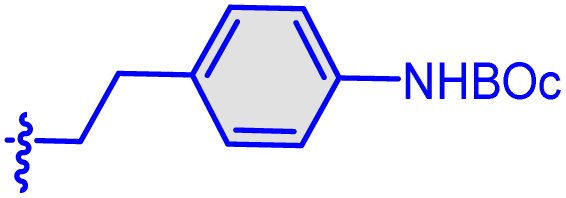	–CH_2_CH_3_	1.15 ± 0.08	0.41 ± 0.04	2.19 ± 0.08	1.00 ± 0.03	128.3 ± 4.1
53i	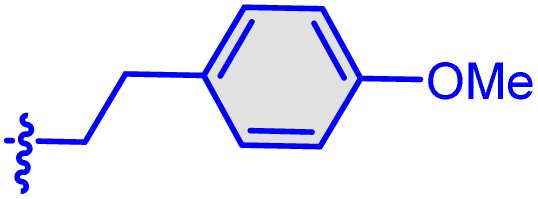	–CH_2_CH_3_	0.61 ± 0.03	0.21 ± 0.02	2.16 ± 0.08	0.61 ± 0.02	102.3 ± 9.1
53j	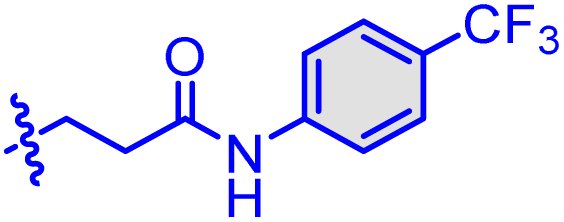	–CH_2_CH_3_	8.52 ± 0.22	1.87 ± 0.04	9.44 ± 0.19	26.12 ± 3.15	452.0 ± 9.8
53k	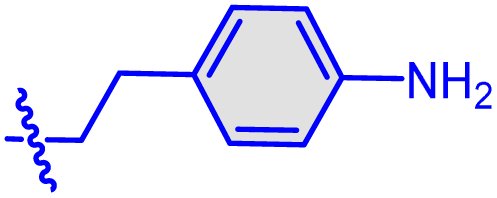	–CH_2_CH_3_	0.97 ± 0.06	0.49 ± 0.01	4.61 ± 0.13	0.92 ± 0.04	209.6 ± 5.9
ELT-31	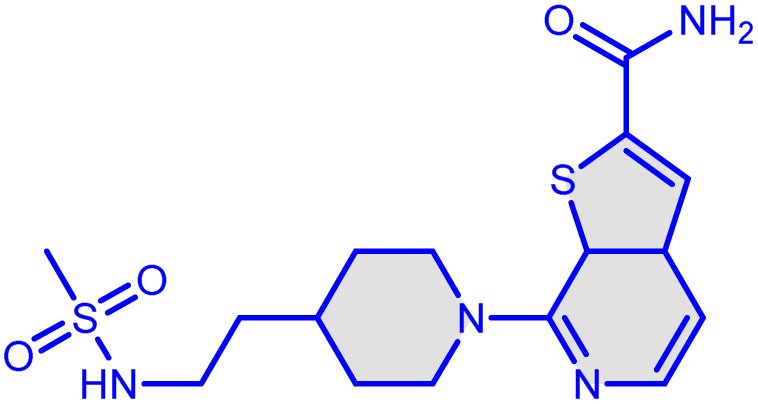		0.35 ± 0.02	0.27 ± 0.02	0.12 ± 0.01	>500	462.0 ± 14.9

##### 2.2.4.1 Structure activity relationship

**Fig. 7:**
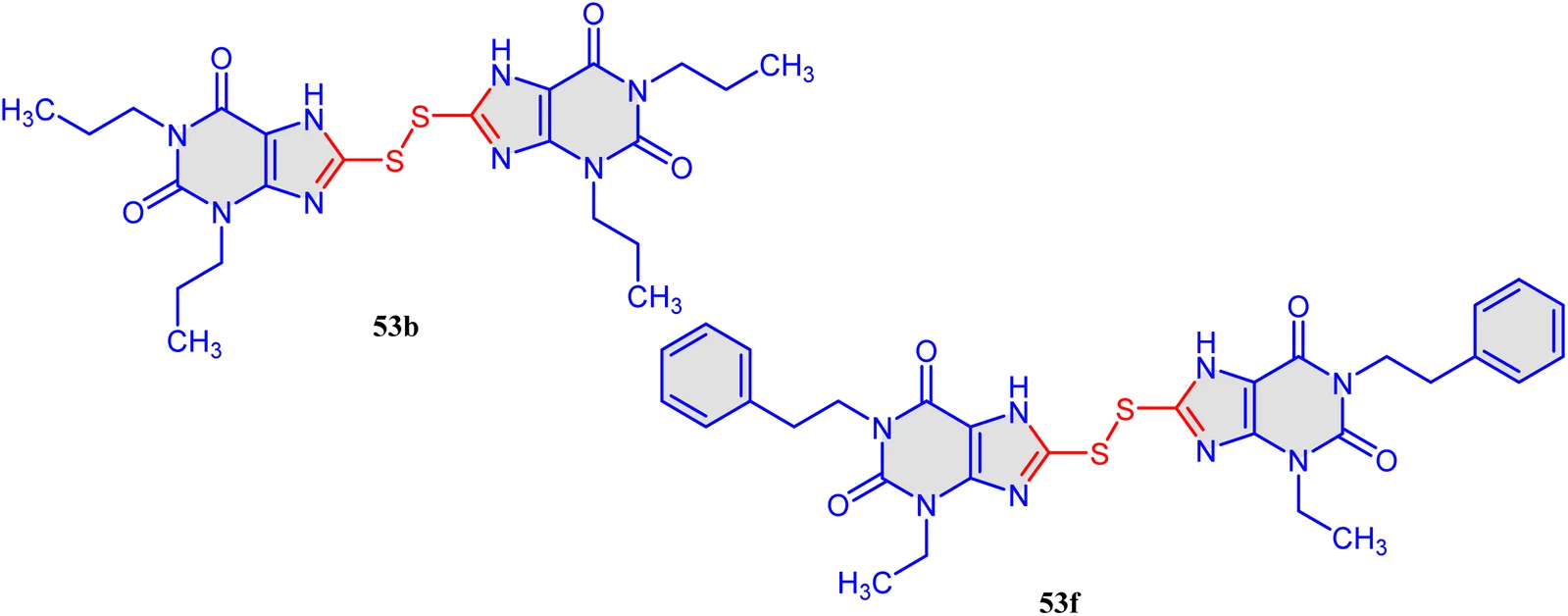
Structures of sirtuin inhibitor compounds 53b and 53f.

The data in Table 10 showed that compound 53b (R = propyl) was more potent than compound 53a (R = Et), demonstrating that the existence of long-chain alkyls at the R site improves the activity. Comparing the activities of 53a, 53b, and 53c (R = *tert*-Bu), it was noted that the results in groups with branched chains were not satisfactory, indicating that there is a restricted space surrounding the R location in SIRT3. Correspondingly, derivatives 53d (R = tetrahydropyran) and 53e (R = tetrahydrofuran) cause a decrease in inhibitory activity compared to 53b. Moreover, the presence of cycloalkanes at R (53d and 53e) increased the hydrophobicity, leading to an improvement in activity compared to 53c. Attempting to increase the activity of compound 53f (R = phenethyl) (Fig. 7), the inhibitory action of SIRT3 was significantly increased. Besides, compounds 63g–i and 53k, which had a fluoro, Boc-protected amino, OMe, or NH_2_ at the *p*-position of the phenethyl, respectively, showed a reduction in the activity. Furthermore, substituting the phenethyl with *N*-(3-(trifluoromethyl)phenyl)propionamide (compound 53j) significantly decreased inhibitory action (23-fold) compared to 53f (Fig. 7), suggesting that a larger group at the R position may cause steric hindrance with SIRT3.^114^

**Scheme 16:**
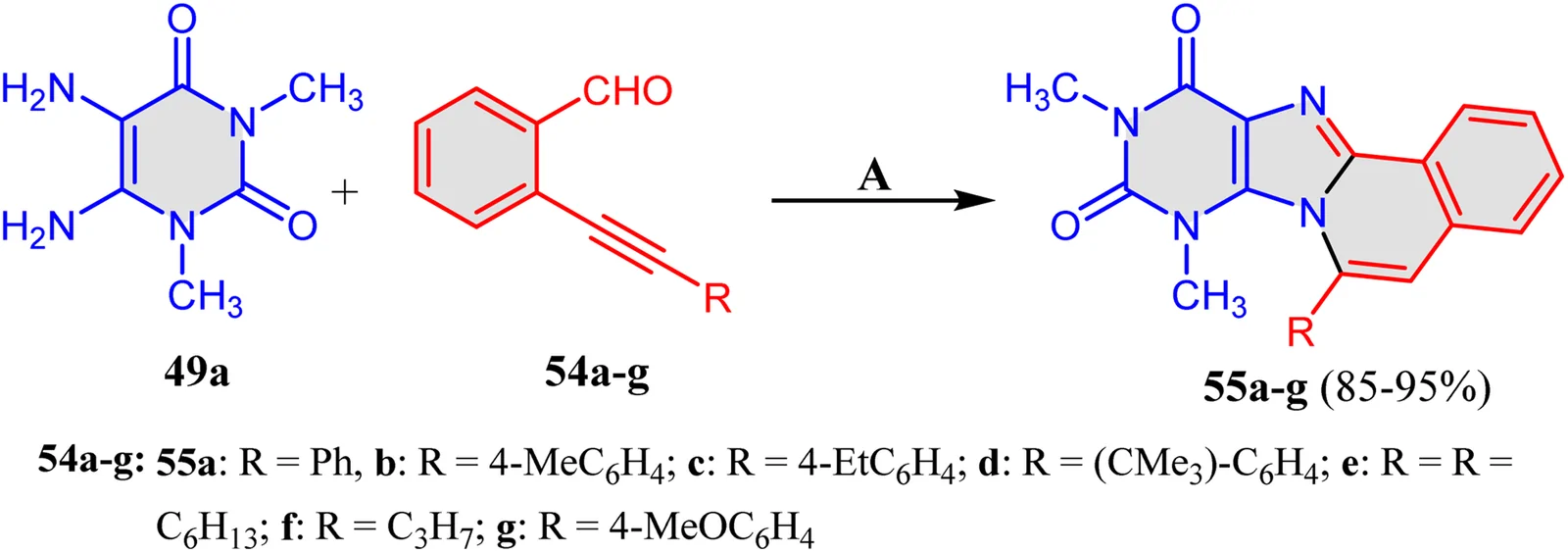
Synthesis of 8,10-dimethyl-6-substituted purino[8,9-*a*]isoquinoline-9,11(8*H*,10*H*)-diones 55a–g. Reagents and conditions: A; DMF, CuI, reflux.

Furthermore, 5,6-diamino-1,3-dimethylpyrimidine-2,4(1*H*,3*H*)-dione (49a) was allowed to react with 2-(ethynyl)-benzaldehydes 54a–g catalyzed by CuI at refluxing DMF. The reaction proceeded to obtain 8,10-dimethyl-6-substituted-purino[8,9-*a*]isoquinoline-9,11-diones 55a–g in good yields (Scheme 16).^115^

**Scheme 17:**
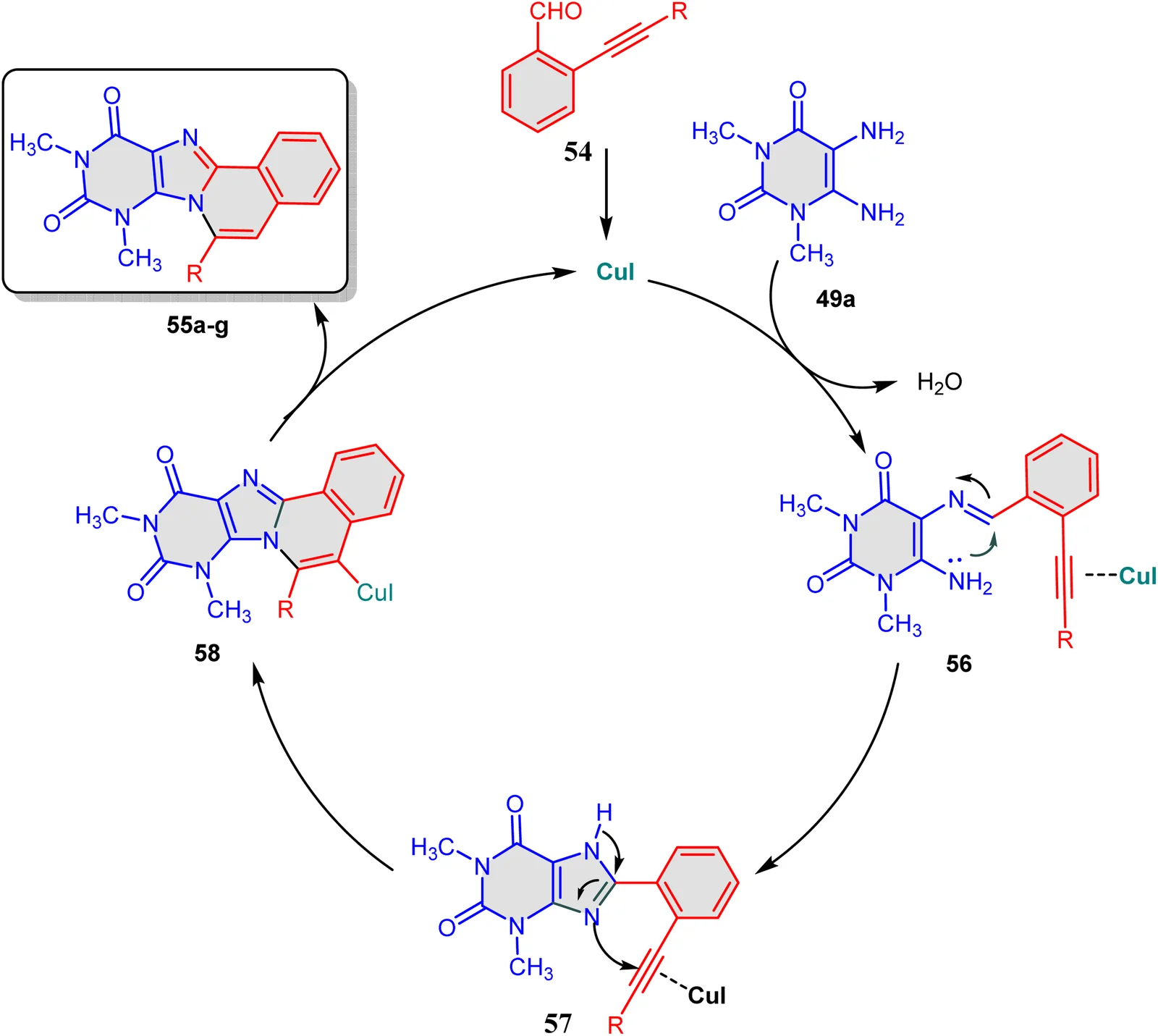
Suggested mechanism for the formation of 6-substituted purino[8,9-*a*]isoquinoline-9,11-diones 55a–g.

The suggested mechanism for the formation of compounds 55a–g is illustrated in Scheme 17. Initially, imine 56 was produced through a reaction between 5,6-diamino-1,3-dimethyluracil (49a) and *o*-alkynyl aromatic aldehydes 54. Subsequently, CuI promoted the nucleophilic attack of the NH_2_ group on the imine carbon. Furthermore, aerial oxidation occurred on imine 56 to produce intermediate 57. This was followed by a second intramolecular nucleophilic attack of the unsaturated imidazole nitrogen atom on the activated alkyne to form intermediate 58, while the cyclized products 55a–g were produced after the protonation process of intermediate 58.^115^

**Scheme 18:**
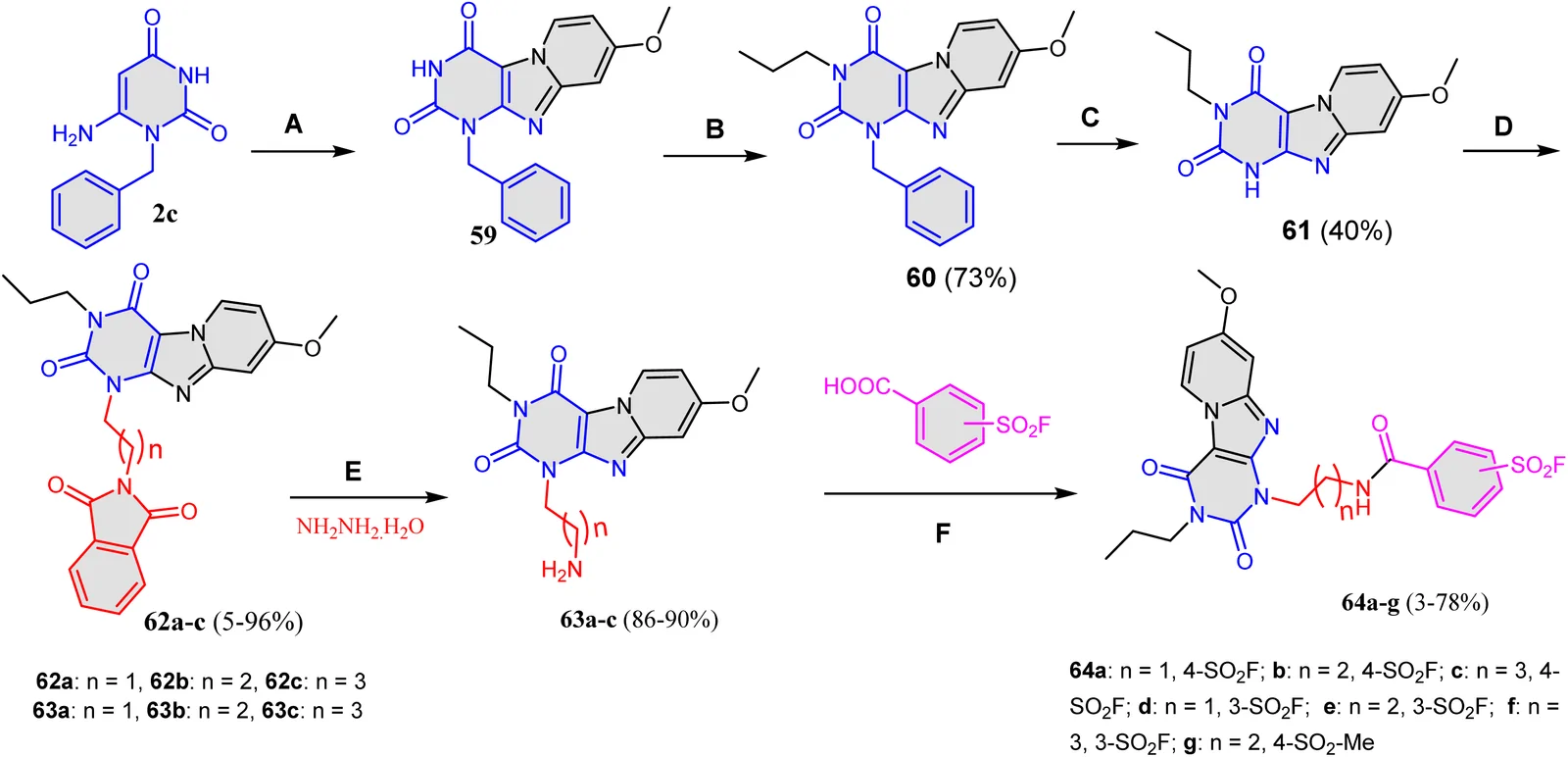
Multistep reaction for the synthesis of hybrids 64a–g. Reagents and conditions: A; (i) NBS, MeCN, 80 °C, 1 h. (ii) 4-methoxypyridine, 80 °C, overnight. B; ^*n*^Pr-Br, DBU, MeCN, 80 °C. C; Pd(OH)_2_/C, HCOONH_4_, EtOH, reflux. D; *N*-bromoalkyl phthalamide, K_2_CO_3_, DMF, 100 °C, E; N_2_H_4_·H_2_O, MeOH, reflux. F; SOCl_2_, K_2_CO_3_, dry DMF, 40 °C.

A multi-step pot reaction for the synthesis of compounds 64a–g was developed by Yang and co-workers,^32^ as summarized in Scheme 18. They started by reacting 1-benzyl-6-aminouracil (2c) with 4-methoxypyridine in acetonitrile (MeCN) containing *N*-bromosuccinimide (NBS) to give 1-benzyl-8-methoxypyrido[2,1-*f*]purine-2,4(1*H*,3*H*)-dione (59). Compound 59 was then alkylated using *n*-propyl bromide (^*n*^Pr-Br) to produce compound 60. Subsequently, an elimination of the benzyl group using palladium hydroxide (Pd(OH)_2_) gave the corresponding xanthine scaffold 61 in 40% yield. Thereafter, compound 61 was allowed to react with *N*-bromoalkyl phthalamide in DMF at 100 °C to give compounds 62a–c. The reaction of 62a–c with hydrazine hydrate led to the formation of the corresponding amines 63a–c. Eventually, the reaction of compounds 63a–c with various benzoic acids afforded the desired products of 64a–g (Scheme 18).^32^

**Scheme 19:**
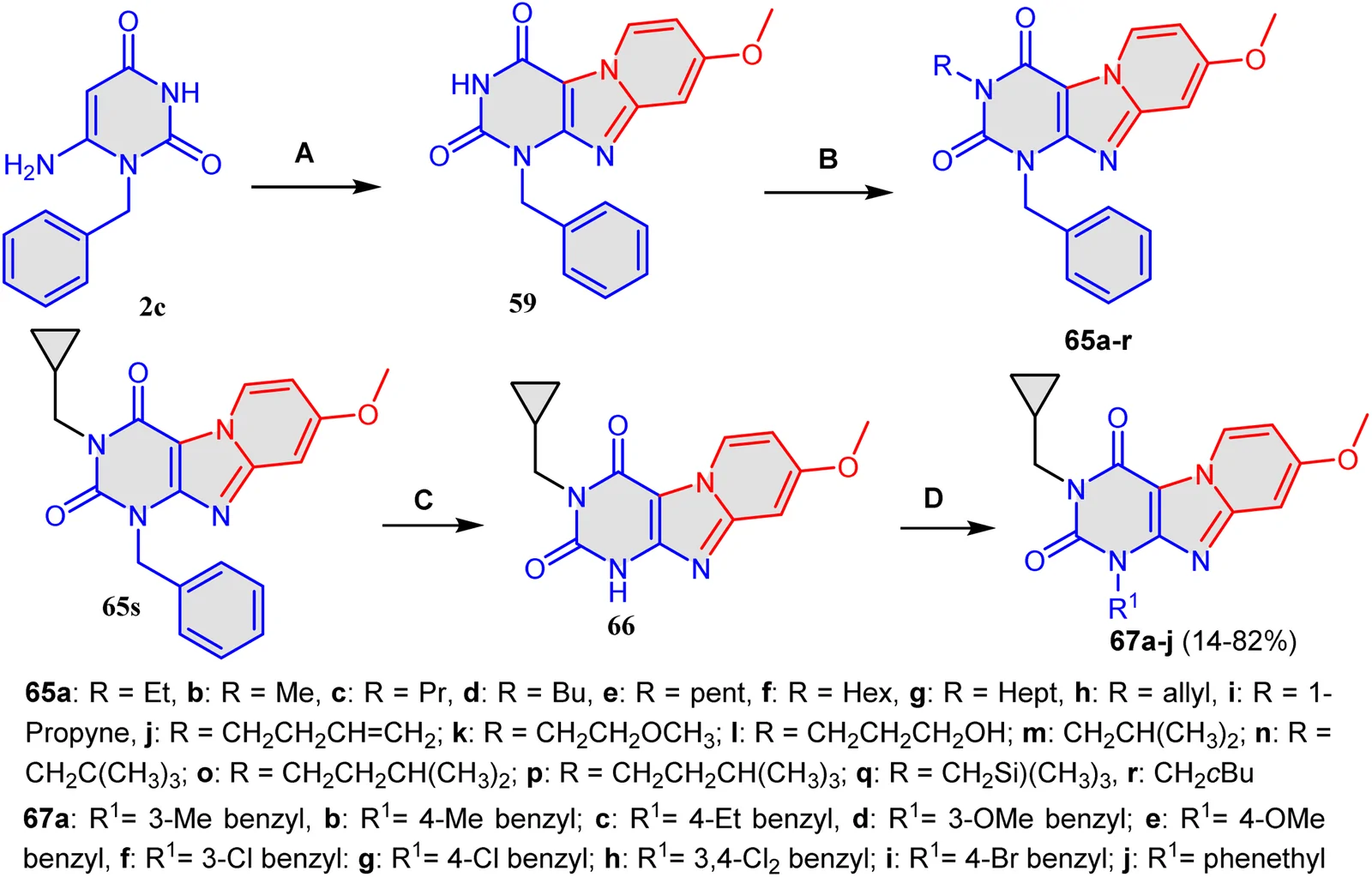
Synthesis of xanthine derivatives 67a–j. Reagents and conditions: A; (i) NBS, MeCN, 80°^o^C, 1 h. (ii) 4-methoxypyridine, 80 °C, overnight. B; R–Br, DBU, MeCN, 80 °C. C; Pd(OH)_2_, ammonium formate, EtOH, reflux, overnight. D; R^1^-Br, K_2_CO_3_, DMF, overnight.

Xia *et al.*^116^ prepared compound 59, which was further alkylated using different alkyl bromides with 1,8-diazabicyclo[5.4.0]undec-7-ene (DBU) and MeCN at 80 °C, followed by heating in 4-methoxypyridine at 80 °C, to give 1-benzyl-8-methoxypyrido[2,1-*f*]purine-2,4(1*H*,3*H*)-dione (59). Alkylation of *N*-3 in 59 by various alkyl halides using DBU as a catalyst afforded 65a–r (Scheme 19). In the case of 3-(cyclopropylmethyl)pyrido[2,1-*f*]purine (65s), the benzyl group in *N*-1 was removed *via* the reaction with Pd(OH)_2_ and ammonium formate in refluxing EtOH to give 3-(cyclopropylmethyl)-8-methoxypyrido[2,1-*f*]purine-2,4(1*H*,3*H*)-dione (66). Compounds 67a–j were then obtained, and *N*-1 was alkylated by treating 66 with different alkyl bromides in a mixture of DMF and K_2_CO_3_ (Scheme 19). Benzyl-3-propyl-1*H*,3*H*-pyrido[2,1-*f*]purine-2,4-dione derivative 65c was illustrated as a lead compound of 65a–s since it exhibits a *K*_i_ value of 4.0 ± 0.3 nM against the hA_3_ receptor. Generally, pyrido[2,1-*f*]purine-2,4-dione derivatives 65a–s have been described as a family of adenosine receptor antagonists.

#### 2.2.5. Synthesis of pyrido[2,3-*d*]pyrimidine derivatives

**Scheme 20:**
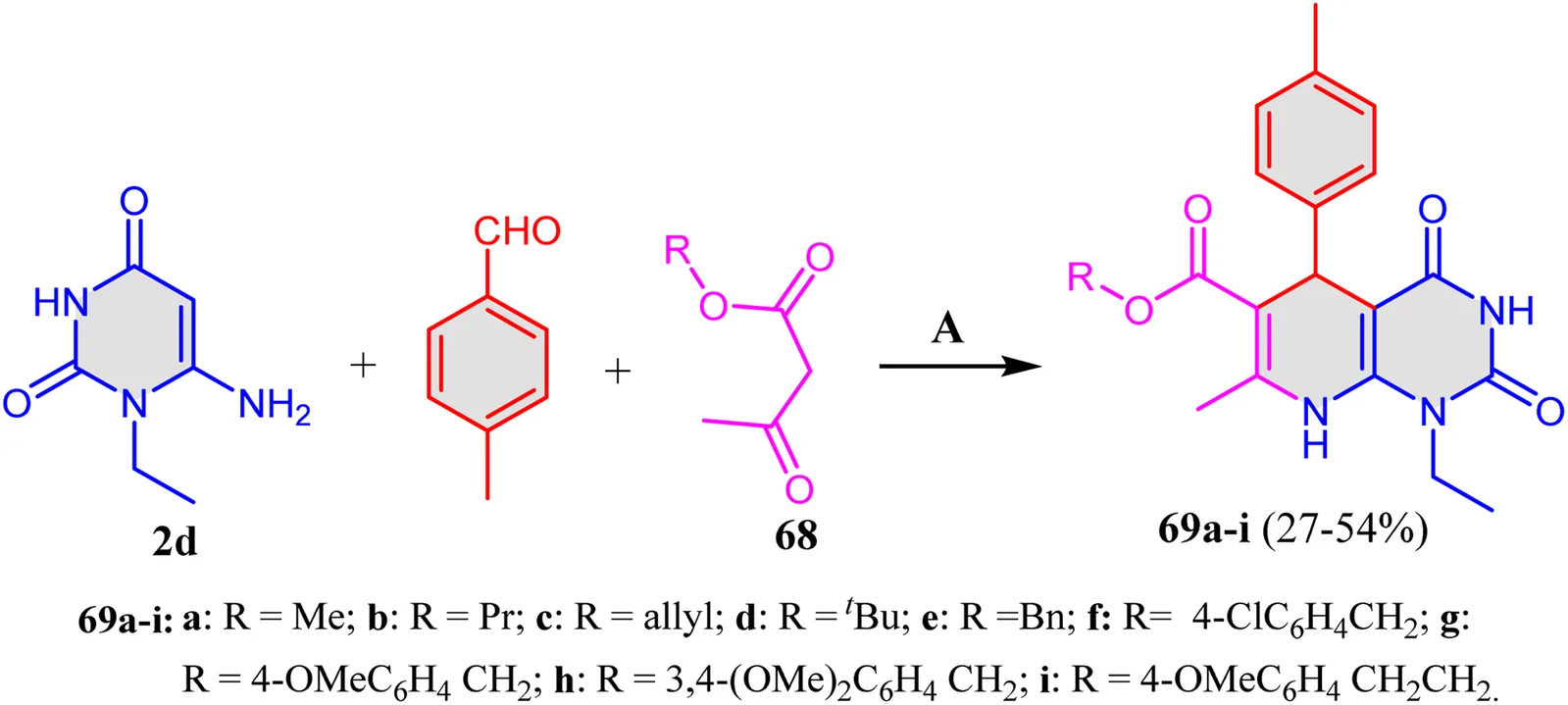
Synthesis of pyrido[2,3-*d*]pyrimidine-6-carboxylates 69a–i. Reagents and conditions: A; AcOH, reflux.

A three-component one pot synthesis succeeded in obtaining substituted 1-ethyl-*N*-7-methyl-2,4-dioxo-5-*p*-tolyl-1,2,3,4,5,8-hexahydropyrido[2,3-*d*]pyrimidine-6-carboxamides 69a–i in 27–54% yields.^117^ The latter series was established *via* the reaction of a mixture of 6-amino-1-ethyluracil (2d), 4-methylbenzaldehyde and 3-oxobutanoate ester derivatives 68 in refluxing AcOH (Scheme 20).^117^ The formed compounds 69a–i were examined for BRDT-1 and BRD4-1 inhibition (*i.e.* these proteins are involved in regulating gene expression, and their inhibition is explored as a potential therapeutic strategy for various diseases) using an Alpha Screen assay. Benzyl ester derivatives 69e revealed the greatest affinity for BRD4-1 and BRDT-1. Profiling across bromodomains showed a high selectivity of 69e, where R = Bn in case of the BET bromodomain family (Table 11).^117^

**Table 11:** Structures and affinity profiles for compounds 69a–i

Compd	R	IC_50_ (μM) BRDT-1	IC_50_ (μM) BRD4-1
69a	Me	5.9	5.4
69b	Propyl	7.9	5.5
69c	Allyl	4.7	3.1
69d	*t* Bu	10	5.5
69e	Bn	0.79	0.97
69f	4-ClC_6_H_4_CH_2_	14	9.4
69g	4-OMeC_6_H_4_CH_2_	15	11
69h	3,4-(OMe)_2_C_6_H_4_CH_2_	7.0	4.5
69i	4-OMeC_6_H_4_CH_2_CH_2_	11	9.1
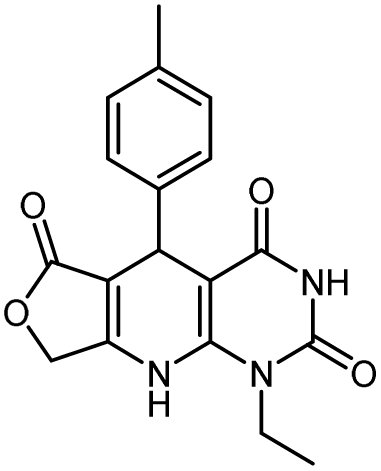		5.4	4.1
(+)-JQ1^a^		0.16	0.050

a (+)-JQ1 was used as the positive control. All compounds were tested once in duplicate.

##### 2.2.5.1 Structure activity relationship

**Fig. 8:**
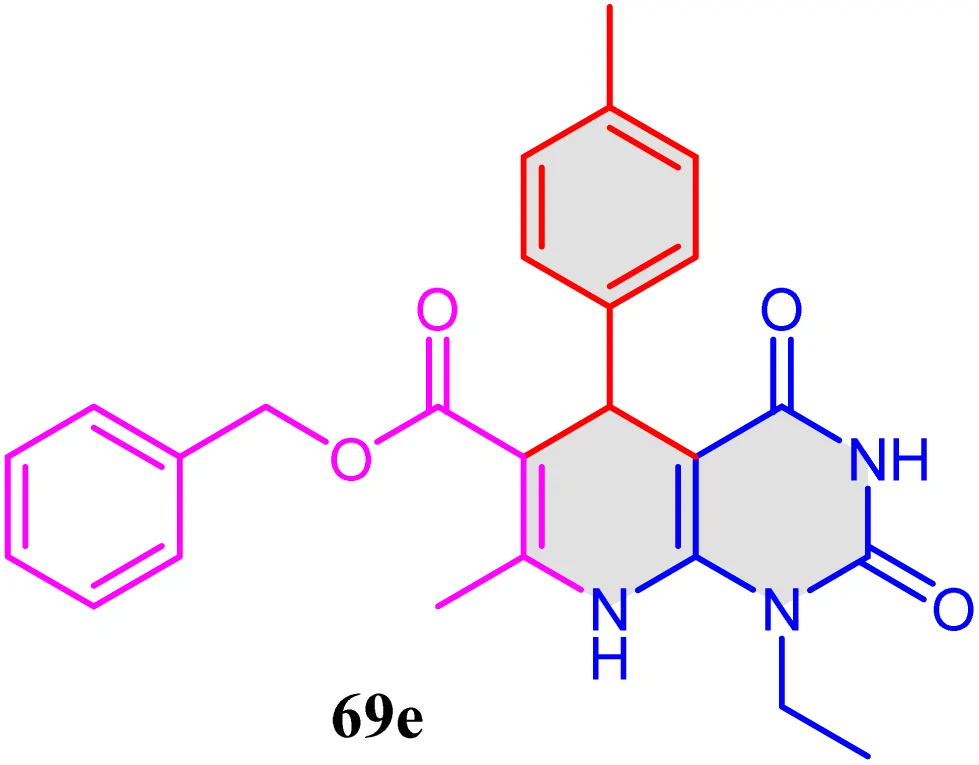
Structure of compound 69e with high affinity for BRDT-1 and BRD4-1.

The results in Table 11 show that compounds 69a, 69b, and 69c, which have linear aliphatic chains, exhibited moderate activity, while the *tert*-butyl substituent 69d decreased 2 times in BRDT-1 affinity compared with the lactone. In the case of the benzyl group 69e (Fig. 8), the affinity was extremely enhanced for BRDT-1 and BRD4-1 (IC_50_ = 0.79 and 0.97, respectively). In addition, adding an (OMe) group or a chlorine atom to the aryl ring resulted in a reduction in affinity, as in the 69f and 69g derivatives. Furthermore, a comparison between 69g, 69h, and 69i demonstrated that di-substitution and linker extension had only a small impact on affinity.^117^

**Scheme 21:**
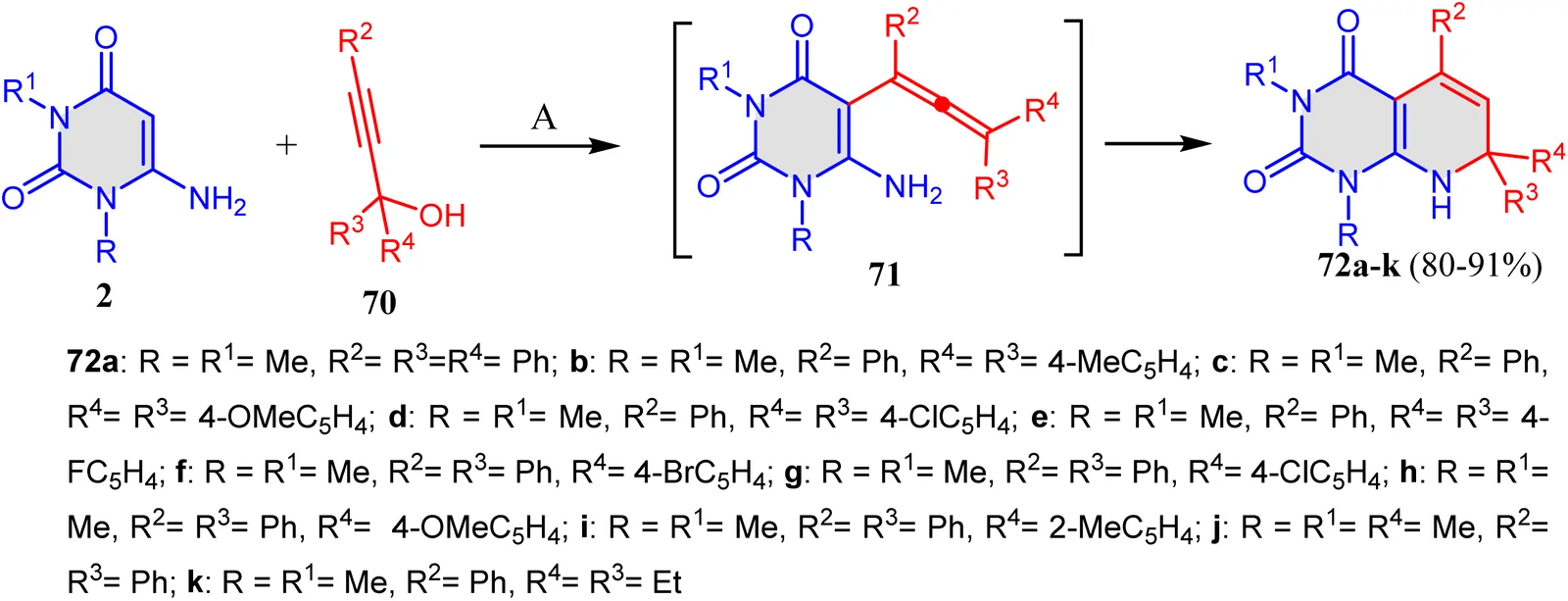
Mechanochemical reaction between 6-aminouracil 2 and propargyl alcohols 70. Reagents and conditions: A; HFIP/*p*-TsOH, 1–2 h.

In 2024, Yaragorla,^118^ developed a simple reaction for the synthesis of pyrido[2,3-*d*]pyrimidine scaffolds 72a–k during the reaction of 6-aminouracils 2 with propargyl alcohols 70. The reaction proceeded *via* a [3 + 3] cascade annulation through the allenylation of uracil, followed by 6-*endo* trig cyclization in the presence of hexafluoroisopropanol (HFIP) and *p*-toluene sulfonic acid (*p*-TsOH). The final products were obtained through the formation of intermediate 71, as illustrated in Scheme 21.^118^

**Scheme 22:**
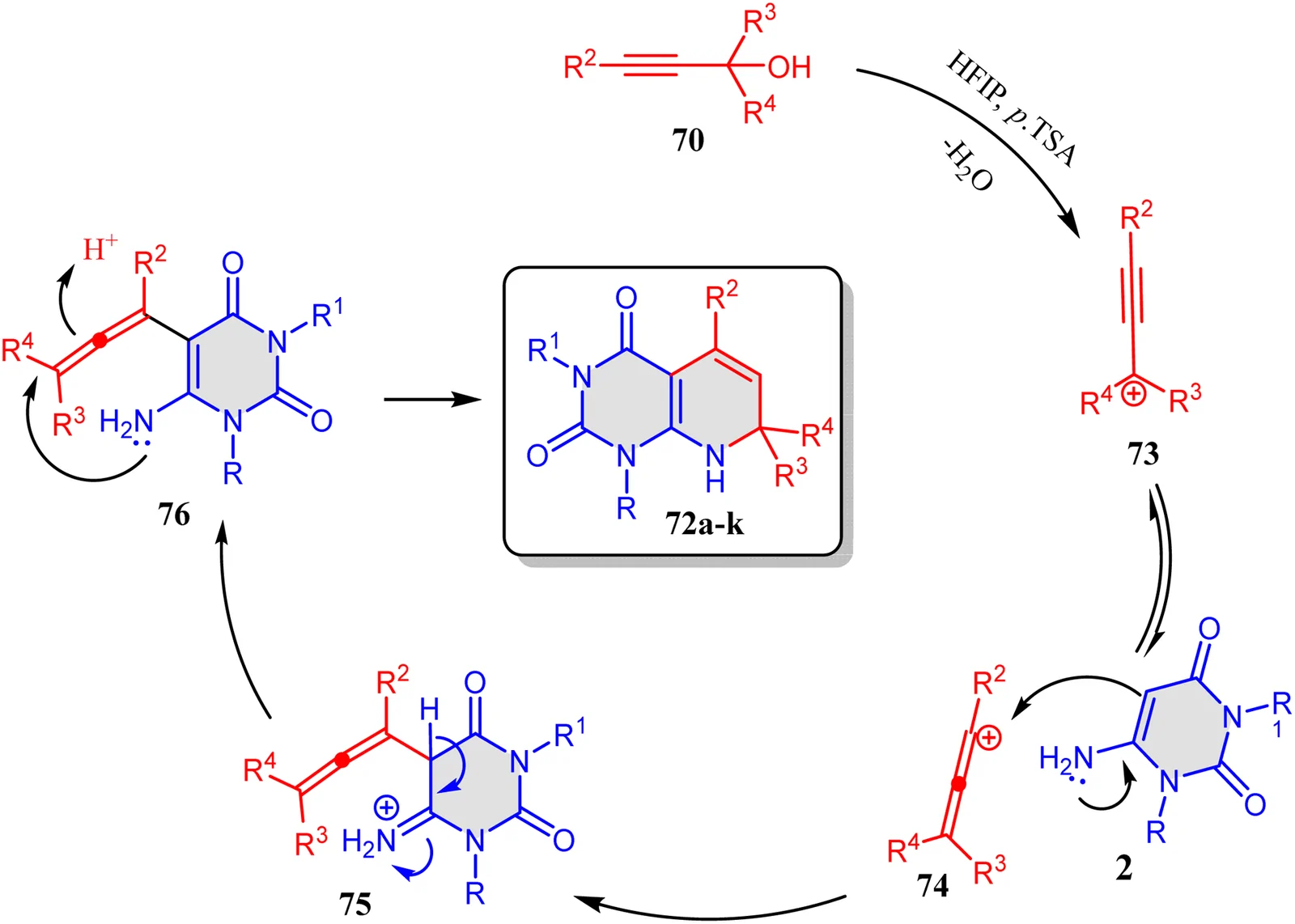
Suggested mechanism for the formation of compounds 72a–k.

A plausible mechanism for the synthesis of compounds 72a–k is shown in Scheme 22. First, the catalyst mixture (HFIP/*p*-TSA) acts as a Brønsted acid and protonates propargyl alcohols 70 to produce propargylic cation 73 through the elimination of a water molecule. Intermediate 73 was then converted to an allenyl cation (74). Subsequently, the alkylation of uracil proceeded to afford the allene intermediate 75. Finally, the targeted pyrido[2,3-*d*]pyrimidines 72a–k were formed through the intramolecular 6-*endo-trig*-cyclization of 76 (Scheme 22).^118^

**Scheme 23:**
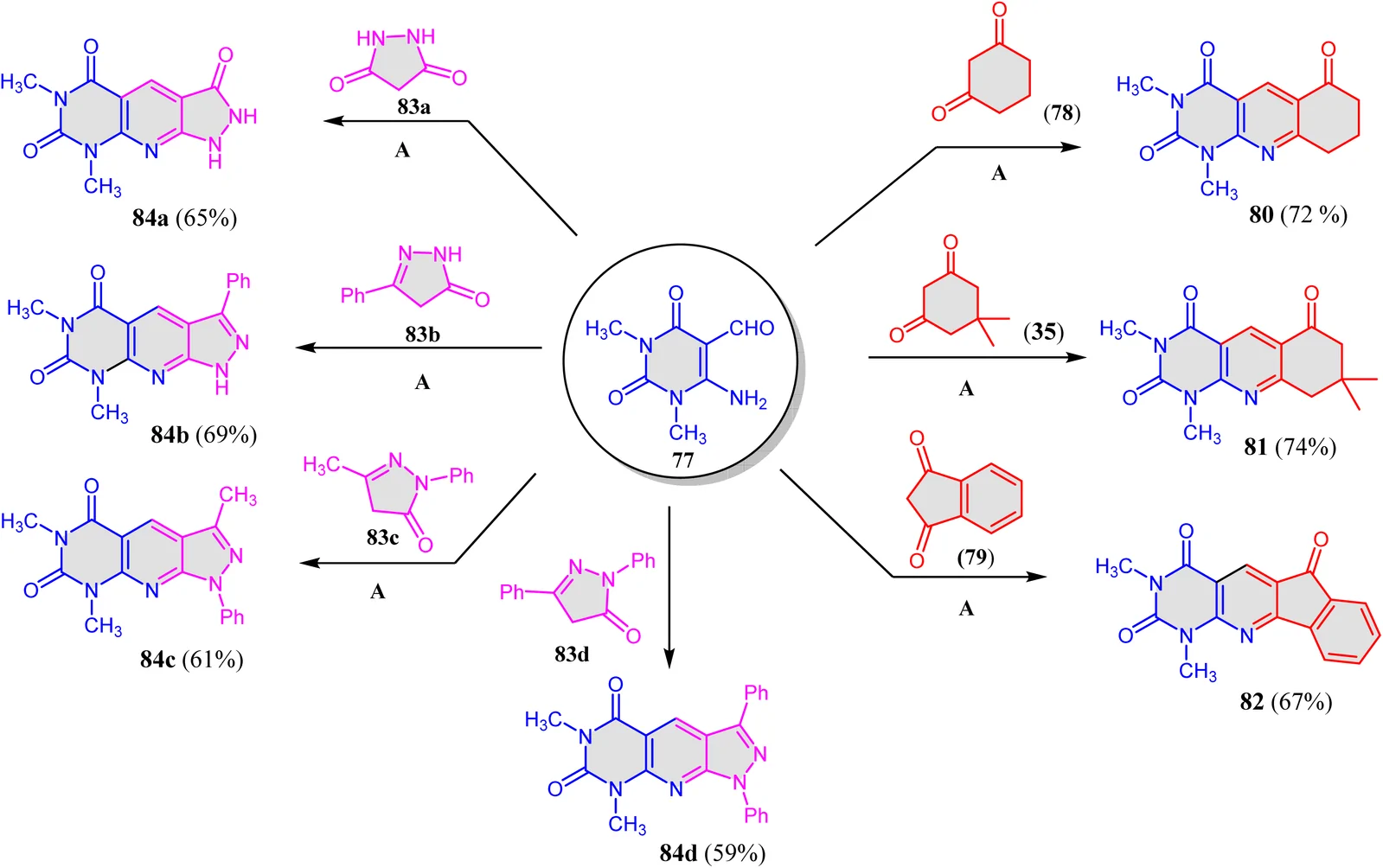
Reaction of 6-amino-5-formyluracil 77 with cyclic carbon nucleophiles. Reagents and conditions: A; DMF/DBU, reflux. 120 min.

Hussain *et al.*^50^ utilized 6-amino-5-formyluracil (77) as a precursor for several heterocyclic compounds. Scheme 23 described the reaction of 77 with several active methylene compounds, such as cyclohexane-1,3-dione (78), dimedone (35) and 1*H*-indene-1,3(2*H*)-dione (79), to form pyrido[2,3-*d*]pyrimidine scaffolds 80, 81, and 82, respectively. However, compound 77 was allowed to react with pyrazole derivatives 83a–d to obtain the corresponding pyrazolo[4′,3′:5,6]pyrido[2,3-*d*]pyrimidines derivatives 84a–d (Scheme 23). All the reactions were performed in DMF and catalyzed by DBU.^51^

**Scheme 24:**
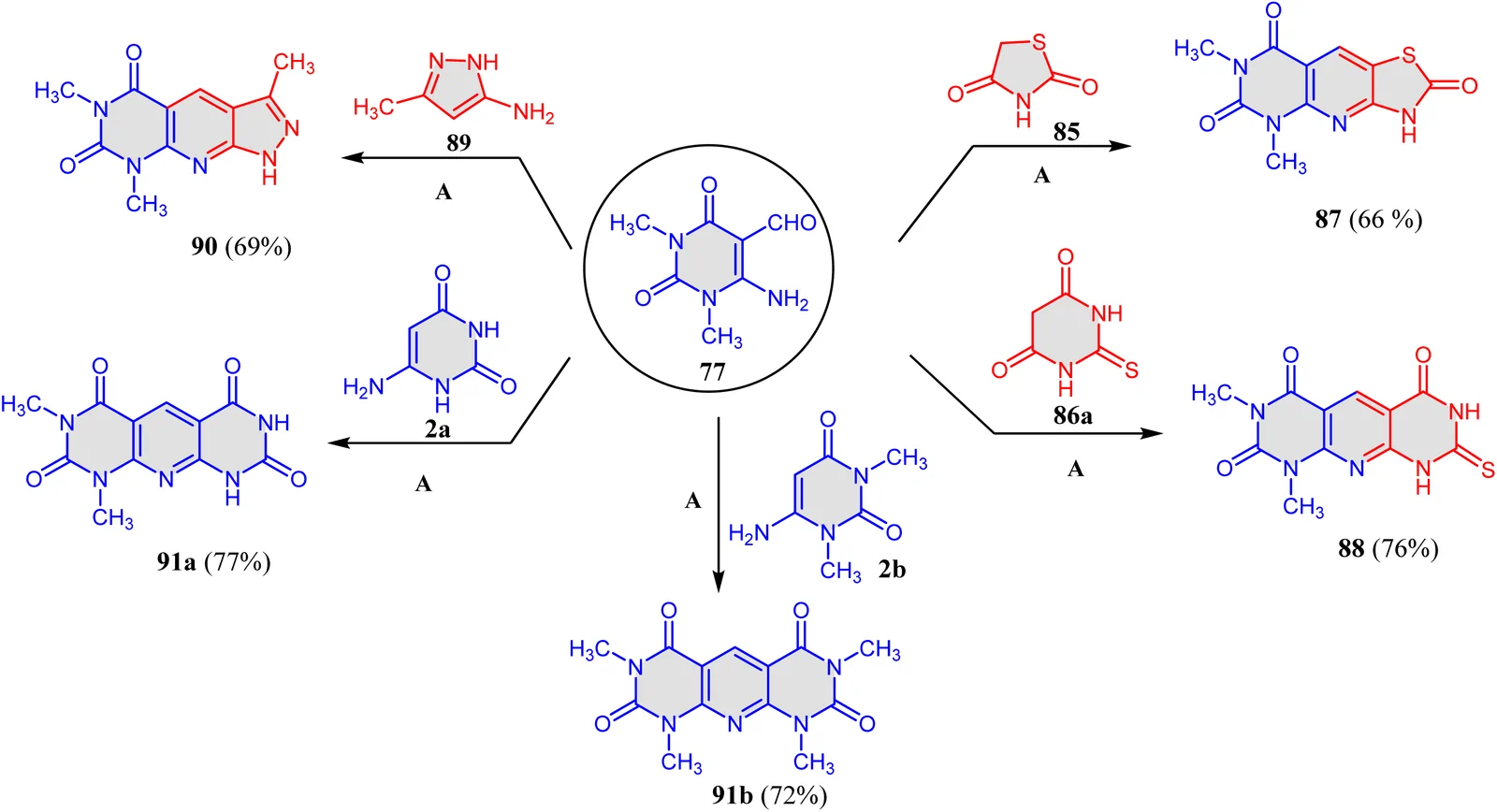
Synthesis of compounds 87, 88, 90 and 91a,b. Reagents and conditions: A; DMF/DBU, reflux. 120 min.

The same group utilized compound 77 in the synthesis of other heterocycles during its reaction with various compounds, such as thiazolidine-2,4-dione (85) and 2-thioxo-dihydropyrimidine-4,6(1*H*,5*H*)-dione (86a). The corresponding 5,7-dimethylthiazolo[5′,4′:5,6]-pyrido[2,3-*d*]pyrimidine-2,6,8(3*H*,5*H*,7*H*)-trione (87) and 2-thioxodihydropyrimidine-4,6-(1*H*,5*H*)-dione (88) were obtained, respectively. When compound 77 was reacted with 3-methyl-1*H*-pyrazol-5-amine (89), the reaction afforded 3,6,8-trimethyl-1,8-dihydro-5*H*-pyrazolo[4′,3′:5,6]pyrido[2,3-*d*]pyrimidine-5,7(6*H*)-dione (90). The reaction of 77 with 2a,b afforded pyrido[2,3-*d*:6,5-*d*′]dipyrimidines 91a,b (Scheme 24).^50^

**Scheme 25:**
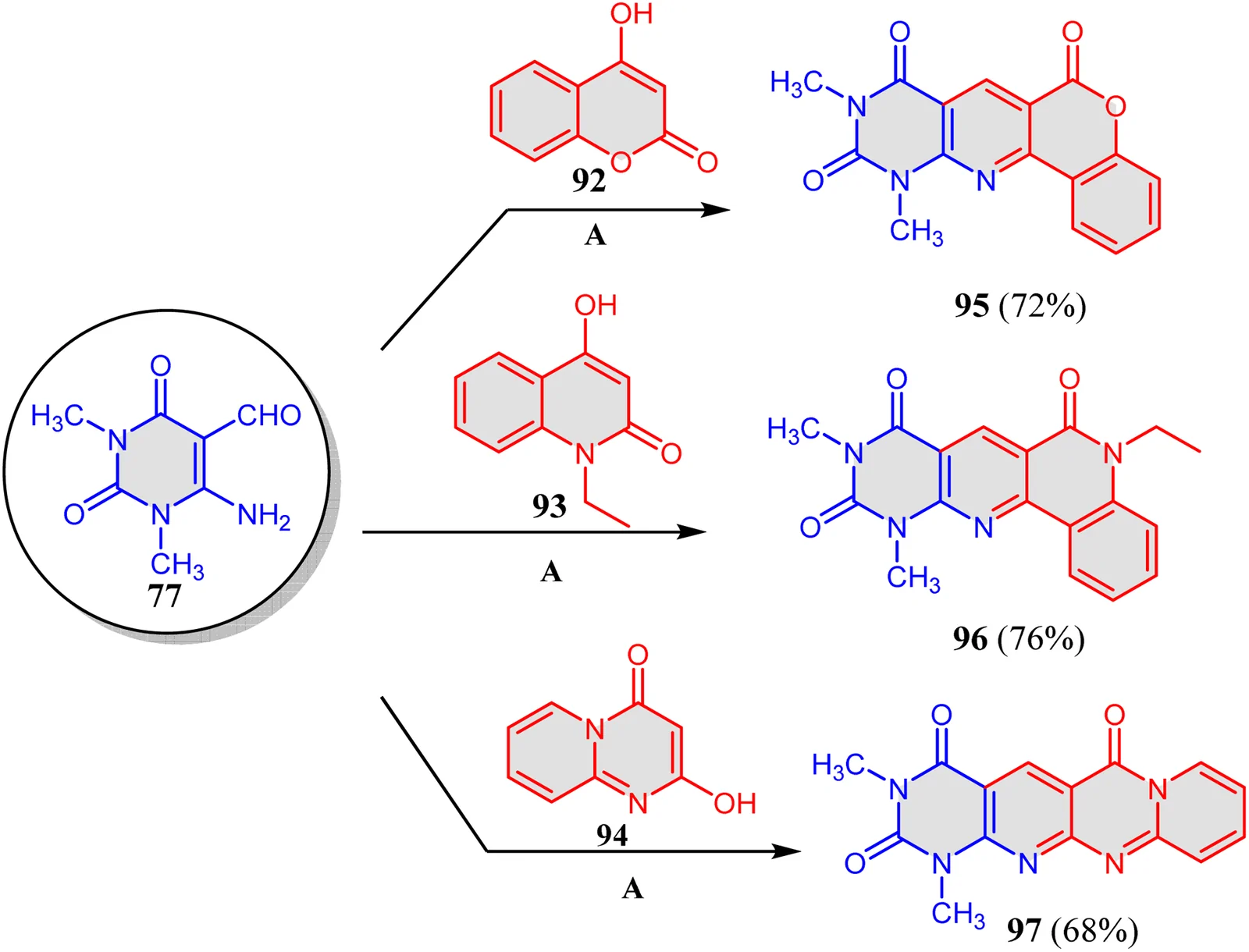
Reaction of 6-amino-5-formyluracil 79 with heterocyclic compounds. Reagents and conditions: A; DMF/DBU, reflux, 30 min.

In continuation, the reactions of 6-amino-5-formyluracil (77) with 4-hydroxy-2*H*-chromen-2-one (92), 1-ethyl-4-hydroxyquinolin-2(1*H*)-one (93) and 2-hydroxy-4*H*-pyrido[1,2-*a*]pyrimidin-4-one (94) in DMF and DBU for 30 min produce pyrido[2,3-*d*]pyrimidine building blocks 95, 96 and 97, respectively, as depicted in Scheme 25.^50^

The formed products were evaluated owing to their antimicrobial activity against Gram-positive bacteria, namely *Bacillus subtilis* (ATCC6635) and *Staphylococcus aureus* (ATCC25923), as well as Gram-negative bacteria, namely *E. coli* (ATCC 25922) and *Salmonella typhimurium* (ATCC 14028). Moreover, they were tested for yeast (*Candida albicans* ATCC 10231) and fungus (*Asperigillus fumigatus*). The majority of the compounds demonstrated good to excellent activities against human hepatocellular carcinoma cell lines (HePG-2) and colon carcinoma cell lines (HCT-116) (Tables 12 and 13). Compounds 84a and 88, 90 and 91a displayed higher antiproliferative activity (from 2.68 to 16.82 μg mL^−1^) against two types of cancer cell lines compared to the standard drug (5-fluorouracil).^50^

**Table 12:** *In vitro* antimicrobial evaluations of synthesized products (Schemes 23–25) at 500 and 1000 μg mL^−1^^a^

Compd	Mean of zone diameter (mm)											
	Gram-positive bacteria				Gram-negative bacteria				Yeasts and fungi			
	*S. aureus*		*B. subtilis*		*S. typhimurium*		*E. coli*		*C. albicans*		*A. fumigatus*	
	1000 μg mL^−1^	500 μg mL^−1^	1000 μg mL^−1^	500 μg mL^−1^	1000 μg mL	500 μg mL^−1^	1000 μg mL^−1^	500 μg mL^−1^	1000 μg mL^−1^	500 μg mL^−1^	1000 μg mL^−1^	500 μg mL^−1^
80	15 I	12 I	17 I	13 I	13 I	10 I	11 L	8 L	16 I	13 I	14 I	10 I
81	18 I	14 I	15 I	12 I	16 I	12 I	10 L	7 L	13 I	10 I	17 I	12 I
82	16 I	10 I	19 I	15 I	15 I	12 I	12 L	8 L	15 I	11 I	16 I	12 I
84a	20 I	15 I	17 I	13 I	18 I	13 I	16 I	13 I	17 I	13 I	17 I	11 I
84b	17 I	12 I	14 I	10 I	12 I	9 I	14 I	10 I	14 I	10 I	13 I	9 I
84c	14 I	11 I	19 I	15 I	17 I	13 I	10 L	6 L	16 I	11 I	18 I	13 I
84d	13 I	10 I	14 I	10 I	15 I	11 I	13 I	10 I	18 I	14 I	14 I	10 I
87	33 H	23 H	32 H	21 H	34 H	25 H	33 H	24 H	30 H	22 H	33 H	24 H
88	18 I	13 I	18 I	13 I	19 I	14 I	11 L	8 L	26 H	19 H	26 H	18 H
90	16 I	11 I	17 I	15 I	16 I	12 I	14 I	10 I	19 I	14 I	18 I	14 I
91a	19 I	15 I	14 I	10 I	13 I	10 I	16 I	12 I	24 H	19 H	27 H	20 H
91b	15 I	10 I	16 I	11 I	15 I	11 I	15 I	10 I	28 H	21 H	25 H	19 H
95	22 I	16 I	19 I	15 I	17 I	14 I	13 I	9 I	19 I	15 I	15 I	12 I
96	30 H	21 H	27 H	21 H	27 H	20 H	26 H	18 H	20 I	16 I	17 I	13 I
97	31 H	22 H	26 H	19 H	19 I	14 I	18 I	13 I	26 H	20 H	21 I	15 I
S	35	26	35	25	36	28	38	27	35	28	37	26

a S: Standard drugs such as chloramphencol in the case of Gram-positive bacteria, cephalothinin in the case of Gram-negative bacteria and cycloheximide in the case of yeast and fungi.

**Table 13:** IC_50_ values of the synthesized compounds in Schemes 23–25 against human tumor cells after 24 h of incubation

Compd	(HepG-2 cells) IC_50_(μg mL^−1^)	HCT-116 cells IC_50_(μg mL^−1^)
80	26.55 ± 1.76	21.10 ± 1.36
81	23.07 ± 1.45	14.62 ± 1.01
82	12.54 ± 0.95	35.45 ± 2.34
84a	2.68 ± 0.24	13.58 ± 0.96
84b	9.16 ± 0.76	15.78 ± 1.12
84c	14.62 ± 1.00	19.03 ± 1.32
84d	25.52 ± 1.56	35.25 ± 2.31
87	11.37 ± 0.88	15.78 ± 1.11
88	5.92 ± 0.59	10.33 ± 0.82
90	5.81 ± 0.57	16.82 ± 1.21
91a	4.88 ± 0.41	11.37 ± 0.87
91b	11.39 ± 0.90	14.61 ± 1.02
95	26.55 ± 1.66	47.18 ± 2.94
96	15.78 ± 1.09	28.76 ± 1.87
97	12.54 ± 0.93	44.98 ± 2.86
5-FU	6.44 ± 0.61	21.5 ± 1.35

##### 2.2.5.2 Structure activity relationship

**Fig. 9:**
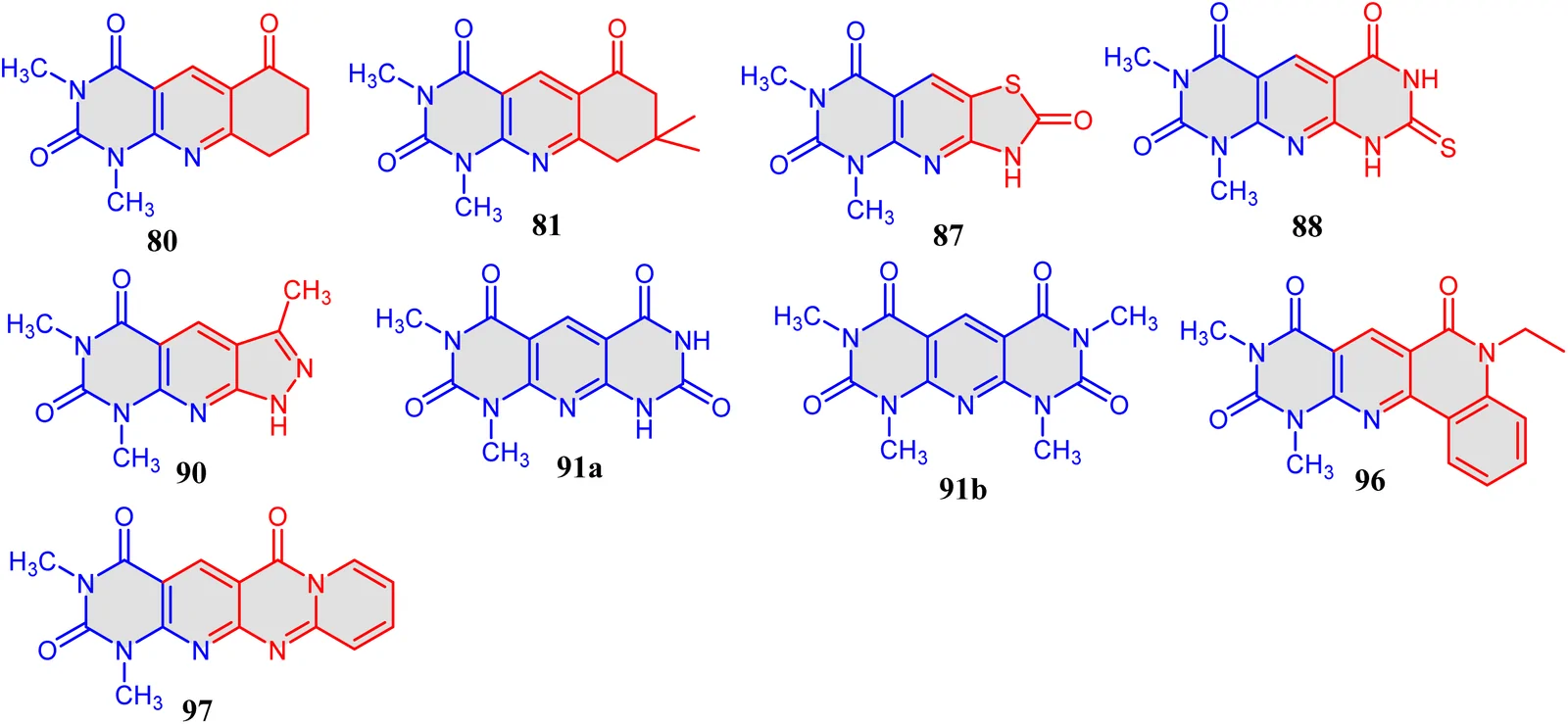
Structures of antimicrobial and anticancer compounds 90, 91a, 91b, 96 and 97.

The existence of an annulated system, namely thiazolo[5,4:5,6]pyrido[2,3-*d*]pyrimidine, may be the reason why compound 87 (Fig. 9) exhibited an inhibitory action that was closer to that of the reference drug. Compound 96 had significant action against Gram-negative bacteria, but compounds 96 and 97 (Fig. 9) demonstrated outstanding antimicrobial activity against Gram-positive bacteria. Additionally, compound 97 showed a significant level of effectiveness against *Candida albicans*, a kind of yeast. Compounds 88, 91a, and 91b (Fig. 9) had strong inhibitory impacts on fungus *A. fumigatus* and yeast *C. albicans*, which was attributed to the existence of a fused system called pyrimidopyridopyrimidine. It is interesting to note that compounds 88, 90, and 91a (Fig. 9) had more cytotoxic activity against both types of cancer cell lines than the standard drug. This might be explained by the fact that the same molecular frame contains pyrazole and pyrimidine annulated with a pyrido[2,3-*d*]pyrimidine backbone. Additionally, compounds 80, 81, 87, and 91a (Fig. 9) showed greater cytotoxic action against colon cancer (HCT-116) than the reference drug. Due to the presence of quinoline, pyrazolopyridine, thiazolopyridine, and pyrimidopyridine coupled with a 1,3-dimethyl-pyrimidine moiety, these compounds showed a moderately inhibiting impact on HepG-2 cells. More activity is displayed by free hydrogen in pyrazole and pyrimidine rings than by methyl or phenyl groups. Derivatives 96 and 97 (Fig. 9) had moderate efficacy against two of the cancer cell lines. The presence of indenopyridine, pyrazolopyridine, chromenopyridine, benzonaphthyridine, and pyrimidodipyridine moieties fused with the 1,3-dimethylpyrimidine moiety may be the cause of this.

**Scheme 26:**
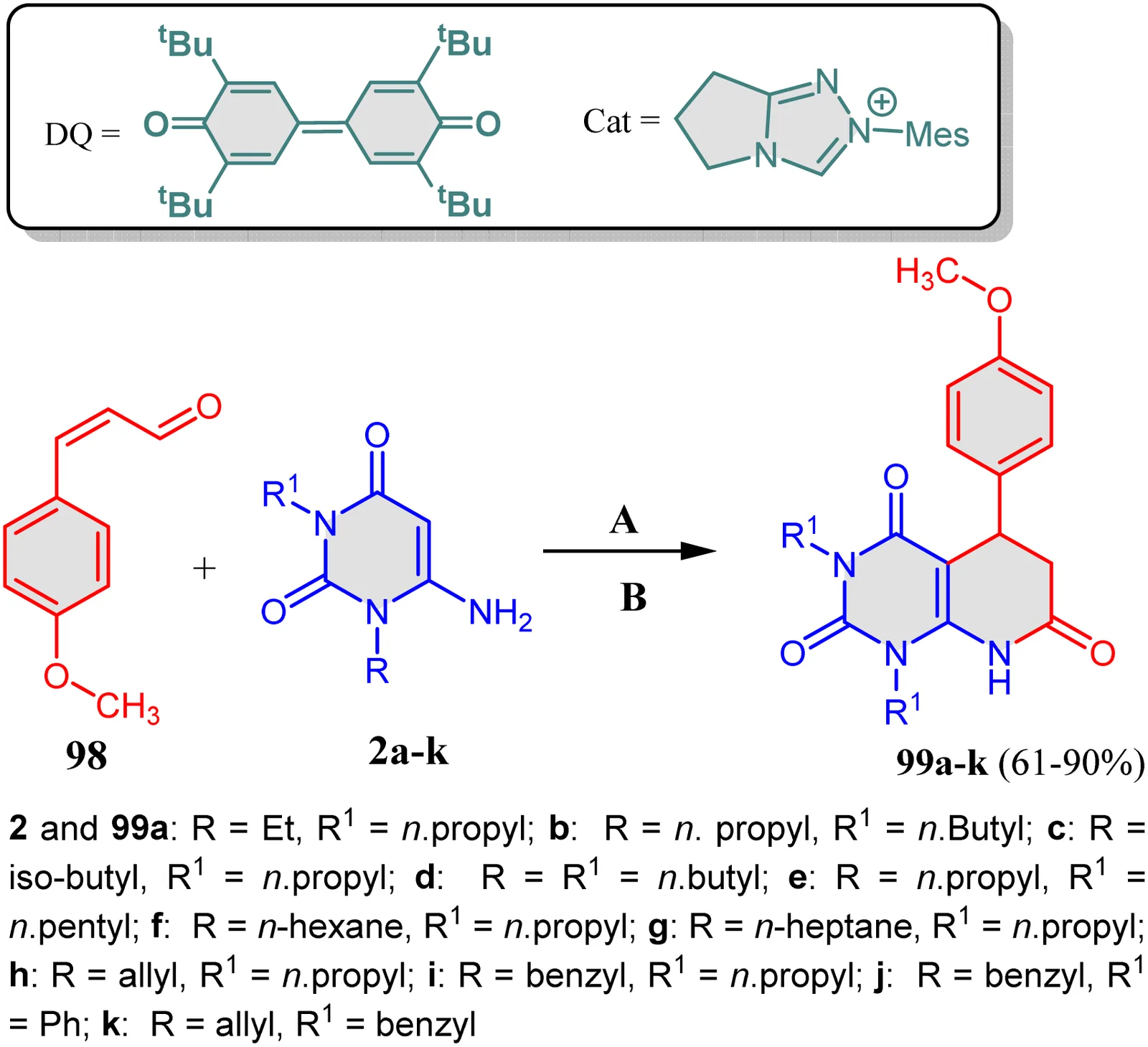
Synthesis of dihydropyrido[2,3-*d*]pyrimidines 99a–k. Reagents and conditions: A; DQ, cat. K_3_PO_4_, B; toluene, rt, 20 h.

Furthermore, a series of dihydropyrido[2,3-*d*]pyrimidines 99a–k were obtained in good yields (61–90%) *via* aza-Claisen rearrangement between 3-(4-methoxyphenyl)acrylaldehyde (98) and 6-amino-uracils 2. The reaction was catalyzed by K_3_PO_4_, and DQ was used as an oxidant reagent (Scheme 26).^119^

**Scheme 27:**
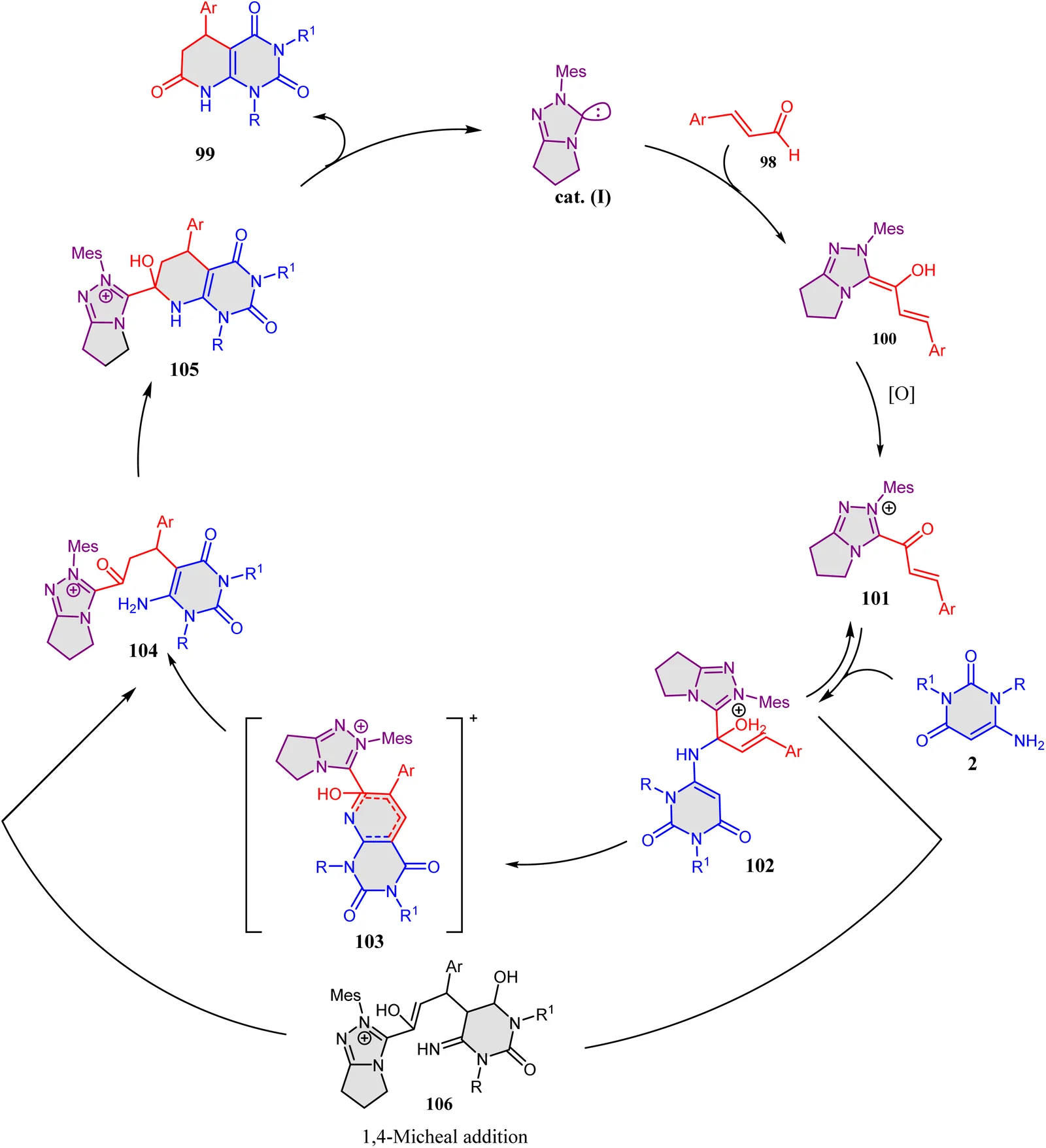
Suggested mechanism for the formation of compounds 99a–k.

Scheme 27 illustrates the suggested mechanism. The organo-catalyst was initially provided by the deprotonation of the triazolium salt. When catalyst I and compound 98 were added nucleophilically, intermediate 100 was obtained. This intermediate was then oxidized to form α,β-unsaturated acyl azolium 101. The 1,2-addition of cyclic enamine to acyl azolium 101 subsequently resulted in the formation of an *N*-acylation product. Through transition state intermediate 103, hemiaminal 102 then underwent aza-Claisen rearrangement. After this rearrangement, the catalyst was regenerated *vi*a intramolecular lactamization to afford adduct 105 and yield the end product 99. The nucleophilic addition of uracil enamine as a Michael acceptor in a 1,4-fashion to the α,β-unsaturated acyl azolium intermediate may also account for this catalyzed annulation. Subsequently, enol intermediate 106 underwent intramolecular acylation and proton transfer to yield targeted compounds 99a–k (Scheme 27).^119^

**Scheme 28:**
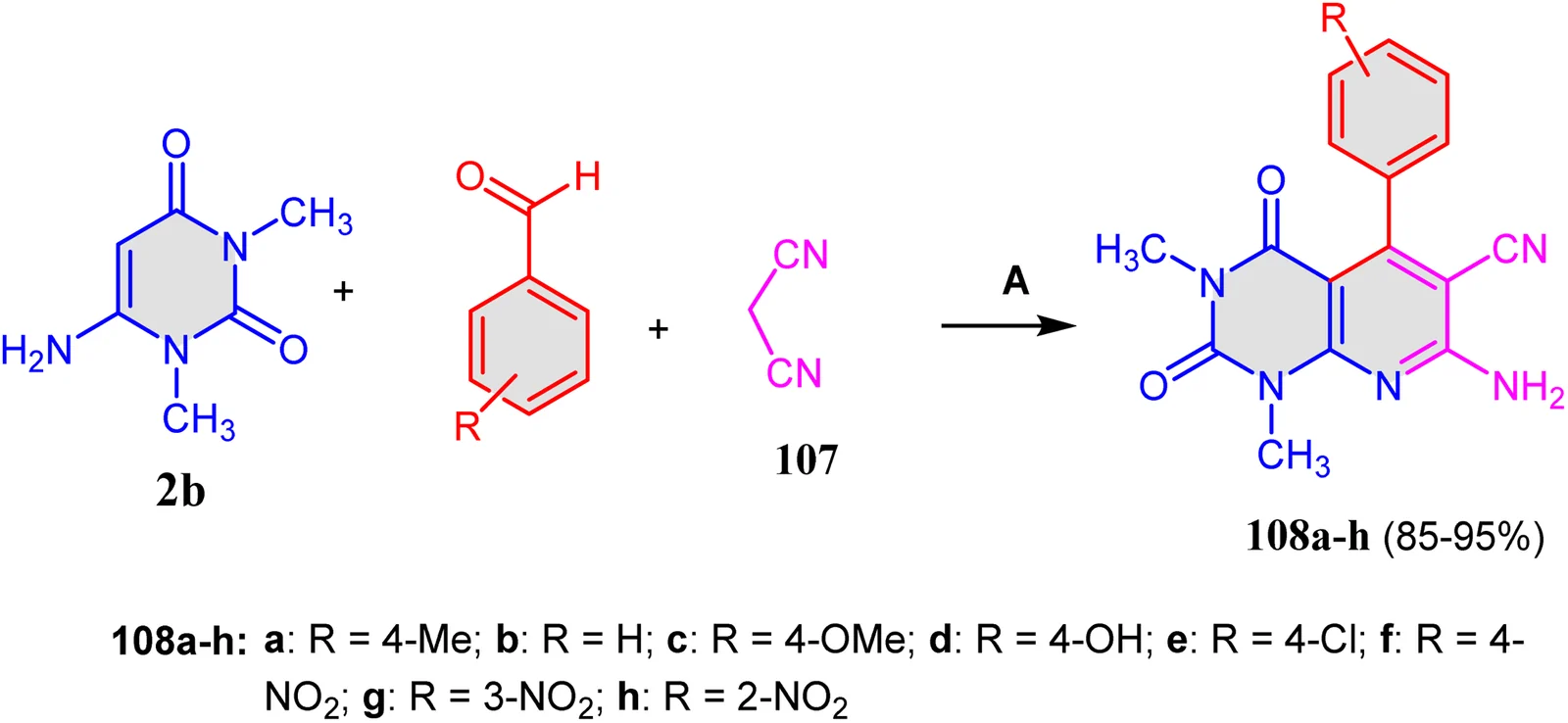
Synthesis of biologically active pyridopyrimidines 108a–h. Reagents and conditions: A = EtOH/H_2_O, Et_3_N.

Additionally, Dongre *et al.*^120^ used another environmentally friendly protocol to synthesize another series of pyrido[2,3-*d*]pyrimidine-6-carbonitrile derivatives 108a–h in excellent yields (85–95%) using EtOH/H_2_O and Et_3_N (Scheme 28).^120^

**Fig. 10:**
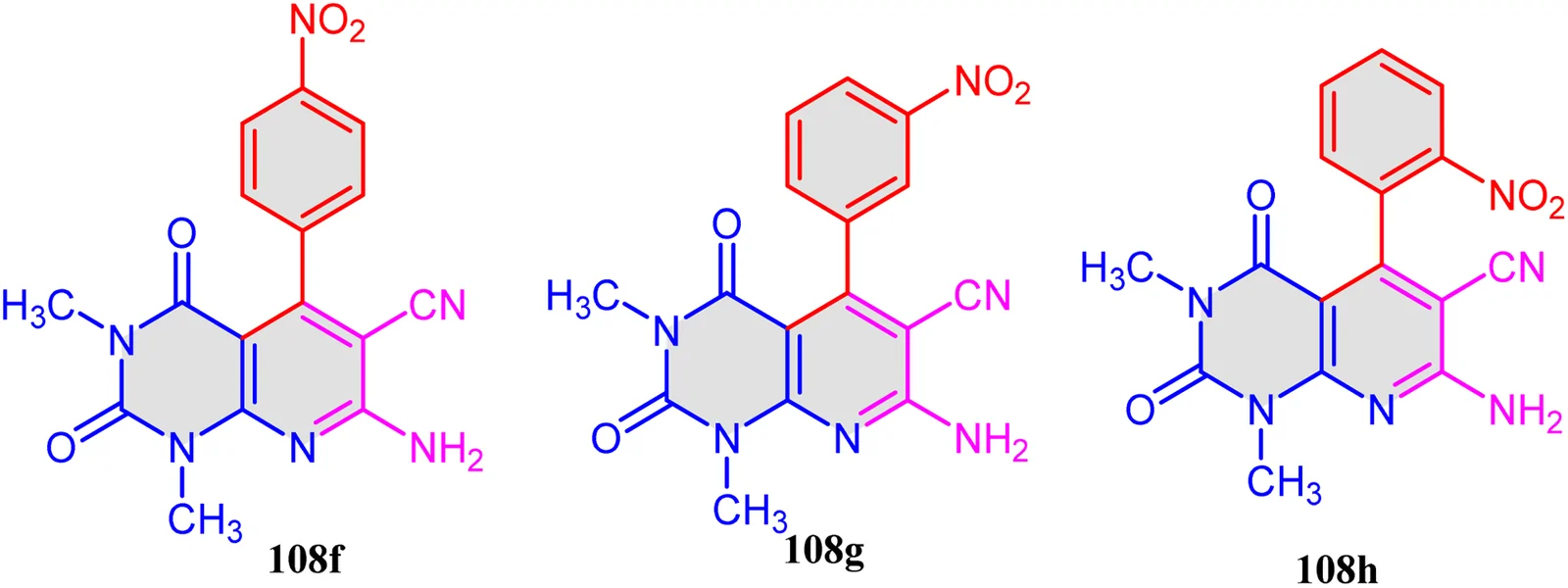
Structures of antibacterial molecules 108f–h.

The antibacterial activity of compounds 108a–h was evaluated *in vitro* against Gram-positive bacteria, such as *Staphylococcus* and *Bacillus cereus*, as well as against Gram-negative bacteria, such as *P. merabitis* and *S. maresens*. Maximum antibacterial efficacy against *Staphylococcus*, *B*. *cereus*, *P*. *merabitis*, and *S. maresens* was demonstrated by pyrido[2,3-*d*]pyrimidines 108a–d (Table 14). Electron-donating functionalities, such as benzaldehyde, –OH, –Me, and –OMe, attached to the phenyl ring of the fused pyridine skeleton of the annulated pyrido[2,3-*d*]pyrimidines were responsible for the extensive effects on the membrane potential associated with the bactericidal activity attributed to pharmacologically active compounds. However, compound 108f (R = 4-NO_2_) exhibited moderate activity, while compounds 108g (R = 3-NO_2_) and 108h (R = 2-NO_2_) decreased the inhibition activity due to their electron withdrawing groups (Fig. 10).^120^

**Table 14:** Antibacterial activity values of pyrido[2,3-*d*]pyrimidines [minimum inhibitory concentration (MIC) in mg mL^−1^]

Compd	Ar	Gram-positive bacteria			Gram-negative bacteria	
		log *P*	*B. cereus*	*Staphylococcus*	*P. merabitis*	*S. maresens*
108a	4-Me	1.54	12	11	14	13
108b	H	1.10	11	12	12	14
108c	4-OMe	1.15	12	11	13	17
108d	4-OH	0.62	18	15	16	17
108e	4-Cl	1.77	13	10	10	1
108f	4-NO_2_	1.05	11	9	8	10
108g	3-NO_2_	1.03	9	7	11	9
108h	2-NO_2_	1.01	7	6	10	7
SD1:standard drug (streptomycin)		—	21	23	22	22

**Scheme 29:**
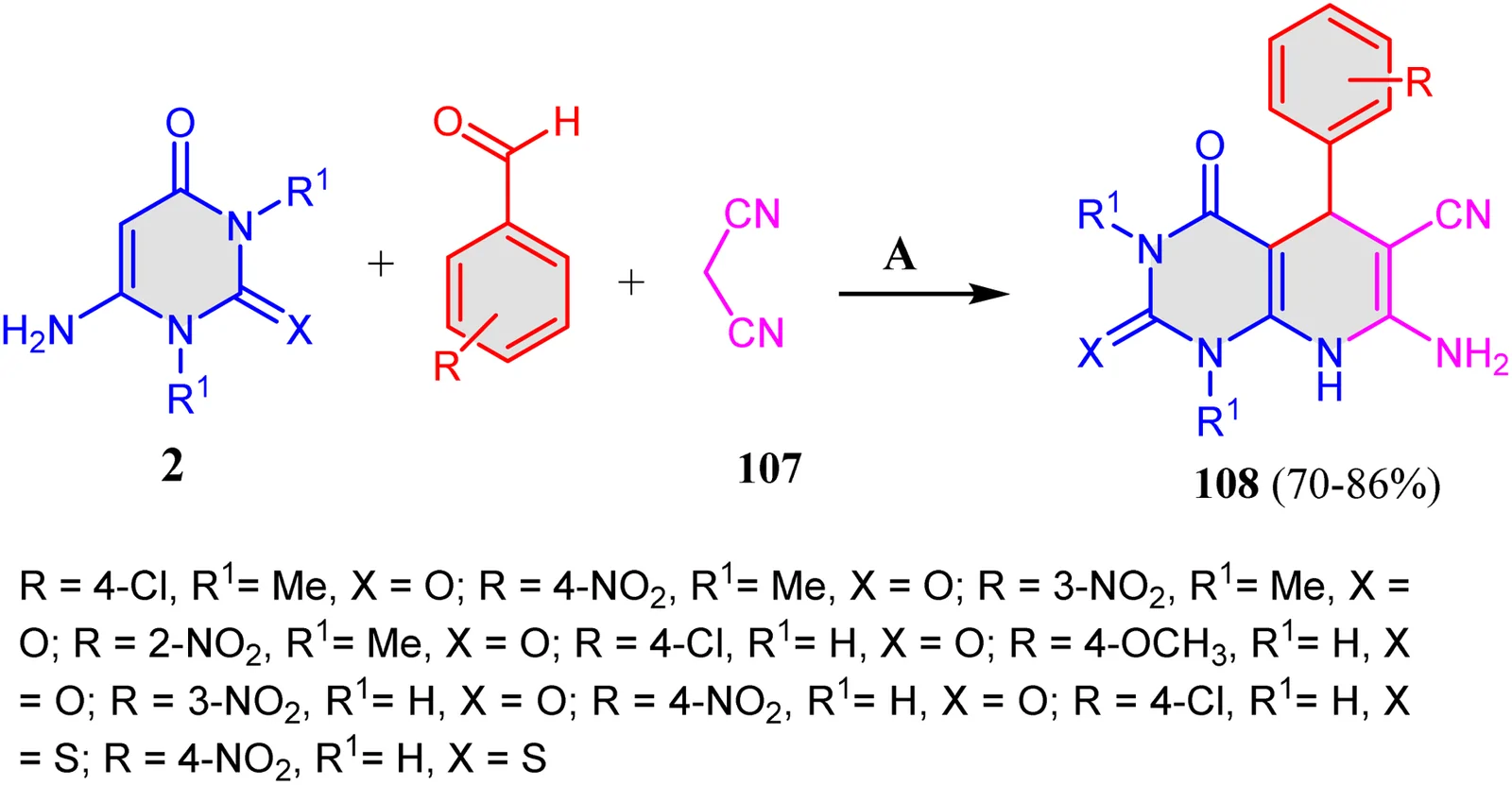
Three-component reaction for the synthesis of 108. Reagents and conditions: A; urea, EtOH:H_2_O, 60 °C.

Meanwhile, Javahershenas and Khalafy^121^ developed a three-component reaction between various 6-aminouracils (2), malononitrile (107), and aromatic aldehydes, which afforded the final products of pyrido[2,3-*d*]pyrimidine-6-carbonitrile derivatives 108 in 70–86% yields. The reaction was performed in EtOH:H_2_O at 60 °C with urea as an organo-catalyst (Scheme 29).^121^

**Scheme 30:**
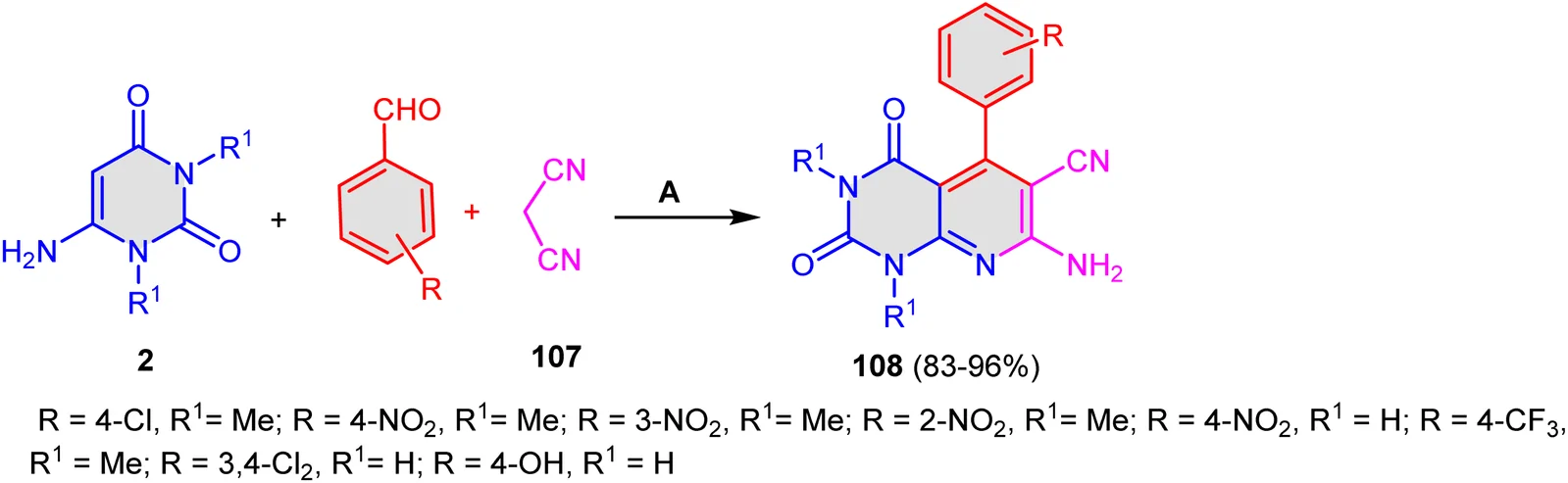
Green synthesis of pyridopyrimidine 108. Reagents and conditions: A; glycerol: H_2_O, 95 °C.

With regard to the methods for the synthesis of pyridopyrimidine frameworks, Anbhule *et al.*,^122^ utilized green solvents, specifically a glycerol–water system, in the synthesis of pyrido[2,3-*d*]pyrimidine derivatives 108 in high yields (83–96%) through a multicomponent reaction between 6-aminouracil 2, various aldehydes, and malononitrile (107) (Scheme 30).^122^

**Scheme 31:**
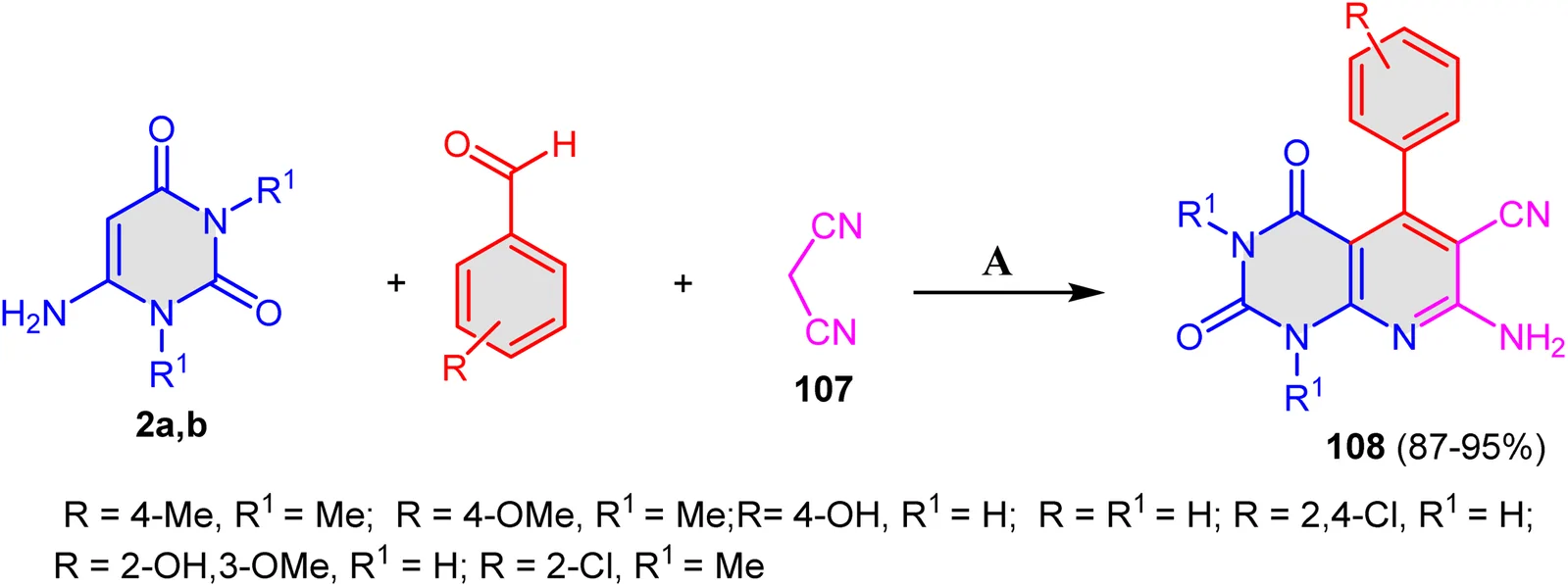
Mn nano catalyst-mediated synthesis of pyrido[2,3-*d*]pyrimidines 108. Reagents and conditions: A; Mn-Zif-8@ZnTiO_3_ NPs, B; EtOH:H_2_O.

In 2020, Hashemian *et al.*,^123^ also prepared a diversity of pyrido[2,3-*d*]pyrimidines skeletons using Mn-ZIF-8@ZnTiO_3_ nanoparticles as a catalyst (Scheme 31). The three-component reaction between aromatic aldehydes, various substituted 6-aminouracil 2a,b, and malononitrile (107) was performed in a H_2_O/EtOH system at 70 °C, and the final products 108 were formed in good to excellent yields (87–95%).^123^

Table 15 shows the utility of various solvents in the synthesis of 108 (R = 4-Me, R^1^ = Me) as a model example. The mixture solvent of H_2_O and EtOH in a ratio of 1 : 1 was found to give the highest yield and lowest reaction time.^123^

**Table 15:** Effects of different solvents on the reaction yield for the synthesis of 108

Entry	Solvent	Condition	108 (R = 4-Me, R^1^ = Me)	
			Time (min)	Yield^a^ (%)
	Solvent-free	120	100	50
1	CH_3_CN	Reflux	80	44
2	CHCl_3_	Reflux	70	46
3	Acetone	Reflux	80	55
4	MeOH	Reflux	50	70
5	EtOH	Reflux	40	85
7	H_2_O	Reflux	30	90
8	EtOH/H_2_O(1 : 1)	Reflux	15	97

a Isolated yield reaction conditions: benzaldehyde (1 mmol), malononitrile (1 mmol) and pyrimidines, Mn-ZIF-8@ZnTiO_3_, temp. 70 °C.

**Scheme 32:**
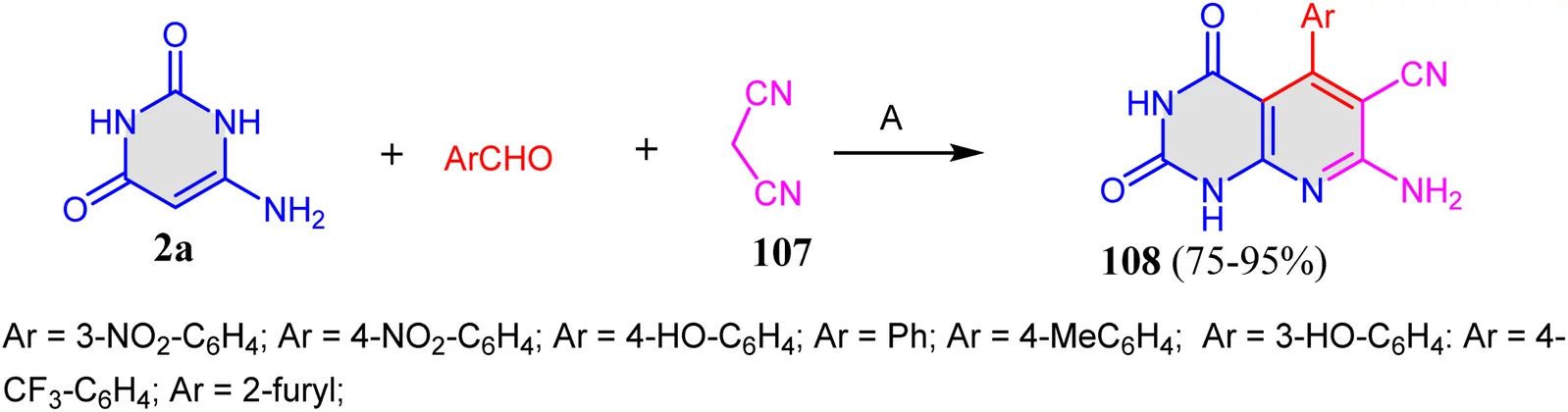
Nano-catalyzed synthesis of tetrahydropyrido[2,3-*d*]pyrimidine-6-carbonitriles 108. Reagents and conditions: A; Fe_3_O_4_-ZnO-NH_2_-PW_12_O_40_. B; H_2_O, 80 °C.

When 6-aminouracil (2a) reacted with aromatic aldehydes and 107 in water at 80 °C and catalyzed by Fe_3_O_4_-ZnO-NH_2_-PW_12_O_40_ nanocatalyst, the reaction proceeded to give 7-amino-pyrido[2,3-*d*]pyrimidine-6-carbonitriles 108 in 75–95% yields (Scheme 32).^124^

**Scheme 33:**
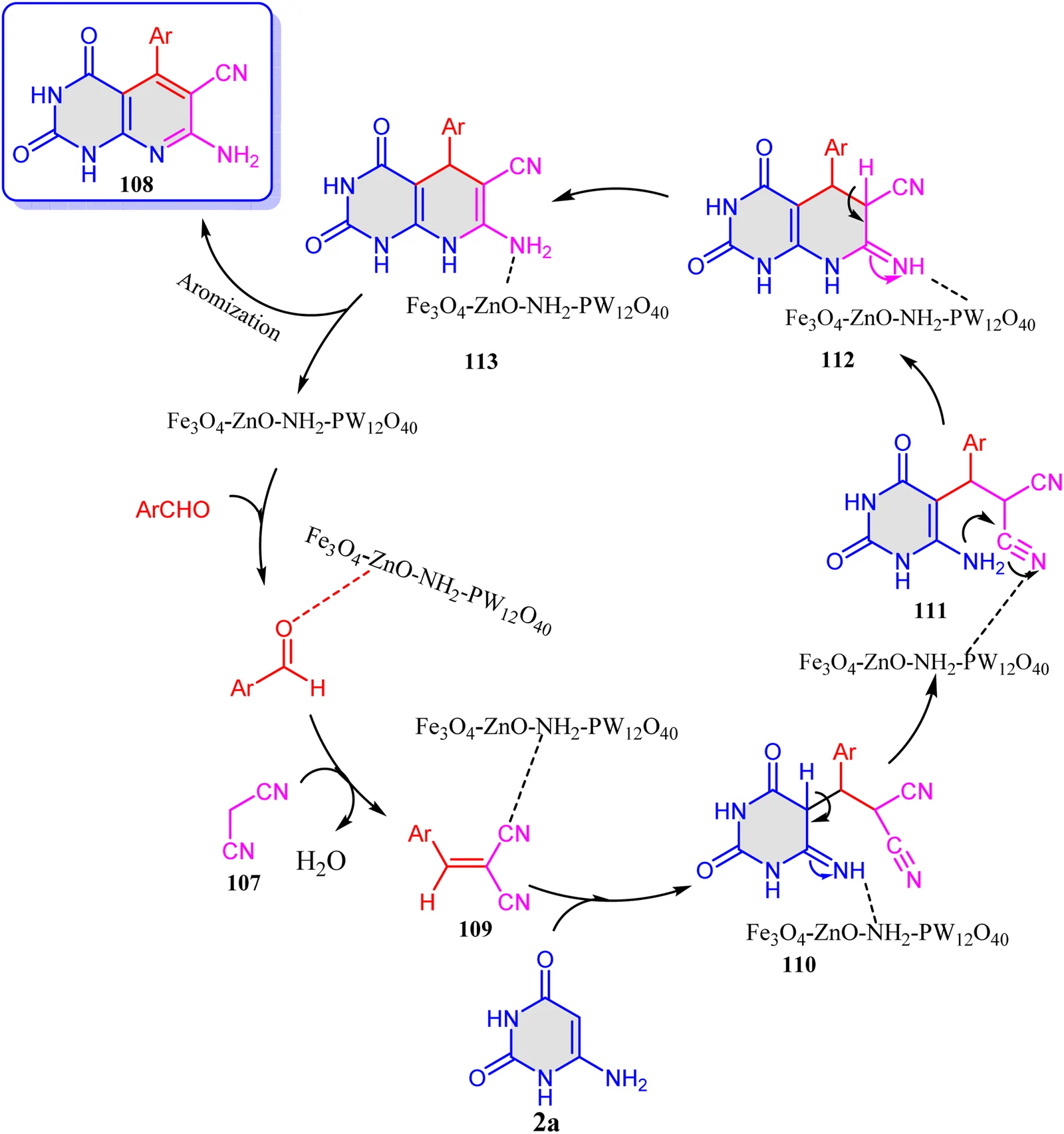
Plausible mechanism for the synthesis of compound 108.

The plausible reaction mechanism is shown in Scheme 33. First, intermediate 109 was formed *via* Knoevenagel condensation between arylaldehyde and malononitrile (107). Second, 6-aminouracil (2a) was added to intermediate 109*via* Michael's addition to generate intermediate 110. After that, intermediate 110 was converted into tautomer 111, which underwent intermolecular cyclization to produce 112. Following this, the tautomerization process was achieved *via* the interconversion of intermediate 112 into 113. Eventually, aromatization of 113 afforded the final products 108 (Scheme 33).^124^

**Scheme 34:**
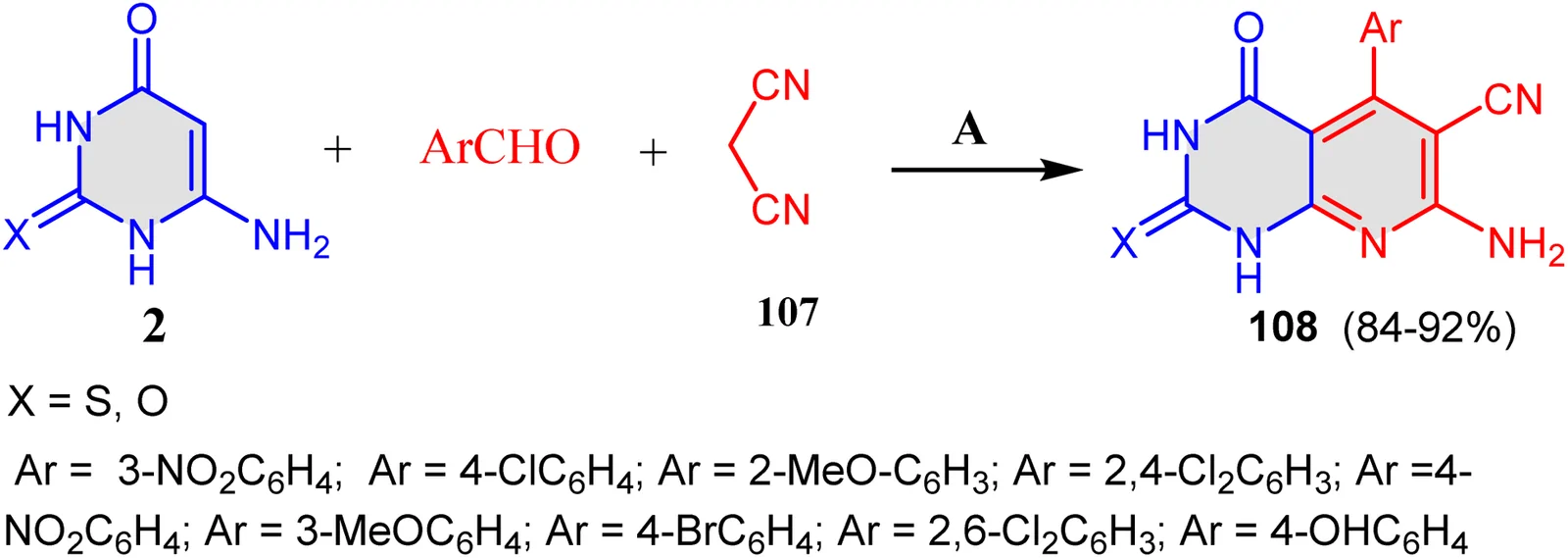
MgO catalytic synthesis of pyrido[2,3-*d*]pyrimidine-6-carbonitriles 108. Reagents and conditions: A = MgO NPs. B = H_2_O, 80 °C.

Moreover, Rad and Mokhtary^125^ used MgO nanoparticles in the synthesis of pyrido[2,3-*d*]pyrimidine derivatives 108 by reacting aminouracil derivatives 2, various aromatic aldehydes and 107. The reaction was performed in H_2_O at 80 °C, and MgO nano-particles (NP) were applied as a catalyst to obtain the final products 108 in good to excellent yields (84–92%) (Scheme 34).

**Scheme 35:**
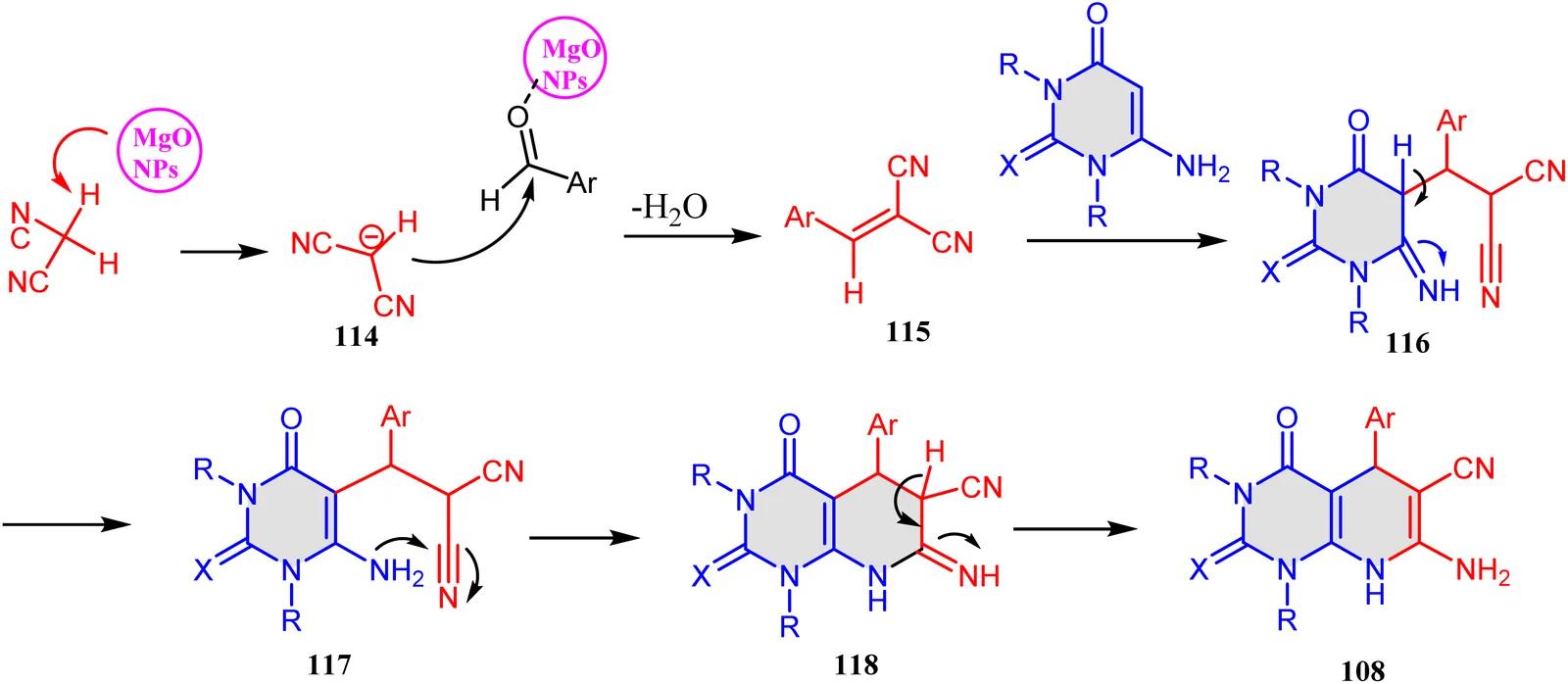
Plausible mechanism for the formation of pyrido[2,3-*d*]pyrimidine-6-carbonitriles 108.

The mechanism for the formation of the final products is shown in Scheme 35. The reaction between the 107 and the aryl aldehydes was promoted by MgO nano particles and afforded intermediate 115, which reacted with 2*via* Michael addition to form intermediate 116. Following that, 116 underwent a rearrangement to give intermediate 117. Next, intermolecular cyclization occurred in 117 to produce 118. Finally, proton transfer and aromatization occurred to intermediate 118 to obtain the final products 108.^125^

**Scheme 36:**
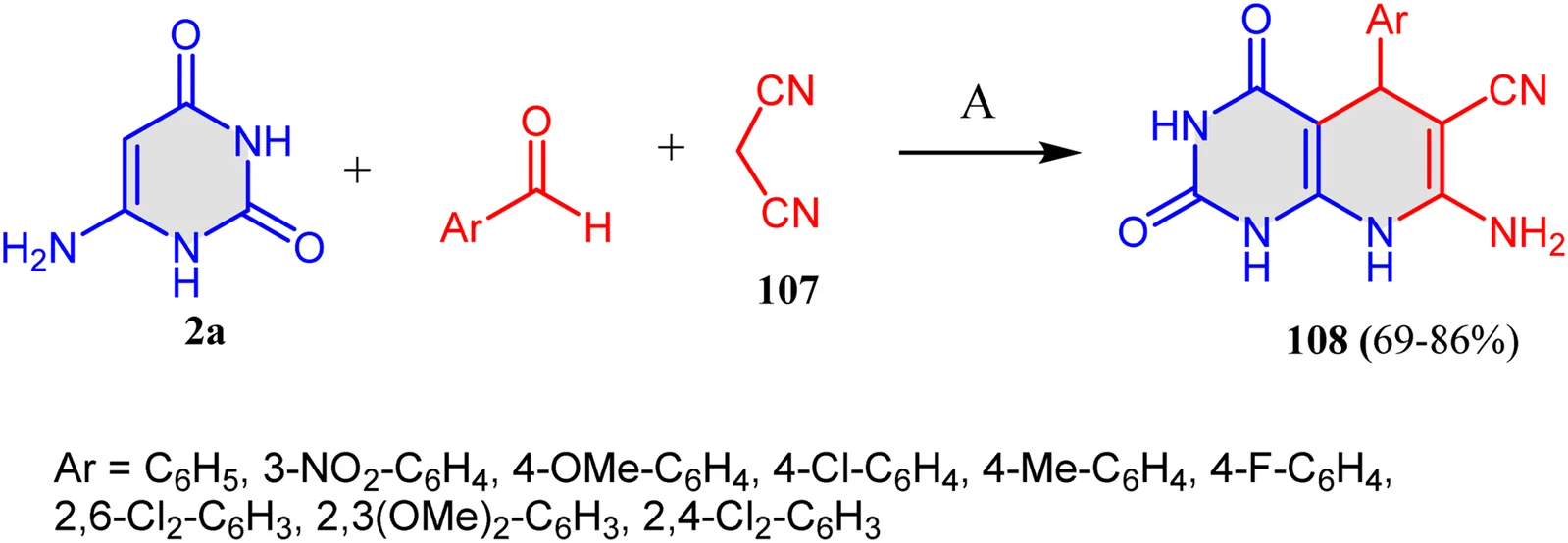
Synthetic pathway for compound 108. Reagents and conditions: A; SBA-Pr-SO_3_H, solvent-free, 60 °C.

Similarly, Ziarani *et al.*^126^ reported an interesting approach for the formation of pyrido[2,3-*d*]pyrimidine derivatives 108 by utilizing SBA-15-Pr-SO_3_H nano-catalyst (NP) under solvent free conditions in the reaction between 6-aminouracil (2a), 107, and aromatic aldehydes to obtain 7-amino-5-aryl-2,4-dioxo-1,2,3,4-tetrahydropyrido[2,3-*d*]pyrimidine-6-carbonitriles 108 in 69–86% yields (Scheme 36).^126^

The antimicrobial activity for synthesized products showed potent activities (Scheme 36) with Ar = C_6_H_5_ (28 and 23 mm), 4-ClC_6_H_4_ (26 and 30 mm), 4-CH_3_C_6_H_4_ (28 and 20 mm), 4-FC_6_H_4_ (30 and 24 mm) and 2,6-Cl_2_C_6_H_3_ (28 and 18 mm) along with inactivity for the compounds with 3-NO_2_C_6_H_4_, 4-OCH_3_C_6_H_4_, and 2,3-(OCH_3_)_2_C_6_H_3_ against *B. subtilis* and *S. aureus* species compared to chloramphenicol and gentamicin standards antibiotics. Thus, the introduction of nitro and methoxy substituents in the phenyl ring is not favored for potent antimicrobial consequences. Additionally, compounds 4-ClC_6_H_4_ (8 and 12 mm) and 4-CH_3_C_6_H_4_ (9 and 10 mm) showed good activities against *E. coli* and *C. albicans* species, respectively. The only recorded activity against the growth inhibition of *P. aeruginosa* was observed for the compound featuring a 4-ClC_6_H_4_ substituted group (10 mm). The compound with an unsubstituted phenyl ring presented the most potent antifungal activity against the *C. albicans* species (14 mm). The MIC values of the different assessments presented potent values for compounds with Ar = C_6_H_5_, 4-CH_3_C_6_H_4_, and 4-FC_6_H_4_ against *B. subtilis* species, along with 4-ClC_6_H_4_ against *S. aureus* species, with MIC values of 2 mg mL^−1^ (Table 16).^126^

**Table 16:** Minimum inhibitory concentrations (lg mL^−1^) of synthesized pyrido[2,3-*d*]pyrimidines against fungi and Gram-positive and Gram-negative bacteria

Ar	*B. subtilis*	*S. aureus*	*E. coli*	*P. aeruginosa*	*C. 2albicans*
Ph	2	8	—	—	128
3-NO_2_C_6_H_4_	—	—	—	—	—
4-OCH_3_C_6_H_4_	—	—	—	—	—
4-ClC_6_H_4_	8	2	512	512	256
4-CH_3_C_6_H_4_	2	32	512	—	512
4-FC_6_H_4_	2	8	—	—	—
2,6-Cl_2_C_6_H_3_	4	64	—	—	—
2,3-(OCH_3_)_2_C_6_H_3_	—	—	—	—	—
Chloramphenicol	4	8	4	256	—
Gentamicin	0.125	0.5	0.5	1	—
Nystatin	—	—	—	—	8

**Scheme 37:**
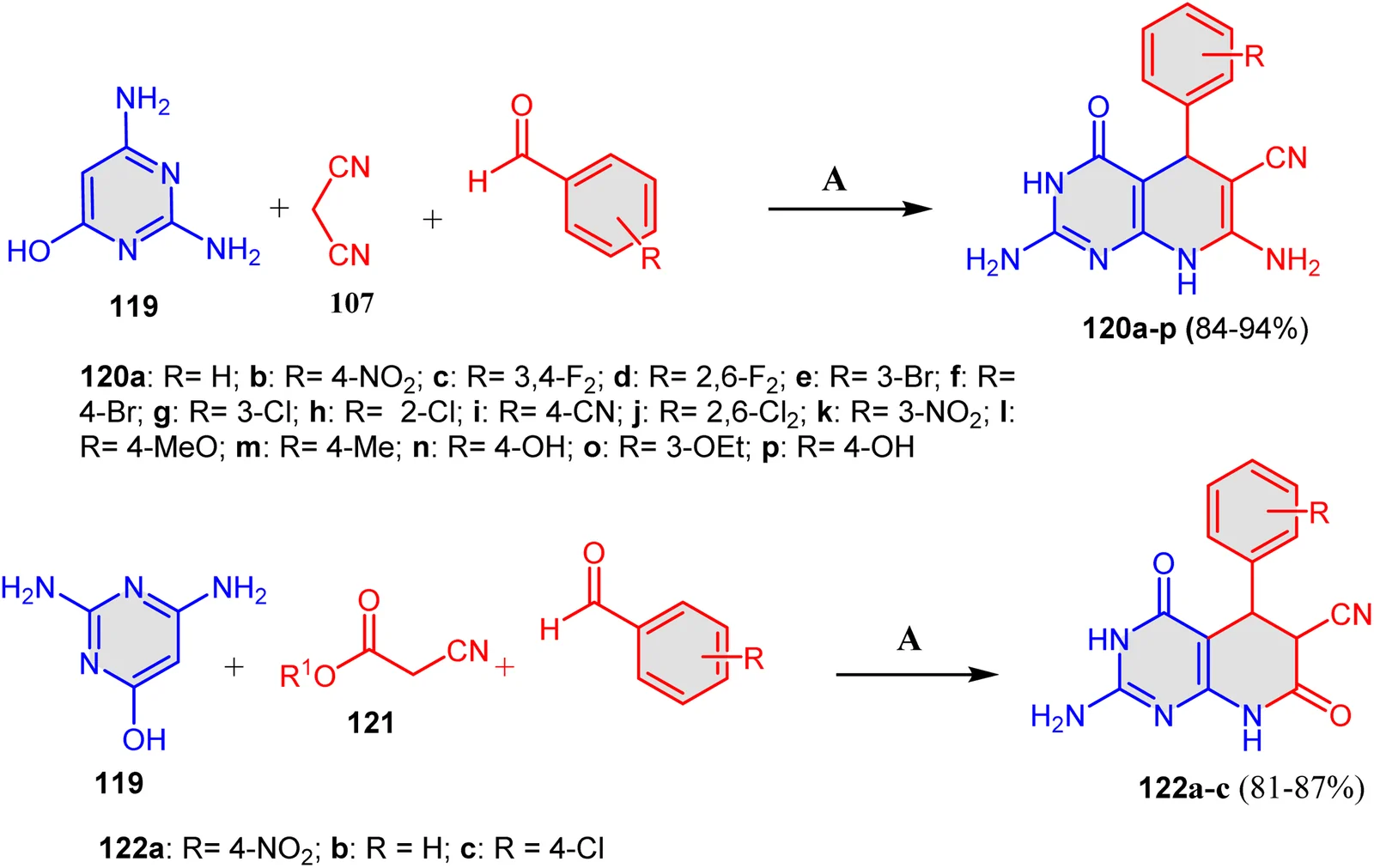
Reaction of 2,6-diaminopyrimidin-4-ol (119) with active methylene compounds. Reagents and conditions: A; Fe_3_O_4_@SiO_2_@(CH_2_)_3_S-SO_3_H, neat, 100 °C.

Furthermore, in 2018, Moradi *et al.*^127^ developed another synthesis of pyrido[2,3-*d*]pyrimidines core 120a–p utilizing Fe_3_O_4_@SiO_2_@(CH_2_)_3_S-SO_3_H nano-magnetic catalyst by reacting 2,6-diamino-pyrimidin-4-ol (119), 107, and various aldehydes under neat conditions at 100 °C to afford 2,7-diamino-5-oxo-4-phenyl-1,8-naphthyridine-3-carbonitriles 120a–p in 84–94% yield (Scheme 37).^127^ Meanwhile, using the same catalyst, a three component reaction of 119 with alkyl 2-cyanoacetates 121 and aldehydes afforded 7-amino-2,5-dioxo-4-phenyl-1,2,3,4,5,6-hexahydro-1,8-naphthyridine-3-carbonitriles 122a–c in 81–87% yields (Scheme 37).^127^

**Scheme 38:**
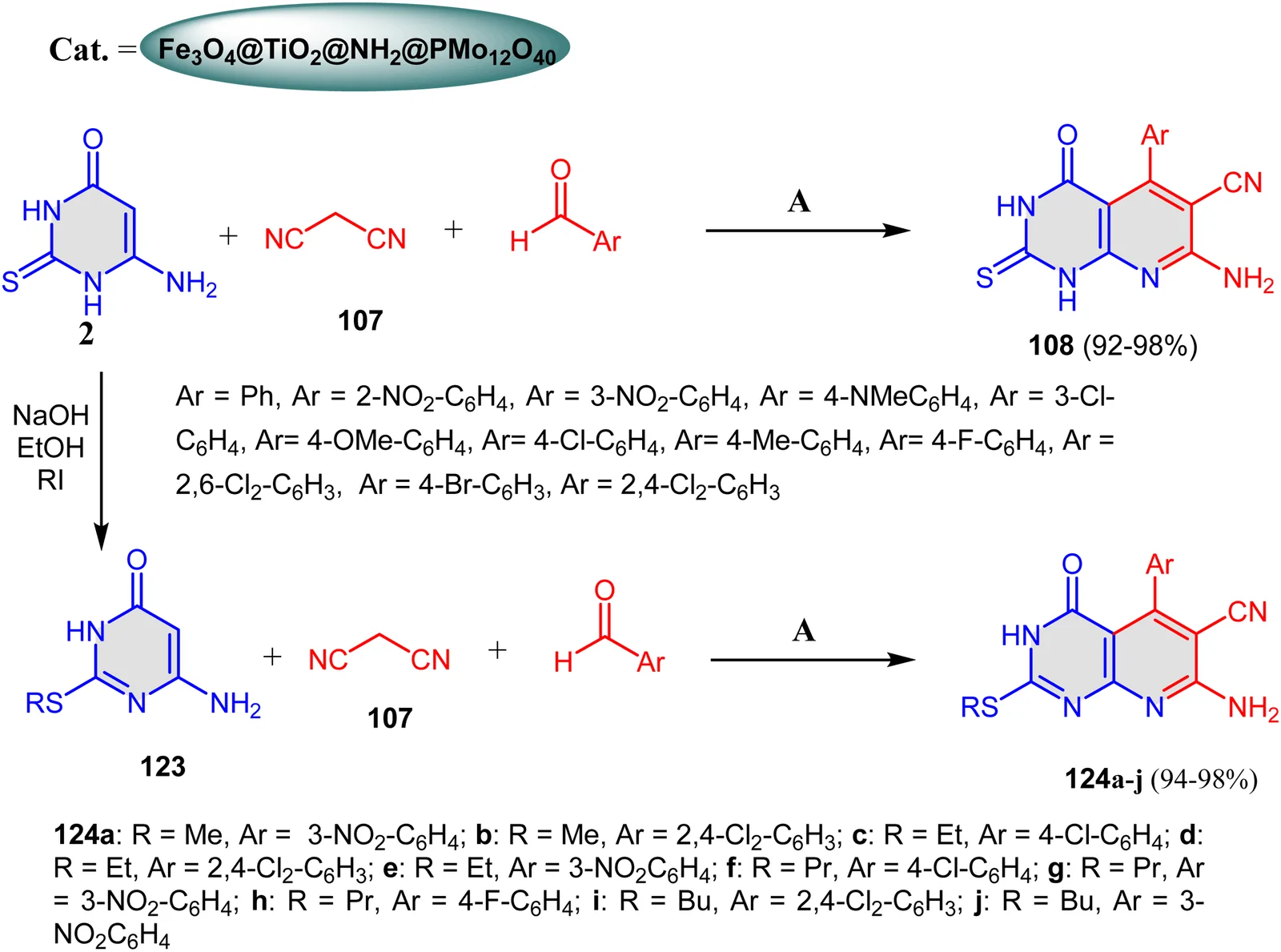
Synthesis of compounds 108 and 124a–j. Reagents and conditions: A; Fe_3_O_4_@TiO_2_@NH_2_@PMo_12_O_40_, H_2_O, 80 °C.

Additionally, Saberikhah *et al.*^128^ developed a simple reaction for synthesizing pyrido[2,3-*d*]pyrimidines 108 through the reaction between aromatic aldehydes 107 and amino-thiouracil 2 to produce the targeted products 108 (Scheme 38). The reaction was achieved under green conditions utilizing Fe_3_O_4_@TiO_2_@NH_2_@PMo_12_O_40_ as a catalyst (Scheme 38). However, the reactions of 6-amino-2-(alkylthio)uracil (123) with aryl aldehydes and 107 under the same conditions afforded 7-amino-2-(alkylthio)-4-oxo-3,4-dihydropyrido[2,3-*d*]pyrimidine-6-carbonitrile derivatives 124a–j (Scheme 38).^128^

**Scheme 39:**
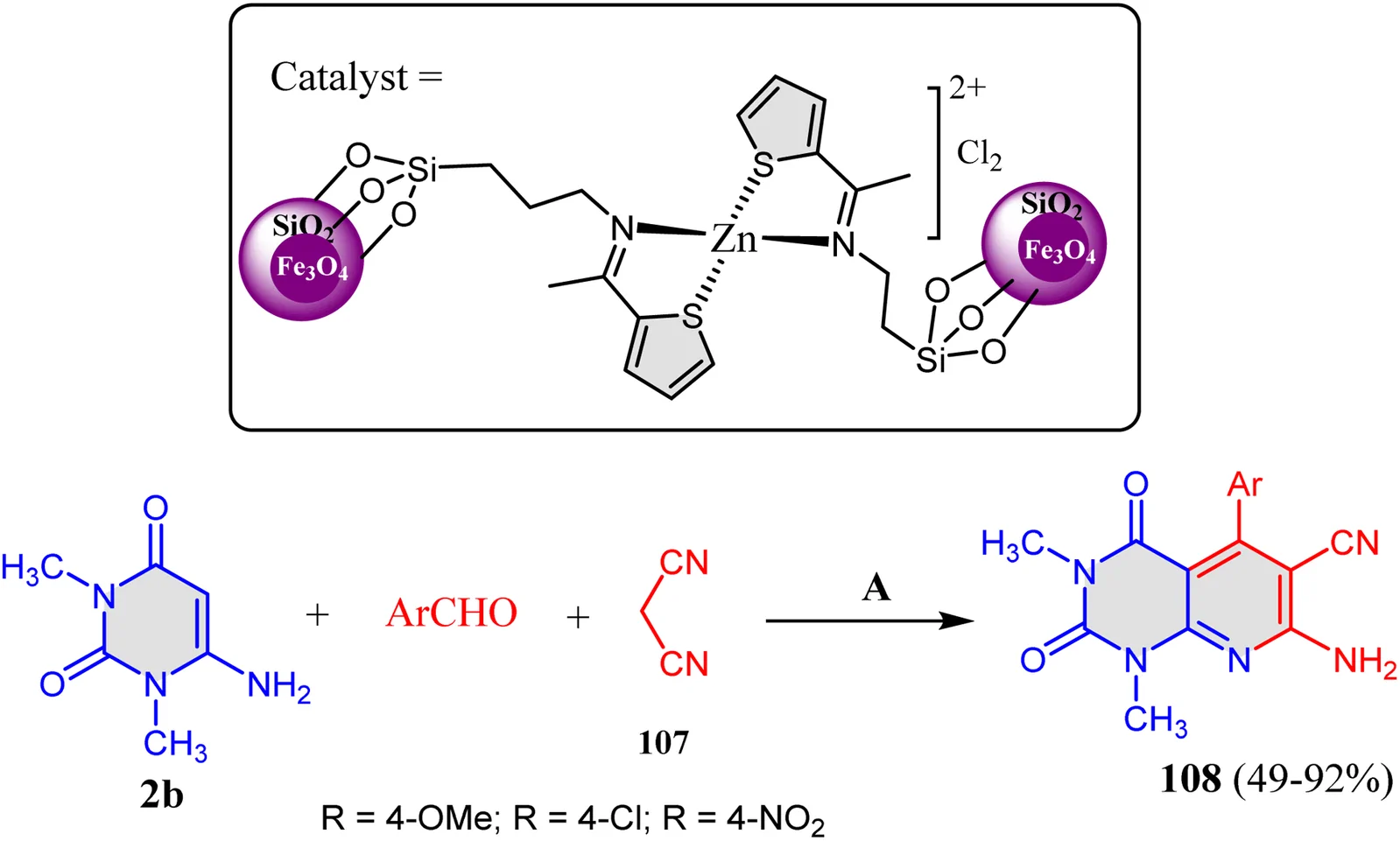
Synthetic pathway for compound 108. Reagents and conditions: A; EtOH, reflux, nano-[Fe_3_O_4_@SiO_2_/*N*-propyl-1-(thiophen-2-yl)ethanimine][ZnCl_2_].

Another series of pyrido[2,3-*d*]pyrimidines 108 was synthesized by Esmaili *et al.*^129^ through a multicomponent reaction between 6-amino-1,3-dimethyluracil (2b), aryl aldehydes and 107 in refluxing EtOH and catalyzed by nano-[Fe_3_O_4_@SiO_2_/*N*-propyl-1-(thiophen-2-yl) ethanimine][ZnCl_2_] as a catalyst (Scheme 39).^129^

**Scheme 40:**
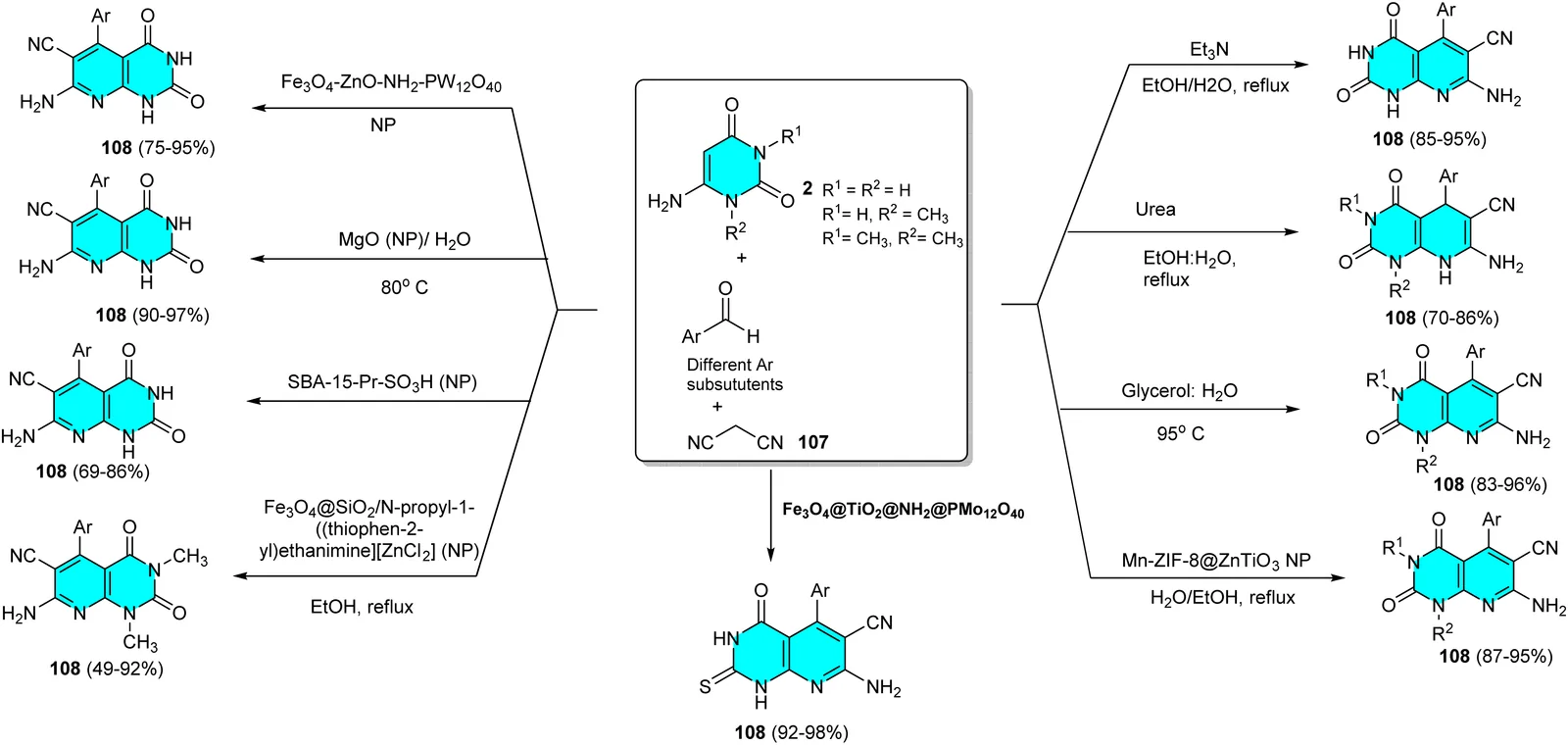
Synthetic pathways for compound 108 under the conditions mentioned in Schemes 28–32, 34, 36, and 39.

In summary, Scheme 40 depicts the conditions listed in Schemes 28–32, 34, 36, and 39 detailed for the synthesis of the derivatives of compound 108 from the reaction of 6-aminouracil derivatives 2, aromatic aldehydes, and 107. The same kind of conversion under various reaction circumstances is depicted in Scheme 40. It is worth mentioning that the most intriguing circumstance resulted from the use of nano-catalysts. The nanocatalysts that achieved the best yields were Fe_3_O_4_@TiO_2_@NH_2_@PMo_12_O_40_ nanocatalyst (92–98%), MgO NPs (90–97%), and Mn-ZIF-8@ZnTiO_3_ (87–95%).

**Scheme 41:**
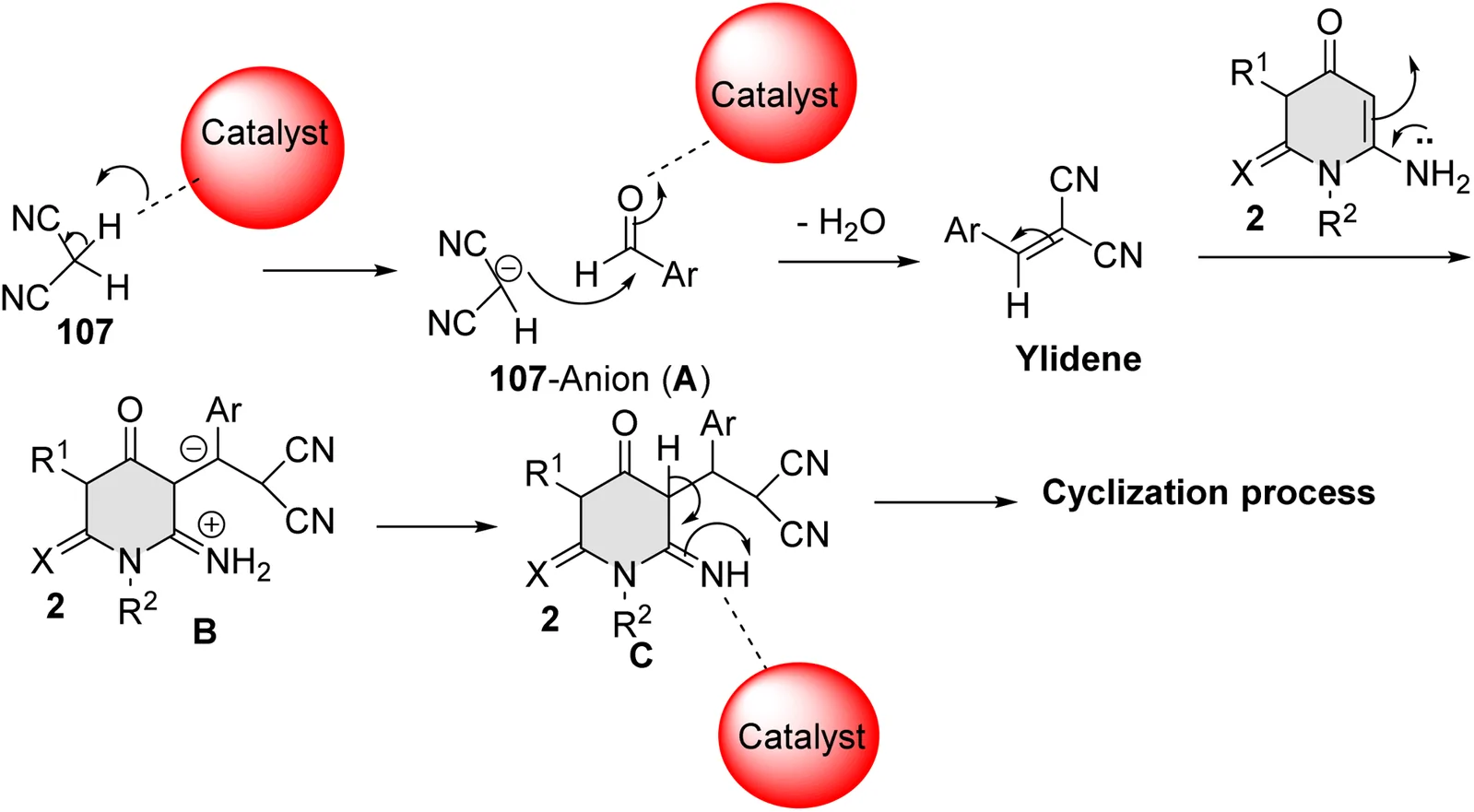
General features of the role of a catalyst in the pathways describing the formation of 108.

In addition, the role of the catalyst is described in the formation of 108 from the reaction of 6-aminouracil derivatives 2 with aryl aldehydes and 107, as it enhances the abstraction of a hydrogen proton from 107 to form its anion (A, Scheme 41). The second role of the catalyst is to enhance the electrophilicity of the carbon of the carbonyl group of the aldehydes. The latter enables the previously formed anion A to attack the carbonyl group to form the corresponding ylidene. Furthermore, nucleophilic attack of the nucleophilic carbon CH-5 of 2 to the ylidene forms intermediate B. The neutralization of B then gives intermediate C. The third role of the catalyst is to enhance the 1,3-H shift so as to facilitate the subsequent sequence and cyclization process, which would then occur (Scheme 41).

**Scheme 42:**
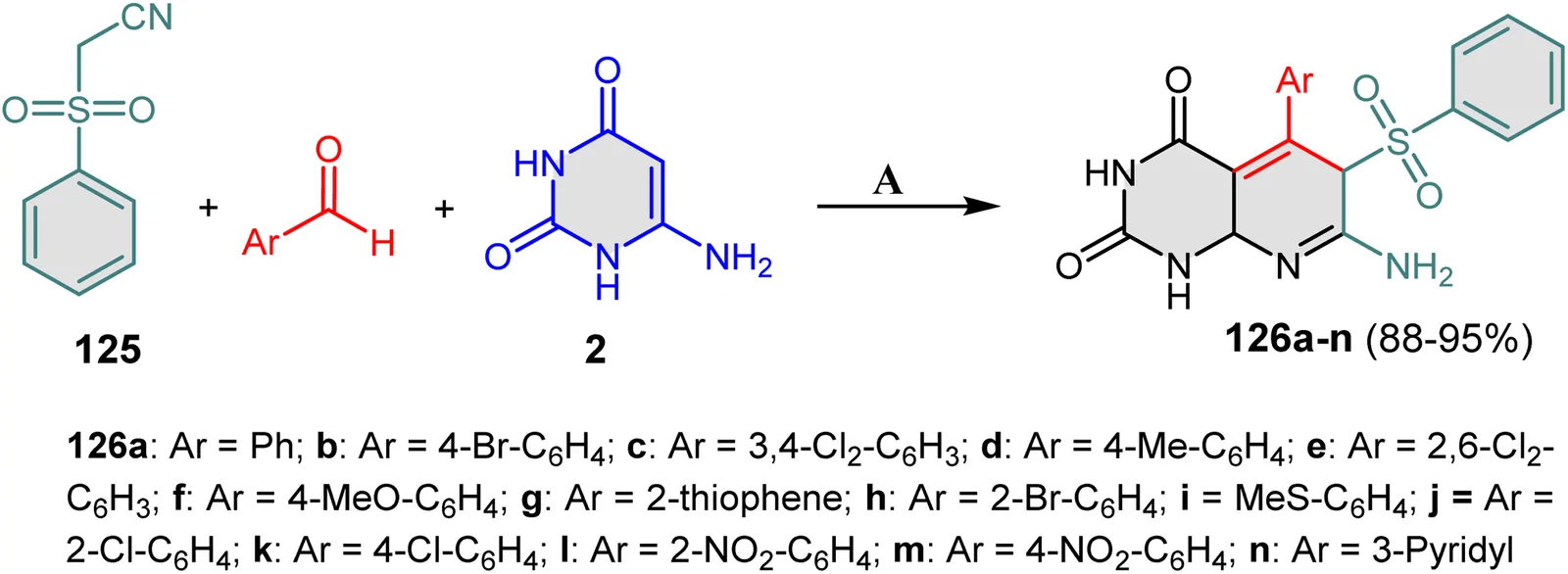
Synthesis of pyrido[2,3-*d*]pyrimidines 126a–n. Reagents and conditions: A = Glycerol, 80 °C.

Additionally, the reaction of (phenylsulfonyl)acetonitrile (125), aromatic aldehydes and 6-aminouracil (2) in glycerol as a green solvent at 80 °C yielded the desired products of 7-amino-5-aryl-6-(phenylsulfonyl)-6,8*a*-dihydropyrido[2,3-*d*]pyrimidine-2,4-diones 126a–n in excellent yields (88–95%), as depicted in Scheme 42.^130^

Table 17 shows that glycerol acts well and produces 7-amino-5-phenyl-6-(phenylsulfonyl)-6,8a-dihydropyrido[2,3-*d*]pyrimidine-2,4(1*H*,3*H*)-dione (126a) at 80 °C for 2 h with 90% yield (entry 10).^129^

**Table 17:** Optimization conditions for the preparation of 126a under various solvents and temperatures

Entry	Solvent	Temperature (°C)	Time (h)	Yield (%)
1	Acetonitrile	80	12	NR
2	DMF	80	12	NR
3	Toluene	80	12	NR
4	DMSO	80	12	NR
5	EtOH	80	12	NR
6	H_2_O	80	12	NR
7	Neat	80	12	NR
8	Glycerol	RT	7	72
9	Glycerol	60	3	85
10	Glycerol	80	2	90
11	Glycerol	100	2	90

**Scheme 43:**
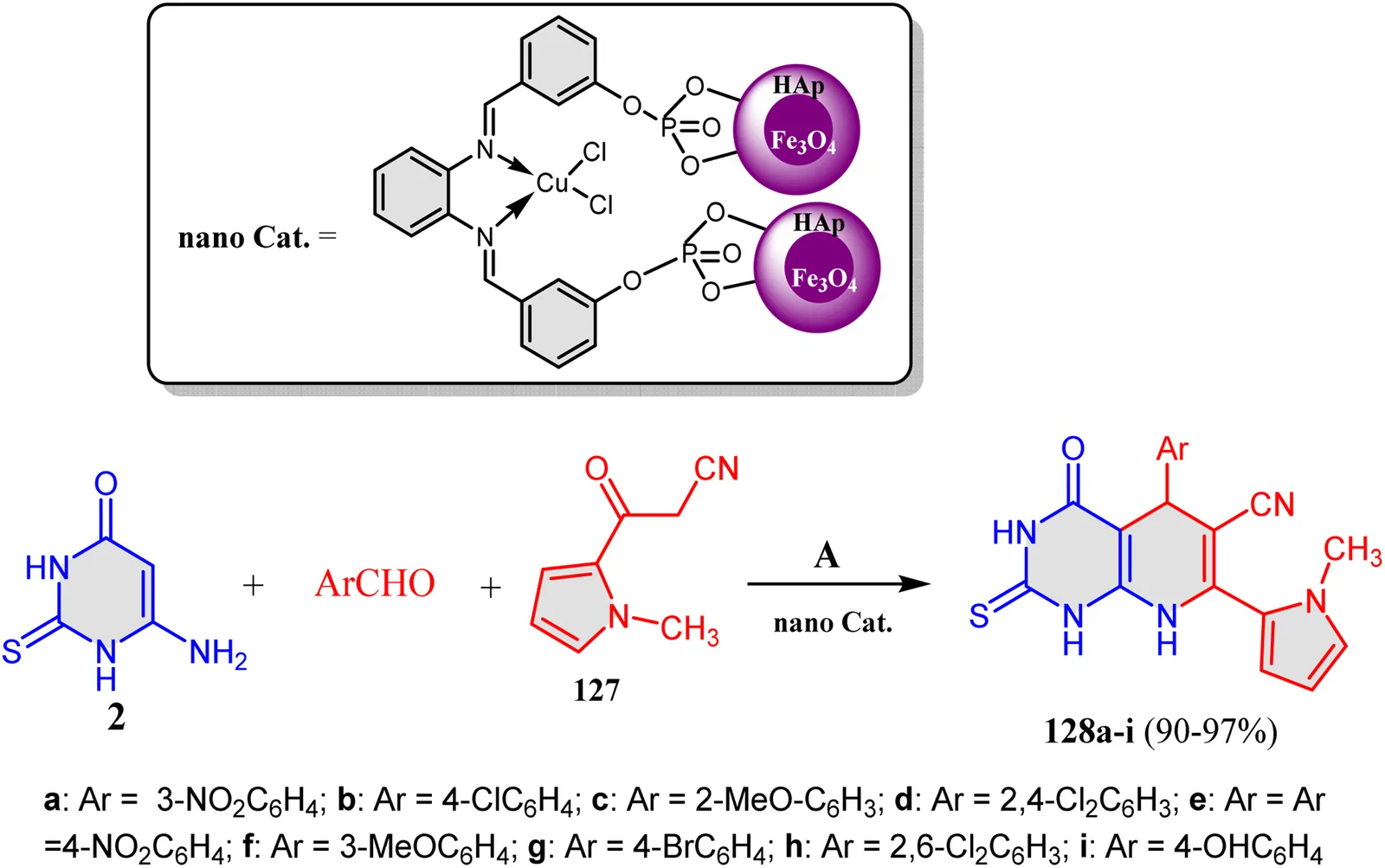
Synthesis of pyrido[2,3-*d*]pyrimidines 128a–n. Reagents and conditions: A; EtOH, 80 °C, 8–13 min.

In the same manner, Saberikhah *et al.*^131^ reported the synthesis of 1-methyl-1*H*-pyrrolyl-hexahydropyrido[2,3-*d*]pyrimidine scaffolds 128a–i through multicomponent reaction between 6-amino-thiouracil, 1-methyl-2-cyanoacetyl-1*H*-pyrrole (127), and aryl aldehydes in EtOH and catalyzed by γ-Fe_2_O_3_@HAp@PBABMD@Cu magnetic nanoparticles to give the targeted products 128a–i in 90–97% yields (Scheme 43).^131^ Due to the multi-step process of the nano-catalyst preparation, the catalyst's remarkable productivity and recyclability gave this procedure additional significance. Incorporating the pyrrole ring into bicyclic pyridopyrimidines is advantageous for the potential activity of biological potency.^130^

**Scheme 44:**
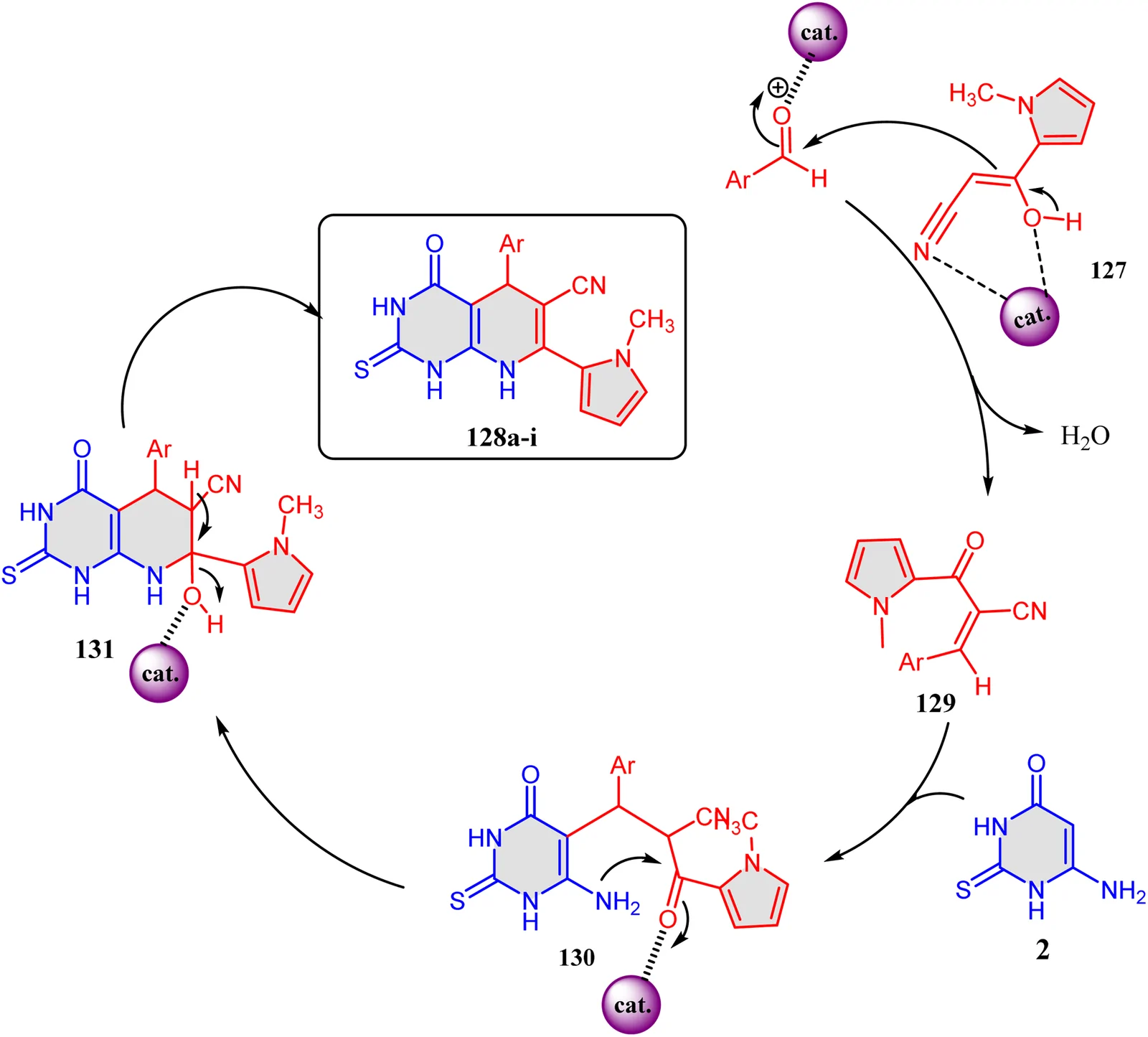
Suggested mechanism for the synthesis of compound 128.

The proposed mechanism of the synthesis of pyrrolyl-hexahydropyridopyrimidines 128a–i is shown in Scheme 44. Initially, the carbonyl group of aldehydes was activated by the nano-catalyst during Knoevenagel condensation between aldehydes and compound 127 to give arylidene 129. Subsequently, intermediate 130 was formed *via* the Michael addition reaction between 129 and 2. Finally, intermediate 130 went through intramolecular cyclization to produce intermediate 131, which lost a molecule of water to produce the desired products 128a–i (Scheme 44).^131^

**Scheme 45:**
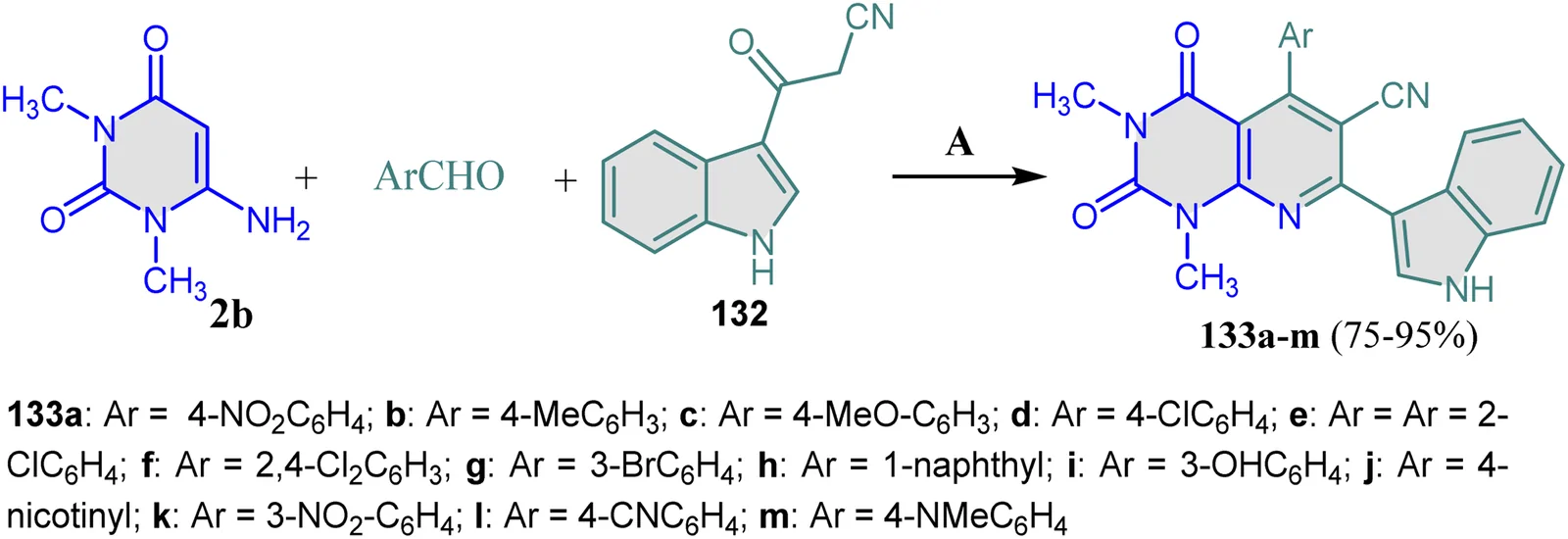
Synthesis of pyridopyrimidine-indole hybrids 133a–m. Reagents and conditions: A; EtOH, Fe_3_O_4_@FAp@Ni.

**Scheme 46:**
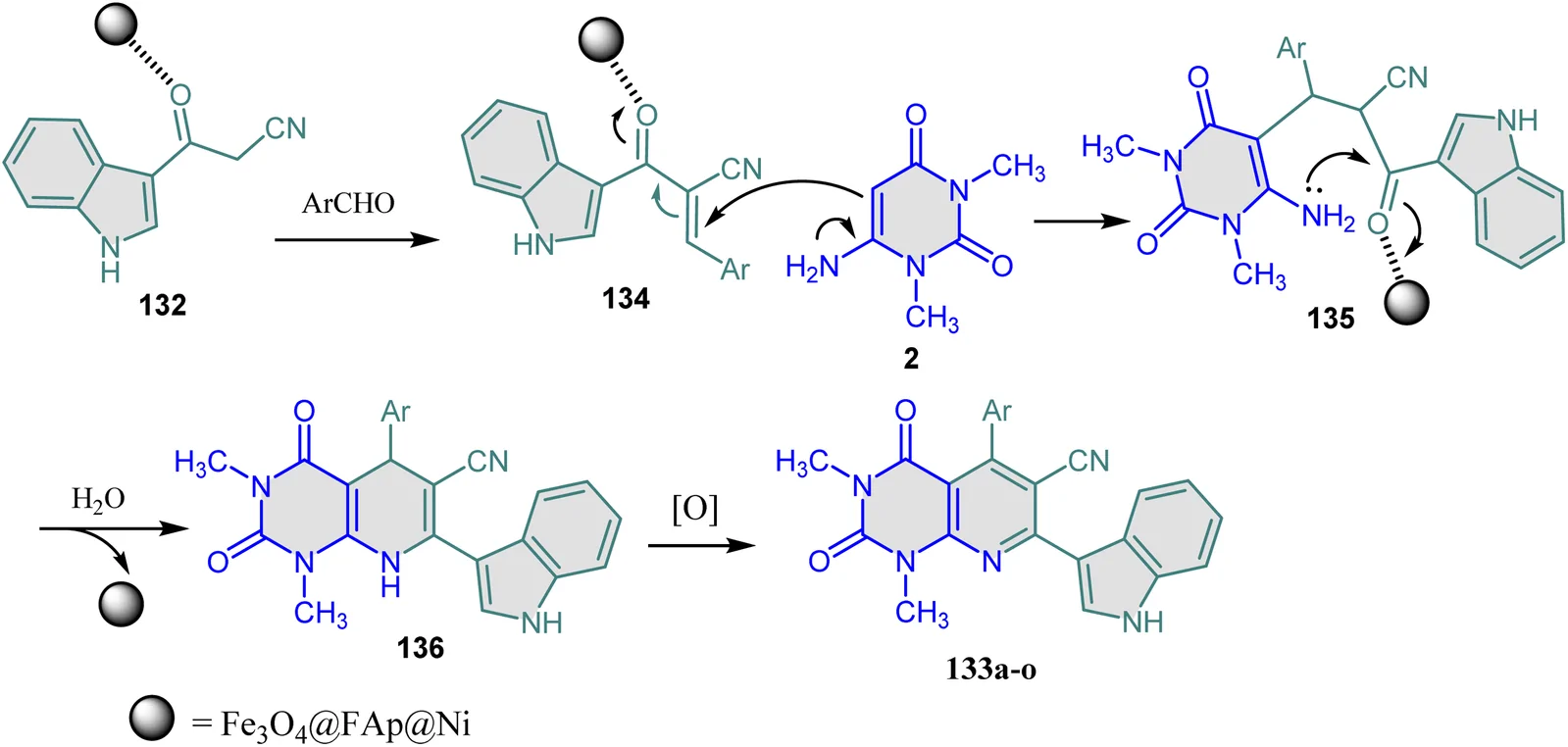
Possible mechanistic pathway for the formation of 133a–m.

In 2020, Dastmard *et al.*^132^ reported green efficient synthesis of pyridopyrimidine-indole hybrid molecules 133a–m*via* multicomponent reaction between 6-amino-1,3-dimethyl-pyrimidine-2,4-dione (2b) with 3-cyanoacetyl-indole (132) and aromatic aldehydes in EtOH, catalyzed by Fe_3_O_4_@FAp@Ni nano catalyst (Scheme 45). The possible mechanistic pathway for the formation of 133a–m is shown in Scheme 46. The presence of Fe_3_O_4_@FAp@Ni nano catalyst activated the reaction between compound 132 and aldehydes to form intermediate 134. Next, a Michael addition between 134 and 2b occurred and afforded 135. Subsequently, the intermolecular cyclization and elimination of a molecule of water afforded adduct 136. Following that, the oxidation and aromatization of 136 occurred to obtain pyridopyrimidine compounds 133a–m (Scheme 46).^132^

**Scheme 47:**
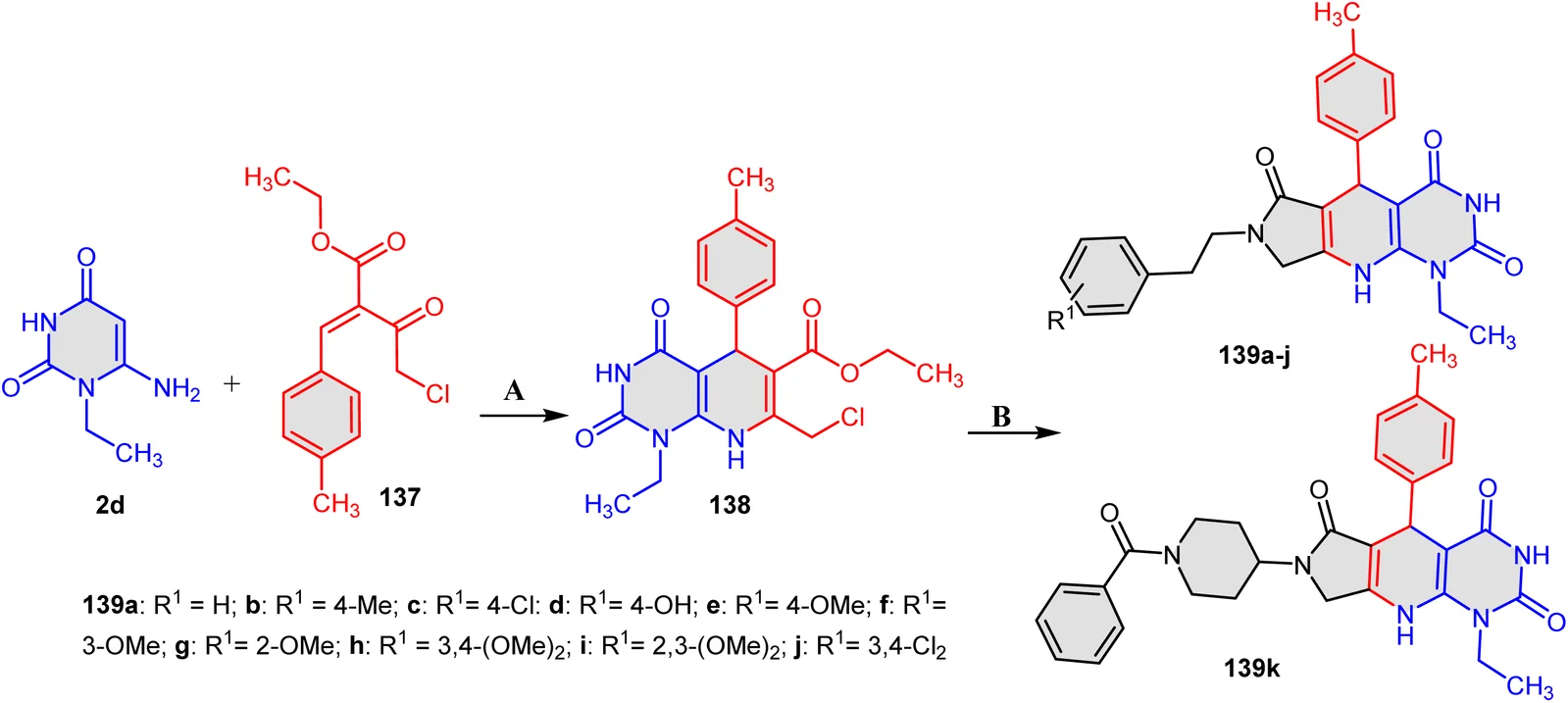
Formation of pyrrolo[3′,4′:5,6]pyrido[2,3-*d*]pyrimidine-2,4,6(3*H*)-triones 139a–k. Reagents and conditions: A; MgSO_4_, MeOH, 45 °C. B; RNH_2_, EtOH, Microwave, 120 °C.

A diversity of pyrrolo[3′,4′:5,6]pyrido[2,3-*d*]pyrimidine-2,4,6(3*H*)-trione compounds 139a–j were successfully synthesized by Jiang and coworkers.^28^ The reaction occurred between 6-amino-1-ethyluracil 2d and ethyl-4-chloro-2-(4-methylbenzylidene)-3-oxobutanoate (137) in MeOH at 45 °C in the presence of MgSO_4_ to produce ethyl-7-(chloromethyl)-1-ethyl-2,4-dioxo-5-(*p*-tolyl)-1,2,3,4,5,8-hexahydro-pyrido[2,3-*d*]pyrimidine-6-carboxylate (138), which then reacted with primary amines in EtOH at MW irradiation to produce the final products 139a–k in 10–45% yields, as depicted in Scheme 47.^28^ Structural changes in the dihydropyridine lactam side chain of compound 139 were investigated to study its impact on both BET affinity (the BET affinity constant describes the material's adsorption characteristics) and selectivity. All derivatives demonstrated a slight preference for BRD4-1 over BRDT-1 (Table 18), including 139k and 139f. According to the results, compound 139f was highly selective for the bromodomain and extra terminal (BET) bromodomains.^28^

**Table 18:** Structure and inhibitory profile of second-round modifications

Compd		R^1^	IC_50_ [μM] BRDT-1	IC_50_ [μM] BRDT-2	IC_50_ [μM] BRD4-1	IC_50_ [μM] BRD4-2
139a	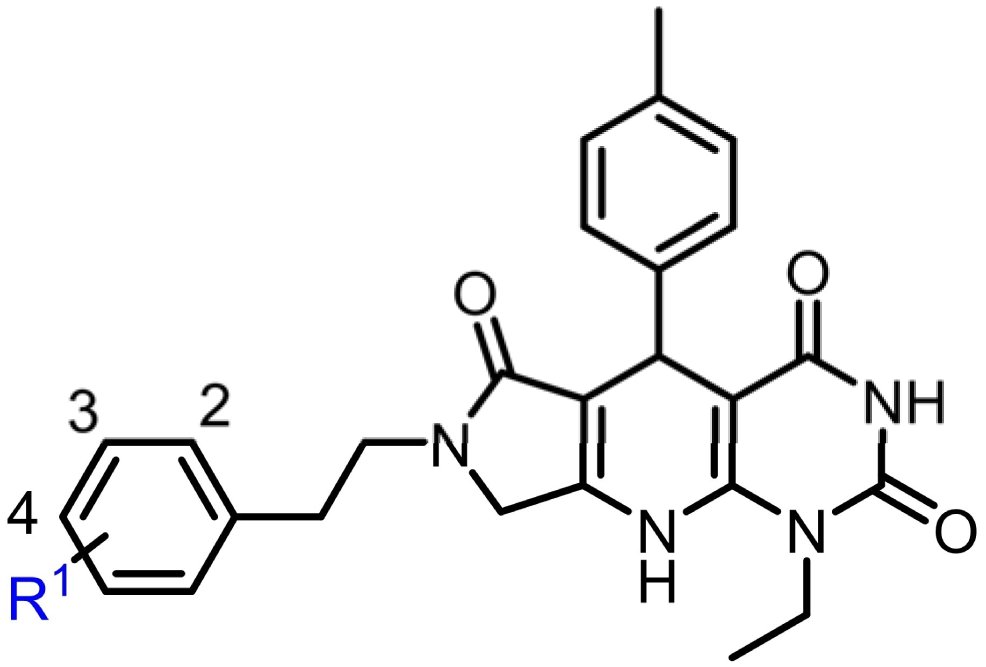	H	7.6 ± 1.7	1.2 ± 0.08	2.3 ± 0.48	1.4 ± 0.12
139b		4-Me	12 ± 1.9	2.2 ± 0.07	3.5 ± 0.93	2.1 ± 0.16
139c		4-Cl	25 ± 4.1	3.1 ± 0.24	6.3 ± 2.1	3.0 ± 0.49
139d		4-OH	2.2 ± 0.76	0.61 ± 0.06	1.3 ± 0.20	0.82 ± 0.03
139e		4-OMe	4.3 ± 0.26	104 ± 13	0.60 ± 0.15	0.98 ± 0.04
139f		3-OMe	18 ± 3.6	1.2 ± 0.08	1.9 ± 0.42	1.3 ± 0.09
139g		2-OMe	9.4 ± 1.1	1.5 ± 0.07	2.3 ± 0.40	1.6 ± 0.08
139h		3,4-(OMe)_2_	103 ± 12	14 ± 2	23 ± 0.79	17 ± 2.84
139i		2,3-(OMe)_2_	69 ± 8.7	13 ± 2.8	20 ± 1.2	11 ± 3.5
139j		3,4-Cl_2_	35 ± 3.64	8.4 ± 1.7	13 ± 3.8	6.0 ± 1.5
(+)-JQ1^a^		—	1.0 ± 0.1	1.1 ± 0.8	0.37 ± 0.1	0.30 ± 0.01

a (+)-JQ1 was used as a positive control in the AlphaScreen assay. All compounds were tested in quadruplicate; ± indicates standard deviation.

##### 2.2.5.3 Structure activity relationship

**Fig. 11:**
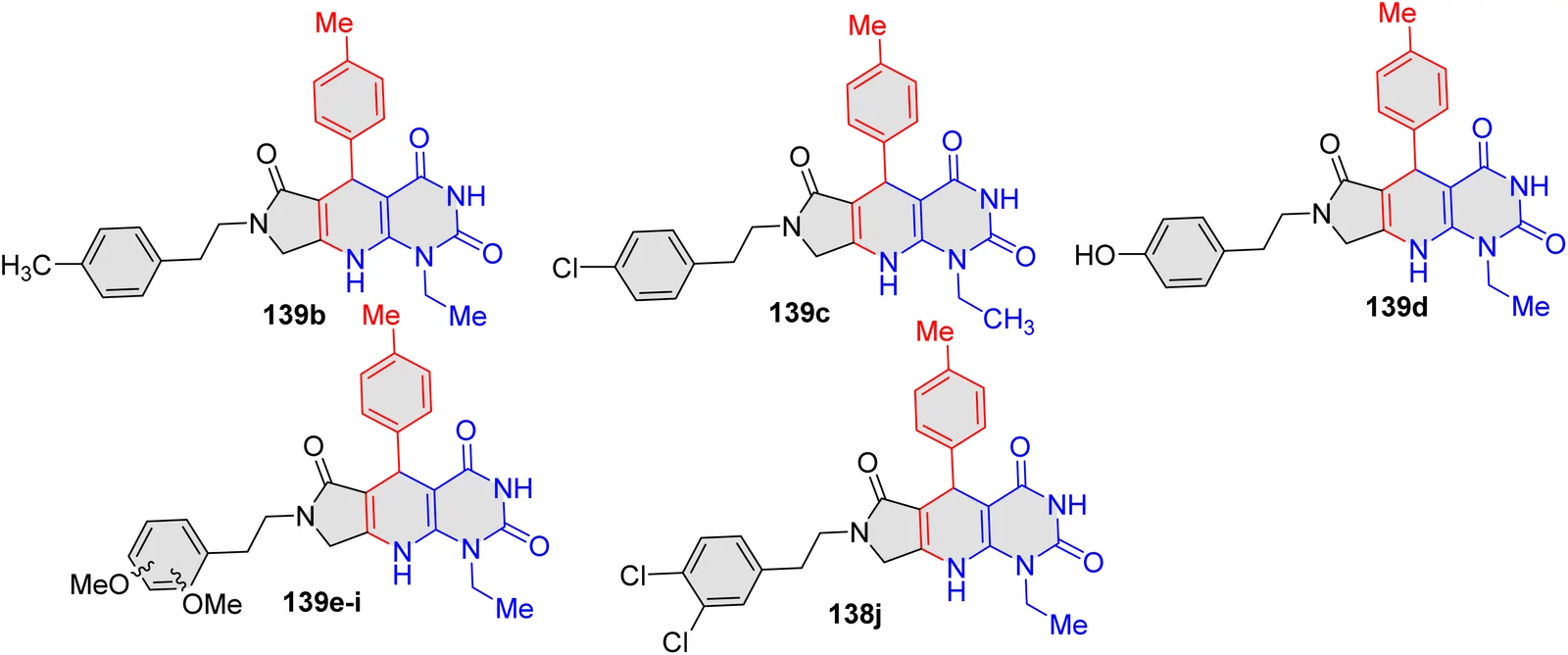
Structure of compounds 139b–h with inhibition activity against BRDT-1.

Table 18 shows that an electron-rich aromatic ring may interact with special R54 in BRDT-1 through a cation-pi process. Overall, aromatic substitution had no discernible impact on affinity. Fig. 11 shows that the structures of compounds 139b (R = Me), 139d (R = 4-OH), and 139e (R = 4-OMe), containing electron-donating groups, were slightly more potent than that of compound 139c (R = 4-Cl) with an electron withdrawing group. Compounds 139e, 139f, and 139g containing OMe groups were investigated, and compound 139e (R = 4-OMe) had the highest inhibition activity, followed by 2-OMe and 3-OMe substitutions. BET activity was not improved by disubstitution with OMe or chlorine moieties, as shown in (139h, 139i, and 139j). However, 139h (R = 3,4-(OMe)_2_) revealed the least inhibition activity against BRDT-1. None of the aryl changes significantly affected BRDT-1 specificity. Furthermore, this subset of analogs showed similar activities for BRD4-1 and BRD4-2.^28^

**Scheme 48:**
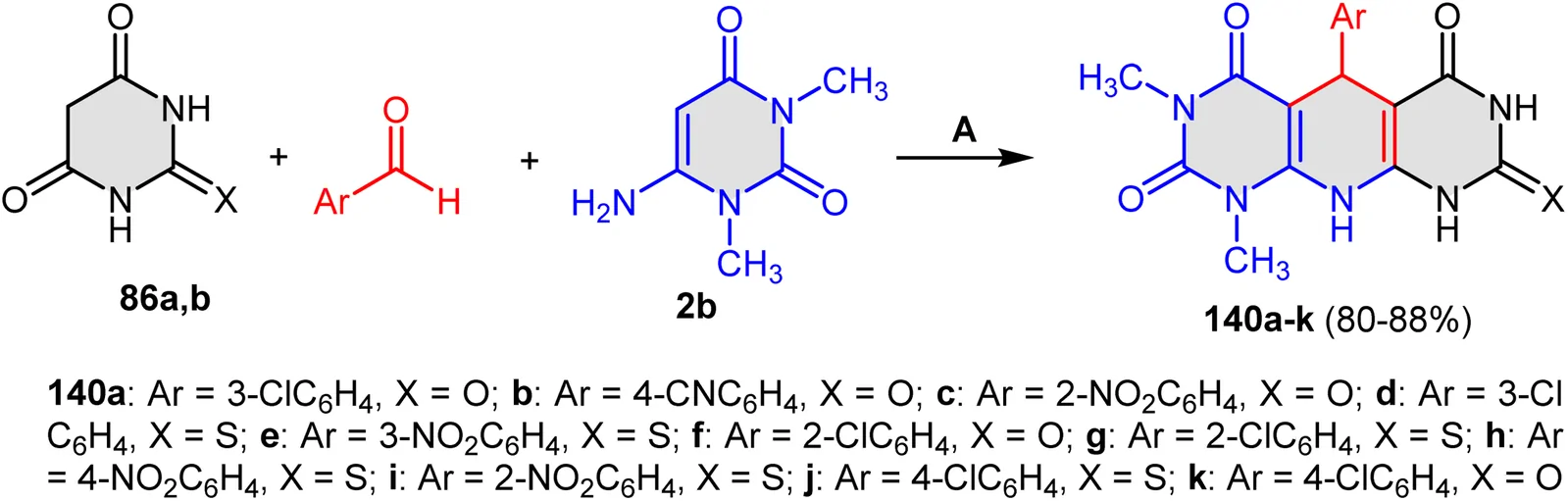
P_2_O_5_-mediated synthesis of 140a–k. Reagents and conditions: A = P_2_O_5_/EtOH.

Patil *et al.*^133^ also synthesized another series of substituted pyrido[2,3-*d*:6,5-*d*]dipyrimidines 140a–k in good yields (80–88%) through the reaction between thiobarbituric acid or barbituric acid 86a,b, aromatic aldehydes, and 1,3-dimethyl-6-aminouracil (2b) (Scheme 48). The reaction was catalyzed by phosphorous pentoxide (P_2_O_5_) and carried out in EtOH as a solvent.

All synthetic products were examined for their antituberculosis activity. The majority of the products demonstrated moderate to good activity against the *M. tuberculosis* H37RV strain according to the results (Table 19). Using standard drugs, such as pyrazinamide, ciprofloxacin, and streptomycin, for comparison purposes, the synthesized compounds were screened for their antimycobacterial activity at concentrations of 0.8, 1.6, 3.12, 6.25, 12.5, 25, 50, and 100 μg mL^−1^. The results showed that all the compounds demonstrated activity at 50 μg mL^−1^ (Table 19).^126^

**Table 19:** Antituberculosis activity of compounds 140a–k against *Mycobacterium tuberculosis*^a^

Compd	100 μg mL^−1^	50 μg mL^−1^	25 μg mL^−1^	12.5 μg mL^−1^	6.25 μg mL^−1^	3.12 μg mL^−1^	1.6 μg mL^−1^	0.8 μg mL^−1^
140a	S	S	R	R	R	R	R	R
140b	S	S	R	R	R	R	R	R
140c	S	S	R	R	R	R	R	R
140d	S	S	R	R	R	R	R	R
140e	S	S	R	R	R	R	R	R
140f	S	S	R	R	R	R	R	R
140g	S	S	R	R	R	R	R	R
140h	S	S	R	R	R	R	R	R
140i	S	S	R	R	R	R	R	R
140j	S	S	R	R	R	R	R	R
140k	S	S	R	R	R	R	R	R
Minimum inhibitory concentration	Pyrazinamide				3.125 μg mL^−1^			
	Streptomycin				6.25 μg mL^−1^			
	Ciprofloxacin				3.125 μg mL^−1^			

a S, susceptible; R; resistant.

**Scheme 49:**
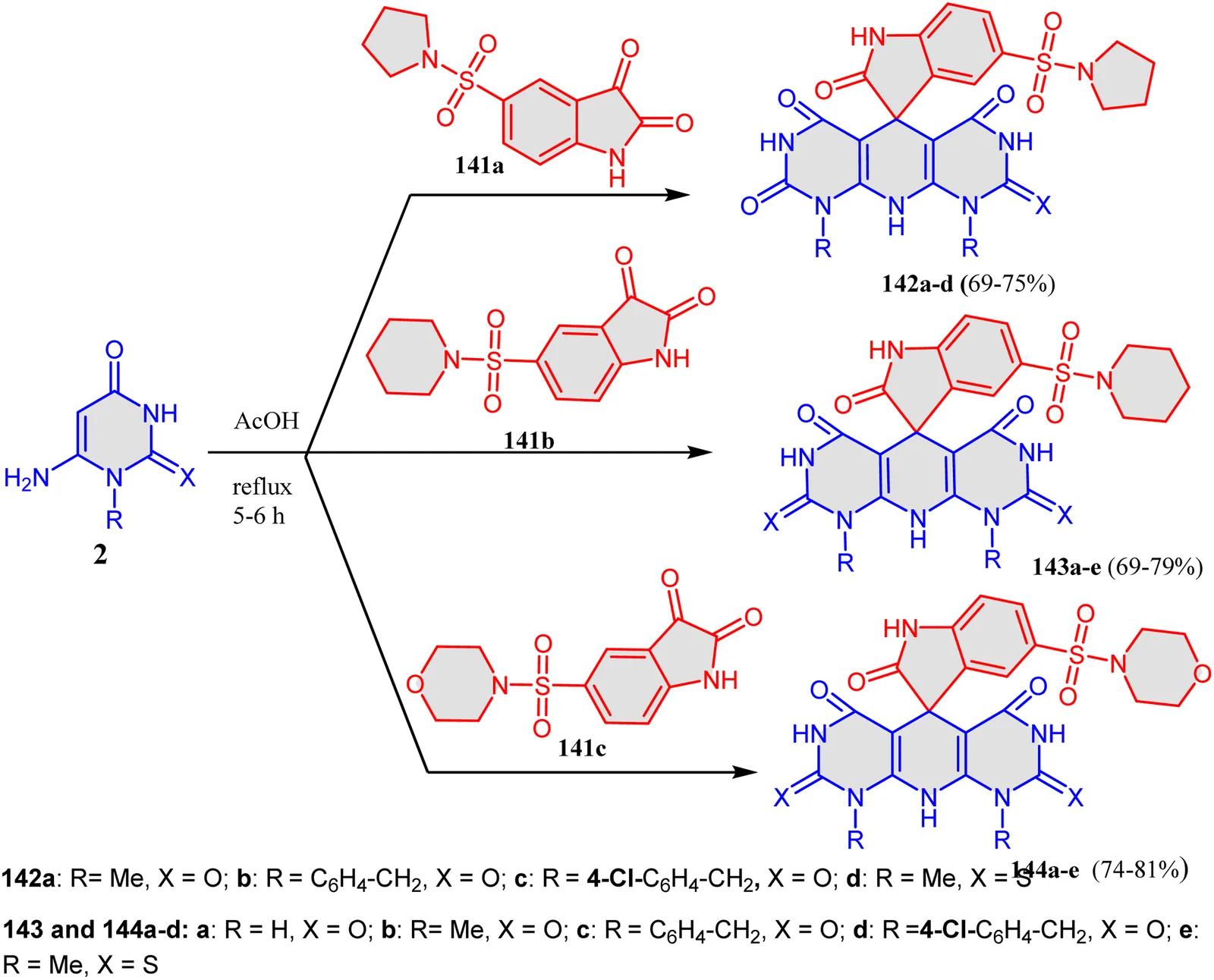
Reaction of 6-aminouracil 2 with 5-(substituted-1-yl-sulfonyl)indoline-2,3-diones 141a–c.

Moreover, El-Kalyoubi *et al.*^100^ fortunately reacted 6-aminouracils 2 with 5-(substituted-1-yl-sulfonyl)indoline-2,3-diones 141a–c in AcOH under reflux conditions to give the final products of spiro-oxindole-pyrido[2,3-*d*:6,5-*d*′]dipyrimidines 142–144 in good yields, as illustrated in Scheme 49.^100^ The antiviral activity of the synthesized compounds against SARS-CoV-2 was examined. All products were evaluated for the percentage of inhibition using the plaque reduction assay, which demonstrated that compounds 142a, 143b, 143d, and 143e had a high activity. The four compounds exhibited potent inhibitory activity ranging from 40.23 ± 0.09 to 44.90 ± 0.08 nM and from 40.27 ± 0.17 to 44.83 ± 0.16 nM, respectively, when compared with chloroquine as a reference standard, which showed 45 ± 0.02 and 45 ± 0.06 nM against RdRp and spike glycoprotein, respectively. These four substances were used to further study their mechanisms of action on spike glycoprotein and RNA-dependent RNA polymerase (RdRp). The results were quite encouraging, demonstrating effectiveness comparable to that of chloroquine, which was previously used in the treatment of COVID-19.

##### 2.2.5.4 Structure activity relationship

**Fig. 12:**
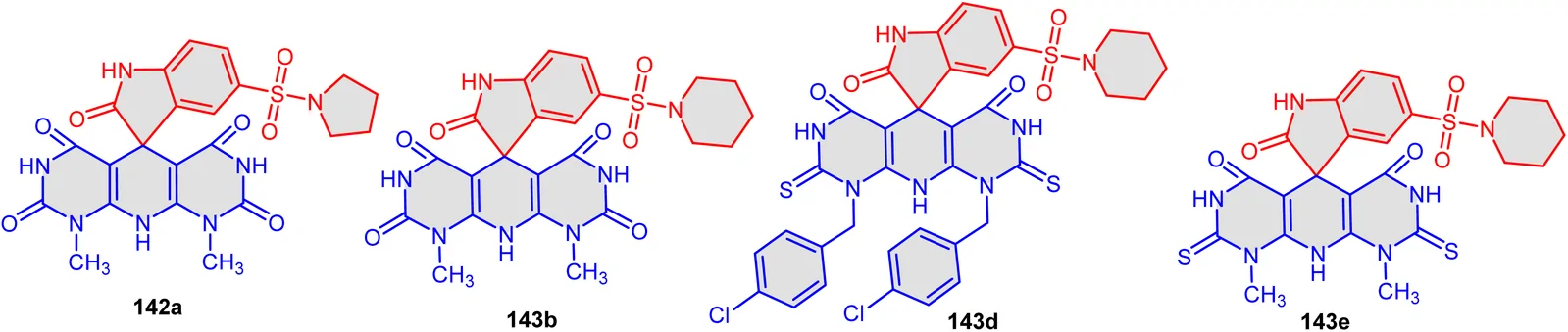
Structures of compounds 142a, 143b, 143d and 143e.

SAR of compounds 142a, 143b, 143d and 143e (Fig. 12) indicates that 142a, which has R = Me and X = O, is the most active derivative among these series 142a–d against replication of the virus, with the percentage of inhibition = 84%. Additionally, replacing the methyl group with a benzyl or 4-Cl-Bn moiety, as in compounds 143b and 143c, the activity decreased to 75% and 0%, respectively. This decreases in activity is because of the presence of the benzylidene moiety in general, and the substitution of one hydrogen bond by a chlorine atom at position four in the benzylidene moiety leads to the removal of the activity. Furthermore, 5-(piperidin-1-ylsulfonyl)-1′*H*-spiroindoline derivatives 143b and 143e with a methyl moiety and carbonyl or thiocarbonyl (X = O or S) showed the highest activity among the tested derivatives against the replication of SARS-CoV-2, with a percentage of inhibition of 99% and 91%, respectively. Moreover, 1′*H*-spiroindoline derivative 143c demonstrated an inhibition percentage of 74%, and this decrease in activity may be related to the presence of the benzyl moiety. Additionally, compound 143d exhibited good activity with an inhibition percentage of 80% when the substitutions were R = 4-Cl-Bn and X = O.

**Scheme 50:**
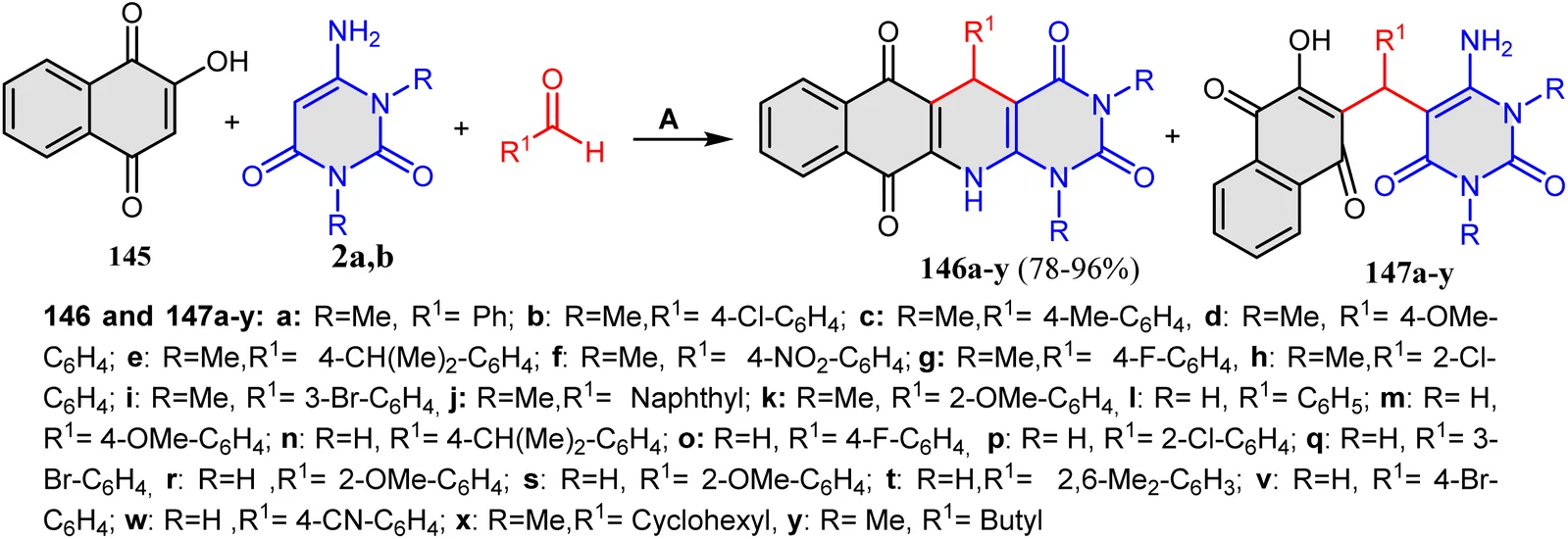
Microwave-assisted synthesis of pyridopyrimidines 146a–y. Reagents and conditions: A; MW, AcOH/H_2_O.

Under microwave irradiation, the reaction of aminouracil derivatives 2a,b, 2-hydroxy-1,4-naphthaquinone (145) and different aldehydes in AcOH/H_2_O for 25 min afforded pyrimido[4,5-*b*]quinolines 146a–y. Minor products of compound 147a–y were also formed and isolated during the reaction, as shown in Scheme 50.^134^

**Scheme 51:**
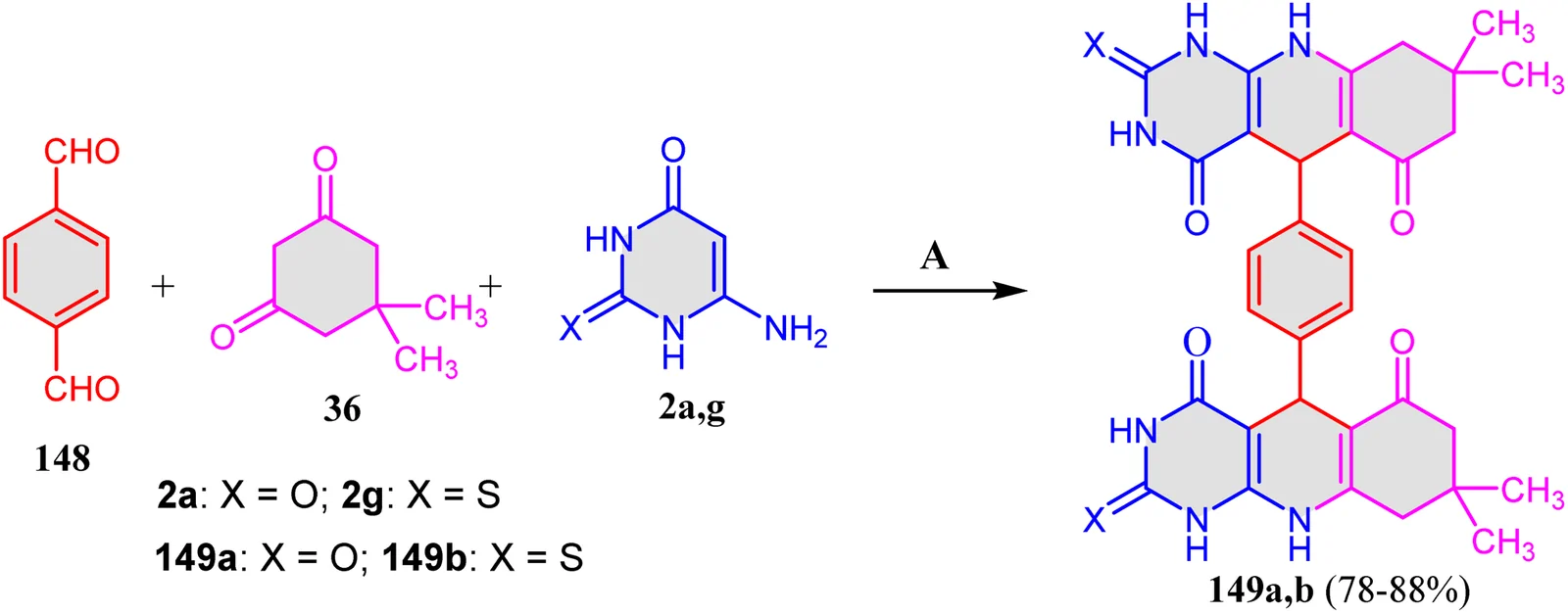
Synthesis of bis pyrido[2,3-*d*]pyrimidine scaffolds 149a,b. Reagents and conditions: A; MW, 5 min, AcOH.

Recently, in 2025, Alatawi *et al.*^135^ reacted 6-aminouracil derivatives 2a,g, terephthalaldehyde (148) and dimedone (35) in AcOH under microwave irradiation for 5 min to form bis-pyrido[2,3-*d*]pyrimidine derivatives 149a,b in 78–88% yield (Scheme 51).^135^ Compounds 149a and b displayed great efficiency in inhibiting the *candida albicans* fungus's growth, with MIC levels between 51 and 74 mg mL^−1^, which is similar to the positive control. MIC levels range from 62 to 82 mg mL^−1^. Meanwhile, in 2020, Masoumi *et al.*^136^ performed the same reactions in EtOH in a catalyst-free reaction condition to obtain the same scaffolds.

**Scheme 52:**
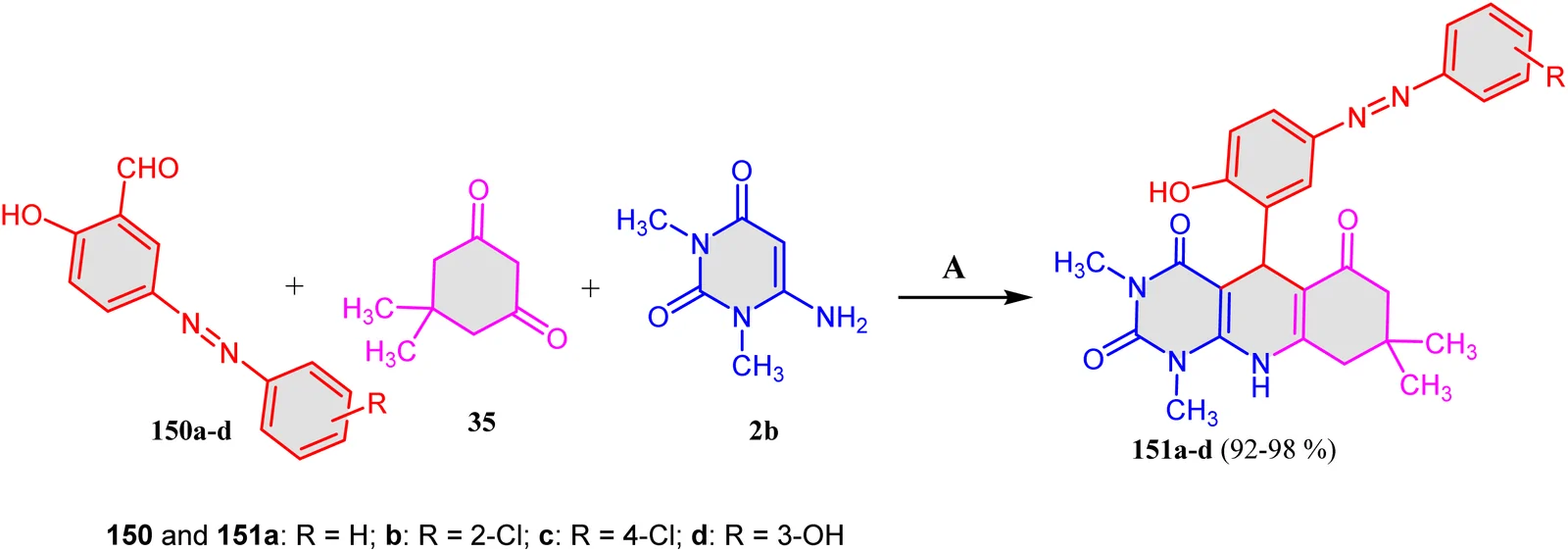
Three-component synthesis of pyrimido[4,5-*b*]quinolines 151a–d. Reagents and conditions: A; choline chloride, oxalic, B = DESs, 80 °C.

Moreover, Gholami and his group^136^ reported a multicomponent reaction with azo aldehydes 150, dimedone (35) and 6-amino-1,3-dimethyluracil (2b) to obtain pyrimido[4,5-*b*]quinoline derivatives 151a–d in 92–98% yields. The reaction was carried out in deep eutectic solvents (a class of solvents formed by the eutectic mixture of two or more compounds, resulting in a liquid with a melting point significantly lower than that of its individual components) and choline chloride/oxalic acid (ChCl/Oxa) at 80 °C was present (Scheme 52).^137^

**Scheme 53:**
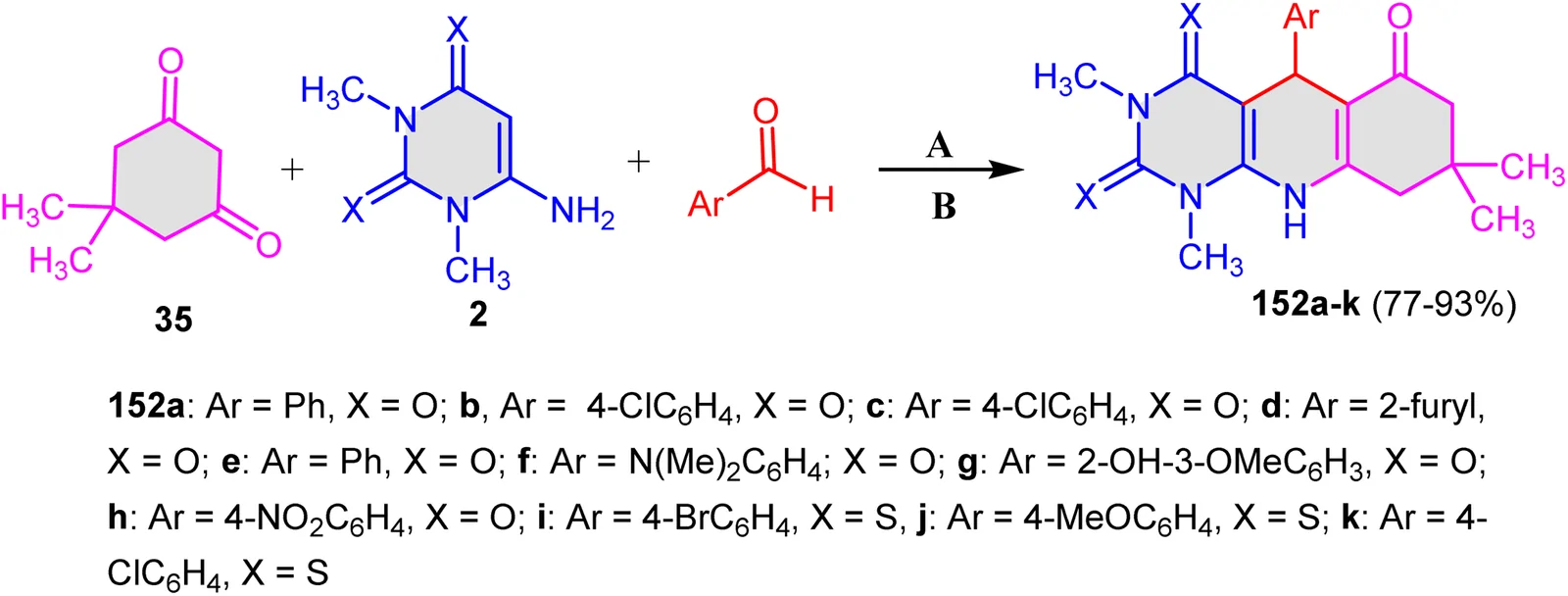
Synthesis of naphthyridines 152a–k. Reagents and conditions: A; MIL-100(Cr)/NHEtN(CH_2_PO_3_H_2_)_2_; B; DMF, 100 °C.

In continuation to previous discussion about the synthesis of pyrido-pyrimidine frameworks, tetrahydropyrimido[4,5-*b*]quinolines 152a–k were obtained in high yields (77–93%) through a three-component reaction between dimedone (35), 6-aminouracil derivatives 2 and different aldehydes under reflux conditions in the presence of MIL-125(Ti)–N(CH_2_PO_3_H_2_)_2_ as a catalyst (Scheme 53).^138^ Interestingly, the preparation of 152a–k was achieved *via* a vinylogous anomeric-based oxidation mechanism with a high yield and short reaction time.^138^

**Scheme 54:**
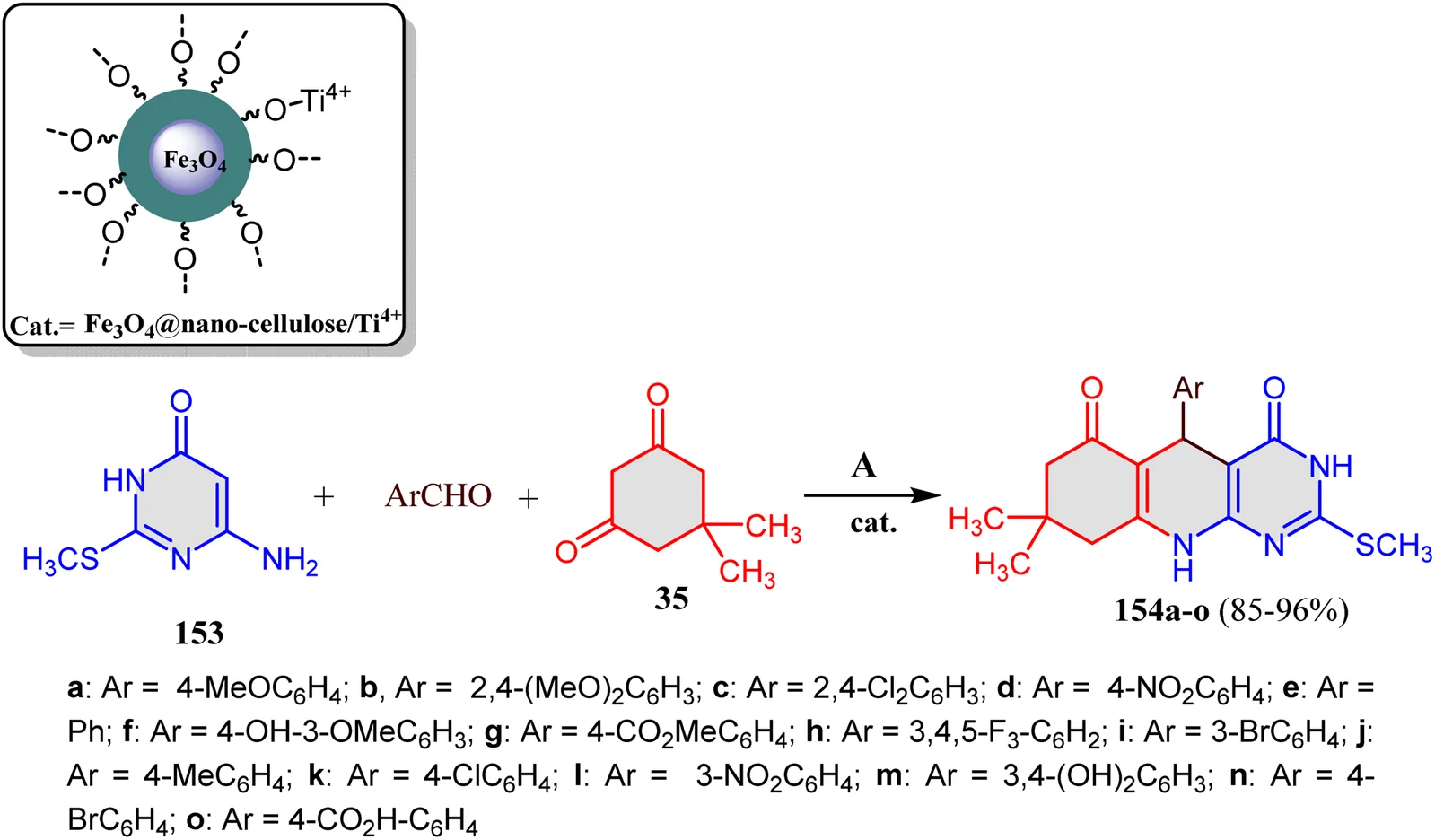
Synthesis of naphthyridines 154a–k. Reagents and conditions: A; Cat. H_2_O, 70 °C.

Mirjalili *et al.*^139^ reported the synthesis of tetrahydropyrimido[4,5-*b*]quinoline analogs 154a–o*via* the utilization of Fe_3_O_4_@nano-cellulose/Ti(iv) as a nanocatalyst. Therefore, a one-pot technique was applied in the reactions of 6-amino-2-(methylthio) pyrimidin-4(3*H*)-one (153) with aryl aldehydes and dimedone (35) under H_2_O at 70 °C (Scheme 54).

**Scheme 55:**
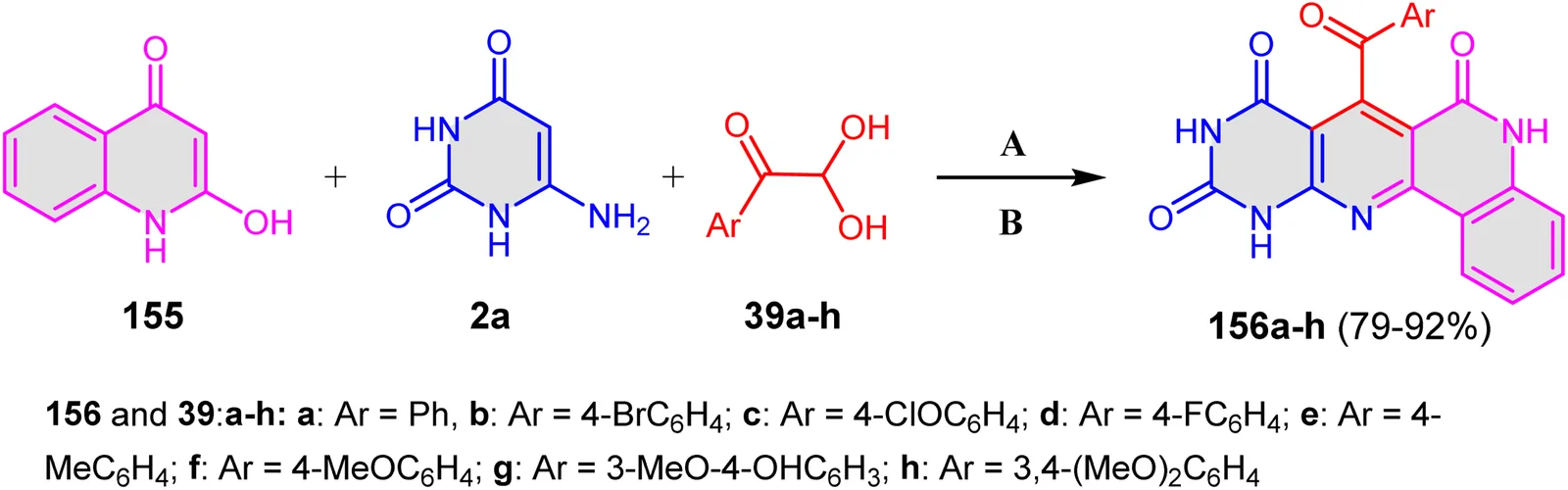
Ag NP-mediated synthesis of pyrimido[4,5-*b*][1,6]naphthyridines 156a–h. Reagents and conditions: A; Ag NPs; B; EtOH/H_2_O/60 °C.

It was also reported^140^ that silver nanoparticles were used to catalyze the reaction between 2-hydroxyquinoline-4(1*H*)-one (155), 6-aminouracil (2a) and arylglyoxal monohydrates 39a–h in a mixture solvent of EtOH/H_2_O at 60 °C, affording pyrimido[4,5-*b*][1,6]naphthyridines 156a–h as the final products in 79–92% yields (Scheme 55).^140^

The reactions of 39e, 2a and 155 were chosen as optimized reactions (Table 20). The reaction mixture was stirred using various catalysts and, in a mixture, solvent systems. A solid precipitate separated out in 50–92% yields of substituted pyrimido[4,5-*b*][1,6]naphthyridine (156e). The highest yield (91%) was achieved when the reaction was performed using 10 ppm of AgNPs as a nanocatalyst in H_2_O/EtOH (1 : 1) after 3 h of reaction time (Table 20, entry 6). To investigate the effect of the catalyst amount, the reaction was repeated in the presence of various amounts of AgNPs, as increasing the amount of catalyst did not significantly affect the reaction yield.^140^

**Table 20:** Various solvents and catalysts used in the synthesis of compounds 156a–h

Entry	Solvent	Temp. (°C)	Catalyst (mol%)	Time (h)	Yield 156e (%)
1	EtOH/H_2_O (1 : 1)	Reflux	l-proline (20 mol%)	7	52
2	EtOH	Reflux	l-proline (20 mol%)	7	58
3	EtOH	Reflux	*p*-TSA (20 mol%)	6	57
4	EtOH/H_2_O (1 : 1)	Reflux	*p*-TSA (20 mol%)	6	50
5	EtOH/H_2_O (1 : 1)	60	AgNPs (5 ppm)	3	74
6	EtOH/H_2_O (1 : 1)	60	AgNPs (20 ppm)	3	92
7	H_2_O	60	AgNPs (10 ppm)	24	—
8	AcOH	70	l-proline (20 mol%)	10	79
9	AcOH	Reflux	l-proline (20 mol%)	10	70

The one-pot, three-component reaction of aryl 39a–h, 2a and 155 in the presence of AgNPs (10 ppm) using H_2_O/EtOH (1 : 1) as a solvent afforded the desired compounds 156a–h in high yields (Table 21).^140^

**Table 21:** Time of reaction for 39a–h, 2a and 155 and the yield (%) for the synthesis of products 156a–h

Entry	Ar	Time (min)	Yields (%)
1	Ph	300	156a (79)
2	4-BrC_6_H_4_	240	156b (88)
3	4-ClC_6_H_4_	270	156c (82)
4	4-FC_6_H_4_	240	156d (87)
5	4-Tol	180	156e (91)
6	4-MeOC_6_H_4_	150	156f (92)
7	3-MeO-4-HOC_6_H_3_	180	156g (89)
8	3,4-(MeO)_2_C_6_H_3_	210	156h (90)

**Scheme 56:**
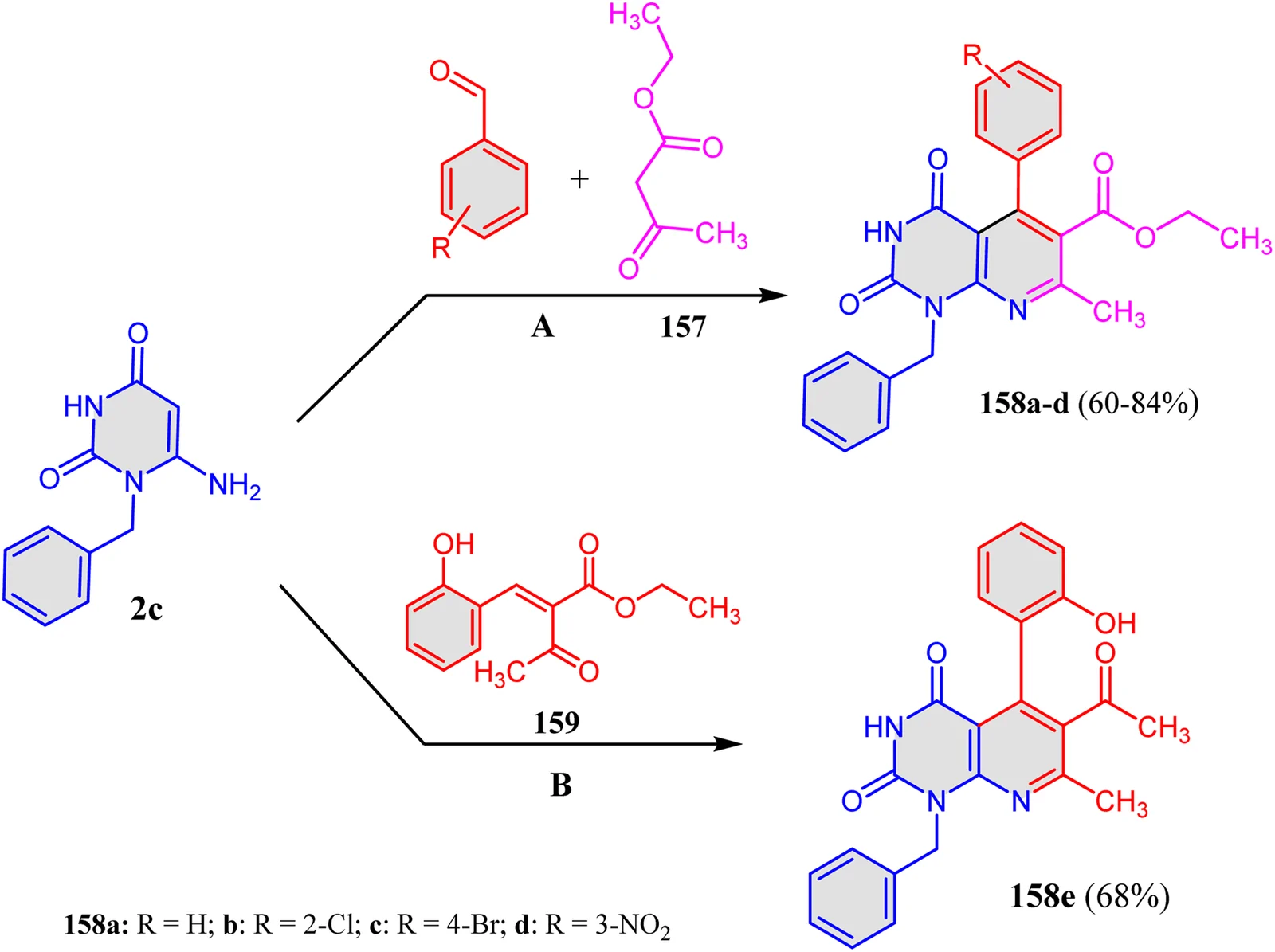
Reaction of 1-benzyl-6-aminouracil with compound ethyl acetoacetates 157 and 159. Reagents and conditions: A; EtOH, TEA, reflux, 12 h. B; DMF, TEA, reflux, 4 h.

El-Kalyoubi *et al.*^141^ reacted 1-benzyl-6-aminouracil 2c, aromatic aldehydes and ethyl acetoacetate (157) in EtOH/Et_3_N at reflux temperature for 12 h to give tetrahydropyrido[2,3-*d*]pyrimidine-6-carboxylates 58a–d in 60–84% yields. During the reaction of 1-benzyl-6-aminouracil 2c with ethyl 2-(2-hydroxybenzylidene)acetoacetate (159) under the same conditions for 4 h, the reaction proceeded to give 6-acetyl-1-benzyl-5-(2-hydroxyphenyl)-7-methylpyrido[2,3-*d*]pyrimidine-2,4(1*H*,3*H*)-dione (158e), as shown in Scheme 56.^141^

**Scheme 57:**
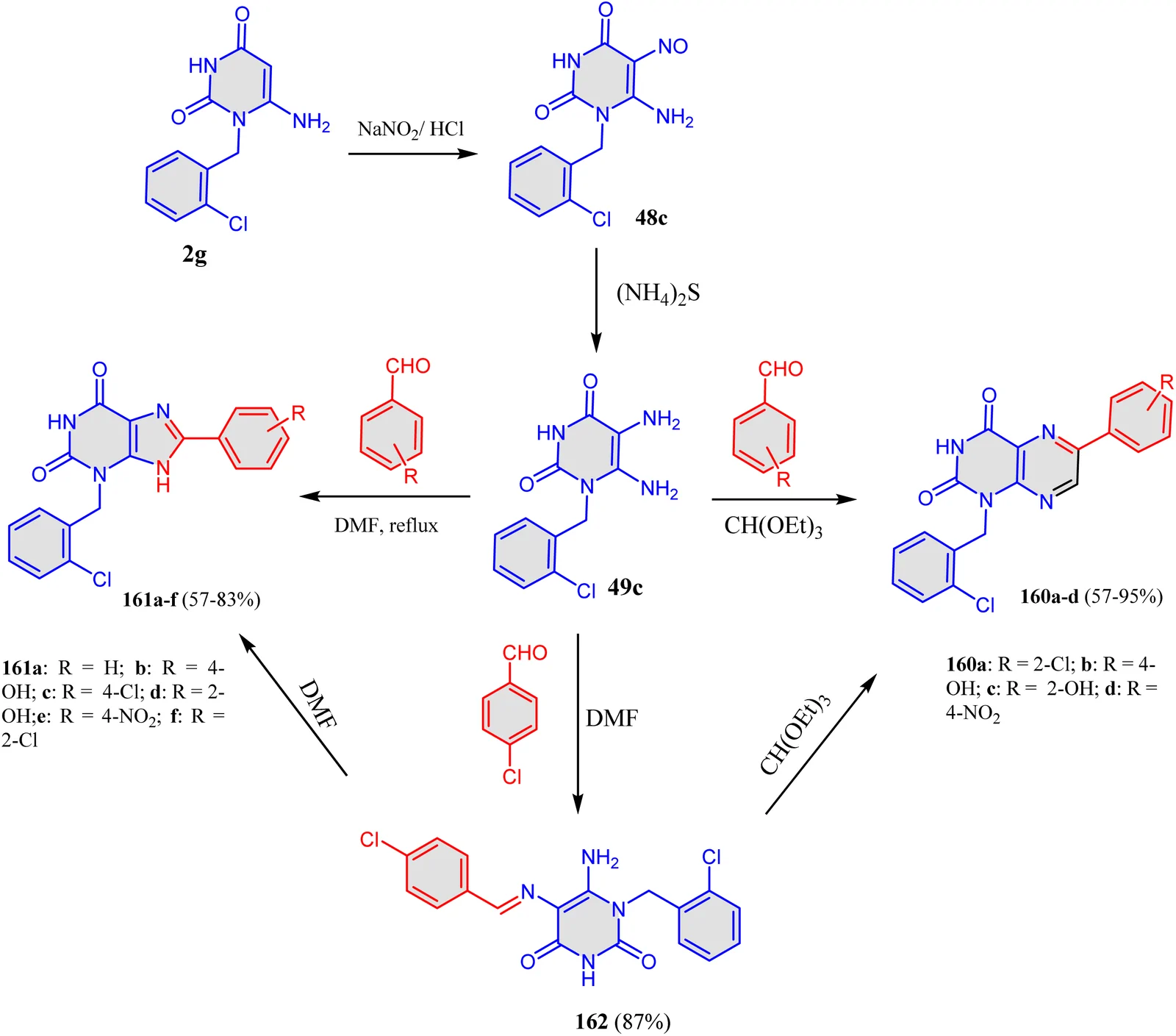
Synthesis of pteridines 160a–d and purine compounds 161a–f.

The same authors^141^ also prepared compounds containing pteridine and purine frameworks. The strategy started with nitrosation of 1-(2-chlorobenzyl)-6-aminouracil 2g to give 6-amino-1-(2-chlorobenzyl)-5-nitrosopyrimidine-2,4-dione (48c), which was treated with ammonium sulfide (NH_4_)_2_S to produce 5,6-diamino-1-(2-chlorobenzyl)-pyrimidinedione (49c) (Scheme 57), when compound 49c reacted with triethyl orthoformate and various aromatic aldehydes afforded pteridine derivatives 160a–d. However, when it reacted only with aromatic aldehydes, it afforded purine 161a–f. On the other hand, when diaminouracil 49c reacted with 4-chlorobenzaldhyde, it gave ylidene form 162, which produced the corresponding pteridine 160 when it was treated with triethyl orthoformate. Besides, refluxing ylidene 162 in DMF gave the corresponding purine (Scheme 57).^141^

The formed products were examined for *in vitro* lung carcinoma inhibitory activity against the cell line A549. The results showed that the most effective compounds against lung carcinoma are 158b, 161c, 161d, 161e, 160c and 160d (Table 22) using methotrexate as a drug reference. The pteridine scaffold containing the 2-Cl-Bn group 158b exhibited good inhibitory activity (IC_50_ = 10.3 ± 0.2). Purine compounds 161c, 161d, and 161e, which have R = 4-Cl, 2-OH and 4-NO_2_, displayed IC_50_ = 27.0 ± 1.1, 23.1 ± 0.6, and 26.3 ± 1.3, respectively. Moreover, pteridine compounds 160c and 160d with an OH group in positions 2 and 4 demonstrated a significant increase in inhibition activity (IC_50_ = 24.9 ± 1.2 and 12.2 ± 0.3) compared to the reference methotrexate (IC_50_ = 36.3 ± 3.9).^141^

**Table 22:** *In vitro* inhibitory activity of tested compounds against the human lung carcinoma cell line (A549) expressed as IC_50_ values μM ± standard deviation

Tested Compd	Structure	IC_50_ values (μM)	Tested compounds	Structure	IC_50_ values (μM)
158a	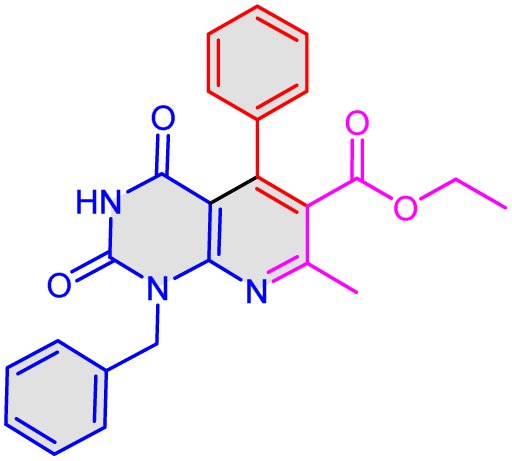	237 ± 6.3	161c	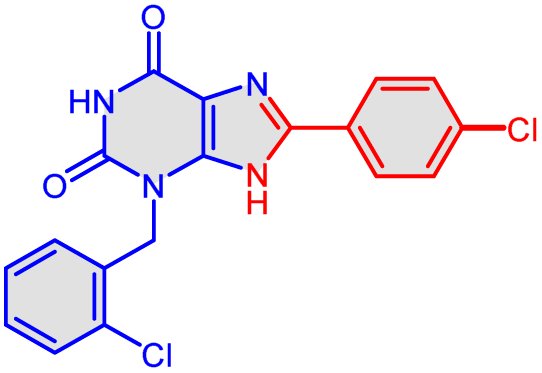	27.0 ± 1.1
158b	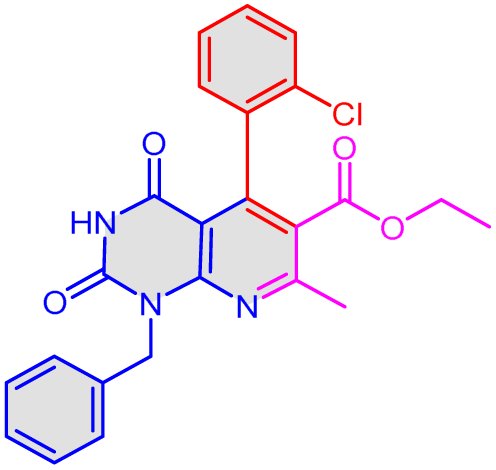	10.3 ± 0.2	161d	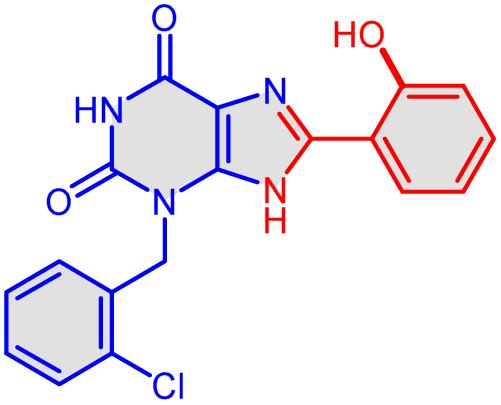	23.1 ± 0.6
158c	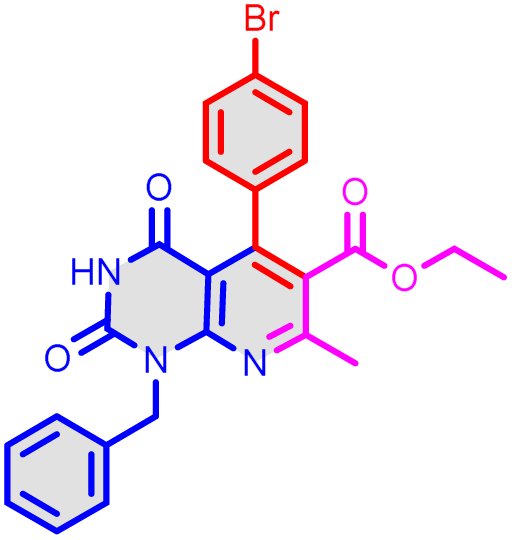	349 ± 7.8	161e	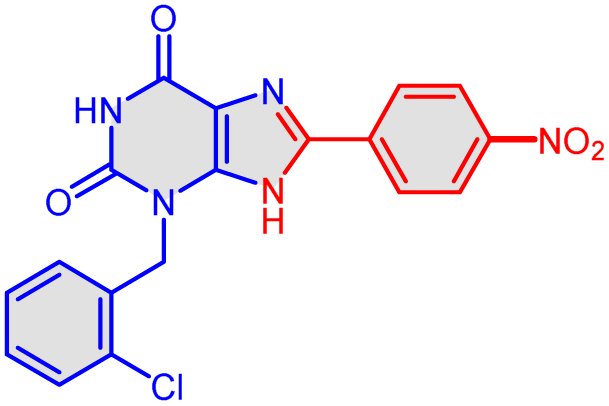	26.3 ± 1.3
158d	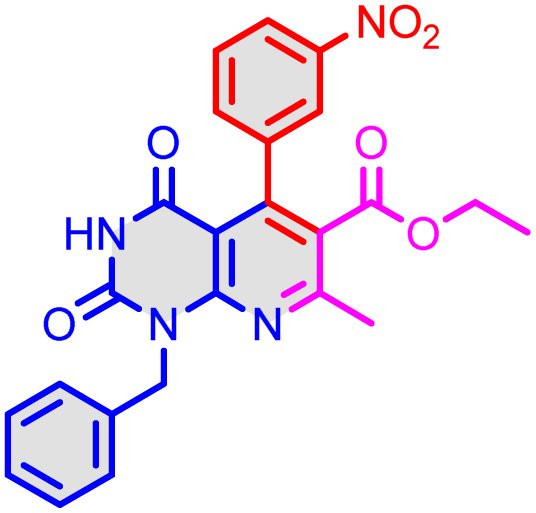	59.5 ± 2.5	161f	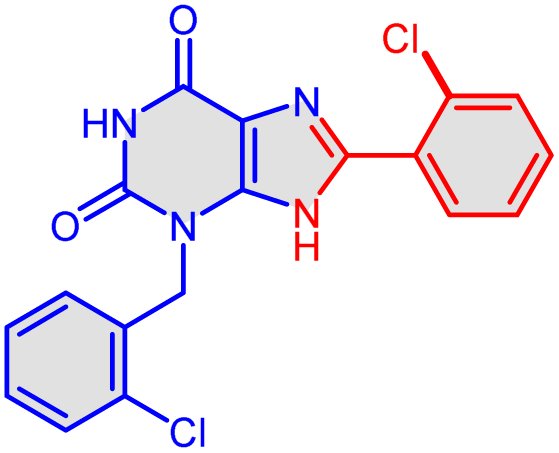	141 ± 3.9
158e	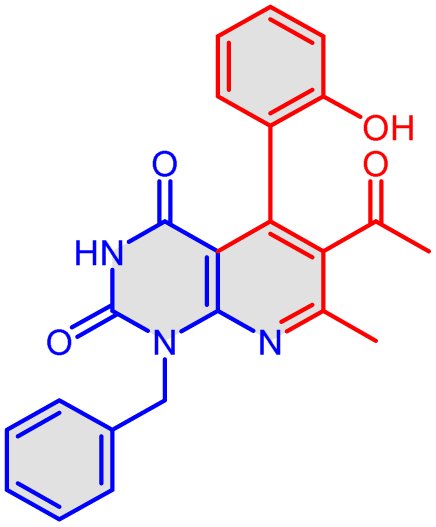	246 ± 7.1	160a	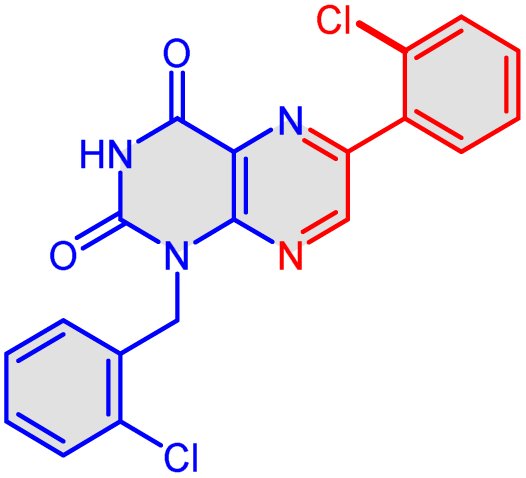	86.1 ± 2.8
162	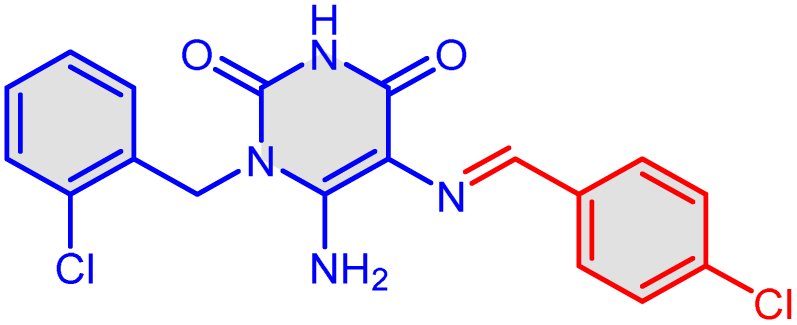	62.0 ± 2.4	160b	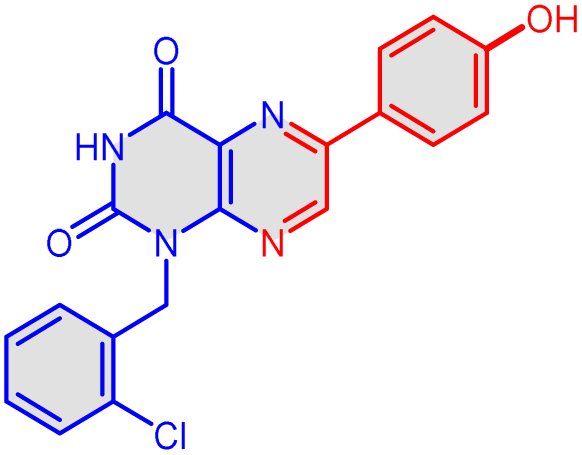	84.8 ± 3.4
161a	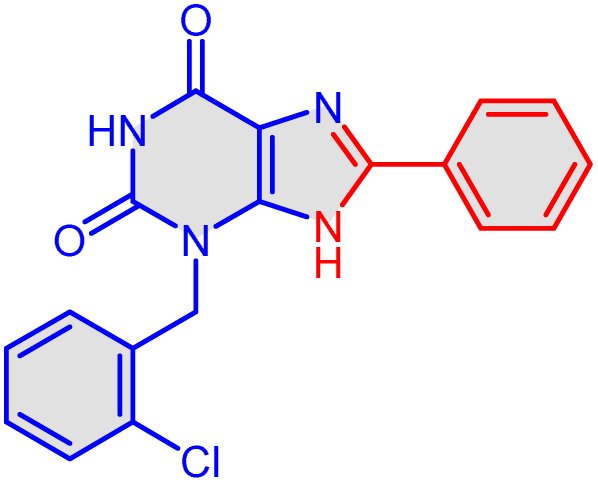	54.0 ± 1.8	160c	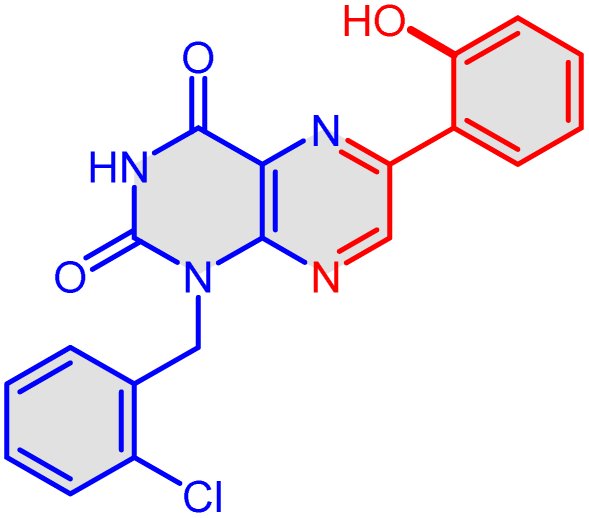	24.9 ± 1.2
161b	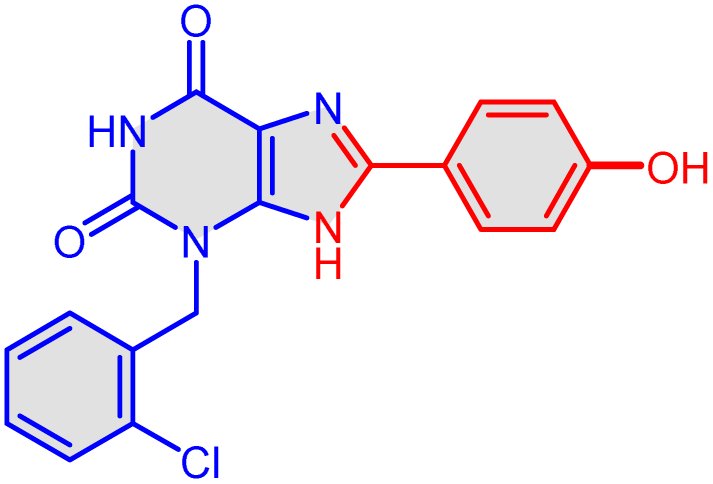	58.5 ± 1.7	160d	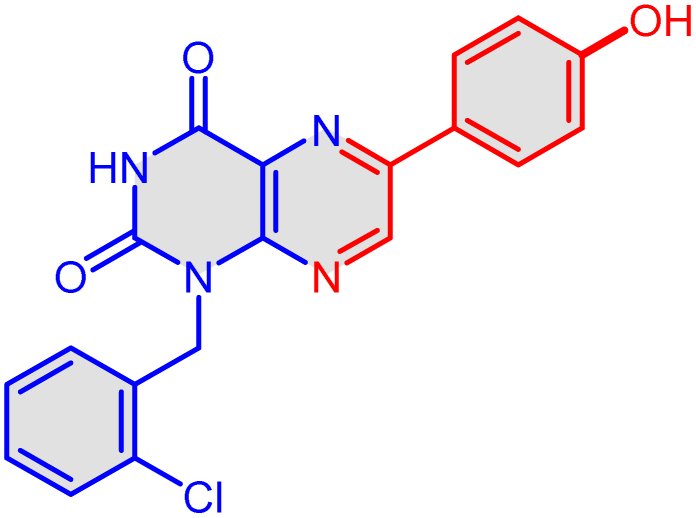	12.2 ± 0.3
**Methotrexate**		**36.3** ±**3.9**			

**Scheme 58:**
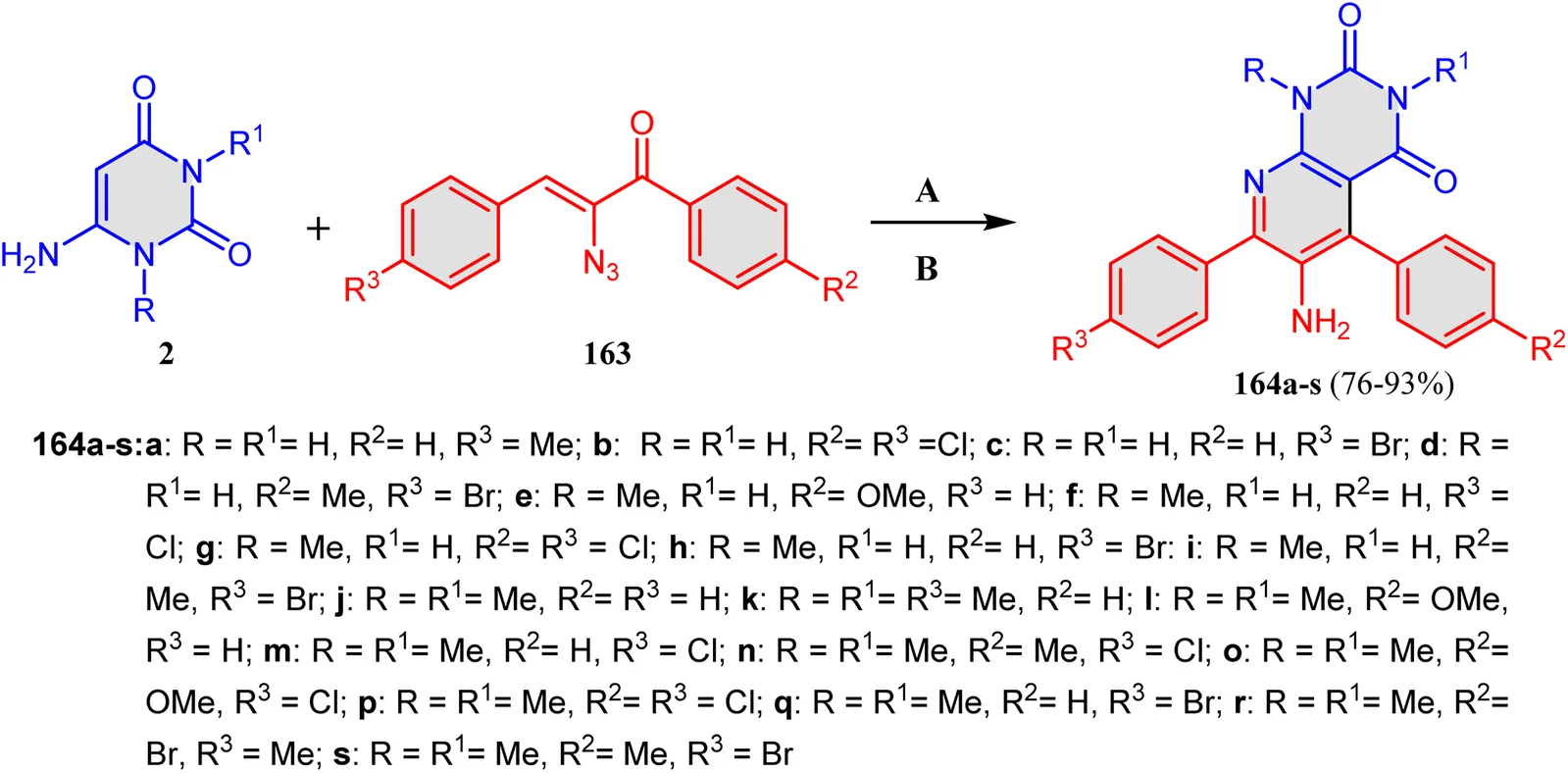
Reaction of α-azidochalcones 163 with 6-amiouracils 2. Reagents and conditions: A = DMF, Et_3_N; B = 50 °C, 30 min.

The synthesis of 6-amino-pyrido[2,3-*d*]pyrimidine-2,4-diones 164a–s was reported by Adib *et al.*^142^ through the reaction between α-azidochalcones 163 and 6-amiouracil derivatives in DMF/Et_3_N at 50 °C for 30 min (Scheme 58). The corresponding products were formed in high yields (79–93%). α-Glucosidase inhibitory activity of the products 164a–s was investigated and displayed significant *in vitro* yeast α-glucosidase inhibition with IC_50_ values between 78.0 ± 2.0 and 252.4 ± 1.0 μM. According to the results, the compound with the highest significant activity was 164o and was around 10-fold more potent than the reference drug, acarbose (IC_50_ = 750.0 ± 1.5 μM).^142^ Compound 164a with 4-Me substituent on the 7-phenyl ring showed the most inhibitory activity in this series (IC_50_ = 164.5 ± 1.5 mM). The replacement of the methyl group with a Br atom in the mentioned position (compound 164c) decreases the inhibitory activity. Moreover, the anti-α-glucosidase activity decreased in compound 164b (R^2^ = R^3^ = Cl). In the case of derivatives 164e–i (R = Me), a better result was obtained for compound 164i with (R^2^ = 4-CH_3_ and R^3^ = 4-Br). The absence of a methyl group in compound 164h decreased the inhibitory effect. The activity of compound 164f (R^3^ = Cl) decreased (IC_50_ = 224.0 ± 1.5 mM). The presence of a chlorine atom (R = Me, R^2^ = R^3^ = Cl) in compound 164g results in a significant increase in inhibition (IC_50_ = 187.0 ± 1.0 mM). Compound 164e (R^3^ = 4-OMe) was the second most potent compound in this series. A comparison of the IC_50_ values of derivatives with R = Me 164g–i with their analogs (R = H) 164b–d showed that the derivatives containing the Me group were more potent than the others. Furthermore, in 164j–s derivatives, compound 164o with (R^2^ = 4-OCH_3_ and R^3^ = 4-Cl) was the most potent compound among all the synthesized compounds (IC_50_ = 78.0 ± 2.0 mM) (Table 23).^142^

**Table 23:** *In vitro* α-glucosidase inhibitory activity of compounds 164a–s and acarbose (as a reference drug)

Compd	Structure	IC_50_ (μM)	Compd	Structure	IC_50_ (μM)
164a	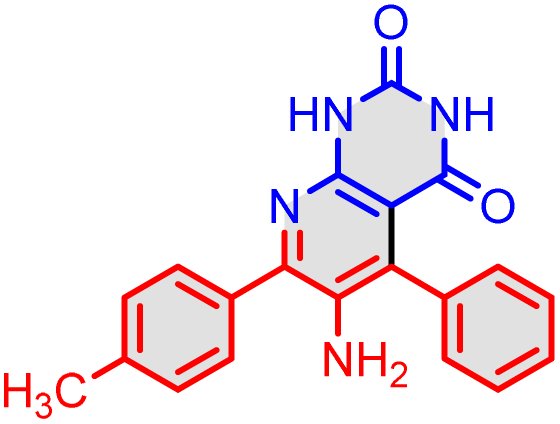	164.5 ± 1.5	164k	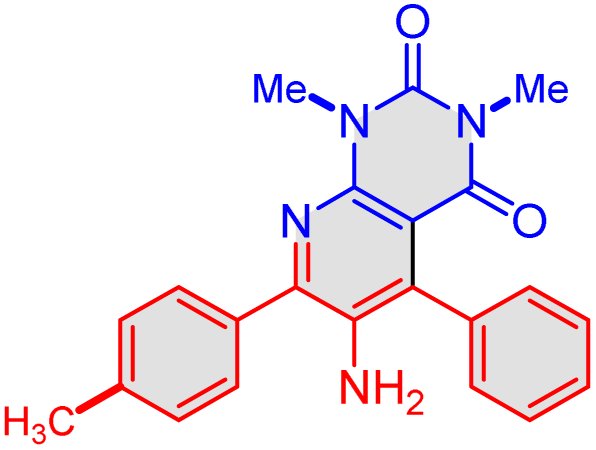	145.0 ± 1.5
164b	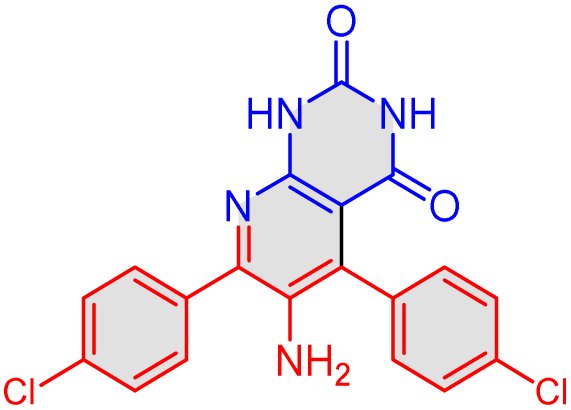	237.0 ± 1.0	164l	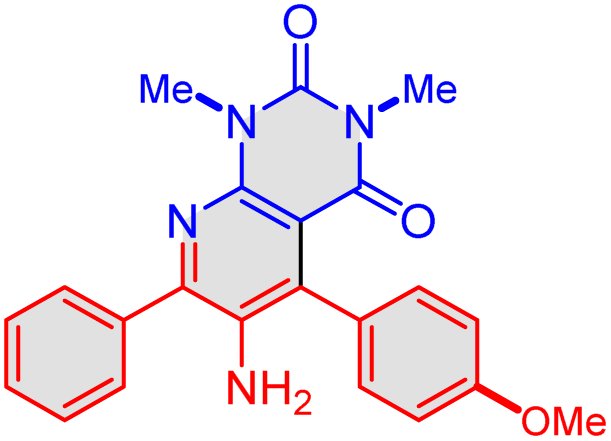	185.0 ± 1.0
164c	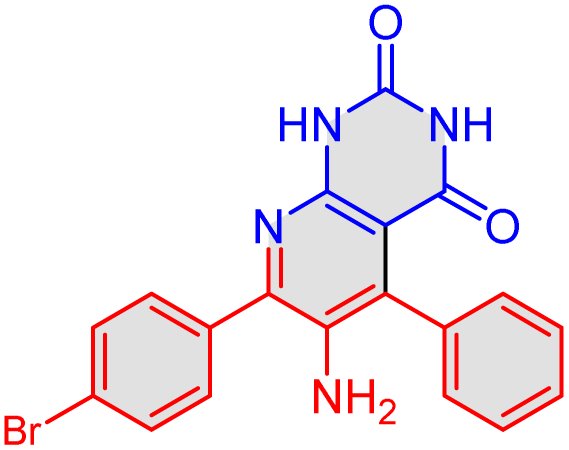	223.2 ± 1.5	164m	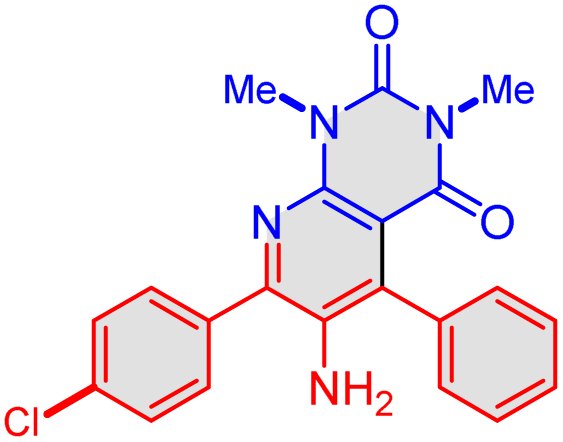	122.3 ± 2.5
164d	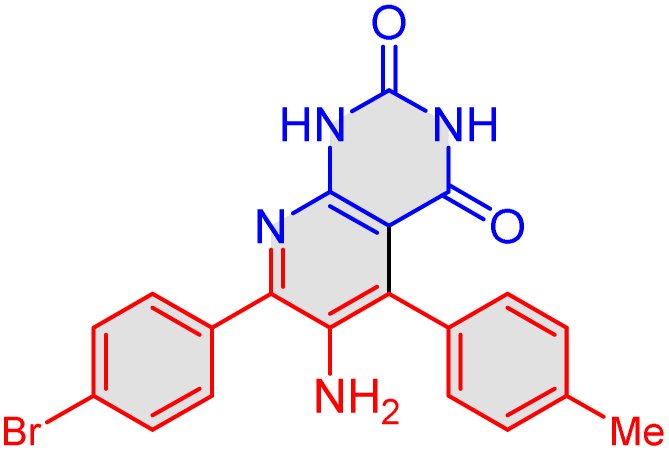	222.0 ± 2.0	164n	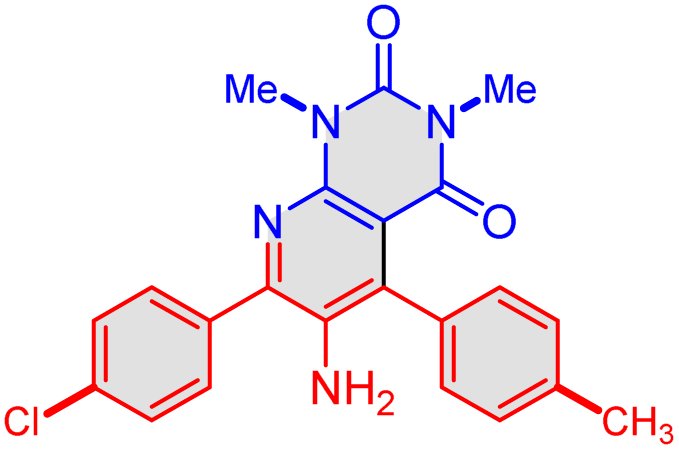	177 ± 1.0
164e	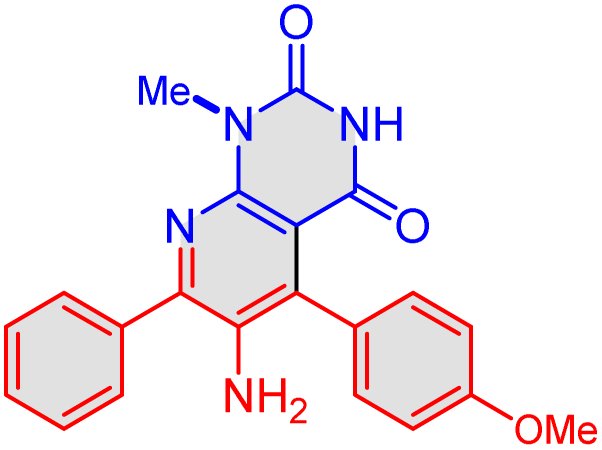	112.5 ± 1.0	164o	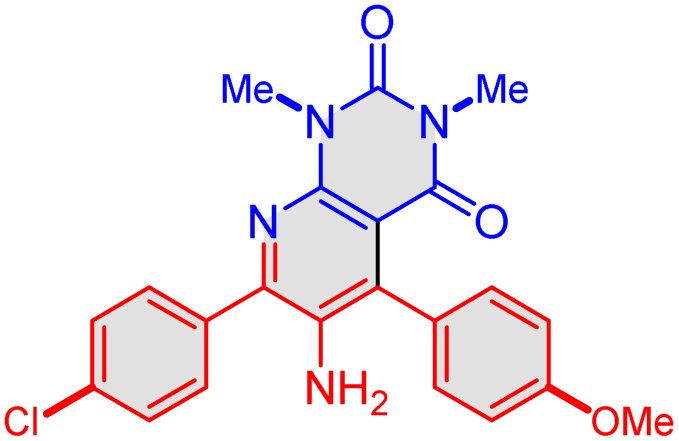	78.0 ± 2.0
164f	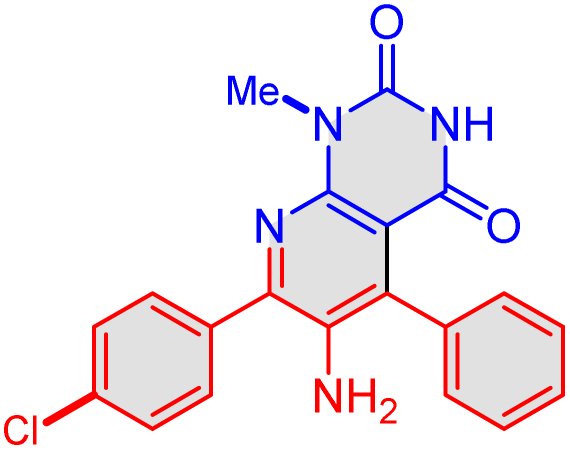	224.0 ± 1.5	164p	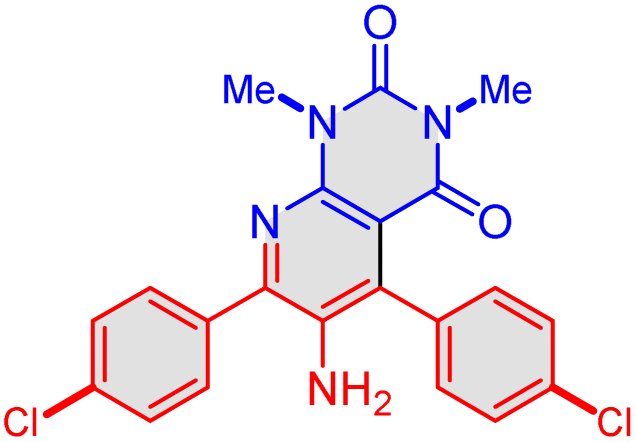	215.0 ± 1.0
164g	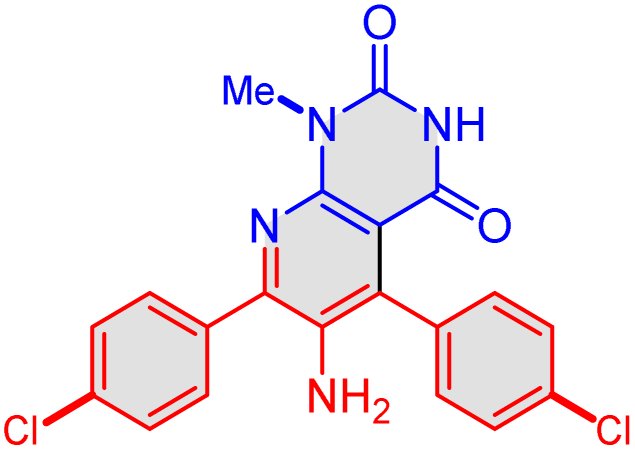	187.0 ± 1.0	164q	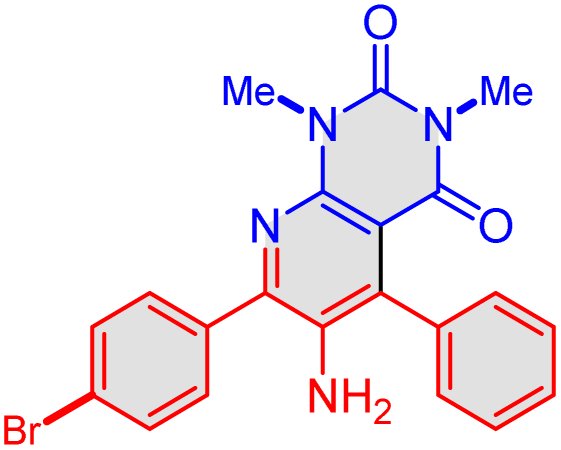	132.0 ± 1.5
164h	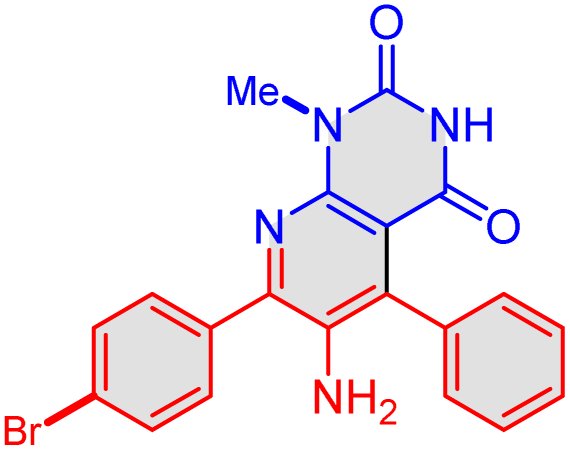	177.1 ± 1.0	164r	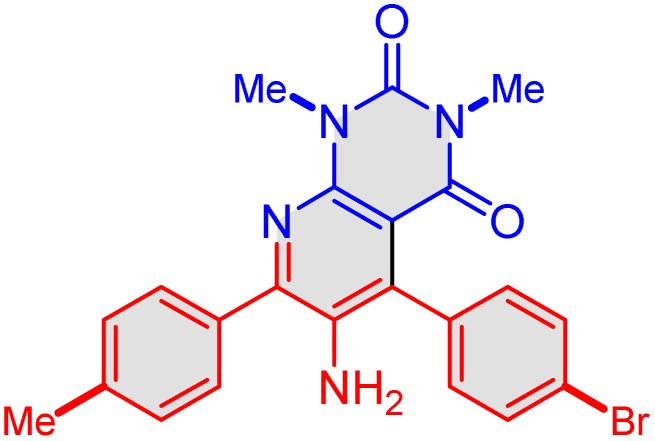	193.0 ± 1.5
164i	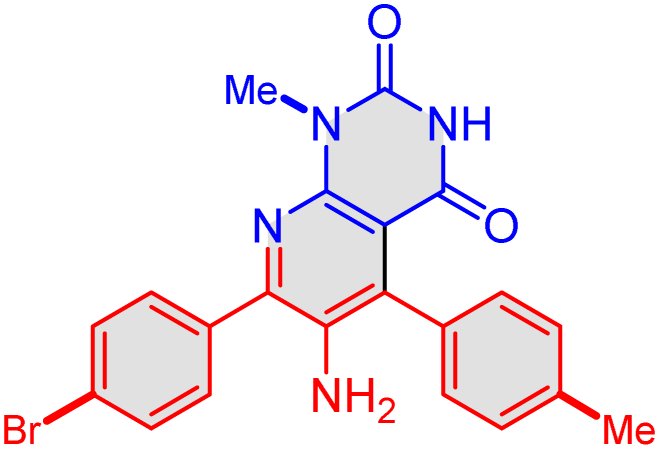	91.0 ± 1.0	164s	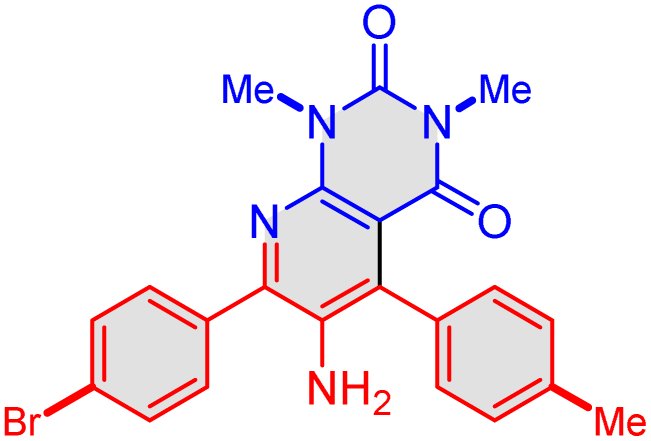	236.0 ± 1.3
164j	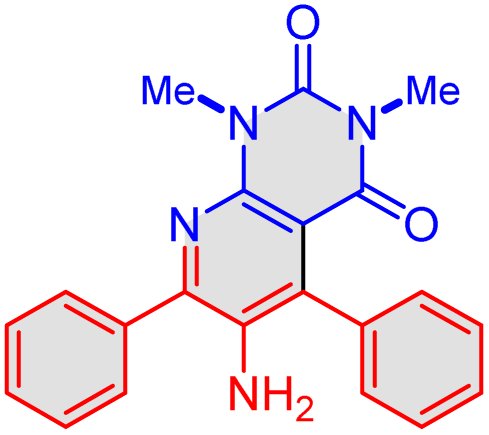	252.4 ± 1.0			
**Acarbose**		**750.0** ±**1.5**			

**Scheme 59:**
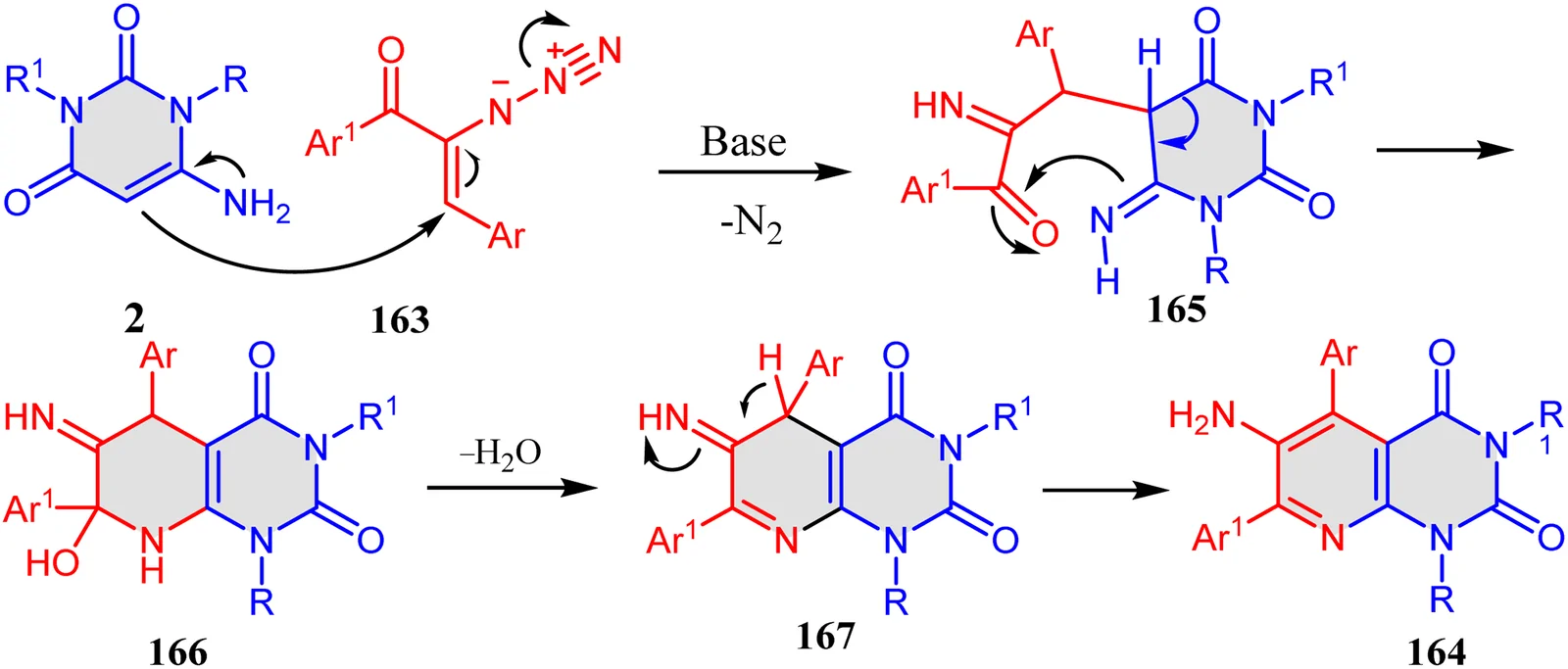
Suggested mechanism for the formation of compound 164a–s.

A plausible mechanism for the synthesis of 6-amino-pyrido[2,3-*d*]pyrimidine-2,4-diones 164a–s is illustrated in Scheme 59. The mechanism could be explained by the presence of base C-5 of 6-aminouracil 2, which would undergo a Michael addition to α-azidochalcone 163, and the nitrogen molecule was eliminated to produce adduct 165. Next, cyclic form 166 was formed *via* a nucleophilic attack of the imine on the adjacent carbonyl. The adduct 166 then loses a molecule of H_2_O to form intermediate 167. Finally, a proton shift occurred, leading to the generation of the final products 164a–s.^142^

**Scheme 60:**
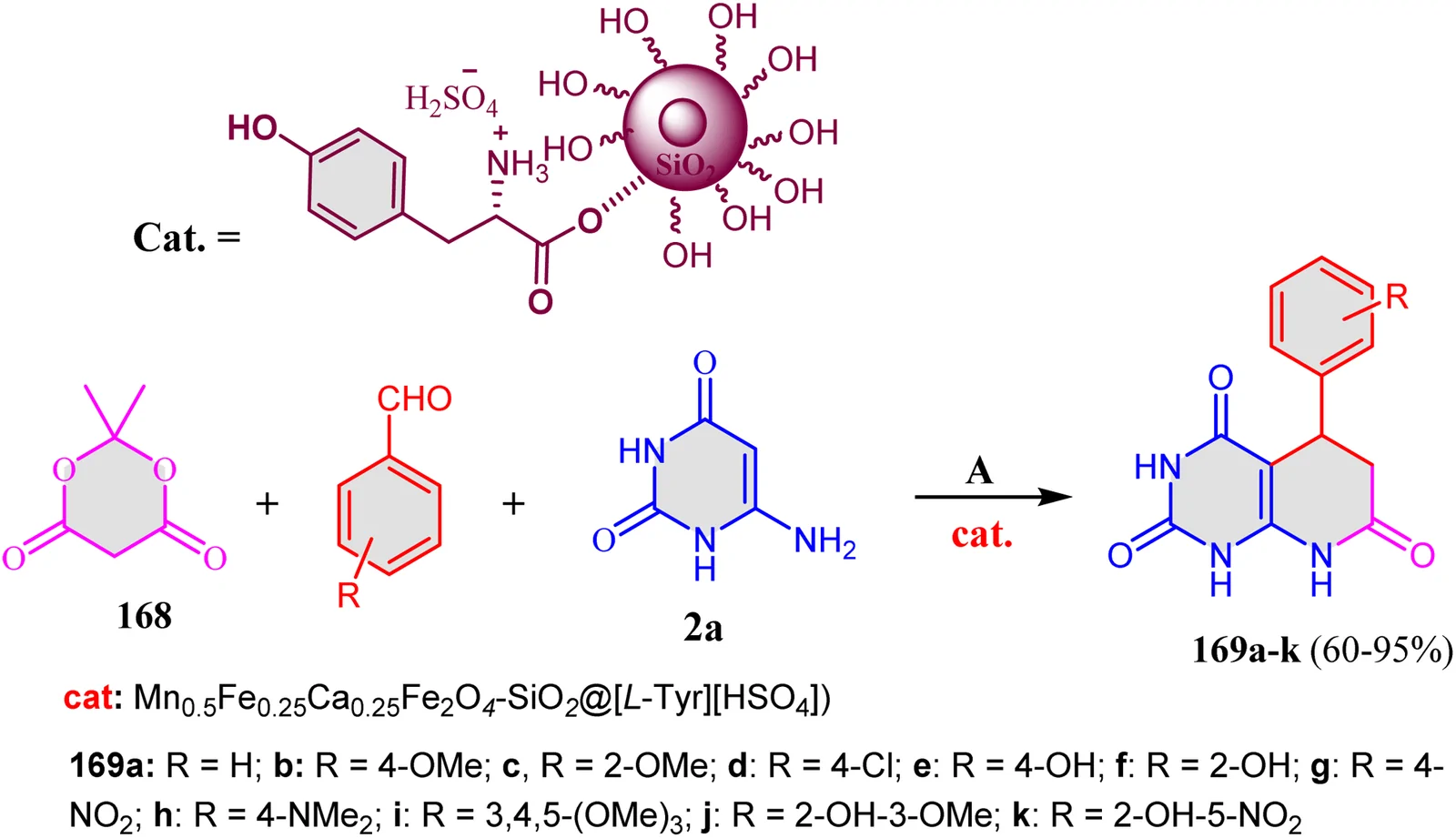
Formation of dihydropyrido[2,3-*d*]pyrimidines 169a–k. Reagents and conditions: A; H_2_O, 70 °C.

Shaddel *et al.*^143^ developed a green synthetic technique for the synthesis of dihydropyrido[2,3-*d*]pyrimidine 169a–k. The reaction proceeded between Meldrum's acid 168, 2a and aldehydes in H_2_O at 70 °C in the presence of l-tyrosinium hydrogen sulfate IL (Mn_0.5_Fe_0.25_Ca_0.25_Fe_2_O_4_–SiO_2_@[l-Tyr][HSO_4_]) as a catalyst to obtain the final products 169a–k (Scheme 60).

**Scheme 61:**
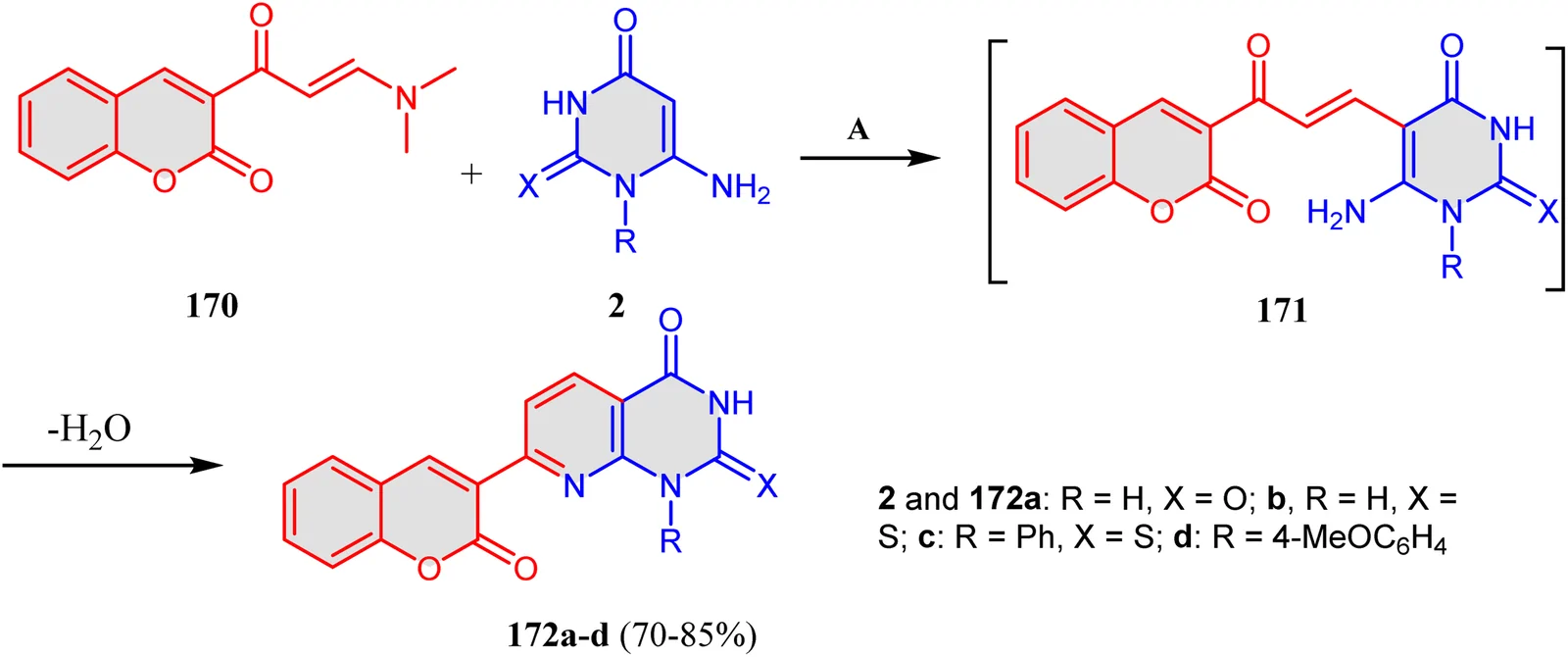
Reaction of 3-(3-(dimethylamino)acryloyl)-2*H*-chromen-2-one 170 with 6-aminouracils 2. Reagents and conditions: A; AcOH, reflux, 5–6 h.

Recently, Abdelaal *et al.*^144^ synthesized pyrido[2,3-*d*]pyrimidine 172a–d bearing coumarin ring through the reaction of 3-(3-(dimethylamino)acryloyl)-2*H*-chromen-2-one (170) with 6-aminouracil derivatives 2 in AcOH at reflux temperature for 5–6 h (Scheme 61). Compound 172a demonstrated a potent and selective activity against CNS cancer cell lines U251 and SF-295 (80.51% and 96.86%, respectively). Compound 172c displayed lethal activity (GI > 100%) toward 41 tumor cell lines that belong to all the nine subpanels except leukemia (GI = 23.13–97.50%). This broad activity was narrowed to non-small cell lung cancer NCI-H226 (GI = 64.09%) and ovarian cancer cell line OVCAR-3 (75.53%) by the addition of a 4-methoxy group to the appended phenyl ring at N−1 (compound 172d).^144^

#### 2.2.6. Synthesis of pyrimido[4,5-*d*]pyrimidine-2,4-dione derivatives

**Scheme 62:**
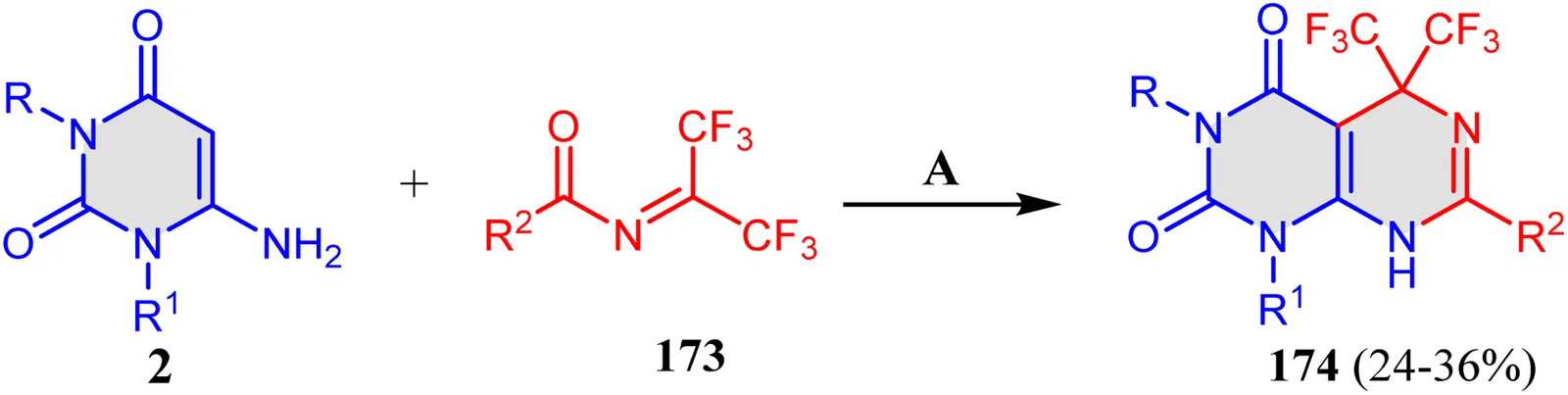
Reaction of 6-aminouracils 2 with compound 173. Reagents and conditions: A = DMF, Et_3_N.

On mixing substituted 6-aminouracils 2 with *N*-acylimines of hexafluoroacetone (173) in DMF and using Et_3_N as a catalyst, the reaction afforded substituted 5,5-bis(trifluoromethyl)-5,8-dihydro-pyrimido[4,5-*d*]pyrimidine-2,4-diones 174a–e in 24–36% yields (Scheme 62 and Table 24). The glucagon-like peptide-1 receptor (GLP-1R) is a major area of interest in the treatment of type II diabetes. It is essential for increasing insulin secretion and decreasing glucagon secretion.^145^ The results showed that 7-(4-chlorophenyl)-1,3-dimethyl-5,5-bis(trifluoromethyl)-5,8-dihydropyrimido-[4,5-*d*]pyrimidine-2,4(1*H*,3*H*)-dione (174e) is an effective GLP-1R antagonist. Compound 174e inhibited Exendin-4 (EX-4) simulation, which blocked insulin release in islets; moreover, it decreased insulin levels while increasing blood glucose. The structures and GLP-1R antagonist activities of representative analogs 174a–e are highlighted in Table 24; when R = H (174a, 174b, and 174c), the compounds were inactive (IC_50_ > 10 μM), resulting in weak partial antagonists, while in 174b (R^1^ = benzyl and R^2^ = 2-thienyl), more GLP-1R antagonist potency was observed (GLP-1 (7–36) amide, IC_50_ = 1.3 μM, pIC_50_ = 5.89 ± 0.04, 36.2 ± 1.9 GLP-1 min; exendin-4, IC_50_ = 1.5 μM, pIC_50_ = 5.82 ± 0.06, 32.0 ± 2.4 GLP-1 min) than the lead 174a. GLP-1 antagonist potency increased when Me was substituted for hydrogen at R (Table 24).^145^

**Table 24:** Structures and activities of compounds 174a–e

Entry	R	R^1^	R^2^	GLP-1R	GLP-1R p IC_50_	GLP-1 min (%)
				^IC^_50_ (μM)^a^^,^^b^		
174a	H	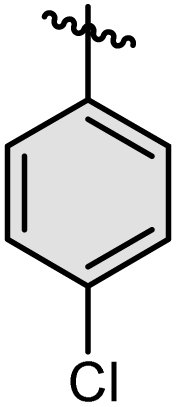	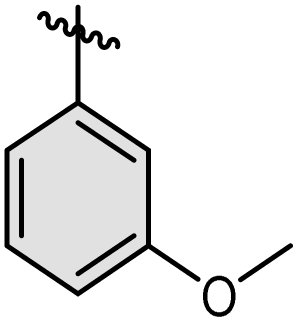	5.9^a^	5.23 ± 0.09^a^	16.8 ± 6.3^a^
				5.8^b^	5.24 ± 0.08^b^	23.9 ± 5.5^b^
174b	H	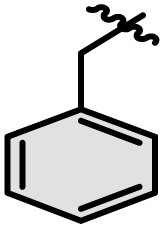	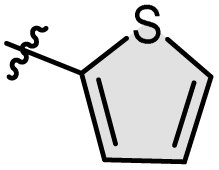	1.3^a^	5.89 ± 0.04^a^	36.2 ± 1.9^a^
				1.5^b^	5.82 ± 0.06^b^	32.0 ± 2.4^b^
174c	H	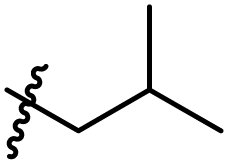	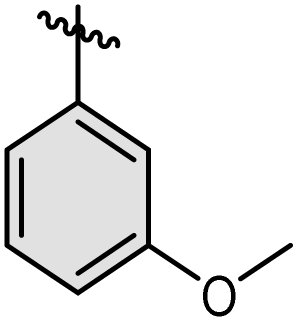	7.2^a^	5.14 ± 0.08^a^	22.7 ± 6.2^a^
				7.2^b^	5.14 ± 0.08^b^	26.4 ± 6.2^b^
174d	Me	Me	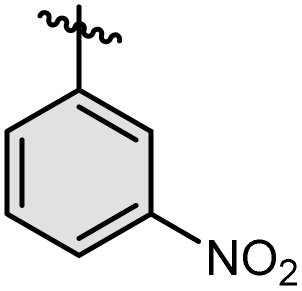	0.61^a^	6.22 ± 0.03^a^	16.4 ± 1.5^a^
				0.69^b^	6.16 ± 0.04^b^	15.5 ± 1.7^b^
174e	Me	Me	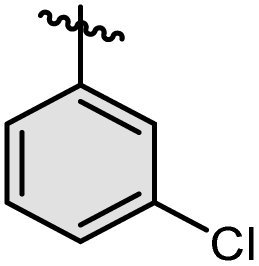	0.65^a^	6.19 ± 0.03^a^	20.9 ± 1.4^a^
				0.69^b^	6.16 ± 0.05^b^	19.7 ± 2.1^b^

a GLP-1R IC_50_, pIC_50_ and GLP-1 min (%) in TREx293 HEK cell line with GloSensor cAMP assay upon activation with an EC_80_ of GLP-1 (7–36) amide; *n* = 3.

b GLP-1R IC_50_, pIC_50_ and GLP-1 min (%) in TREx293 HEK cell line with GloSensor.

**Scheme 63:**
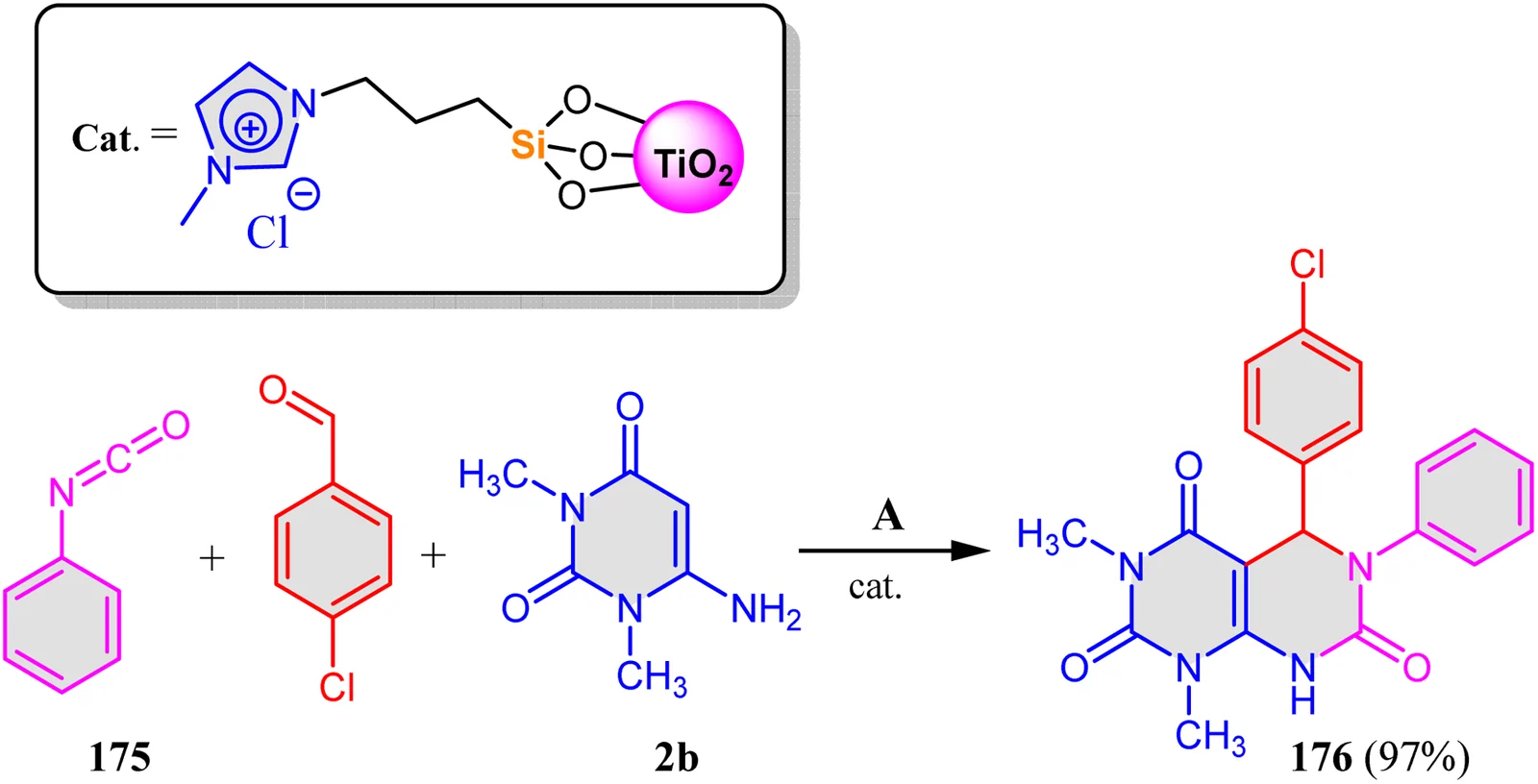
Synthesis of compound 176 using TiO_2_@LLs nanocatalyst. Reagent and conditions: A; EtOH:H_2_O, reflux, cat.

In 2020, Bakhshali-Dehkordi *et al.*^146^ utilized TiO_2_ nanoparticles to synthesize 5-(4-chlorophenyl)-1,3-dimethyl-6-phenyl-5,8-dihydropyrimido[4,5-*d*]pyrimidine-trione (176) in 97% yield. A three-component reaction was performed between phenyl isocyanate (175), 4-chlorobenzaldhyde and 1,3-dimethyl-6-aminouracil (2b) in EtOH/H_2_O containing TiO_2_ nanoparticles immobilized by an ionic liquid based on imidazole at reflux temperature (Scheme 63).^146^

**Scheme 64:**
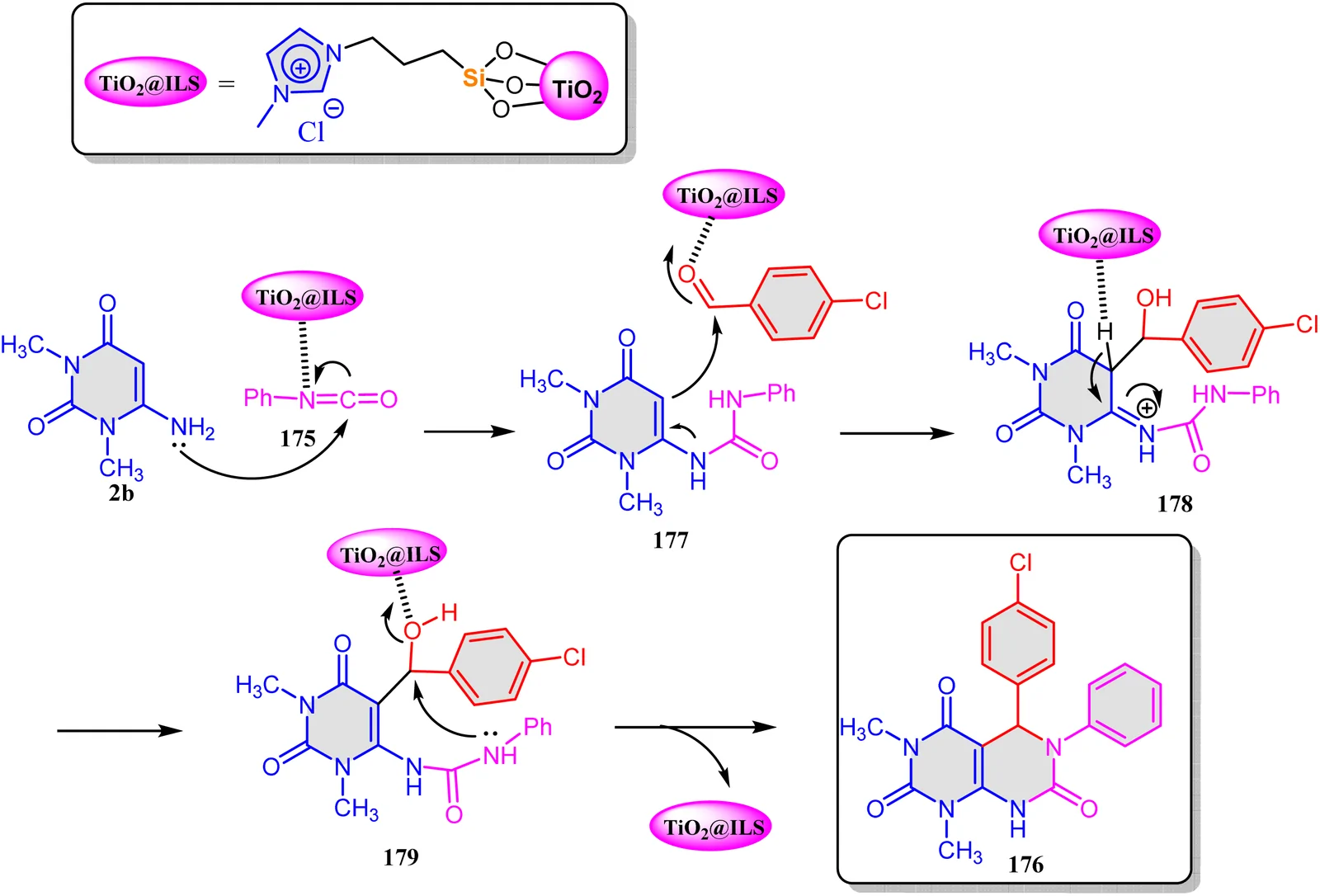
Proposed mechanism for the synthesis of compound 176.

The suggested mechanism is illustrated in Scheme 64. First, the nitrogen atom of 6-amino-*N*,*N*-dimethyluracil 2b attacked the carbonyl of 175 to obtain intermediate 177. The presence of TiO_2_@ILs promoted the nucleophilic attack of C-5 in intermediate 177 on the aldehyde to produce intermediate 178, which underwent a proton shift and rearrangement to yield intermediate 179. Finally, 179 intermolecular cyclization and dehydration were undergone to give the final product 176 (Scheme 64).^146^

**Scheme 65:**
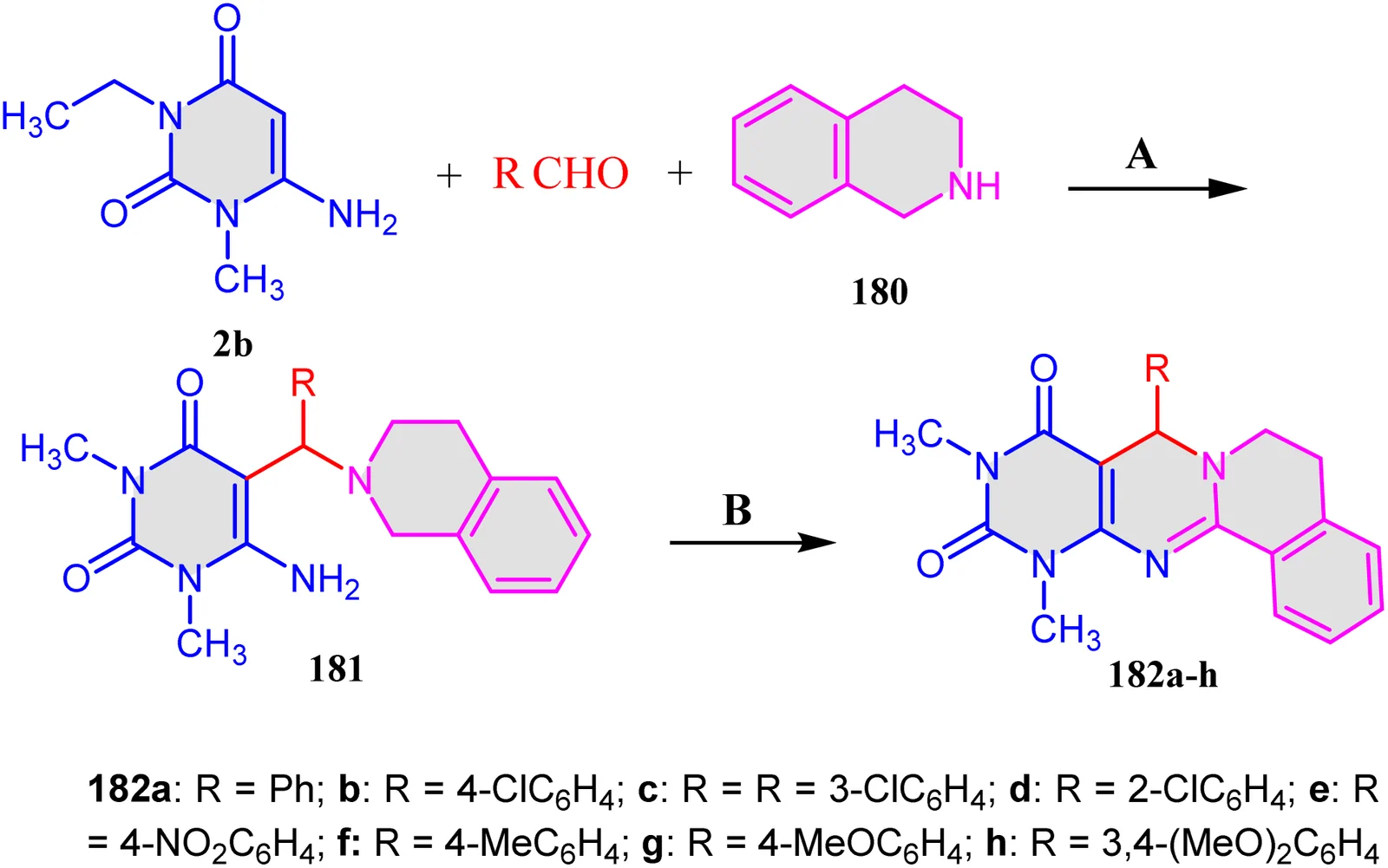
Synthesis of pyrimido[4,5-*d*]pyrimidines 182a–h. Reagents and conditions: A; EtOH, AcOH, rt; B; I_2_, TBHP, EtOH, rt.

Furthermore, Borpatra *et al.*^147^ reported an interesting approach for the formation of pyrimido[4,5-*d*]pyrimidine derivatives 182a–h*via* the reaction between 1,3-dimethyl-6-aminouracil (2b), various aldehydes and 1,2,3,4-tetrahydroisoquinoline (180) in EtOH and AcOH at room temperature to give compound 153. Following that, compound 181 was stirred in EtOH at room temperature in the presence of *t*-butyl hydroperoxide (TBHP) and I_2_ to obtain the cyclic form of pyrimido[4,5-*d*]pyrimidines 182a–h (Scheme 65).

**Scheme 66:**
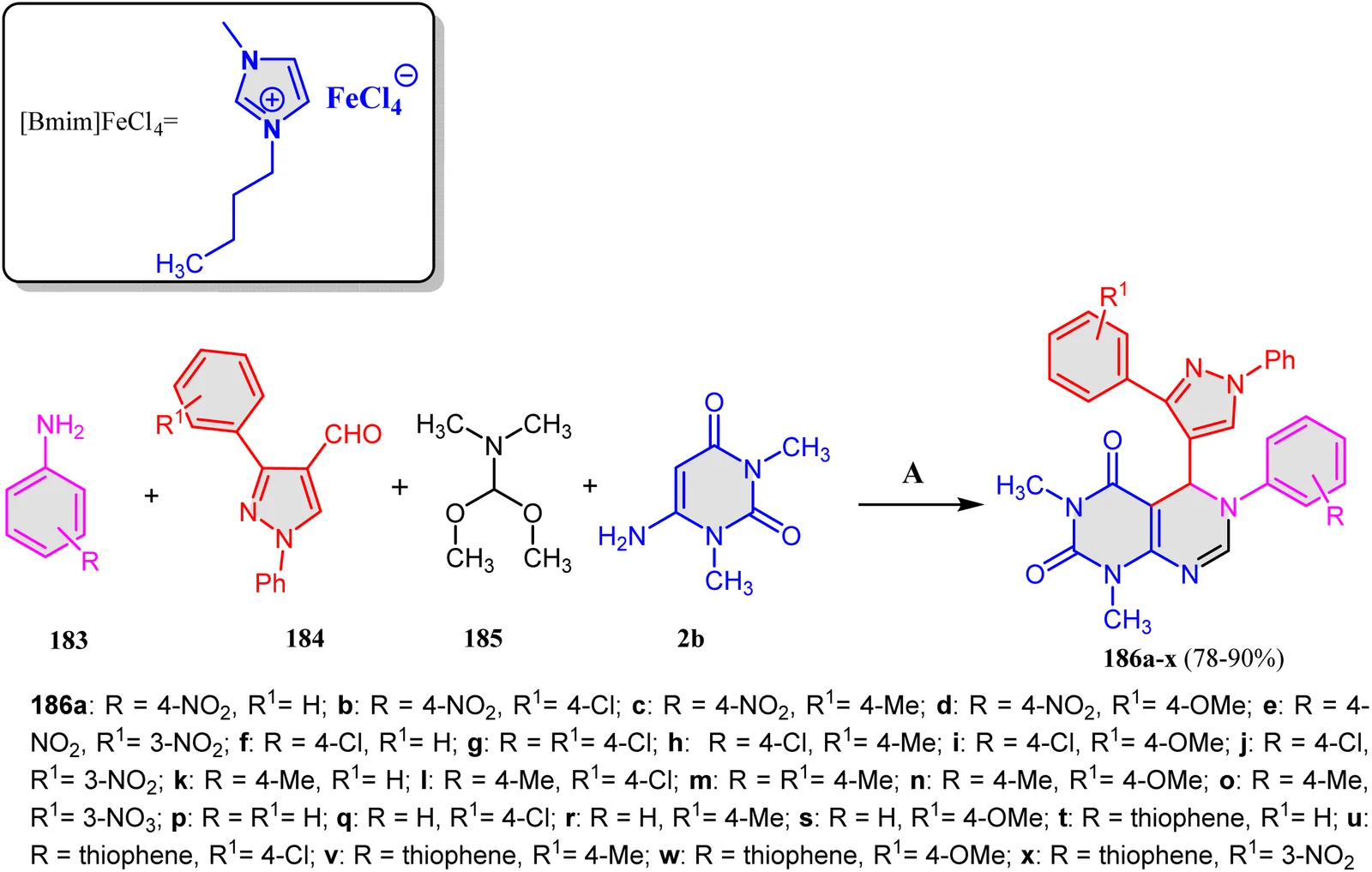
Formation of pyrazolo-pyrimido[4,5-*d*]pyrimidine hybrids 186a–x. Reagents and conditions: A; [Bmim]FeCl_4_, 80 °C.

The multicomponent reaction between anilines 183, 6-amino-1,3-dimethyluracil (2b), 1-phenyl-3-(4-substituted-phenyl)-4-formyl-1*H*-pyrazoles 184, and *N,N*-dimethylformamide dimethylacetal (185) was performed in the presence of [Bmim]FeCl_4_ to obtain pyrazolo-pyrimido[4,5-*d*]pyrimidines 186a–x in 78–90% yields (Scheme 66).^148^ The formed products were investigated for their antibacterial activity (Table 25). Among all the tested compounds, compounds 186d, 186t, 186u, 186v, and 186w had good activity with MIC values ranging from 15.6 to 31.2 μg mL^−1^, while compounds 186c, 186i, 186l, and 186m were of nearly promising activity with MIC values ranging from 3.9 to 15.6 μg mL^−1^ (Table 25).^148^

**Table 25:** Antibacterial activity of pyrazolo-pyrimido[4,5-*d*]pyrimidines 186a–x

Compd	Minimum inhibitory concentration (lg mL^−1^)						
	*Bacillus subtilis* MTCC121	*Staphylococcus aureus* MTCC 96	*S. aureus* MLS16 MTCC 2940	*Micrococcus luteus* MTCC 2470	*Klebsiella planticola* MTCC 530	*Escherichia coli* MTCC 739	*Pseudomonas aeruginosa* MTCC 2453
186a	>125	>125	>125	>125	>125	>125	>125
186b	>125	>125	>125	>125	>125	>125	>125
186c	7.8	15.6	7.8	15.6	>125	>125	>125
186d	31.2	31.2	31.2	15.6	>125	>125	>125
186e	>125	>125	>125	31.2	>125	>125	>125
186f	>125	>125	>125	>125	>125	>125	>125
186g	>125	>125	>125	>125	>125	>125	>125
186h	>125	>125	>125	15.6	>125	>125	>125
186i	31.2	15.6	15.6	7.8	>125	>125	>125
186j	>125	>125	>125	>125	>125	>125	>125
186k	>125	>125	>125	>125	>125	>125	>125
186l	7.8	7.8	7.8	3.9	>125	>125	>125
186m	7.8	7.8	15.6	7.8	>125	>125	>125
186n	>125	>125	>125	>125	>125	>125	>125
186o	>125	>125	>125	>125	>125	>125	>125
186p	62.5	31.2	15.6	7.8	>125	>125	>125
186q	>125	>125	>125	>125	>125	>125	>125
186r	>125	>125	>125	>125	>125	>125	>125
186s	>125	>125	>125	>125	>125	>125	>125
186t	31.2	31.2	15.6	15.6	>125	>125	>125
186u	15.6	31.2	15.6	15.6	>125	>125	>125
186v	31.2	15.6	15.6	15.6	>125	>125	>125
186w	31.2	15.6	15.6	15.6	>125	>125	>125
186x	>125	>125	>125	>125	>125	>125	>125
Ciprofloxacin	0.9	0.9	0.9	0.9	0.9	0.9	0.9

##### 2.2.6.1 Structure activity relationship

**Fig. 13:**
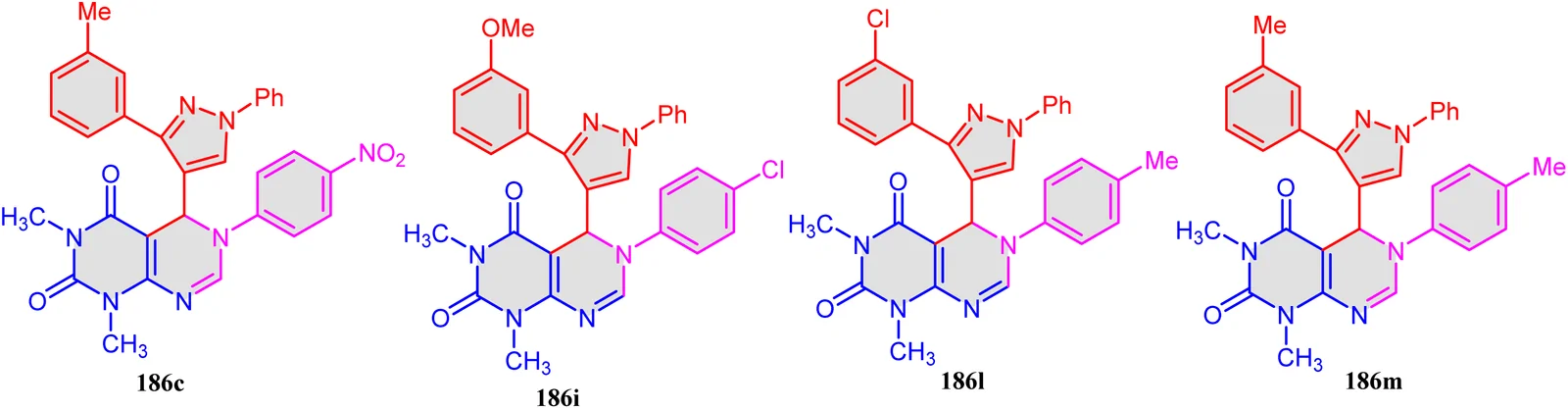
Structures of antibacterial compounds 186c, 186i, 186l and 186m.

Based on the data presented in Table 25, derivatives 186c, 186i, 186l and 186m containing (–NO_2_), (–Cl), (–Me) and (–OMe) groups, respectively, increased the activity considerably (Fig. 13). It was observed that compound 186f (R = 4-Cl, R^1^ = H) has a simple hydrogen atom attached to the basic pyrazolo-pyrimido[4,5-*d*]pyrimidines scaffold, which has neutral properties and may probably contribute to the antibacterial activity. However, compound 186l (R = 4-Me, R^1^ = 4-Cl) contains an electron withdrawing chlorine atom, which probably may contribute to the antibacterial activity.

**Scheme 67:**
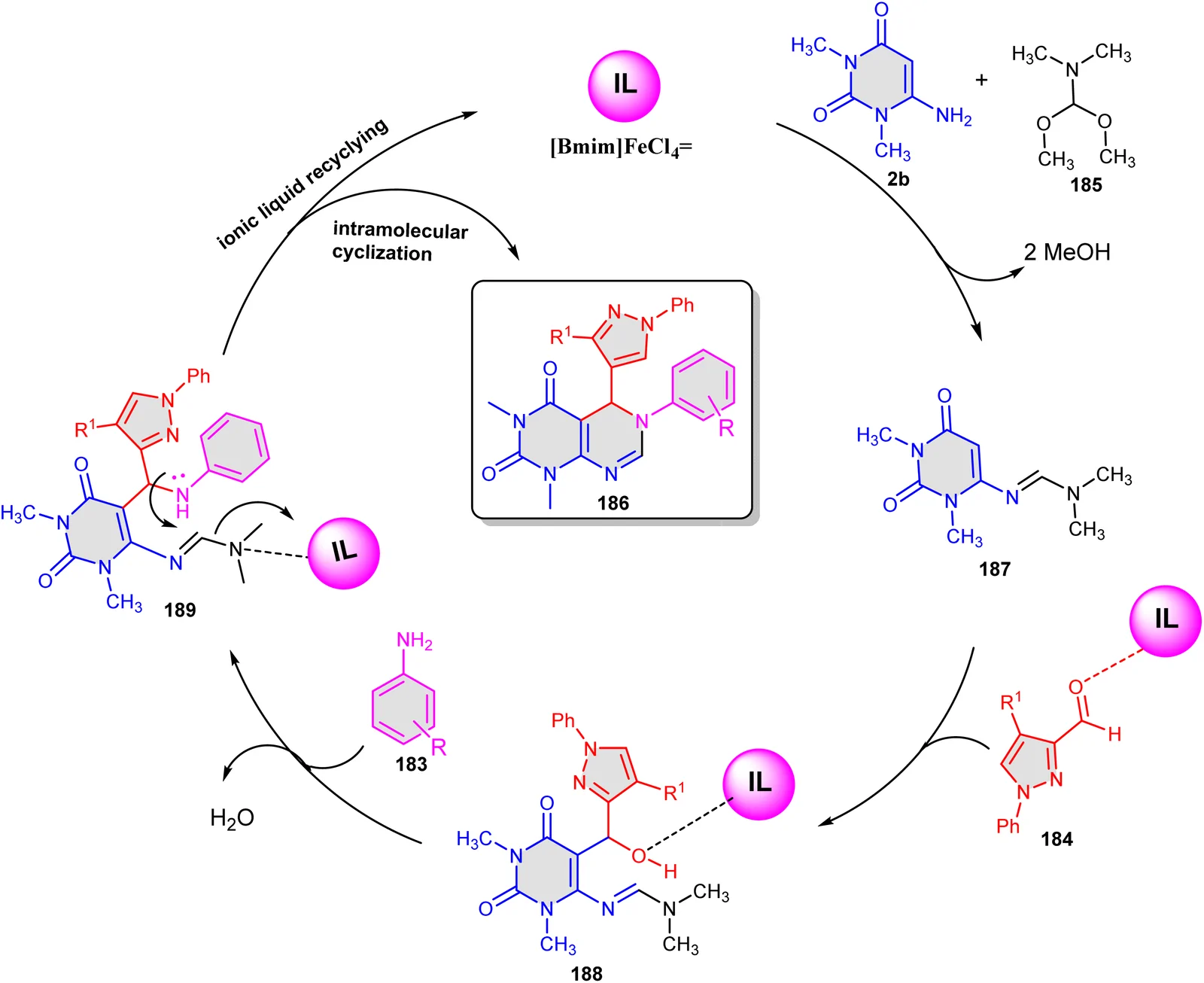
Suggested mechanism for the synthesis of pyrazolo-pyrimido[4,5-*d*]pyrimidines 186a–x.

Scheme 67 illustrates the suggested mechanism for the reaction. As amidine 187 was formed *via* the reaction between 6-amino-1,3-dimethyluracil 2b and *N*,*N*-dimethylformamide dimethyl acetal (185). Next, 1-phenyl-3-(4-substituted-phenyl)-4-formyl-1*H*-pyrazoles 184 underwent a reaction with compound 187 to produce intermediate 188. Intermediate 188 likely reacted with the aromatic amines 183 and lost a molecule of water to give intermediate 189. Finally, a nucleophilic attack occurred in intermediate 189 to the imino carbon atom; accordingly, dimethylamine was removed and subsequently intermolecular cyclized to produce the desired products 186a–x.^148^

**Scheme 68:**
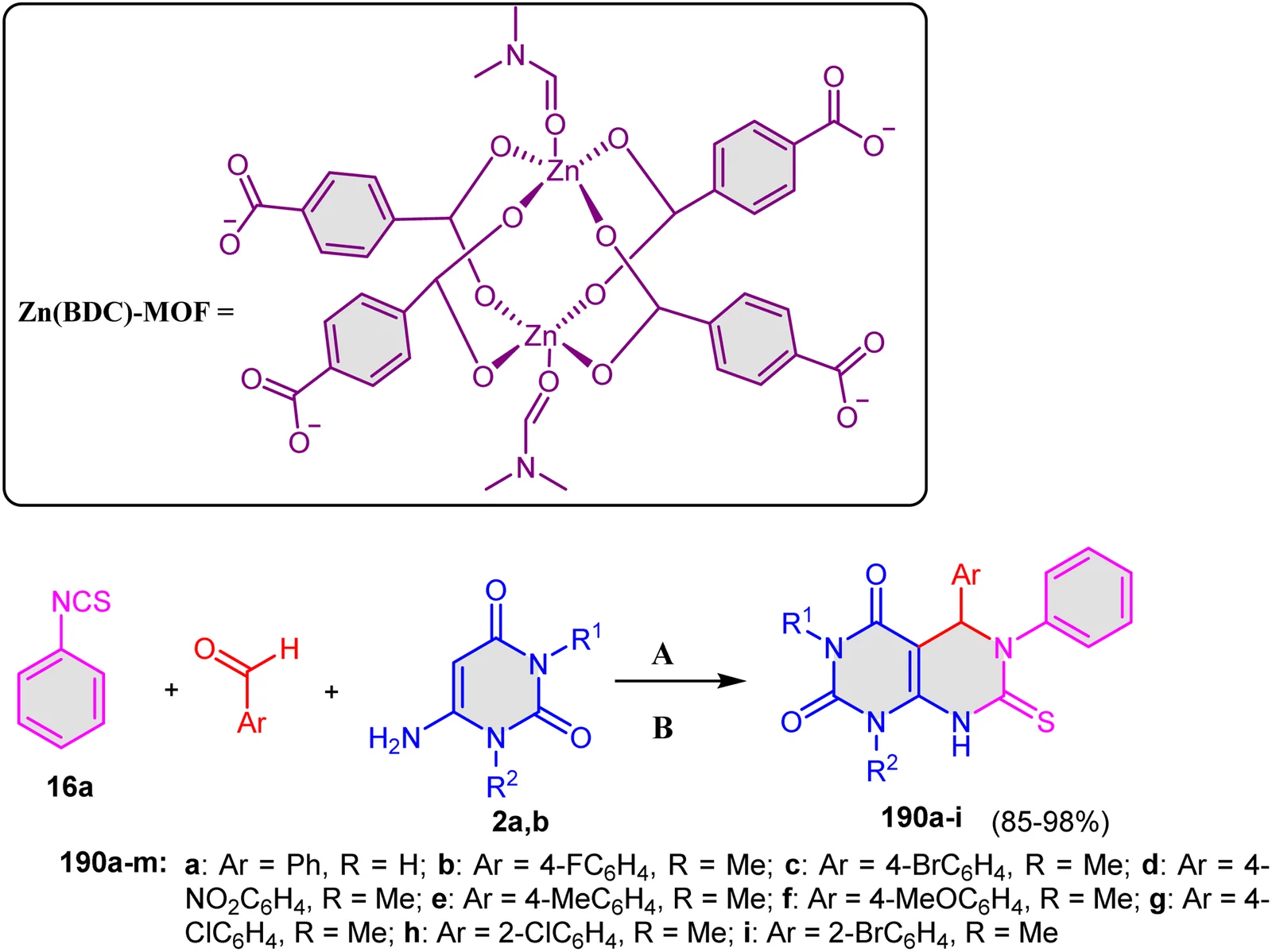
Synthesis of pyrimidopyrimidine derivatives 190a–i. Reagents and conditions: A; Zn(BDC)-MOF, solvent-free; B; ultrasound.

Shirini *et al.*^149^ established a series of pyrimido[4,5-*d*]pyrimidine frameworks 190a–i through a solvent free three-component reaction between phenyl isothiocyanate (16a), aromatic aldehydes, and 6-aminouracil derivatives 2a,b under US irradiation, using Zn(BDC)-MOFs-(BDC,1,4-benzenedicarboxylic acid) as a catalyst. MOFs (metal–organic frameworks) were utilized as a catalyst in 5–10 min (Scheme 68). The final products 190a–i were formed in high yields (85–98%).^149^

**Scheme 69:**
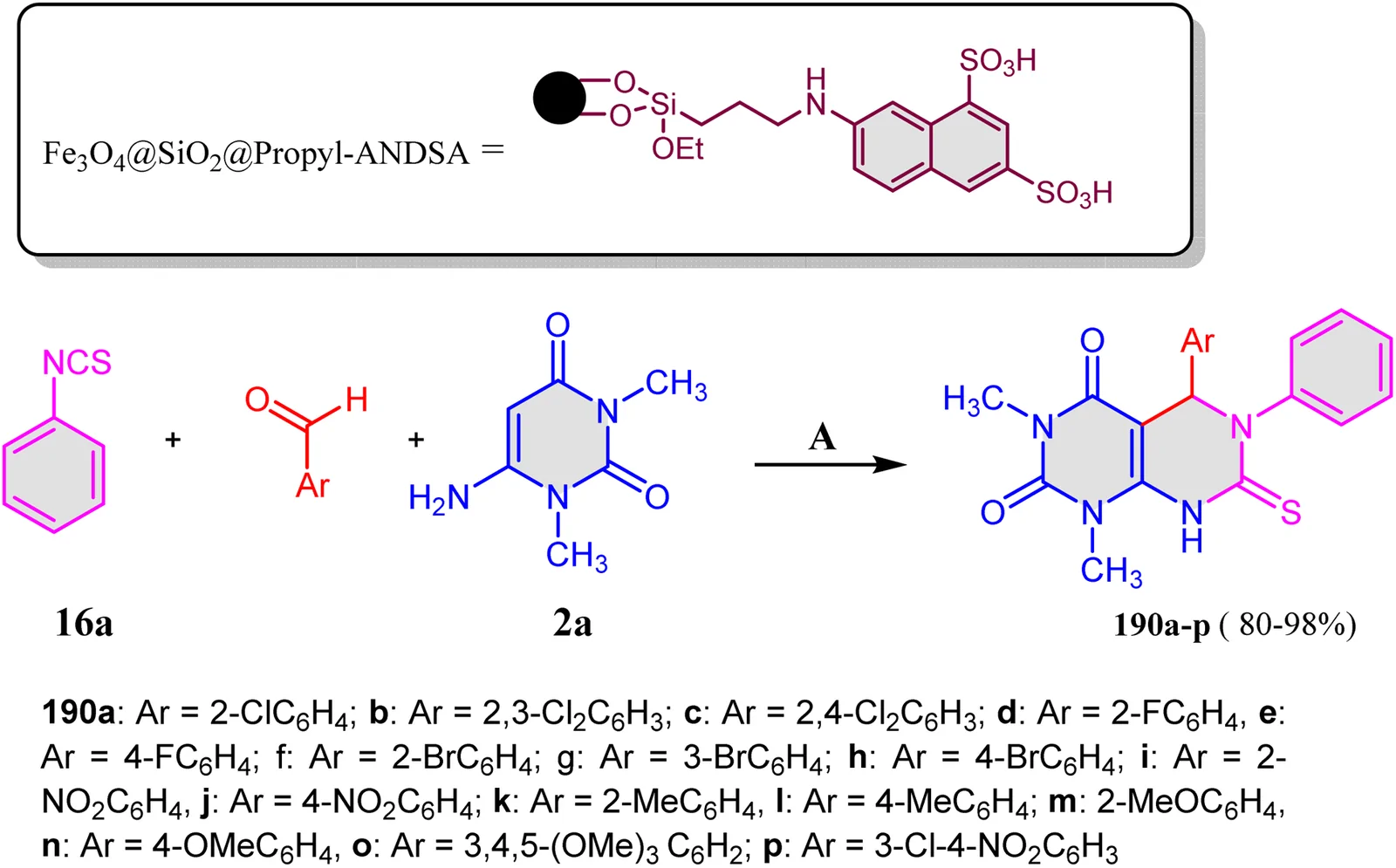
One-pot multicomponent reaction for the synthesis of pyrimido[4,5-*d*]pyrimidines 190a–p. Reagents and conditions: A; Fe_3_O_4_@SiO_2_@Propyl-ANDSA, H_2_O/reflux.

Additionally, Ghorbani-Vaghei *et al.*^150^ utilized 7-aminonaphthalene-1,3-disulfonic acid (ANDSA)-functionalized magnetic Fe_3_O_4_@SiO_2_ particles as a catalyst in the formation of pyrimido[4,5-*d*]pyrimidine derivatives 190a–p (Scheme 69).^150^

**Scheme 70:**
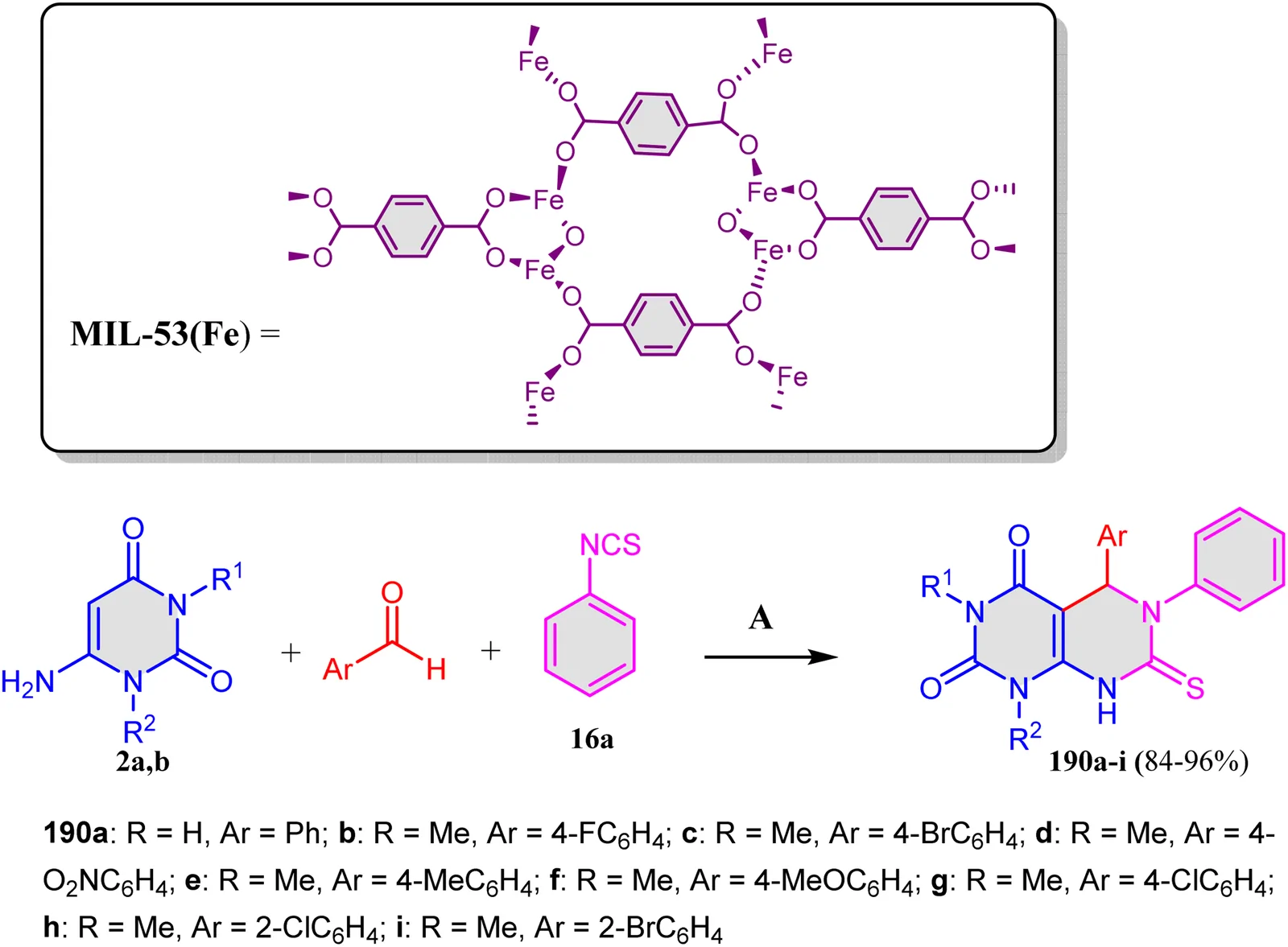
Synthesis of pyrimido[4,5-*d*]pyrimidine 190a–i. Reagents and conditions: A; MIL-53(Fe), solvent free, 110 °C.

Abdollahi-Basir *et al.*^35^ also synthesized a diversity of pyrimido[4,5-*d*]pyrimidine scaffolds 190a–i*via* solvent free reaction between 6-aminouracils 2a,b, aromatic aldehydes and isothiocyanate 16a, and MIL-53(Fe) was employed as a catalyst at 110 °C (Scheme 70).

**Scheme 71:**
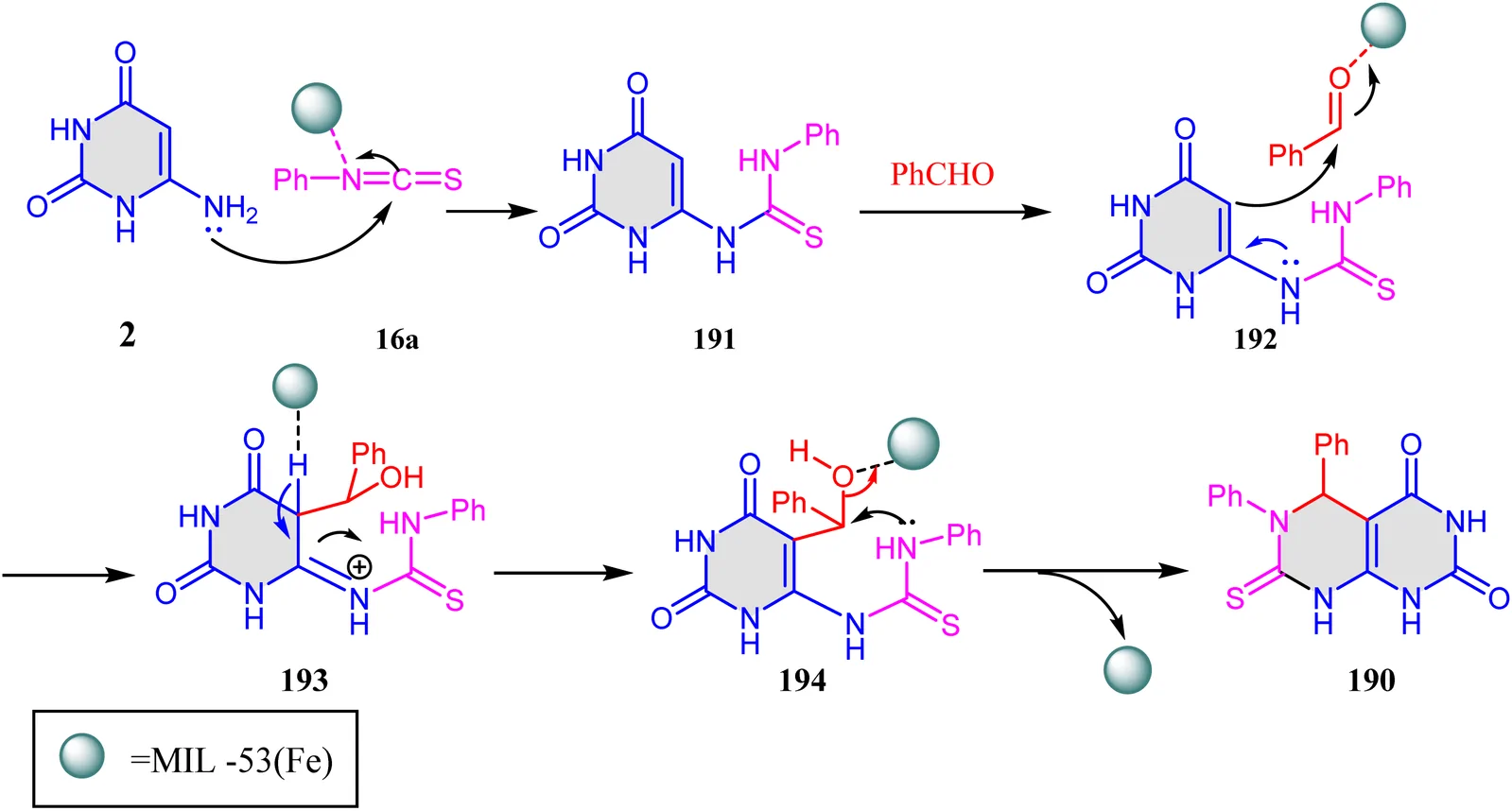
Proposed mechanism for the synthesis of compounds 190a–i catalyzed by MIL-53(Fe).

The suggested mechanism for the synthesis of 192 is shown in Scheme 71.^35^ Initially, 6-aminouracil 2a interacted *in situ* with phenyl isothiocyanate (16a) to produce intermediate 191. Subsequently, with the MIL-53(Fe) present, intermediate 192 underwent a nucleophilic attack on the aldehyde to obtain intermediate 193, which was converted into 194*via* a hydrogen shift. After dehydration and intermolecular cyclization, the final product 190 was formed.^35^

**Scheme 72:**
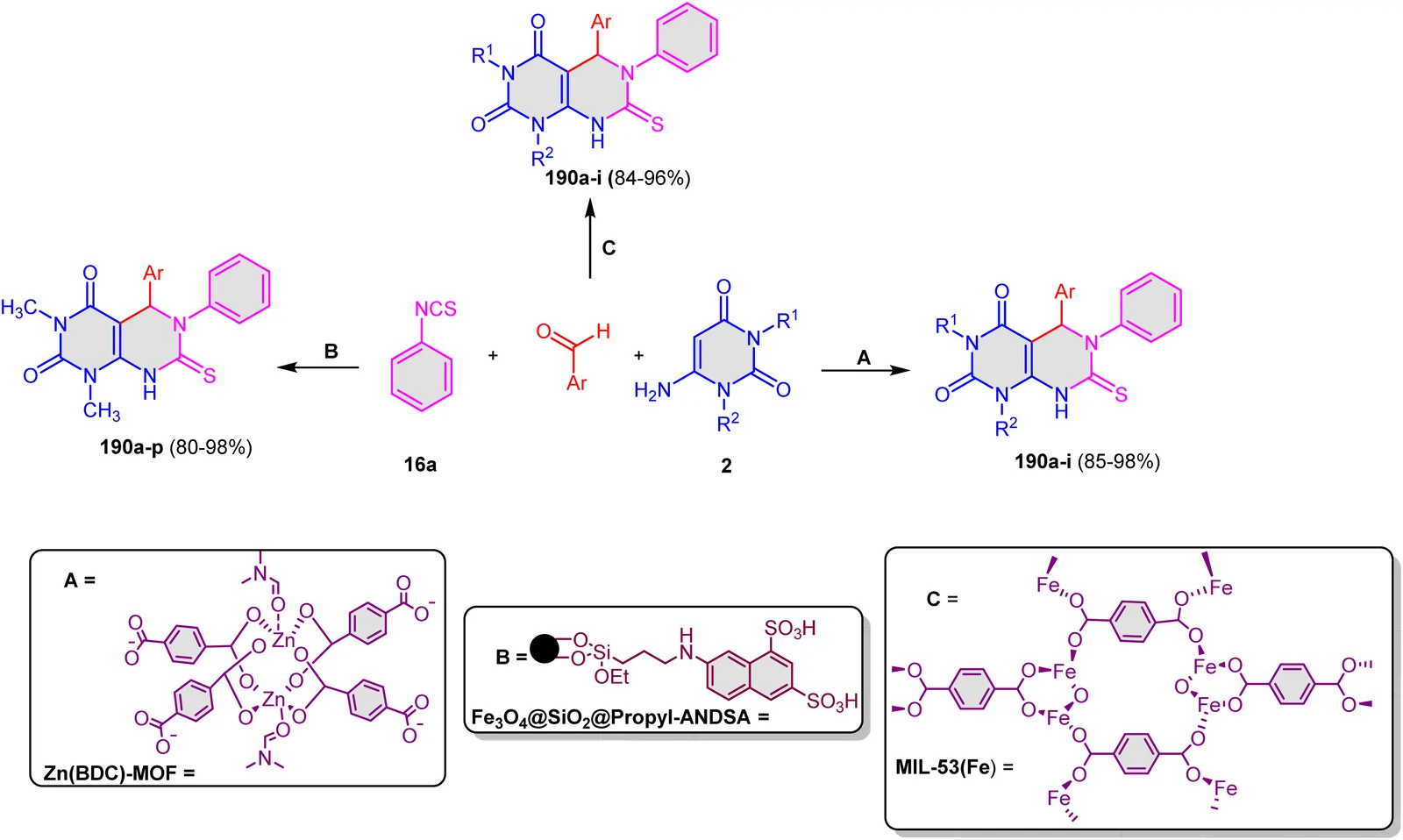
Synthetic pathways for compound 190 under the conditions mentioned in Schemes 28–32, 34, 36, and 39.

The reaction conditions shown in Schemes 66–68 depict comparable conversion under various reaction conditions, which are summed together in Scheme 72. The reaction of aminouracil derivative 2 with aromatic aldehydes and phenyl isothiocyanate (16a), catalyzed by either A, B, or C, produced comparable products 190 in very good to excellent yields. Scheme 70 shows the same type of conversion under different reaction conditions. It is important to note that catalysis with catalyst A can be applied generally to various uracil derivatives 2. In addition, methods employing catalysis by A perform methods B or C in the yields of the obtained products under the preceding conditions (Scheme 72).

**Scheme 73:**
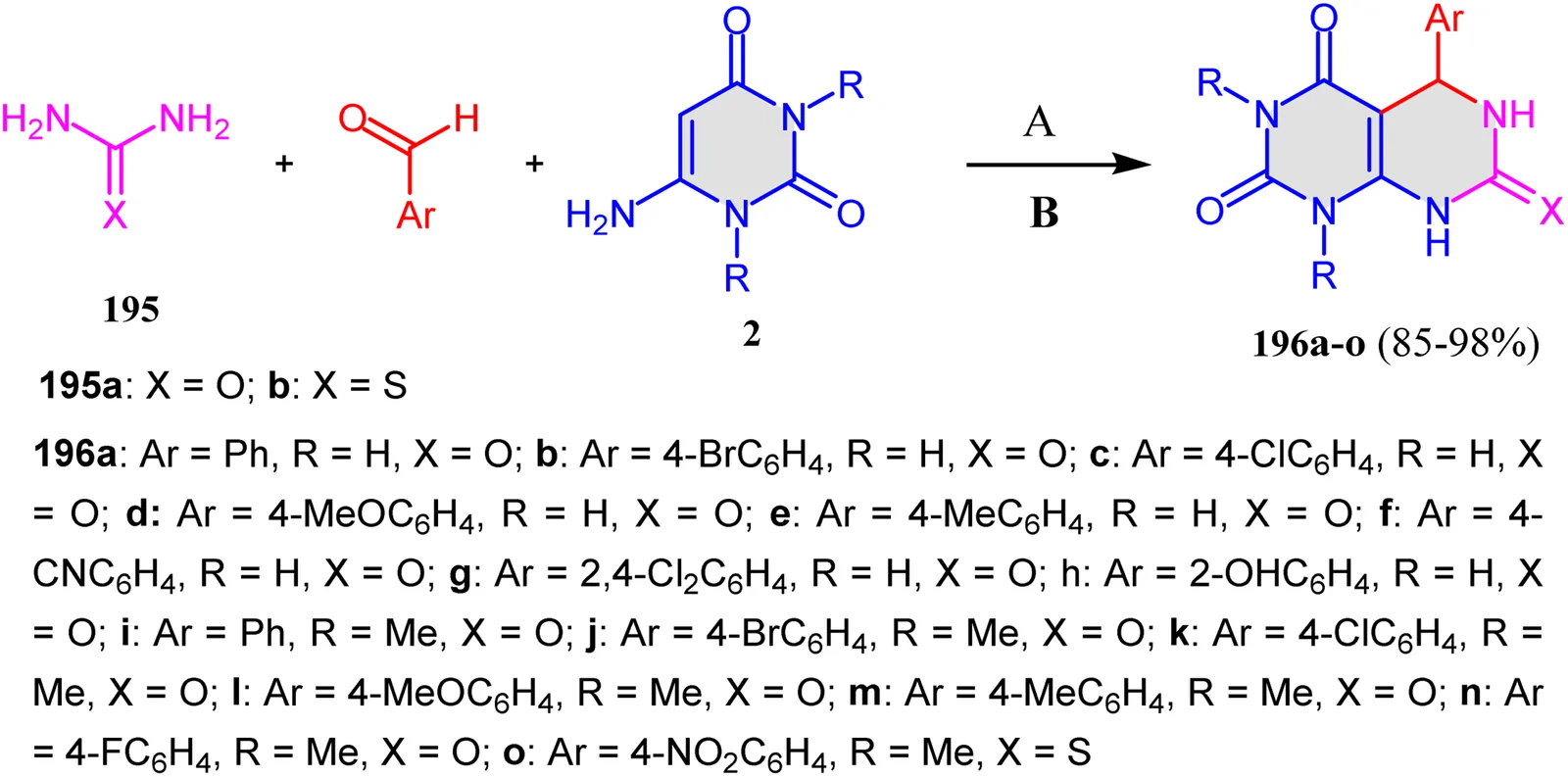
Ultrasound irradiation synthesis of pyrimidopyrimidines 196a–o. Reagents and conditions: A; BAIL@UiO-66, EtOH/r.t, US irradiation.

A series of pyrimidopyrimidines 196a–o were obtained in high yields (85–98%) *via* a multi component reaction between urea derivatives 195a,b and substituted aromatic aldehydes, and 6-aminouracils 2. The reaction was performed in EtOH at room temperature under ultrasound irradiation and catalyzed by TEDA-BAIL@UiO-66 (Scheme 73).^151^

**Scheme 74:**
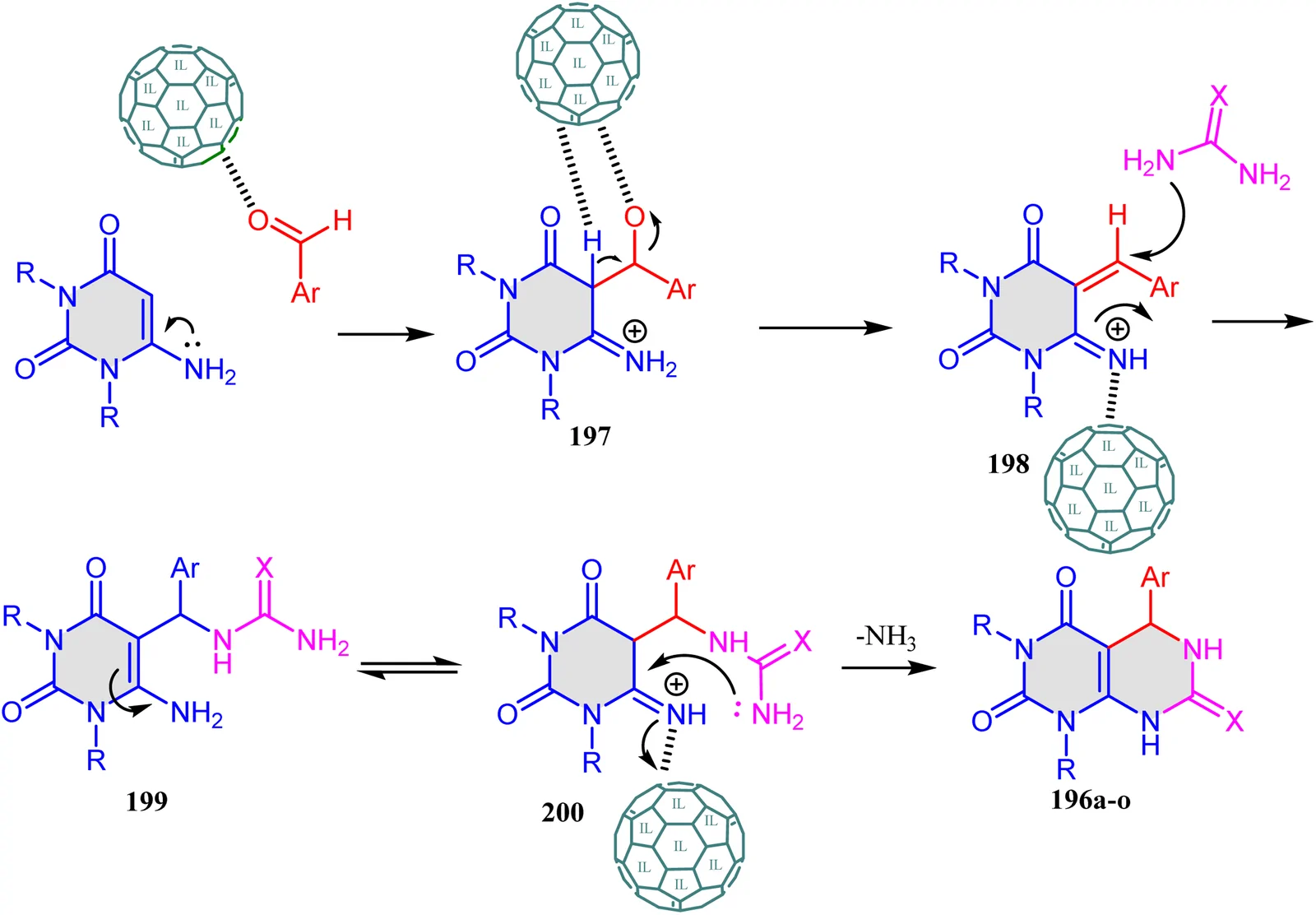
Suggested mechanism for the synthesis of 196a–o.

The suggested mechanism for the formation of pyrimido[4,5-*d*]pyrimidine 196a–o is shown in Scheme 74. The catalyst TEDA-BAIL@UiO-66 promoted the reaction as it acts as a Brønsted acid and enhances the aldehyde's carbonyl group's electrophilicity by releasing a proton. Next, Knoevenagel condensation between 6-aminouracil 2 and aromatic aldehydes afforded intermediate 197, which was converted into alkene 198. Following that, Michael's addition of urea or thiourea 195a,b to alkene 198 led to the formation of intermediate 199. A proton shift occurred to produce intermediate 200. Finally, intermolecular cyclization of 200, followed by the elimination of a molecule of NH_3_, gave the final products 196a–o (Scheme 74).^151^

#### 2.2.7. Synthesis of pyrano-pyrimidine derivatives

**Scheme 75:**
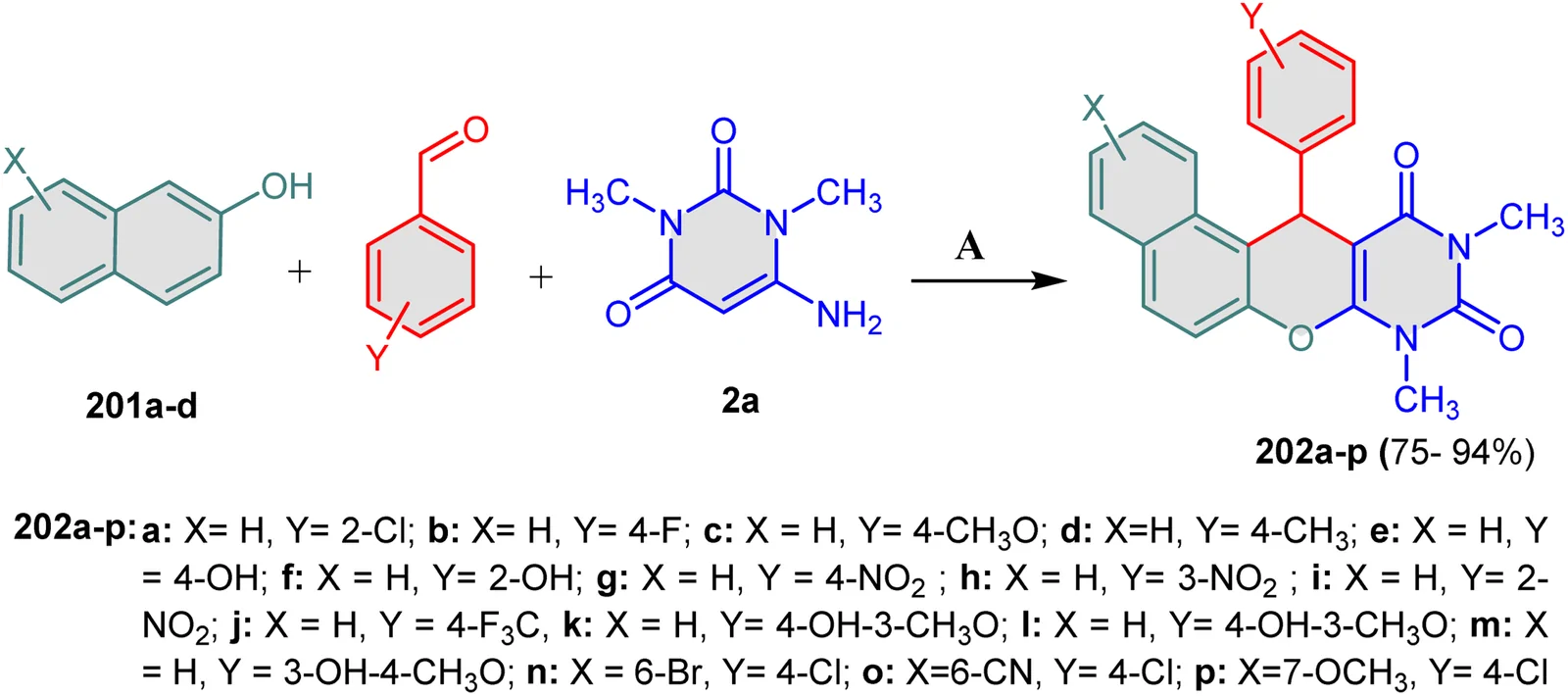
Formation of chromeno[2,3-*d*]pyrimidines 202a–p. Reagents and conditions: A = ChCl : α-CAA (1 : 1), 90 °C, 2 h.

**Scheme 76:**
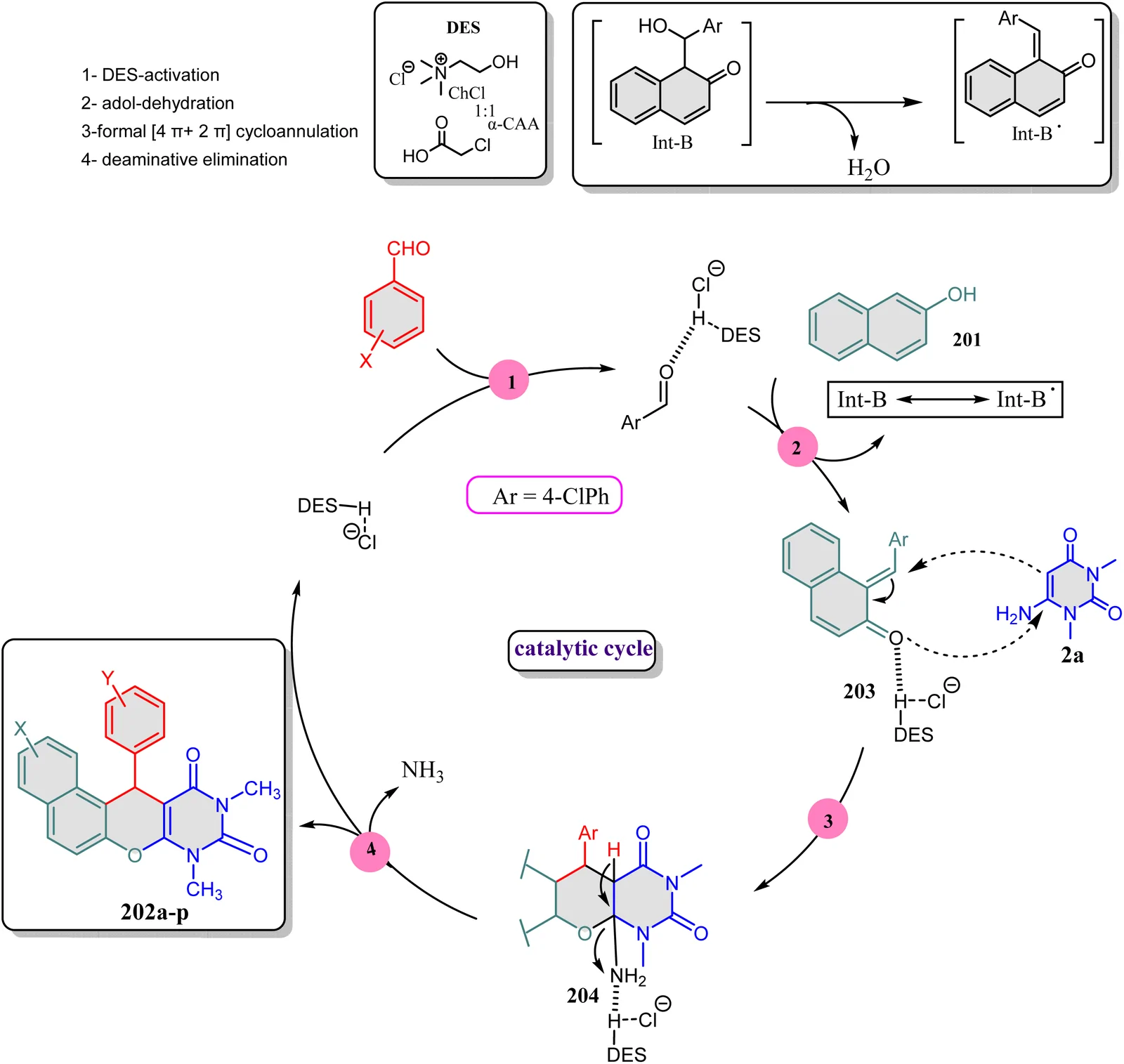
Reaction mechanism for the synthesis of chromeno[2,3-*d*]pyrimidines 202a–p.

Recently, in 2024, Das *et al.*^33^ designed substituted chromeno[2,3-*d*]pyrimidine derivatives 202a–p through the reaction between β-naphthol 201, 6-amino-1,3-dimethyluracil (2a), and aromatic aldehydes in a deep eutectic solvent. The target compounds were produced in (75–94%) yields (Scheme 75). The suggested mechanism for the synthesis of compounds 202a–p is depicted in Scheme 76. Initially, the reaction proceeded by activating an aldehyde in a three-component coupling reaction with DES, which increased the aldehydic functional group's electrophilicity. After that, a regioselective nucleophilic interaction between the pi bond of β-naphthol 201 and the activated aldehyde resulted in the creation of a conjectured intermediate 203. The highly reactive *o*-quinone methide intermediate (B′) was subsequently formed as a result of further dehydration of this intermediate (Scheme 76). B′ is a good hetero-diene for [4 + 2] because of its prolonged conjugation and electron-deficient exocyclic olefinic activity reactions of cycloaddition. After that, intermediate B′ was effectively captured by 6-amino-1,3-dimethyl uracil 2b by a formal [4 + 2] process of hetero-cycloannulation, causing the production of intermediate 204. Finally, the final products 202a–p were obtained *via* DES-mediated deamination of intermediate 204, releasing the active DES for the next catalytic cycle.^33^

The formed products 202a–p exhibit a promising antiproliferative activity against cancer cell lines. Therefore, with moderate to good IC_50_ values, it shows promise as a possible anticancer treatment candidate for hepatocellular and breast cancer. Compound 202m (4-OH-3-MeO-Ph) and 202l (3-OH-4-MeO-Ph) displayed significant inhibitory effects, with IC_50_ values of 31.38 and 28.21 mM against MCF7 cells, respectively. However, other derivatives such as phenyl, tolyl, and trifluoromethylphenyl exhibited approximately 5-fold less efficacy compared to compound 202m and compound 202l. Compounds 202a–n were also screened against HepG2; compound 202m also showcased commendable cytotoxic activity against HepG2, with an IC_50_ value of 46.39 mM. Compound 202f (X = H, Y = 4-OH) displayed moderate cytotoxicity with an IC_50_ value of 64.79 mM against MCF7. Moreover, compounds 202n (X = Y = H), 202l, and 202e (X = H, Y = 4-Me) displayed relatively low toxicity, presenting IC_50_ values of 91.06, 88.53, and 91.05 mM against HepG2 cell lines, respectively.^33^

#### 2.2.8. Synthesis of phenazines and pteridine derivatives

**Scheme 77:**
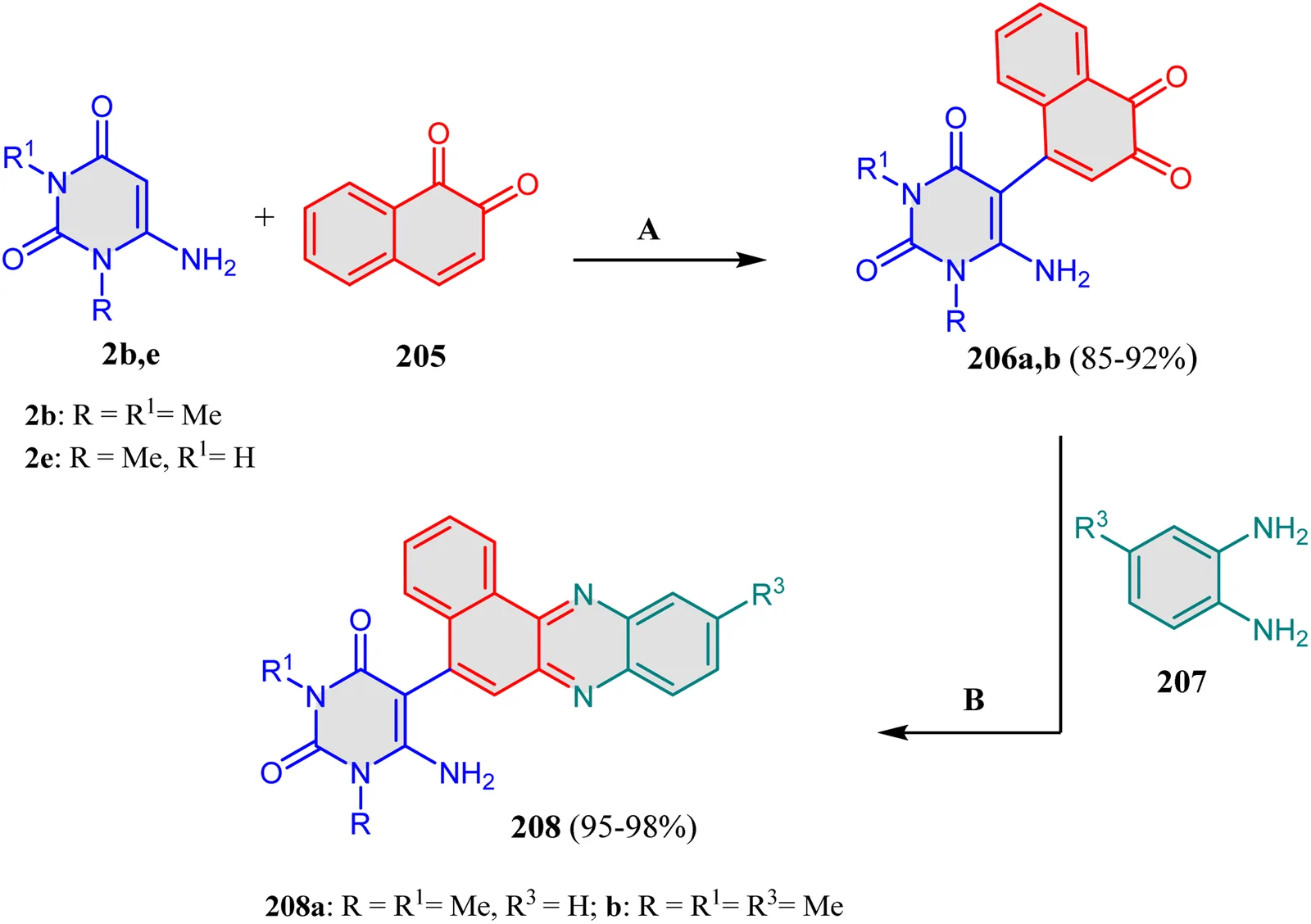
Synthesis of phenazine scaffold 208. Reagents and conditions: A; DMSO, 70 °C. B; CHCl_3_, reflux, 1 h.

Jamaledini *et al.*^34^ synthesized phenazine compounds containing 6-aminouracil moieties through a multi step reaction. At the beginning, 6-aminouracils 2 reacted with naphthalene-1,2-dione 205 in DMSO at 70 °C to form compounds 206a,b in 85–92% yields. Following that, a condensation reaction between compound 206a,b and *o*-phenylenediamine 207a–d derivatives in CHCl_3_ for 1 h afforded the target compounds of phenazine 208 in excellent yields (95–98%) (Scheme 77).

**Scheme 78:**
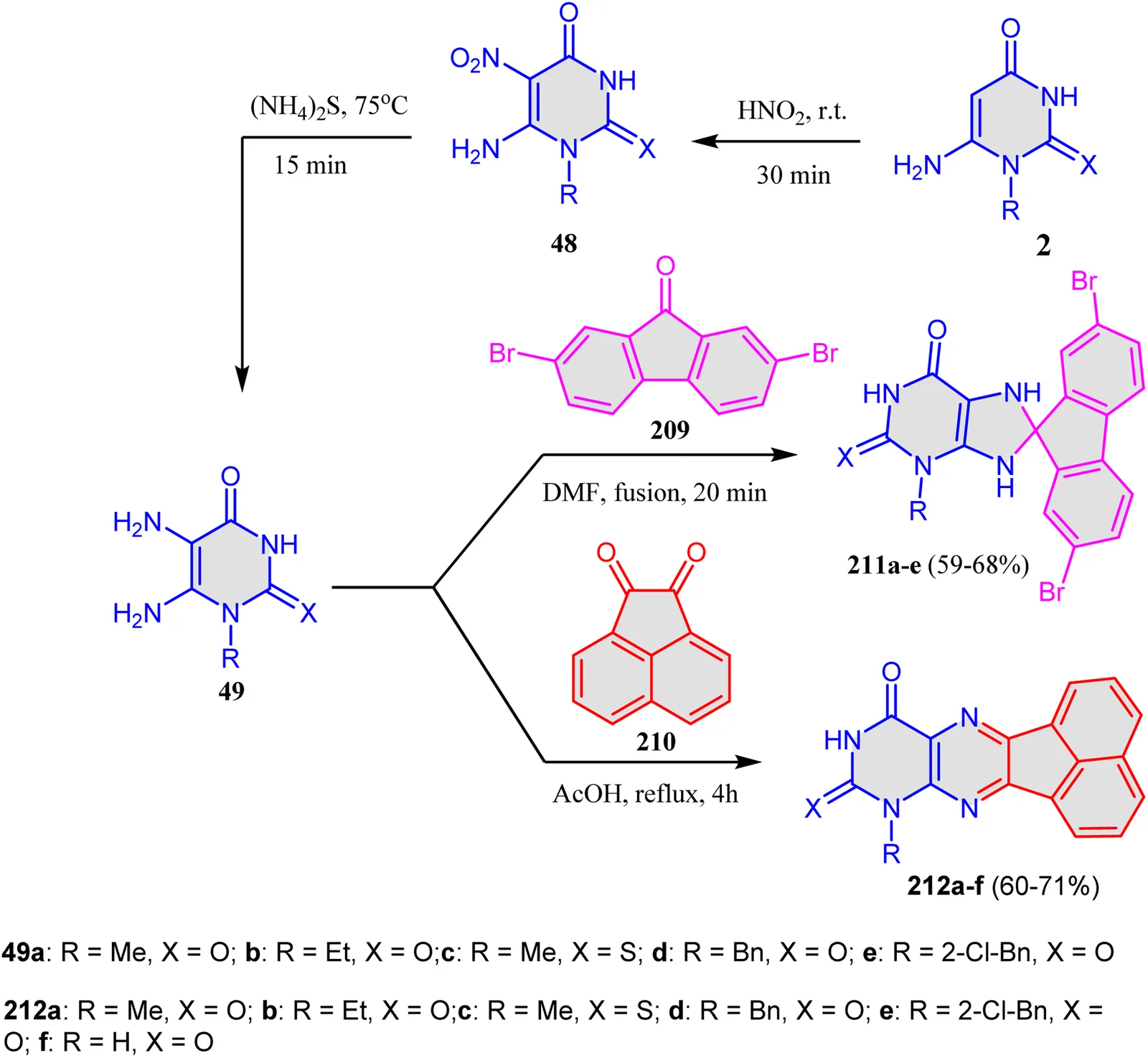
Reaction of 5,6-diaminouracils 49 with 2,7-dibromo-9*H*-fluoren-9-one (209) and acenaphthoquinone 210.

The reaction of 5,6-diamnouracils 49a–e with 2,7-dibromo-9*H*-fluoren-9-one (209) in DMF for 20 min gave spiro compounds 211a–e in 59–68% yields (Scheme 78).^152^ On the other hand, 49 reacted with an acenaphthoquinone (210) in AcOH for 6 h to afford acenaphtho[1,2-*g*]pteridines 212a–f in yields 60–71% (Scheme 78).^152^ Most of the compounds displayed significant antiproliferative activity on the tested cell lines (Tables 26 and 27). Compounds 211a, 211e, and 212e of scaffolds based on pteridine and purines were identified as the most potent hits in anti-proliferative screening, with GI50 values of 38 nM, 46 nM, and 44 nM, respectively. Compounds 211a, 211e, and 212e showed promising EGFR inhibitory activity, with IC_50_ values of 87 nM, 98 nM, and 92 nM, respectively, when compared to erlotinib's IC_50_ value of 80 nM. According to the results, compound 211a (R = Me, X = O) has the most potent anti-proliferative activity and is determined to be the most effective EGFR suppressor, with an IC_50_ value of 87 ± 07 nM, which is equal to erlotinib (IC_50_ = 80 nM).

**Table 26:** IC_50_ values of compounds 211a–e and 212a–f

Compd	R	X	Cell viability%	Anti-proliferative activity IC_50_ ± SEM (nM)				
				A-549	MCF-7	Panc-1	HT-29	Average (GI_50_)
211a	Me	O	89	36 ± 3	40 ± 3	38 ± 3	38 ± 3	38
211b	Et	O	91	85 ± 8	88 ± 8	86 ± 8	86 ± 8	86
211c	Me	S	90	98 ± 9	103 ± 10	100 ± 9	102 ± 10	101
211d	Bn	O	91	52 ± 5	55 ± 5	54 ± 5	52 ± 5	53
211e	2-Cl-Bn	O	89	44 ± 4	48 ± 4	46 ± 4	46 ± 4	46
212a	Me	O	92	56 ± 5	60 ± 6	58 ± 5	58 ± 5	58
212b	Et	O	90	64 ± 6	69 ± 6	66 ± 6	68 ± 6	67
212c	Me	S	89	80 ± 8	83 ± 8	80 ± 8	80 ± 8	81
212d	Bn	O	91	90 ± 9	96 ± 9	90 ± 9	92 ± 9	92
212e	2-Cl-Bn	O	88	41 ± 4	46 ± 4	44 ± 4	44 ± 4	44
212f	H	O	90	76 ± 7	79 ± 7	75 ± 7	75 ± 7	76
Erlotinib	—	—	ND	30 ± 3	40 ± 3	30 ± 3	30 ± 3	33

**Table 27:** IC_50_ values of compounds 211a, 211d, 211e, 212a, and 212e against EGFR and BRAF^V600E^

Compd	EGFR inhibition IC_50_ ± SEM (nM)	BRAF^V600E^ inhibition IC_50_ ± SEM (nM)
211a	87 ± 07	92 ± 07
211d	105 ± 09	164 ± 15
211e	98 ± 08	137 ± 12
212a	112 ± 10	183 ± 17
212e	92 ± 07	109 ± 09
Erlotinib	80 ± 05	60 ± 05

**Scheme 79:**
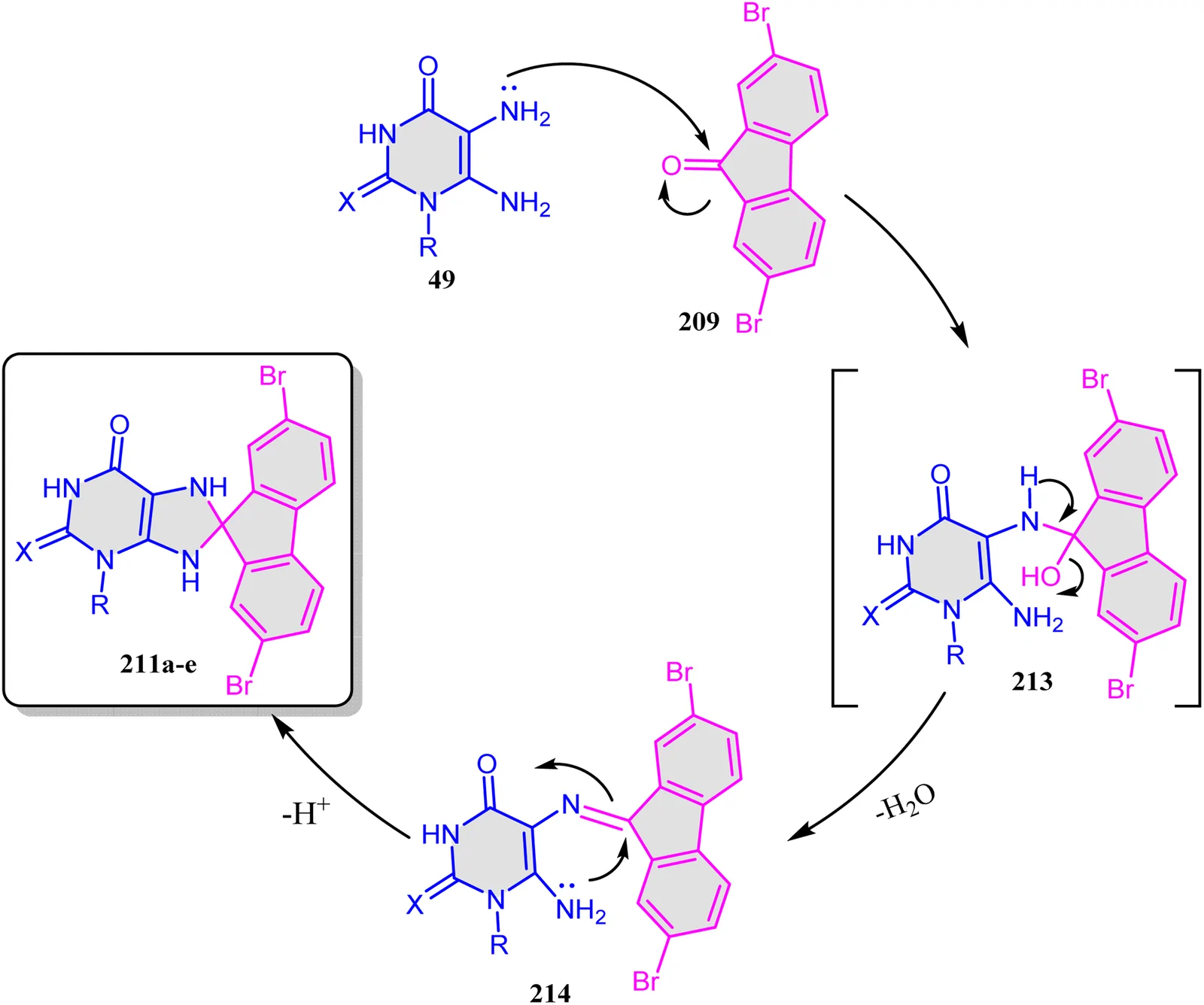
Proposed mechanism for the synthesis of 211a–e.

The mechanism describes the reaction of 49 with 209, as shown in Scheme 79.^151^ The reaction started with the nucleophilic attack of the amino group of diaminouracil 49 to the carbonyl group of 2,7-dibromo-9*H*-fluoren-9-one (209), adduct 213 was then produced, which lost a molecule of water to give adduct 214. An intermediate 214 then underwent intramolecular aza-Michael addition, leading to the production of compounds 211a–e.^152^

##### 2.2.8.1 Structure activity relationship

**Fig. 14:**
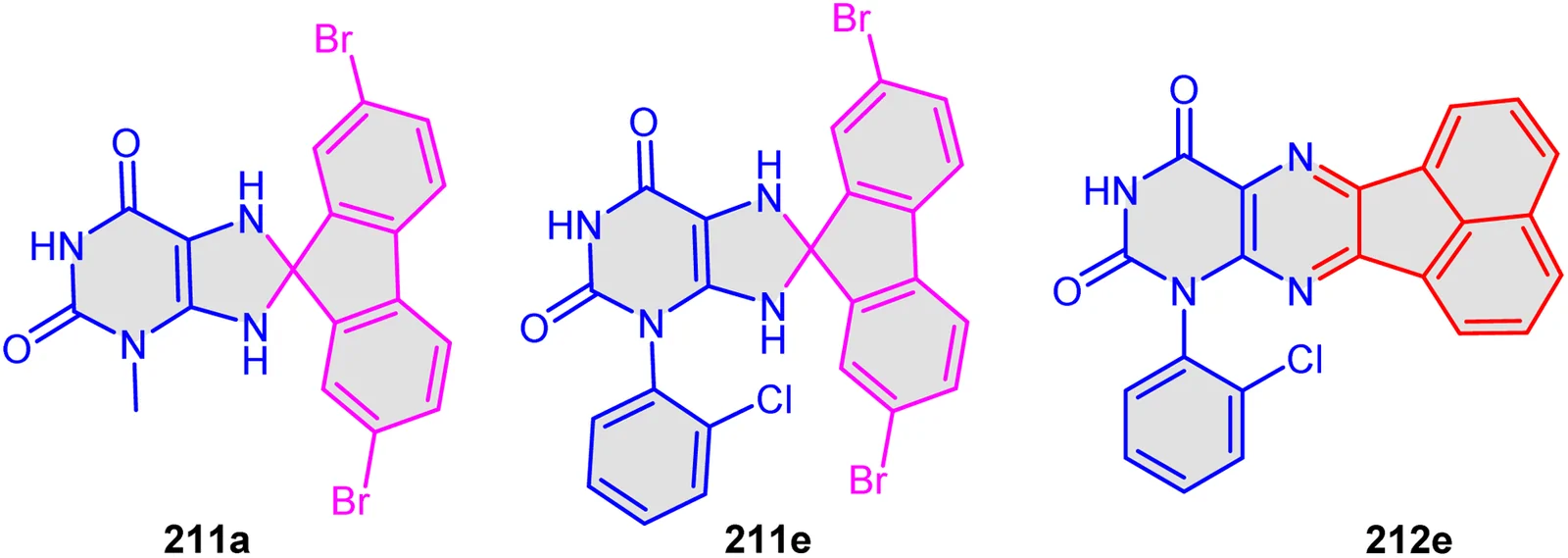
Structures of the most anti-proliferative pteridine and purine molecules.

According to the data presented in Tables 26 and 27, it was concluded that in the case of a replacement of the oxygen atom with a sulfur atom in compound 211c (R = Me, X = S), the antiproliferative activity decreased compared to compound 211a, indicating that the oxygen atom at position 2 is important owing to its anti-proliferative properties. The results also imply that the antiproliferative activity of compound 211 is significantly influenced by the kind of third-position substitution, with activity rising in the following order: methyl > 2-chlorobenzyl > benzyl > ethyl. Correspondingly, pteridine derivatives 212a–f demonstrated a moderate antiproliferative effect. Compound 212e (R = 2-chlorobenzyl, X = O) was the most effective compound in this series against the screened cell lines, but it was 1.3 times less effective than the standard “erlotinib”. However, compound 212e was similar to its analog 211e, and compound 212a (R = Me, X = O) was 1.5 times less effective than its analog 211a (R = Me, X = O) and showed mild anti-proliferative effects. Moreover, antiproliferative activity decreased when a sulfur atom was substituted for the oxygen atom, as in compound 212c (R = Me, X = S). Compounds 212d (R = benzyl, X = O) and 212e (R = 2-chlorobenzyl, X = O), in which the methyl group in 212a was replaced by a benzyl and 2-chlorobenzyl moiety, respectively, demonstrated a significant difference in anti-proliferative activity. Compound 212d demonstrated a marked decrease in anti-proliferative activity, being 1.5-fold less effective than 212a, whereas 212e outperformed 212a in activity. Fig. 14 shows the structure of the most anti-proliferative pteridine and purine molecules.

##### 2.2.8.2 Synthesis of pyridotriazolothiazolopyridopyrimidine

**Scheme 80:**
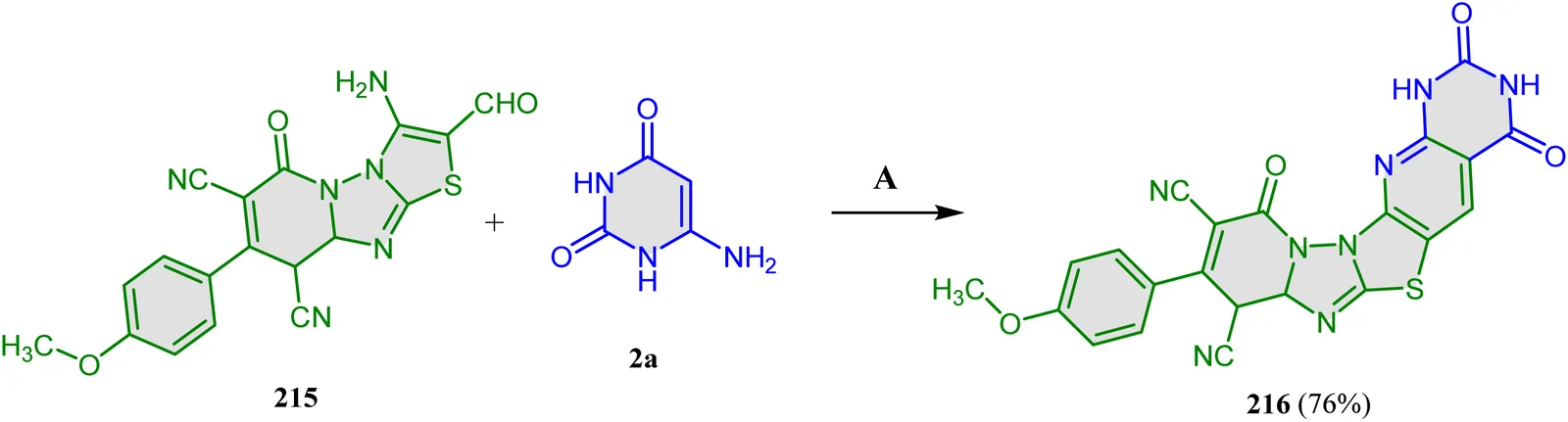
Synthesis of pyridotriazolothiazolopyridopyrimidine 216. Reagents and conditions: A; DMF/DBU, reflux, 30 min.

The reaction of 3-amino-2-formyl-8-(4-methoxyphenyl)-6-oxo-9,9*a*-dihydro-6*H*-thiazolo-[3′,2′:2,3][1,2,4]triazolo[1,5-*a*]pyridine-7,9-dicarbonitrile (215) with 6-aminouracil (2a) in DMF containing DBU at reflux temperature for 30 min yielded compound 216 in 76% yield (Scheme 80). The obtained compound pyridotriazolothiazolopyridopyrimidine 216 was screened for antimicrobial activity and revealed high efficiency against yeast fungus and Gram-negative bacteria.^153^

#### 2.2.9. Synthesis of pyrimidine/isoindoline-1,3-diones

**Scheme 81:**
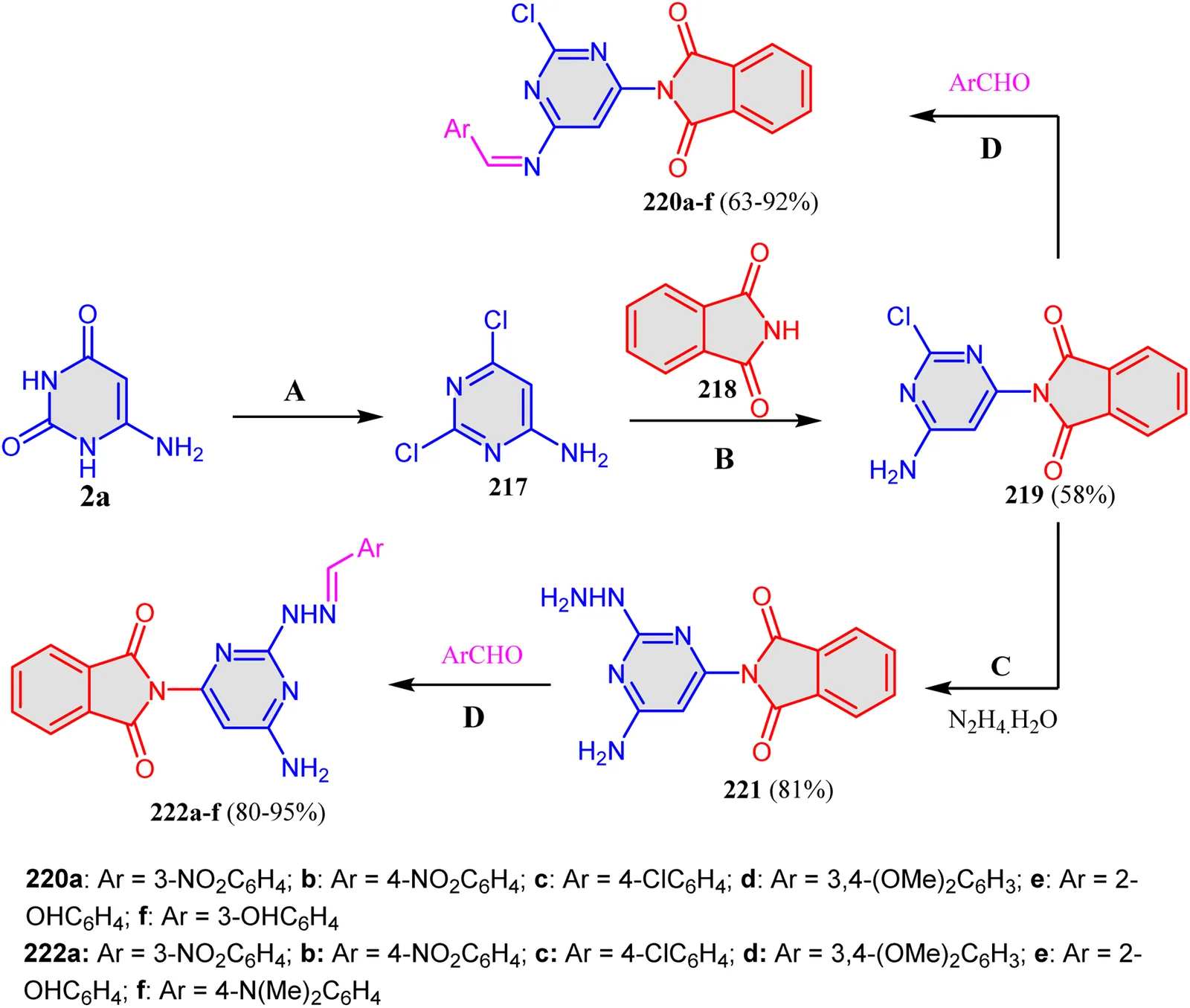
Synthesis of Schiff bases 220a–f and 222a–f. Reagents and conditions: A; POCl_3._B; EtOH, pip. C; EtOH, Et_3_N, 9 h, rt. D; EtOH, Et_3_N, 6 h, rt.

Very recently, in 2025, Shehta *et al.*^154^ developed a multi-step reaction for the synthesis of pyrimidine/isoindoline hybrids. First, they prepared 2,6-dichloropyrimidin-4-amine 217 by treating 6-aminouracil 2a with POCl_3_. Next, they reacted isoindoline-1,3-dione with 218 in EtOH to give compound 219, which reacted with different aromatic aldehydes to obtain the corresponding Schiff bases 220a–f. However, when compound 219 was treated with hydrazine hydrate, the reaction proceeded to give 2-(6-amino-2-hydrazineylpyrimidin-4-yl)isoindoline-1,3-dione (221), which was allowed to react with different aromatic aldehydes to obtain 2-(6-amino-2-(2-(arylmethylene)hydrazineyl)pyrimidin-4-yl)isoindoline-1,3-dione 222a–f (Scheme 81). The authors screened the synthesized compounds 222a–f and 222a–f as potential antitubercular agents against multi-drug-resistant (MDR) and extra-drug-resistant (XDR). Compounds 220b–d, 222a, and 222c–e were inactive against *M. tuberculosis*. Moreover, compounds 220a (Ar = 3-NO_2_Ph), 220e (Ar = 2-OH-Ph), 220f (Ar = 3-OH-Ph), and 222b (Ar = 4-NO_2_-Ph) showed moderate activity (MIC 15.63–31.25 μg mL^−1^) compared to the reference drug. Compounds 220a and 222f (Ar = 4-N(Me)_2_Ph) displayed inhibition of MDR strain with MIC values equal 7.81 and 1.95 μg mL^−1^ and for XRD 15.63 and 7.81 μg mL^−1^, respectively. However, compounds 220a and 222f inhibited the mycobacterial InhA enzyme with IC_50_ = 1.64 ± 0.069 and 0.717 ± 0.033 μM (Tables 28 and 29), respectively, in comparison with isoniazid (INH) with IC_50_ = 0.323 ± 0.014 μM, as a standard drug.

**Table 28:** Antitubercular activity of 220a–f and 222a–f

Compd	R	*M. tuberculosis* MIC (μg mL^−1^)		
		Sensitive	MDR	XDR
220a	3-NO_2_	0.98	7.81	15.63
220b	NO	7.81	31.25	NA
220c	Cl	31.25	NA	NA
220d	3,4-(OMe)_2_	31.25	125	NA
220e	OH	3.9	7.81	31.25
220f	OH	3.9	7.81	31.25
222a	NO_2_	7.81	31.25	NA
222b	NO_2_	15.63	62.5	31.25
222c	Cl	62.5	NA	NA
222d	OH	125	NA	NA
222e	OH	125	NA	NA
222f	4-NMe_2_	0.48	1.95	7.81
Isoniazid		0.12	IA	NA

**Table 29:** *In vitro M. tuberculosis* enoyl-acyl carrier protein reductase inhibitory activity of 222f, 220a, and isoniazid

Compd	InhA IC_50_ (mean ± SD) (μM)
220a	1.646 ± 0.069
222f	0.717 ± 0.033
Isoniazid	0.323 ± 0.014

## 3 Conclusion

Due to the biological activity of 5-amino uracil, 6-aminouracil and their derived heterocycles, their synthesis has attracted a great interest in the field of medicinal chemistry. This study includes different methods reported for synthesizing the target molecules in the past 10 years, especially from 2014 to 2024. We also reported on the advantages of catalysis in synthesizing the target compounds using a multi-component reaction (MCR) sequence. Accordingly, we here shed light on the utility of catalysis in MCRs, which would provide the target molecules with excellent yields in addition to short reaction time, cost efficiency, and a simple workup procedure without extra purification techniques. This review also focused on the biological applications of the target molecules since they covered many biological activities, such as anti-cancer, anti-microbial, anti-inflammatory, anti-Alzheimer, and/or anti-tubercular agents.

Due to the therapeutic importance of heterocycles from 5-amino and 6-aminouracils, their synthesis has become valuable in the synthesis of numerous drugs. The synthesis of the former compounds has become more facile due to the utility of various eco-friendly catalysts in multicomponent reactions. Moreover, mild reaction conditions, good to excellent yields, and the absence of tedious separation procedures are the key advantages of their synthesis. The latter would encourage researchers to synthesize former heterocycles, which would lead to interesting biological applications.

## Author contributions

A. A. A. (supervision, conceptualization, writing, editing, and submitting), E. M. O. (writing, editing), S. M. M. (supervision, editing), T. M. B. (supervision, editing), M. A. (editing), K. U. S. (editing), A. H. M. (supervision, editing), all authors have read and agreed to the published version of the manuscript.

## Conflicts of interest

The authors declare no conflict of interest.

## Data Availability

There are no data generated.
